# Diastereoselective Carbocyclization of 1,6-Heptadienes Triggered by Rhodium-Catalyzed Activation of an Olefinic C=H Bond**

**DOI:** 10.1002/anie.201400080

**Published:** 2014-03-14

**Authors:** Christophe Aïssa, Kelvin Y T Ho, Daniel J Tetlow, María Pin-Nó

**Affiliations:** Department of Chemistry, University of LiverpoolCrown Street, L69 7ZD (UK)

**Keywords:** C=H activation, diastereoselectivity, diene, rearrangement, rhodium

## Abstract

The use of α,ω-dienes as functionalization reagents for olefinic carbon–hydrogen bonds has been rarely studied. Reported herein is the rhodium(I)-catalyzed rearrangement of prochiral 1,6-heptadienes into [2,2,1]-cycloheptane derivatives with concomitant creation of at least three stereogenic centers and complete diastereocontrol. Deuterium-labeling studies and the isolation of a key intermediate are consistent with a group-directed C=H bond activation, followed by two consecutive migratory insertions, with only the latter step being diastereoselective.

The metal-catalyzed activation of otherwise inert carbon–hydrogen (C=H) bonds is increasingly recognized as a powerful synthetic method, and numerous transformations involving aromatic C=H bonds have been described.[1] In contrast, besides hydrovinylation reactions using ethylene,[2] the metal-catalyzed functionalization of an olefinic C=H bond with a different alkene has been reported on far fewer occasions.[3] Specifically, besides the addition of 2-isopropenyl-pyridine to 1,5-hexadiene[3h] and the addition of various olefins to 1,3-dienes,[2], [4] the use of α,ω-dienes as functionalization reagents of olefinic C=H bonds has not yet been explored.

In this context, we anticipated that the treatment of 1,6-heptadiene **I** with a rhodium catalyst would lead to the [2,2,1]-cycloheptane derivative **II** by undergoing the following elementary steps (Scheme 1): a) pyridine-directed C=H bond activation, b) migratory insertion of the first terminal olefin into the metal hydride thus generated, c) migratory insertion of the second olefin,[5] and d) final reductive elimination. Significantly, the success of this design would enable a strong increase of molecular complexity, as measured by the number of sp^3^-hybridized stereogenic centers created in the rearrangement. Specifically, it would contrast with the classical metal-catalyzed cycloisomerizations of 1,6-heptadienes, which are moderately diastereoselective, when the two terminal olefins of the substrate are linked by a prochiral tether.[6], [7] Herein, we report that the implementation of our reaction design leads to the formation of **II** as a single diastereoisomer and we propose a refinement of the initially envisioned mechanism.

**Scheme 1:**
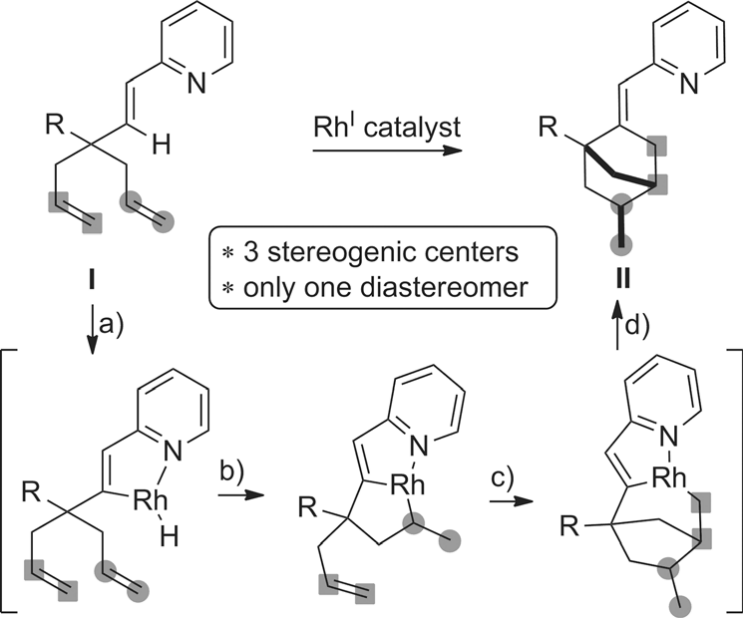
Proposed catalytic cycle for a diastereoselective carbocylization of 1,6-heptadienes into [2,2,1]-cycloheptane derivatives.

Although initial investigations with the model substrate **1 a** validated our reaction design, the desired compound **2 a** was contaminated by **3 a** (Figure 1). This problem was solved by rendering the catalyst cationic through the addition of AgBF_4_.[8] Control experiments confirmed that AgBF_4_ alone cannot act as the catalyst, and that phosphine-free rhodium species are not catalytically competent. Importantly, **2 a** was obtained as a single diastereomer, even in the crude reaction mixtures. The stereochemistry of **2 a** was confirmed by NOESY and by X-ray crystallography of its hydrochloride salt (Figure 1).[9]

**Figure 1:**
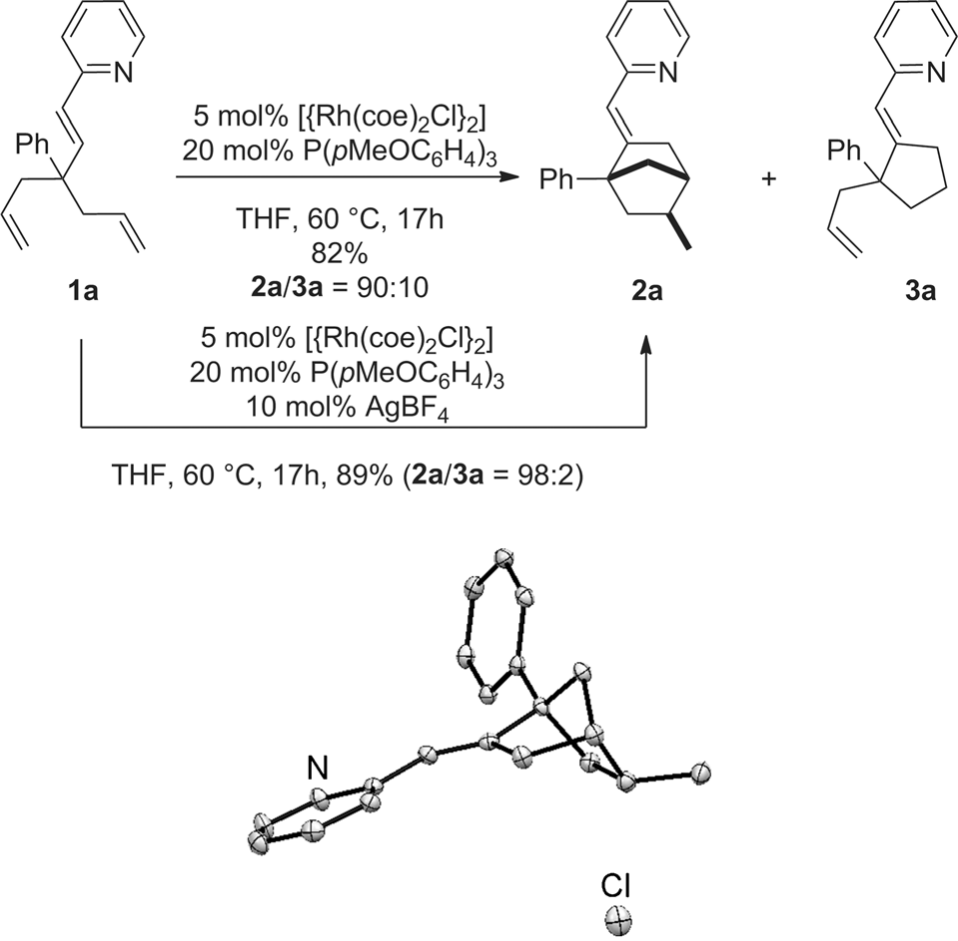
Validation of the reaction design and ORTEP drawing of 2 a⋅HCl. Only the nitrogen and chlorine atoms are labeled for clarity. Thermal ellipsoids are shown at 50 % probability. coe=cyclooctane, THF=tetrahydrofuran.

With these results in hand, we examined the generality of this transformation and observed in all cases the formation of **2** as a single diastereomer in good to excellent yields upon isolation (Scheme 2). The stereochemistry of the compounds **2** was confirmed by NMR spectroscopy and by X-ray crystallography in the case of **2 g** (see the Supporting Information). Naphthyl (**1 b**), electron-poor and electron-rich phenyl (**1 c**–**f**), 1-oxa-3-indenyl (**1 g**), acetal (**1 h**), protected amine (**1 i**), and alkyl (**1 j**–**m**) groups were all tolerated. The selectivity between **2** and **3** remained excellent (≥95:5) except in the case of substrates having a nonbulky substituent (R), such as a linear ether chain (**1 l**) or a simple methyl group (**1 m**). Hence, the Thorpe–Ingold effect appears important for the selective formation of **2**. Further confirming this rationale, the reaction of **1** when R is a hydrogen atom led to the formation of **3** only.[3d–f] Whereas the **2**/**3** selectivity observed with **1 a**–**f** remained consistent, the stronger erosion observed with **1 g** suggests that electronic factors can play a role.[10]

**Scheme 2:**
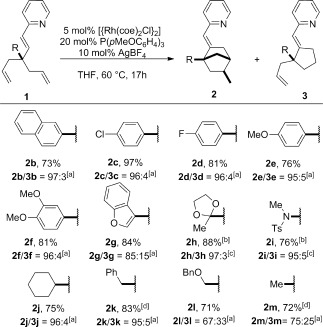
Diastereoselective carbocyclization of 1,6-dienes. Yields are those for the isolated 2 and 3 (combined) unless otherwise noted. [a] 2/3 ratio of isolated material. [b] Yield of isolated 2. [c] 2/3 ratio in crude reaction mixture. [d] 15 mol % of P(*p*MeOC_6_H_4_)_3_ was used. Ts=*p*-tolylsulfonyl.

When we examined the reactivity of the 1,6-octadiene **4 a**, we obtained a complex mixture of products (**5**/**6**/**7**=1:0.7:1.5) in 85 % yield as determined by NMR spectroscopy (Scheme 3). Hence, **5** shows four stereogenic centers and was obtained as a single diastereomer, whereas **6** was formed as an equimolar mixture of *cis* and *trans* olefins. More surprising was the presence of **7**, which was also obtained as a single diastereomer. When a 1:3 mixture of **4 a**/**4 b** was submitted to identical reaction conditions, the same compounds **5**, **6**, and **7** (1:1:3) were obtained in 77 % yield (NMR), with a variation in the *trans*/*cis* ratio of the olefins **6** (3:1). Only sluggish reactions were observed with **4 c** or **4 d**.

**Scheme 3:**
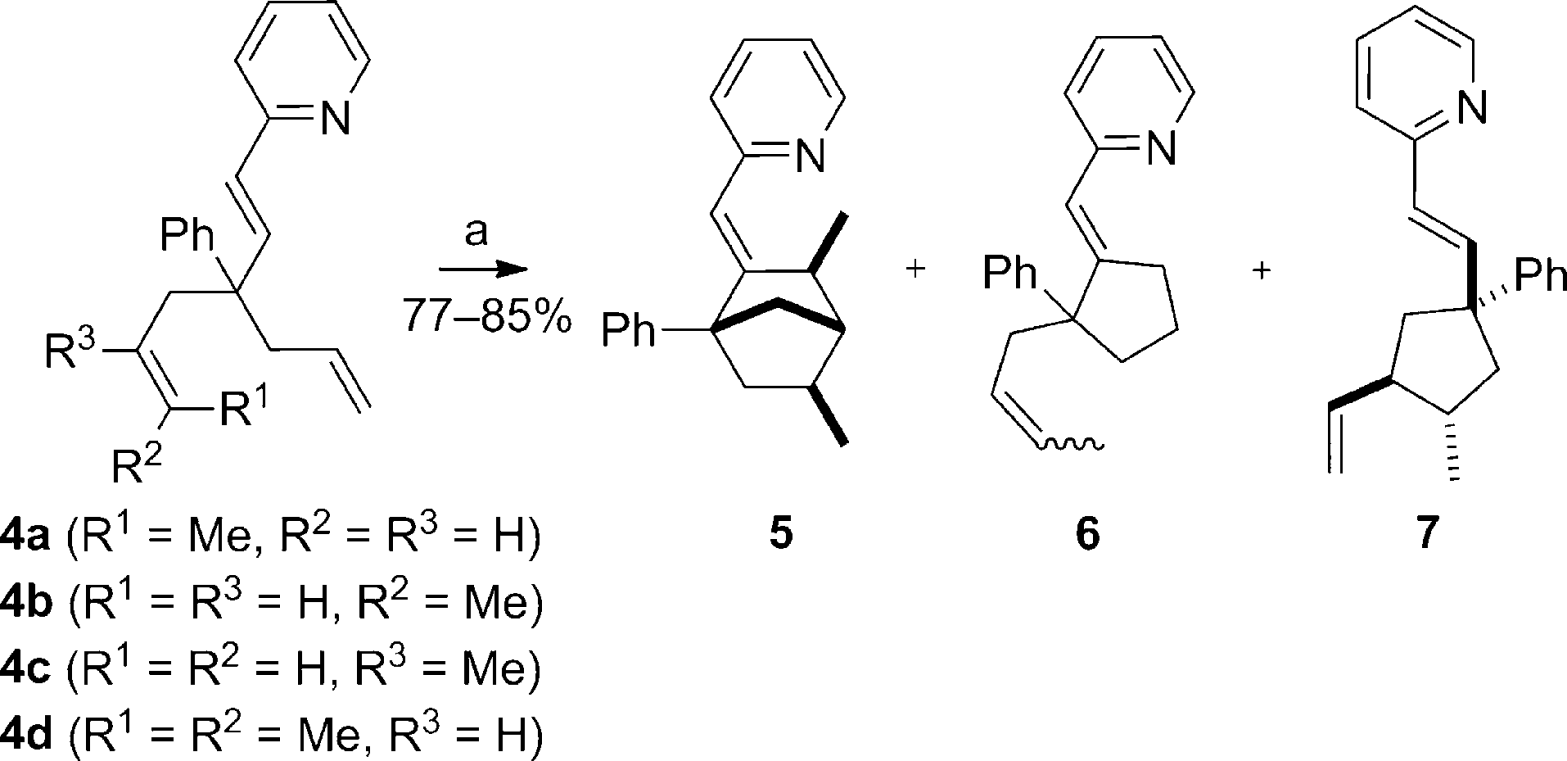
Reaction of substituted the 1,6-dienes 4. a) [{Rh(coe)_2_Cl}_2_] (5 mol %), P(*p*MeOC_6_H_4_)_3_ (20 mol %), AgBF_4_ (10 mol %), THF, 60 °C, 17 h.

Importantly, the vinyl pyridine moiety can easily be converted into other functional groups. Hence, **2 a** was transformed into the ketone **8**, alcohol **9**, α-arylideneketone **10**, and lactone **11** within one or two steps (Scheme 4).

**Scheme 4:**
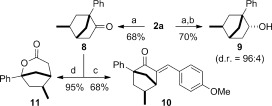
Manipulation of 2 a. a) NaIO_4_ (4 equiv), RuCl_3_ hydrate (20 mol %), 1,2-DCE, MeCN, water. b) DiBAl-H (4 equiv), toluene. c) *t*BuOK (0.1 equiv), *p*-anisaldehyde (1 equiv), DMSO. d) *m*CPBA (2.5 equiv), NaHCO_3_ (5 equiv), CH_2_Cl_2_. DCE=dichloroethane, DMSO=dimethylsulfoxide, *m*CPBA=*m*-chloroperbenzoic acid.

We were eager to gain further understanding of the mechanism of these reactions to explain the exquisite diastereoselectivity and the formation of side products such as **7**. Deuterium-labeling experiments with [D]-**1 a** led to the formation of two monodeuterated isomers ([D]-**2 a**), wherein the deuterium atom was quantitatively and equally distributed between two positions (Scheme 5). Parallel competition experiments with **1 a** and [D]-**1 a** did not indicate any primary kinetic isotope effect (*k*_H_/*k*_D_=1.2). Importantly, the location of the deuterium atom in the [D]-**2 a** isomers did not change with conversion. This observation suggests that the migratory insertion of the first olefin is not only reversible but also occurs much more rapidly than the subsequent steps of the catalytic cycle. In addition, intermolecular transfer of the deuterium atom was not observed in the products for the reaction involving an equimolar amount of [D]-**1 a** and **1 i**, thus confirming the purely intramolecular nature of the hydrometallation step. Hence, [D]-**2 a** and **2 i** were isolated in 95 and 89 % yield, respectively, in this crossover experiment.

**Scheme 5:**
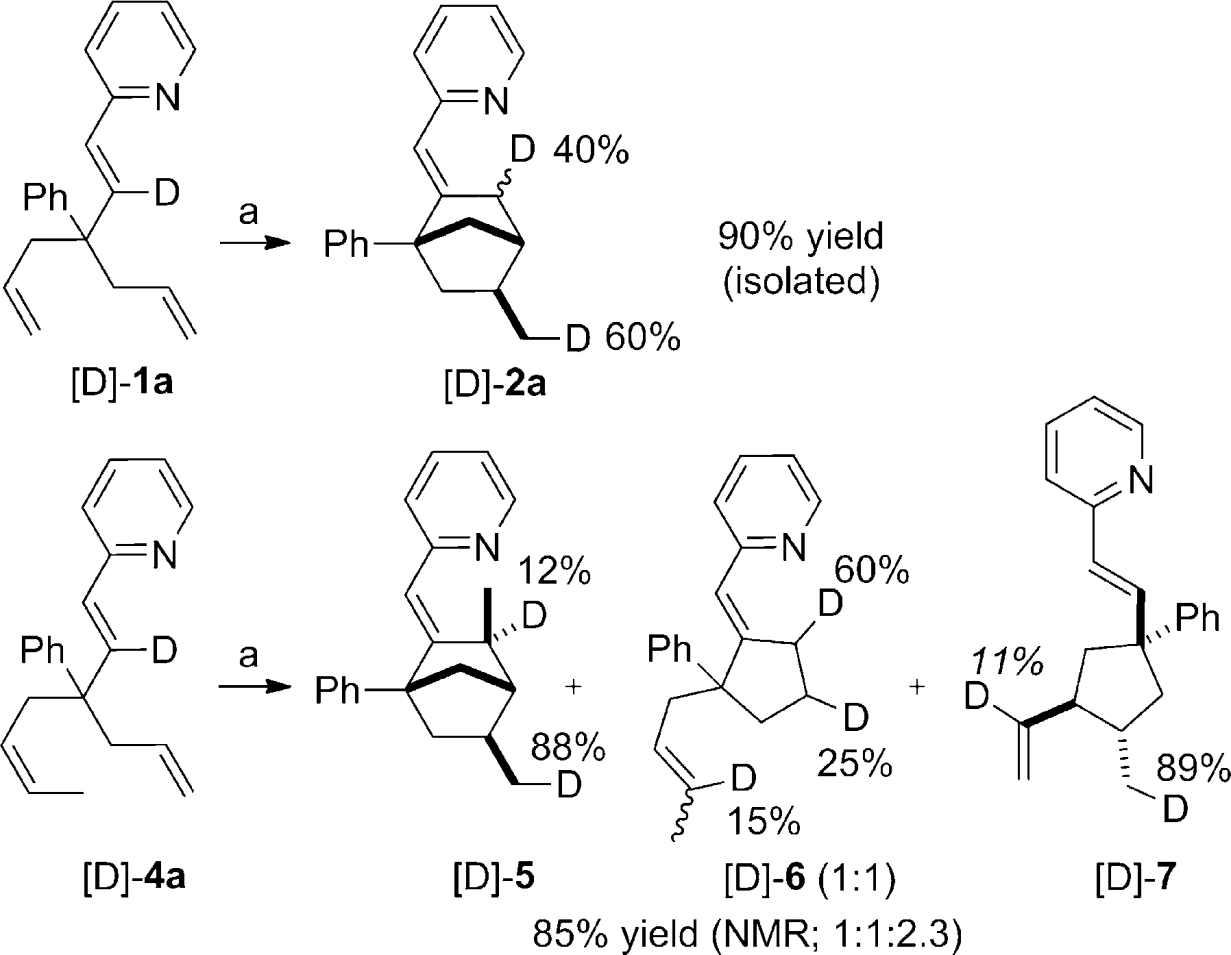
Deuterium-labeling with substrates [D]-1 a and [D]-4 a. a) [{Rh(coe)_2_Cl}_2_] (5 mol %), P(*p*MeOC_6_H_4_)_3_ (20 mol %), AgBF_4_ (10 mol %), THF, 60 °C, 17 h. Percentages of deuterium incorporation are indicated.

Although **7** appears to result from a formal Alder-ene cycloisomerization of **4 a**, the location and extent of incorporation of the deuterium atom in [D]-**7** upon reaction of [D]-**4 a** (Scheme 5) are not consistent with either an oxidative cyclometallation involving the two olefins of the 1,6-diene moiety[6c] or an intermolecular addition of a metal hydride intermediate,[11] and suggests that another mechanism is operative. The other identified components of the mixture were the monodeuterated [D]-**5** and [D]-**6**.

Finally, when examining the kinetic profile of the reaction with the substrate **1 i**, we observed the transient formation of the four-membered ring **12** (Scheme 6). At 86 % conversion, this compound accounts for up to 48 % of the mass balance, before being converted into **2 i**. Hence, we could isolate **12** in 33 % yield. Strikingly, the relative configuration of its stereogenic centers (see arrows) is inverted as compared to those in the final product **2 i**. We observed a similar phenomenon with **1 a** and **1 g**, but the formation of four-membered ring compounds analogous to **12** only peaks at 10 % of the mass balance.

**Scheme 6:**
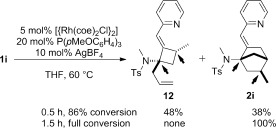
Transient formation of a four-membered ring intermediate.

These results can be accounted for by the following mechanism (Scheme 7). After pyridine-directed C=H bond activation, the migratory insertion of the first olefin into the rhodium–hydrogen bond of **A** is not diastereoselective, and both **B**_*syn*_ and **B**_*anti*_ are formed very rapidly and reversibly (Scheme 7 a). Then, the migratory insertion of the second olefin is not favored in the case of **B**_*anti*_, which undergoes reductive elimination toward **12**, a process particularly favored in the case of R=N(Me)Ts. However, **12** can undergo pyridine-directed C=C bond activation and revert back to **B**_*anti*_. In contrast, **B**_*syn*_ can undergo migratory insertion of the second olefin more favorably than **B**_*anti*_ and give **C**, which after reductive elimination affords **2**. Hence, the exquisite diastereoselectivity of the reaction is dictated by the relative ability of the diastereomers **B** to undergo intramolecular migratory insertion of the terminal olefin. When R is not sterically demanding, or when the second olefin is too substituted, the evolution of **B**_*syn*_ into **C** is more difficult and the formation of **D** becomes competitive, thus leading to the formation of **3**.[3d–f, 10]

**Scheme 7:**
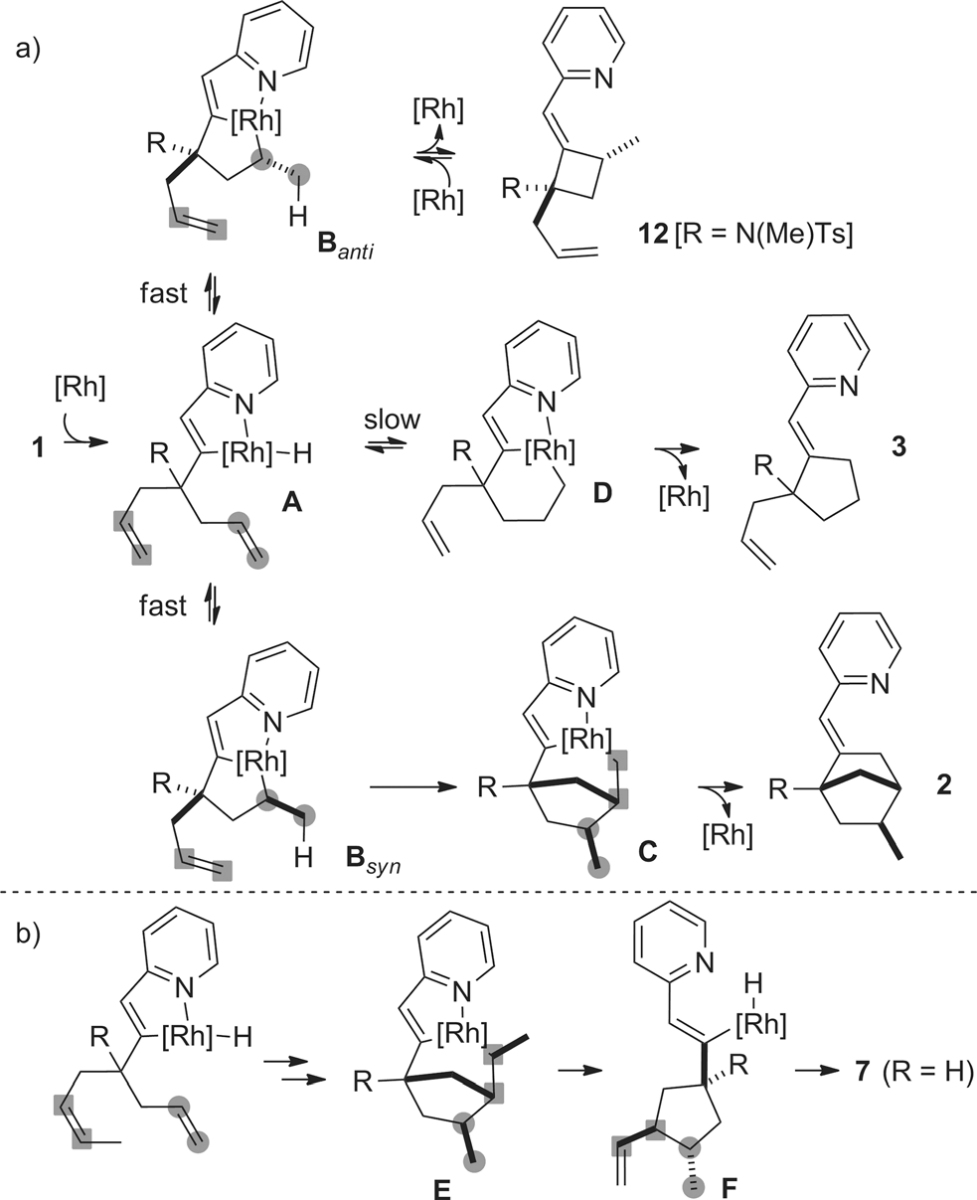
Revised mechanism. [Rh]=[Rh(P(*p*MeOC_6_H_4_)_3_)_*n*_]BF_4_.

The same mechanistic manifold is operative for substrate **4 a**, thus leading to the formation of **5** and **6**. However, reductive elimination from the intermediate **E** toward **5** is in competition with the β-hydride elimination toward **F**, which affords **7** after reductive elimination (Scheme 7 b). The predominant incorporation of the deuterium atom on the methyl group of [D]-**5** indicates that the nonsubstituted olefin undergoes the first migratory insertion ino the rhodium–hydrogen bond more rapidly than the substituted olefin. Nevertheless, this step occurs reversibly, thus explaining the olefin isomerization observed in **6**. The level of deuterium incorporation at the indicated positions in [D]-**6** is in good accordance with previous mechanistic studies on rhodium-catalyzed intramolecular hydrovinylation of olefins.[3f] Incidentally, the predominant incorporation of the deuterium atom on the methyl group of [D]-**7** enables us to propose that the migratory insertion of the second olefin (i.e., **B**_*syn*_ to **C**) occurs as depicted in Scheme 7 and not into the C(sp^2^)=Rh bond of **B**_*syn*_.

In conclusion, we have described the first example of metal-catalyzed functionalization of an olefinic C=H bond using a 1,6-heptadiene as a partner. This rearrangement starkly contrasts with classical reactions of similar prochiral substrates in the presence of late-transition-metal catalysts, as it does not lead to the otherwise typical cyclopentene isomers, but converts the 1,6-dienes into [2.2.1]-cycloheptane derivatives, thereby creating at least three stereogenic centers with complete diastereocontrol. After C=H activation, the carbocyclization proceeds by two consecutive migratory insertions, whereby the first is rapid, reversible, and not diastereoselective, and the second leads to the formation of a single diastereoisomer. Importantly, the vinyl pyridine used to trigger the reaction can be easily transformed into other functional groups which are amenable to further synthetic manipulation.
