# Supplementary material for: Diastereoselective Carbocyclization of 1,6-Heptadienes Triggered by Rhodium-Catalyzed Activation of an Olefinic C=H Bond
Source: Angew Chem Int Ed Engl. 2014 Mar 14;53(16):4209–12. doi: 10.1002/anie.201400080 (PMC4499244; doi:10.1002/anie.201400080)

Angewandte Chemie
Eine Zeitschrift der Gesellschaft Deutscher Chemiker

Supporting Information

© Wiley-VCH 2014

69451 Weinheim, Germany

Diastereoselective Carbocyclization of 1,6-Heptadienes Triggered by Rhodium-Catalyzed Activation of an Olefinic C–H Bond**

Christophe Aïssa, Kelvin Y. T. Ho, Daniel J. Tetlow, and María Pin-Nó*

anie_201400080_sm_miscellaneous_information.pdf

General. Otherwise noted, all reactions were carried out in flame-dried glassware under dry nitrogen atmosphere. The solvents were purified with the solvent purification system Pure Solv MD-6 (THF, Et_2O, CH_2Cl_2, benzene, toluene, hexane) except otherwise noted. Flash chromatography: Merck silica gel 60 (230-400 mesh). NMR: Spectra were recorded on a Bruker DRX 500 and a Bruker DPX 400 spectrometers in $CDCl_3$; chemical shifts (δ) are given in ppm. The solvent signals were used as references and the chemical shifts converted to the TMS scale ($CDCl_3$: δ_C = 77.0 ppm; residual $CHCl_3$ in $CDCl_3$: δ_H = 7.24 ppm). IR: PerkinElmer Spectrum 100 FT-IR spectrometer, wavenumbers ($\tilde{\nu}$) in cm^{-1}. HRMS at the University of Liverpool: micromass LCT mass spectrometer (ES+) and Trio-1000 or Agilent QTOF 7200 mass spectrometers (CI). Melting points: Griffin melting point apparatus (not corrected). Elemental analyses: University of Liverpool. X-Ray crystallography: Bruker D8 Venture Photon 100 Dual Microsource diffractometer. All commercially available compounds were used as received.

Preparation of compounds **1a**–**1m** and **1a-D** S2
Preparation of compounds **4a**–**4d** and **4a-D** S16
Preparation of $[Rh(coe)_2Cl]_2$ S22
Representative procedure for $Rh^{(I)}$-catalysed carbocyclisation of **1a-1m** and **4a** S22
Preparation of compound **8** S31
Preparation of compound **9** S31
Preparation of compound **10** S31
Preparation of compound **11** S32
Preparation of compound **12** S32
NMR spectra for compounds **1a, 1a-D**, **1b**–**1m**, **4a**–**4d**, and **4a-D** S33
NMR spectra for compounds **2a**, **2a-D**, **2b**–**2m**, **3a**, **3l**, **5**, **5-D**, **6**, **6-D**, **7**, **7-D**, **8**–**12** S70
Crystal data and structure refinement for hydrochloride salt of **2a** S166
Crystal data and structure refinement for **2g** S167

Preparation of compounds 1a–1m

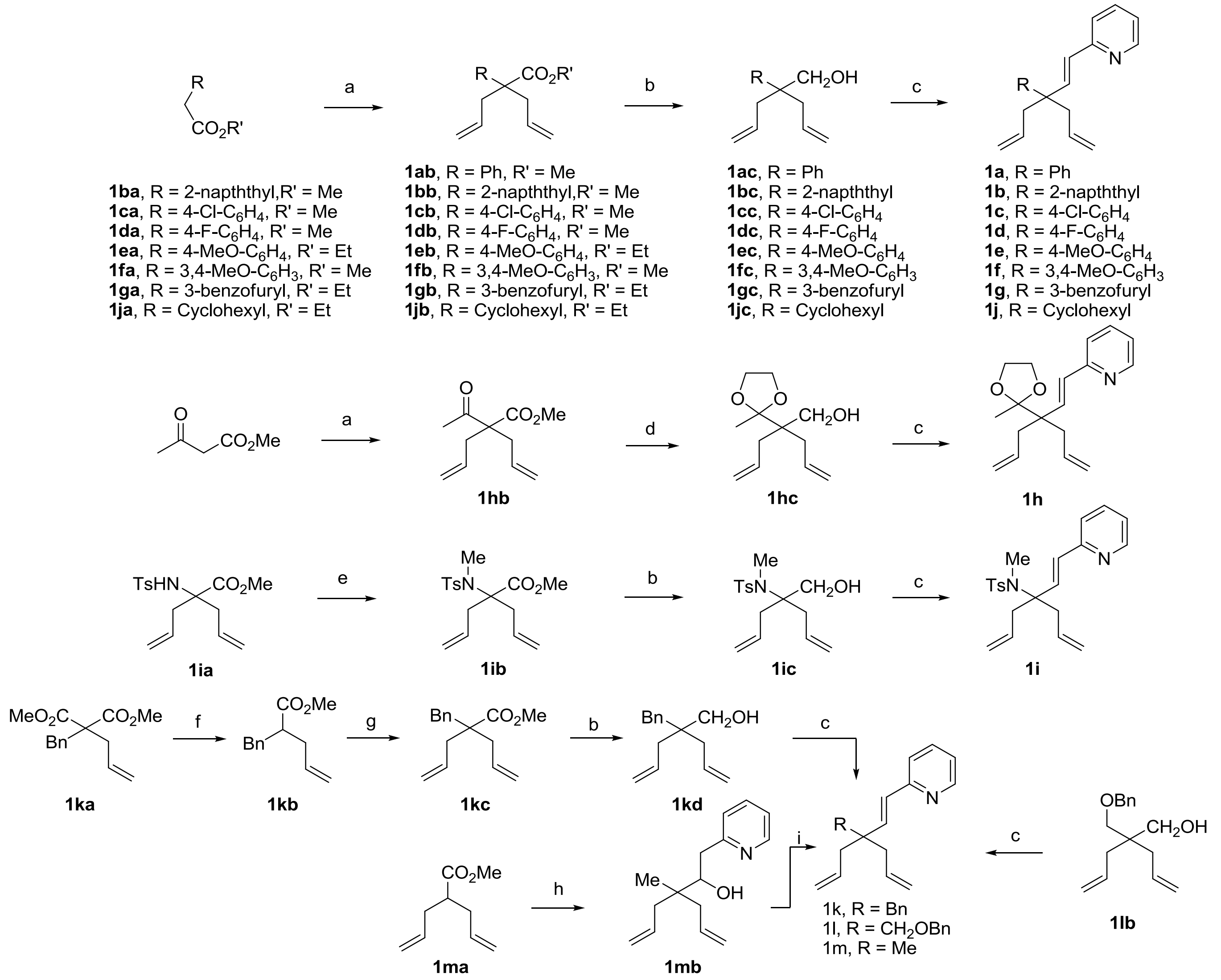

[a] For **1bb**–**1hb**: NaH, allyl bromide, DMF, 81% (**1bb**), 79% (**1cb**), 91% (**1db**), 61% (**1eb**), 76% (**1fb**), 64% (**1gb**), 96% (**1hb**); for **1jb**: i) LDA, THF, -78 °C; ii) allyl bromide, -78 °C to rt, 63%. [b] $LiAlH_4$, Et_2O, 95% (**1ac**), 76% (**1bc**), 80% (**1cc**), 89% (**1dc**), 99% (**1ec**), 97% (**1fc**), 96% (**1gc**), 98% (**1ic**), 75% (**1jc**), 93% (**1kd**). [c] i) $(COCl)_2$, DMSO, Et_3N, CH_2Cl_2; ii) nBuLi, 2-picoline, THF, -78 °C to rt; iv) MsCl, Et_3N, CH_2Cl_2, 0 °C to rt; v) NaHMDS, THF, 0 °C, % (**1a**), 49% (**1b**), 46% (**1c**), 48% (**1d**), 43% (**1e**), 52% (**1f**), 48% (**1g**), 52% (**1h**), 60% (**1i**), 45% (**1j**), 64% (**1k**), 57% (**1l**). [d] i) ethylene glycol, TsOH, $HC(OMe)_3$; ii) $LiAlH_4$, Et_2O, 52%. [e] NaH, MeI, DMF, 92%. [f] LiCl, DMSO, water, 160 °C, 72%. [g] LDA, THF, -78 °C; ii) allyl bromide, -78 °C to rt, quantitative. [h] i) LDA, THF, -78 °C; ii) allyl bromide, -78 °C to rt; iii) $COCl)_2$, DMSO, Et_3N, CH_2Cl_2; iv) nBuLi, 2-picoline, THF, -78 °C to rt, 42%. [i] i) MsCl, Et_3N, CH_2Cl_2, 0 °C to rt; ii) NaHMDS, THF, 0 °C, 68%.

Note: **1ba**–**1ja** are commercially available and **1ab**,[1] **1ka**,[2] and **1lb**[3] are known compounds.

[1] D. Nečas, M. Turský, I. Tišlerová, M. Kotora, *New. J. Chem.* **2006**, *30*, 671
[2] S.-F. Wang, C.-P. Chuang, J.-H. Lee, S.-T. Liu, *Tetrahedron* **1999**, *55*, 2273
[3] K. L. Bray, I. J. S. Fairlamb, J.-P. Kaiser, G. C. Lloyd-Jones, P. A. Slatford, *Topics in Catalysis*, **2002**, *19*, 49

Representative procedure for the bis-allylation leading to 1bb–1hb – NaH (11.2 mmol, 451 mg, 60% dispersion in mineral oil) and allyl bromide (12.3 mmol, 1.1 mL) were added to ester **1ba** (5.13 mmol, 1.11 g) in DMF (25 mL) at 0 °C under N_2. Then the reaction mixture was warmed to room temperature and stirred for 2-16 hours. The reaction mixture was re-cooled to 0 °C, diluted with Et_2O and quenched carefully with H_2O (initially *via* dropwise addition). The organic layer was separated and washed repeatedly with H_2O to remove excess DMF. The organic phase was dried (Na_2SO_4) and the solvent removed under reduced pressure. Purification by flash column chromatography (petroleum ether/EtOAc = 25:1) afforded **1bb** (1.11 g, 81%) as a pale yellow oil.

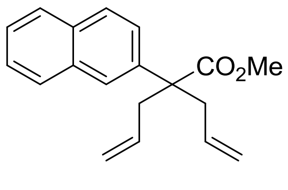

Compound 1bb. ^{1}H NMR (500 MHz, $CDCl_3$): δ = 7.84–7.79 (m, 2H), 7.70–7.68 (m, 1H), 7.51–7.43 (m, 3H), 7.35 (dd, *J* = 8.5, 1.6 Hz, 1H), 5.53 (ddt, *J* = 17.3, 10.0, 7.3 Hz, 2H), 5.09 (dd, *J* = 17.3, 1.1 Hz, 2H), 5.05 (dd, *J* = 10.1, 1.1 Hz, 2H), 3.63 (s, 3H), 2.94–2.85 (m, 4H); ^{13}C NMR (125 MHz, $CDCl_3$): δ = 175.5, 139.1, 133.3 (2C), 133.2, 132.3, 128.1 (2C), 127.5, 126.8, 126.2, 126.0, 125.0, 124.8, 118.7 (2C), 53.7, 52.0, 38.7 (2C); IR (neat): $\tilde{\nu}$ = 3059 (w), 2980 (w), 2949 (w), 1730 (s), 1639 (w), 1600 (w), 1508 (w), 1434 (m), 1340 (w), 1318 (w), 1272 (m), 1212 (s), 1163 (m), 1137 (m), 1040 (w), 1019 (w), 993 (m), 962 (w), 916 (s), 856 (m), 818 (s), 792 (m), 750 (s) cm^{-1}; HRMS ($CI(CH_4)$) calcd for ($C_{19}H_{20}O_2$ + H): 281.1542; found: 281.1539.

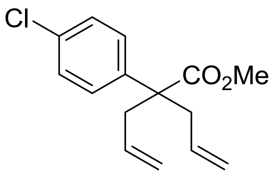

Compound 1cb. Obtained from **1ca** (6.5 mmol, 1.3 g) following the representative procedure. Colourless oil (1.36 g, 79%); ^{1}H NMR (500 MHz, $CDCl_3$): δ = 7.47 (d, *J* = 8.9 Hz, 2H), 7.33 (d, *J* = 8.9 Hz, 2H), 5.67 (ddt, *J* = 17.3, 10.1, 7.3 Hz, 2H), 5.26–5.20 (m, 4H), 3.82 (s, 3H), 2.95 (dd, *J* = 13.6, 8.0 Hz, 2H), 2.91 (dd, *J* = 13.7, 6.8 Hz, 2H); ^{13}C NMR (125 MHz, $CDCl_3$): δ = 175.0, 140.2, 132.9 (2C), 132.8, 128.5 (2C), 127.9 (2C), 119.0 (2C), 53.3, 52.1, 39.0 (2C); IR (neat): $\tilde{\nu}$ = 3078 (w), 2981 (w), 2950 (w), 1731 (s), 1640 (w), 1595 (w), 1492 (m), 1435 (w), 1413 (w), 1402 (w), 1324 (w), 1268 (w), 1213 (s), 1162 (m), 1141 (m), 1093 (m), 1040 (w), 1014 (m), 993 (m), 918 (s), 829 (m), 796 (w), 763 (m), 732 (w) cm^{-1}; HRMS ($CI(CH_4)$) calcd for ($C_{15}H_{17}{}^{35}ClO_2$ + H): 265.0990; found: 265.0993.

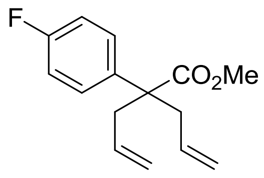

Compound 1db. Obtained from **1da** (11.65 mmol, 1.96 g) following the representative procedure without purification by flash chromatography. Pale yellow gum (2.61 g, 91%);[4] ^{1}H NMR (500 MHz, $CDCl_3$): δ = 7.21 (dd, *J* = 8.8, 5.1 Hz, 2H), 7.03–6.96 (m, 2H), 5.55–5.44 (m, 2H), 5.08–5.02 (m, 4H), 3.64 (s, 3H), 2.79 (dd, *J* = 13.8, 7.8 Hz, 2H), 2.72 (dd, *J* = 13.8 and 6.9 Hz, 2H); ^{13}C NMR (125 MHz, $CDCl_3$): δ = 175.2, 161.6 (d, *J* = 254.6 Hz), 137.4 (d, *J* = 3.8 Hz), 133.0 (2C), 128.0 (d, *J* = 8.0 Hz, 2C), 118.8 (2C), 115.2 (d, *J* = 21.4 Hz, 2C), 53.1, 52.1, 39.0 (2C); IR (neat): $\tilde{\nu}$ = 3078 (w), 2981 (w), 2951 (w), 2924 (w), 2856 (w), 1731 (s), 1640 (w), 1601 (w), 1510 (s), 1443 (w), 1416 (w), 1324 (w), 1297 (w), 1272 (w), 1234 (m), 1211 (s), 1164 (m), 1140 (m), 1106 (w), 1033 (w), 1015 (w), 994 (m), 917 (s), 889 (w), 833 (s), 819 (m), 791 (w), 754 (w) cm^{-1}; MS ($CI(NH_3)$): *m*/*z* (rel. intensity): 249 (100) [M + H]; HRMS ($CI(NH_3)$) calcd for ($C_{15}H_{17}FO_2$ + H): 249.1285; found: 249.1285.

[4] This material contains some monoallylated compound (7 %). The yield indicates the mass recovery. Only signals corresponding to **1db** are described.

Compound 1eb. Obtained from **1ea** (5.5 mmol, 1.21 g) following the representative procedure without purification by flash chromatography. Yellow oil (920 mg, 61%);[5] ^{1}H NMR (500 MHz, $CDCl_3$): δ = 6.79 (s, 1H), 6.78 (d, *J* = 2.0 Hz, 1H), 6.72 (d, *J* = 2.0 Hz, 1H), 5.52–4.45 (m, 2H), 5.08–5.02 (m, 2H), 5.05–5.01 (m, 2H), 3.86 (s, 3H), 3.85 (s, 3H), 3.62 (s, 3H), 2.77 (dd, *J* = 13.7, 7.7 Hz, 2H), 2.71 (d, *J* = 13.7, 6.6 Hz, 2H); ^{13}C NMR (125 MHz, $CDCl_3$): δ = 175.1, 158.2, 133.9, 133.6 (2C), 127.4 (2C), 118.4 (2C), 113.6 (2C), 60.7, 55.2, 52.5, 38.9 (2C), 14.1; IR (neat): $\tilde{\nu}$ = 3075 (w), 2923 (m), 2853 (m), 1728 (s), 1639 (w), 1605 (m), 1589 (m), 1518 (s), 1463 (m), 1442 (m), 1411 (m), 1316 (w), 1260 (s), 1235 (s), 1212 (s), 1150 (s), 1031 (s), 996 (m), 916 (m), 874 (w), 849 (w), 809 (w), 789 (w), 768 (w), 748 (w) cm^{-1}; HRMS (CI(NH_3)) calcd for ($C_{17}H_{22}O_3$ + H): 275.1647; found: 275.1650.

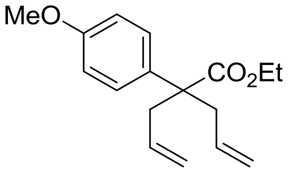

Compound 1fb. Obtained from **1fa** (9.04 mmol, 1.9 g) following the representative procedure without purification by flash chromatography. Pale yellow gum (1.99 g, 76%);[6] ^{1}H NMR (500 MHz, $CDCl_3$): δ = 7.16 (d, *J* = 8.7 Hz, 2H), 6.85 (d, *J* = 8.7 Hz, 2H), 5.51 (ddt, *J* = 17.1, 10.0, 7.2 Hz, 2H), 5.08–5.01 (m, 4H), 4.10 (q, *J* = 7.0 Hz, 2H), 3.79 (s, 3H), 2.76 (dd, *J* = 13.8, 7.7 Hz, 2H), 2.70 (dd, *J* = 13.8, 6.6 Hz, 2H), 0.87 (t, *J* = 6.9 Hz, 3H); ^{13}C NMR (125 MHz, $CDCl_3$): δ = 175.5, 148.7, 147.8, 134.2, 133.4 (2C), 118.6, 118.5 (2C), 110.8, 109.8, 55.9, 55.8, 53.0, 52.0, 38.8 (2C); IR (neat): $\tilde{\nu}$ = 3076 (w), 2979 (w), 2935 (w), 2836 (w), 1725 (s), 1639 (w), 1610 (w), 1581 (w), 1513 (s), 1463 (w), 1442 (m), 1416 (w), 1389 (w), 1366 (w), 1323 (w), 1292 (m), 1272 (m), 1250 (s), 1209 (s), 1183 (s), 1141 (m), 1095 (w), 1035 (s), 996 (w), 916 (s), 856 (m), 831 (m), 809 (w), 791 (w), 750 (w) cm^{-1}; MS (CI(NH_3)): *m*/*z* (rel. intensity): 308 (21) [M + NH_4], 291 (100) [M + H]; HRMS (CI(NH_3)) calcd for ($C_{17}H_{22}O_4$ + H): 291.1591; found: 291.1588.

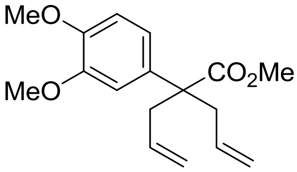

Compound 1gb. Obtained from ethyl-2-(benzo(b)furan-3-yl)acetate (2.45 mmol, 500 mg) following the representative procedure. Colourless oil (442 mg, 64%); ^{1}H NMR (500 MHz, $CDCl_3$): δ = 7.55 (d, *J* = 8.0 Hz, 1H), 7.51 (s, 1H), 7.47 (d, *J* = 8.3 Hz, 1H), 7.28 (dd, *J* = 8.3, 7.2 Hz, 1H), 7.19 (dd, *J* = 8.0,7.2 Hz, 1H), 5.57 (ddt, *J* = 16.9, 10.2, 7.2 Hz, 2H), 5.09–5.03 (m, 4H), 4.13 (q, *J* = 7.1 Hz, 2H), 2.89–2.80 (m, 4H), 1.14 (t, *J* = 7.0 Hz, 3H); ^{13}C NMR (125 MHz, $CDCl_3$): δ = 177.9, 155.5, 142.0, 132.9 (2C), 126.0, 124.3, 122.5, 121.6, 120.7, 118.8 (2C), 111.6, 61.1, 48.6, 37.8 (2C), 14.2; IR (neat): $\tilde{\nu}$ = 2980 (w), 1728 (s), 1640 (w), 1454 (m), 1366 (w), 1261 (w), 1210 (s), 1136 (w), 1110 (m), 1033 (m), 1012 (w), 919 (m), 858 (w), 747 (s) cm^{-1}; MS (CI(NH_3)): *m*/*z* (rel. intensity): 302 (16) [M + NH_4], 285 (100) [M + H]; HRMS (CI(NH_3)) calcd for ($C_{18}H_{20}O_3$ + H): 285.1485; found: 285.1497.

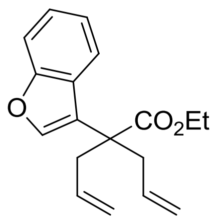

Compound 1hb. Obtained from methyl acetoacetate (9.27 mmol, 1 mL) following the representative procedure. The crude material was used in the next step without further purification. Pale yellow oil (1.72g, 96%); ^{1}H NMR (500 MHz, $CDCl_3$): δ = 5.62 – 5.51 (m, 2H), 5.11 – 5.05 (m, 4H), 3.70 (s, 3H), 2.62 (ddt, *J* = 14.6, 7.4, 1.1 Hz, 2H), 2.56 (ddt, *J* = 14.4, 7.5, 1.1 Hz, 2H), 2.10 (s, 3H); ^{13}C NMR (125 MHz, $CDCl_3$): δ = 203.9, 172.0, 132.1 (2C), 119.2 (2C), 63.4, 52.3, 36.0 (2C), 27.0; IR (neat): $\tilde{\nu}$ = 3080 (w), 2981 (w), 2953 (w), 1744 (m), 1711 (s), 1641 (w), 1435 (m), 1357 (w), 1321 (w), 1279 (m), 1250 (w), 1211 (s), 1179 (m), 1141 (m), 1052 (w), 993 (m), 919 (s), 871 (w), 844 (w), 794 (w), 750 (w), 697 (w) cm^{-1}; MS (CI(CH_4)): 197 (24) [M + H], 165 (28), 137 (97), 123 (100), 95 (21); HRMS (CI (CH_4)) calcd for ($C_{11}H_{16}O_3$ + H): 197.1172 ; found: 197.1170

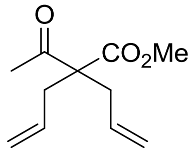

[5] This material contains some monoallylated compound. The yield indicates the mass recovery. Only signals corresponding to **1eb** are described.

[6] This material contains some monoallylated compound. The yield indicates the mass recovery. Only signals corresponding to **1fb** are described.

Compound 1ib. Under N_2, Allyl bromide (17.2 mmol, 1.5 mL) and ^{t}BuOK (17.2 mmol, 1.92 g) were added sequentially to (*E*)-methyl 2-(benzylideneamino)acetate[7] (6.89 mmol, 1.21 g) in THF (35 mL) at 0 °C. The reaction mixture was warmed to room temperature and stirred for 2 hours. The reaction mixture was quenched with H_2O (50 mL) and extracted with Et_2O (2 × 50 mL). The organic phase was dried (Na_2SO_4) and the solvent removed under reduced pressure. The crude residue was re-dissolved in EtOAc (50 mL) and 1M HCl (30 mL) was added. The mixture was stirred at room temperature for 10 minutes. The aqueous layer was washed with EtOAc (30 mL) and the organic layers discarded. The pH of the aqueous layer was adjusted to pH 10–11 with 1M NaOH and re-extracted with CH_2Cl_2 (2 × 50 mL). The combined organic layers were dried (Na_2SO_4) and the solvent removed under reduced pressure to afford 876 mg (75% mass recovery) of a material which was then used without further purification. This material was dissolved in CH_2Cl_2 (26 mL) and *p*-toluenesulfonyl chloride (6.22 mmol, 1.18 g), NEt_3 (6.22 mmol, 0.86 mL) and DMAP (0.57 mmol, 70 mg) were added at 0 °C. The reaction mixture was then stirred at room temperature for 16 hours. The reaction was quenched by addition of 1M HCl (30 mL). The aqueous layer was extracted with CH_2Cl_2 (2 × 30 mL) and the combined organic layers were dried over Na_2SO_4 and concentrated. Purification by flash column chromatography (petroleum ether/EtOAc, 20:1) afforded **1ia** as a solid (824 mg, 49%). NaH (2.85 mmol, 114 mg) was added to **1ia** (2.19 mmol, 710 mg) in DMF (11 mL) at 0 °C under N_2. The reaction mixture was stirred for 30 minutes at 0 °C and MeI (4.38 mmol, 0.27 mL) was added. The reaction mixture was warmed to room temperature and stirred for 2 hours. The reaction was quenched by careful addition of saturated aqueous NH_4Cl (30 mL) and extracted with EtOAc (2 × 50 mL). The combined organic layers were washed with brine, dried (Na_2SO_4) and concentrated under reduced pressure, affording **1ib** as a pale yellow crystalline solid (720 mg, 97%) which was used without further purification. m.p.: 68–70 °C; ^{1}H NMR (500 MHz, $CDCl_3$): δ = 7.81 (d, *J* = 8.3 Hz, 2H), 7.27 (d, *J* = 8.1 Hz, 2H), 5.75–5.65 (m, 2H), 5.17–5.12 (m, 4H), 3.72 (s, 3H), 2.86 (dd, *J* = 14.0, 6.8 Hz, 2H), 2.75 (s, 3H), 2.61 (dd, *J* = 14.0, 8.3 Hz, 2H), 2.41 (s, 3H); ^{13}C NMR (125 MHz, $CDCl_3$): δ = 173.1, 143.4, 137.1, 131.9 (2C), 129.5 (2C), 127.9 (2C), 120.0 (2C), 68.0, 52.2, 39.2 (2C), 32.2, 21.5; IR (neat): $\tilde{\nu}$ = 3083 (w), 3008 (w), 2956 (w), 2924 (w), 1737 (s), 1642 (w), 1597 (w), 1493 (w), 1457 (w), 1431 (m), 1402 (w), 1334 (w), 1312 (s), 1301 (s), 1291 (s), 1264 (w), 1246 (w), 1224 (m), 1208 (m), 1174 (m), 1153 (s), 1113 (s), 1085 (s), 1065 (w), 1038 (w), 1011 (w), 992 (m), 973 (w), 947 (w), 920 (s), 905 (s), 857 (s), 823 (s), 801 (w), 784 (s), 740 (m), 708 (w), 694 (w), 658 (s) cm^{-1}; MS (ESI): *m*/*z* (rel. intensity): 376 (10) [M + K], 360 (100) [M + Na]; HRMS (ESI) calcd for ($C_{17}H_{23}NO_4S$ + Na): 360.1245; found: 360.1232.

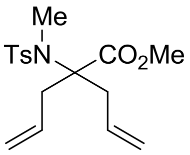

Compound 1jb. nBuLi (2.5 M in hexane, 9.26 mmol, 3.7 mL) was added dropwise to a solution of $^{i}Pr_2NH$ (9.26 mmol, 1.28 mL) in THF (30 mL) at 0˚C before stirring for 20 minutes 0˚C. Then, a solution of ethyl cyclohexylacetate (8.82 mmol, 1.37 mL) in THF (2 mL) was slowly added at -78˚C before stirring for 60 minutes and then adding allyl bromide (9.26 mmol, 0.80 mL). The mixture was then stirred overnight warming to room temperature. The resulting mixture was quenched with a saturated solution of NH_4Cl (20 mL) and it was extracted with EtOAc (2 × 40 mL). The combined organic layers were dried over Na_2SO_4 and concentrated in vacuo. The crude residue was re-dissolved in Et_2O, filtered through a cotton wool plug and the solvent removed under reduced pressure to give the mono allylated ester as pale yellow oil which was used without further purification. Hence, on 300 mg (1.42 mmol) of this material, the same procedure was applied for the second allylation with LDA (2.84 mL) and allyl bromide (2.84 mmol, 0.25

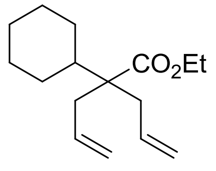

[7] C. M. Griffiths-Jones, D. W. Knight, *Tetrahedron.* **2010**, *66*, 4150

mL) to afford **1jb** as colourless oil (240 mg, 63% over two steps). ^{1}H NMR (500 MHz, $CDCl_3$): δ = 5.77 (ddt, *J* = 17.1, 10.0, 7.5 Hz, 2H), 5.09–5.01 (m, 4H), 4.13 (q, *J* = 6.9 Hz, 2H), 2.47 (dd, *J* = 14.3, 6.6 Hz, 2H), 2.30 (dd, *J* =14.3, 8.0 Hz, 2H), 1.79–1.70 (m, 4H), 1.67–1.59 (m, 1H), 1.54 (tt, *J* = 11.7, 2.5 Hz, 1H), 1.26 (t, *J* = 6.9 Hz, 3H), 1.23–0.99 (m, 5H); ^{13}C NMR (125 MHz, $CDCl_3$): δ = 175.4, 134.9 (2C), 117.6 (2C), 60.0, 51.8, 43.5, 36.9 (2C), 28.0 (2C), 27.0 (2C), 26.5, 14.4; IR (neat): $\tilde{\nu}$ = 3076 (w), 2978 (w), 2926 (s), 2853 (m), 1723 (s), 1638 (w), 1446 (m), 1366 (w), 1335 (w), 1299 (w), 1263 (w), 1207 (s), 1191 (s), 1145 (s), 1096 (w), 1031 (m), 994 (m), 912 (s), 864 (w) cm^{-1}; MS (CI(CH_4)): *m*/*z* (rel. intensity): 251 (100) [M + H], 177 (58), 175 (22), 169 (24), 135 (19), 95 (20); HRMS (CI(CH_4)) calcd for ($C_{16}H_{26}O_2$ + H): 251.2011; found: 251.2005.

Compound 1kb. Lithium chloride (231 mg, 5.45 mmol) was added to a solution of **1ka** (650 mg, 2.48 mmol) in DMSO (17 mL). Water (15 drops) was added and then the mixture was stirred at 160 °C (oil bath temperature) overnight. At room temperature, the mixture was then partitioned between H_2O (10 mL) and Et_2O. The organic layer was washed with H_2O (2 × 20 mL), then brine, and dried over Na_2SO_4, filtered and concentrated. Purification by flash chromatography (petroleum ether/EtOAc : 20/1) gave **1kb** as a colourless oil (348 mg, 72%): ^{1}H NMR (500 MHz, $CDCl_3$): δ = 7.28–7.23 (m, 1H), 7.21–7.16 (m, 1H), 7.15–7.12 (m, 2H), 5.73 (ddt, *J* = 17.1, 10.2, 7.0, 1H), 5.08–5.00 (m, 2H), 3.58 (s, 3H), 2.97–2.88 (m, 1H), 2.80–2.69 (m, 2H), 2.40–2.31 (m, 1H), 2.29–2.21 (m, 1H); ^{13}C NMR (125 MHz, $CDCl_3$): δ = 175.3, 139.1, 135.1, 128.9, 128.4, 126.4, 117.1, 51.4, 47.2, 37.7, 36.0 ; IR (neat): $\tilde{\nu}$ = 3065 (w), 3028 (w), 2950 (w), 1733 (s), 1642 (w), 1604 (w), 1584 (w), 1495 (w), 1455 (w), 1435 (m), 1370 (w), 1262 (w), 1230 (m), 1195 (m), 1161 (s), 1116 (w), 1077 (w), 1031 (w), 994 (w), 915 (m), 832 (w), 762 (w), 743 (m), 698 (s), 656 (w) cm^{-1}; MS (CI(CH_4)): 205 (11) [M + H], 173 (11), 145 (100), 91 (25); HRMS (CI(CH_4)): calcd for ($C_{13}H_{16}O_2$ + H): 205.1229; found: 205.1224.

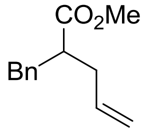

Compound 1kc. Under N_2, a solution of **1kb** (150 mg, 0.73 mmol) in THF (0.6 mL) was added to a solution of LDA (0.95 mmol) at -78°C {LDA prepared from 0.38 mL of a 2.5 M of nBuLi and 0.14 mL of diisopropylamine in THF (1.4 mL) at 0°C}. The resulting solution was stirred at -78°C for 1.5 hour then allyl bromide (89 µL, 1.03 mmol) was slowly added. The mixture was allowed to warm to room temperature overnight before being quenched with few drops of a saturated solution of NH_4Cl. The mixture was diluted with H_2O and extracted three times with diethyl ether. The organic layer was then washed with H_2O and brine and dried over Na_2SO_4, then filtered and concentrated. Purification by flash chromatography (petroleum ether/EtOAc: 25/1) afforded **1kc** as a colourless oil (179 mg, quant.): ^{1}H NMR (500 MHz, $CDCl_3$): δ = 7.26– 7.16 (m, 3H), 7.11–7.06 (m, 2H), 5.78 (ddt, *J* = 16.6, 10.6, 7.3 Hz, 2H), 5.14–5.07 (m, 4H), 3.63 (s, 3H), 2.88 (s, 2H), 2.38 (dd, *J* = 14.3, 7.2 Hz, 2H), 2.27 (dd, *J* = 14.3, 7.4 Hz, 2H); ^{13}C NMR (125 MHz, $CDCl_3$): δ = 175.8, 137.3, 133.6 (2C), 130.0 (2C), 128.1 (2C), 126.5, 118.6 (2C), 51.5, 50.9, 41.4, 38.2 (2C); IR (neat): $\tilde{\nu}$ = 3077(w), 3030 (w), 2980 (w), 2949 (w), 1727 (s), 1639 (w), 1605 (w), 1496 (w), 1435 (m), 1347 (w), 1274 (w), 1200 (m), 1179 (m), 1155 (m), 1079 (w), 1032 (w), 994 (w), 994 (m), 972 (w), 914 (s), 851 (w), 814 (w), 777 (w), 740 (m), 700 (s) cm^{-1}; elemental analysis (%) calcd for $C_{16}H_{20}O_2$: C 78.65, H 8.25; found: C 79.17, H 8.36.

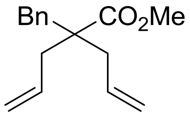

Compound 1ma. Obtained from dimethyl diallyl malonate (1.92 mmol, 0.40 mL) following the procedure described for the preparation of **1kb**. Colourless oil (155 mg, 52 %): ^{1}H NMR (500 MHz, $CDCl_3$): δ = 5.71 (ddt, *J* = 17.1, 10.1, 7.0 Hz, 2H), 5.04 (dt, *J* = 17.1, 1.6 Hz, 2H), 5.03–4.98 (m, 2H), 3.64 (s, 3H), 2.51 (tt, *J* = 8.1, 6.1 Hz, 1H), 2.39–2.30 (m, 2H), 2.28–2.19 (m, 2H); ^{13}C NMR (125 MHz, $CDCl_3$): δ = 135.2 (2C), 117.0 (2C), 51.4, 44.9, 35.8 (2C); IR (neat): $\tilde{\nu}$ = 3080 (w), 2981 (w), 2951 (w), 2846 (w), 1736 (s), 1642 (w), 1437 (m), 1369 (w), 1266 (w), 1236 (m), 1193 (m), 1168 (s), 1139 (m), 994 (m), 914 (s), 862 (w), 833 (w), 763 (w), 702 (w) cm^{-1}; elemental analysis (%) calcd for $C_9H_{14}O_2$: C 70.10, H 9.15; found: C 70.18, H 9.28

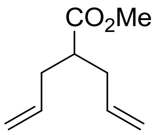

Representative procedure for the reduction of esters into alcohols 1ac–1jc and 1kd – A solution of ester **1ab** (7.38 mmol, 1.7g) in Et_2O (12 mL) was added under N_2 to a suspension of $LiAlH_4$ (4.05 mmol, 150 mg) in Et_2O (24 mL) at 0 °C. After stirring at room temperature for 30 minutes, another portion of $LiAlH_4$ (4.05 mmol, 150 mg) was added. After stirring for 30 minutes, the reaction mixture was quenched carefully at 0 °C with a saturated aqueous solution of Na_2SO_4. After filtration over Celite of the white precipitate thus formed, the solvent was evaporated under reduced pressure and the crude material was purified by flash chromatography (petroleum ether/EtOAc = 10:1) to afford **1ac** as colourless oil (1.42 g, 95%).

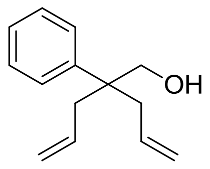

Compound 1ac. ^1H NMR (500 MHz, $CDCl_3$): δ = 7.36–7.31 (m, 4H), 7.24–7.19 (m, 1H), 5.62 (ddt, *J* = 17.1, 10.1, 7.2 Hz, 2H), 5.11–4.99 (m, 4H), 3.78 (d, *J* = 6.6 Hz, 2H), 2.53 (dd, *J* = 13.9, 7.2 Hz, 2H), 2.45 (dd, *J* = 14.1, 7.2 Hz, 2H), 1.26 (t, *J* = 6.6 Hz, 1H(OH)); ^{13}C NMR (125 MHz, $CDCl_3$): δ = 143.4, 134.3 (2C), 128.4 (2C), 126.8 (2C), 126.2, 117.8 (2C), 67.8, 45.8, 39.5 (2C); IR (neat): $\tilde{\nu}$ = 3410 (br), 3074 (w), 3006 (w), 2978 (w), 2925 (w), 1638 (w), 1600 (w), 1581 (w), 1498 (m), 1445 (m), 1415 (w), 1385 (w), 1326 (w), 1294 (w), 1218 (w), 1143 (w), 1046 (m), 997 (m), 911 (s), 859 (w), 768 (m), 744 (w), 698 (s), 672 (m) cm^{-1}; elemental analysis (%) calcd for $C_{14}H_{18}O$: C 83.12, H 8.97; found: C 83.25, H 9.04.

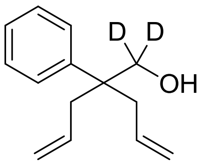

Compound 1ac-D. Obtained from **1ab** (1.23 mmol, 283 mg) following the representative procedure but using $LiAlD_4$. Colourless oil (237 mg, 94%); ^1H NMR (500 MHz, $CDCl_3$): δ = 7.38–7.29 (m, 4H), 7.24 – 7.18 (m, 1H), 5.62 (ddt, *J* = 17.2, 10.0, 7.3 Hz, 2H), 5.11–5.04 (m, 2H), 5.04–4.99 (m, 2H), 2.53 (dd, *J* = 14.1, 7.2 Hz, 2H), 2.45 (dd, *J* = 13.9, 7.4 Hz, 2H), 1.25 (s, 1H(OH)); ^{13}C NMR (125 MHz, $CDCl_3$): δ = 143.3, 134.3 (2C), 128.4 (2C), 126.8 (2C), 126.2, 117.8 (2C), 67.0 (pent, *J* = 21.8 Hz), 45.6, 39.5 (2C); IR (neat): $\tilde{\nu}$ = 3399 (br), 3074 (w), 3006 (w), 2978 (w), 2923 (w), 2207 (w), 2101 (w), 1638 (w), 1600 (w), 1580 (w), 1497 (w), 1445 (m), 1415 (w), 1292 (w), 1193 (w), 1158 (w), 1102 (m), 1030 (w), 998 (m), 975 (m), 910 (s), 838 (w), 807 (w), 759 (m), 733 (w), 697 (s), 665 (w) cm^{-1}; MS (CI(NH_3)): *m*/*z* (rel. intensity): 222 (100) [M + NH_4]; HRMS (CI(NH_3)) calcd for ($C_{14}H_{16}D_2O$ + NH_4): 222.1821; found: 222.1824.

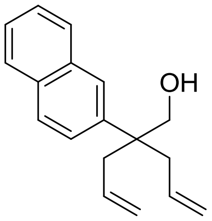

Compound 1bc. Obtained from **1bb** (1.69 mmol, 475 mg) following the representative procedure. Colourless oil (322 mg, 76%); ^1H NMR (400 MHz, $CDCl_3$): δ = 7.91–7.85 (m, 3H), 7.80 (d, *J* = 1.5 Hz, 1H), 7.57 (dd, *J* = 8.5, 1.7 Hz, 1H), 7.55–7.49 (m, 2H), 5.70 (ddt, *J* = 17.1, 10.0, 7.5 Hz, 2H), 5.17 (dd, *J* = 17.1, 1.7 Hz, 2H), 5.08 (dd, *J* = 10.0, 1.7 Hz, 2H), 3.92 (s, 2H), 2.69 (dd, *J* = 13.9, 7.4 Hz, 2H), 2.62 (dd, *J* = 13.9, 7.4 Hz, 2H); ^{13}C NMR (100 MHz, $CDCl_3$): δ = 140.8, 134.2 (2C), 133.2, 131.9, 128.0, 127.9, 127.3, 126.0, 125.9, 125.7, 124.8, 117.9 (2C), 67.5, 45.9, 39.3 (2C); IR (neat): $\tilde{\nu}$ = 3409 (br), 3058, 3005, 2976, 2923, 1637, 1598, 1506, 1443, 1414, 1506, 1384, 1325, 1275, 1203, 1132, 1050, 1018, 997, 914, 855, 816, 748, 690 cm^{-1}; MS (CI(CH_4)): *m*/*z* (rel. intensity): 253 (100) [M + H]; HRMS (CI(CH_4)) calcd for ($C_{18}H_{20}O$ + H): 253.1592; found: 253.1583.

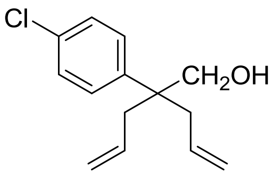

Compound 1cc. Obtained from **1cb** (1.76 mmol, 465 mg) following the representative procedure. Colourless oil (330 mg, 80%); ^1H NMR (400 MHz, $CDCl_3$): δ = 7.32 (d, *J* = 8.6 Hz, 2H), 7.28 (d, *J* = 8.6 Hz, 2H), 5.60 (ddt, *J* = 17.2, 10.1, 7.2 Hz, 2H), 5.08 (d, *J* = 17.2 Hz, 2H), 5.04 (dd, *J* = 10.1, 1.0 Hz, 2H), 3.77 (s, 2H), 2.51 (dd, 13.9, 7.2 Hz, 2H), 2.43 (dd, *J* = 14.0, 7.2 Hz, 2H); ^{13}C NMR (100 MHz, $CDCl_3$): δ = 142.1, 133.9 (2C), 132.1, 128.5 (2C), 128.3 (2C), 118.2 (2C), 67.6, 45.6, 39.7 (2C); IR (neat): $\tilde{\nu}$ = 3410 (br), 3076 (w), 2978 (w), 2924 (w), 1639 (m), 1495 (s), 1444 (m), 1416 (w), 1399 (w), 1329 (w), 1096 (s), 1051 (s), 1012 (s), 997 (s), 912 (s), 824 (s), 761 (w), 729 (s), 683 (w), 682 (w) cm^{-1}; MS (CI(NH_3)): *m*/*z* (rel. intensity): 256 (13) [M(^{37}Cl) + NH_4], 254 (56) [M(^{35}Cl) + NH_4], 144 (26), 142 (100); HRMS (CI(NH_3)) calcd for ($C_{14}H_{17}{}^{37}ClO$ + NH_4): 254.1312; found: 254.1313.

Compound 1dc. Obtained from **1db** (1.64 mmol, 406 mg) following the representative procedure.

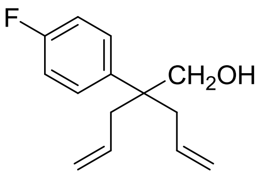

Colourless oil (348 mg, 89%); ^{1}H NMR (400 MHz, $CDCl_3$): δ = 7.31 (dd, *J* = 8.9, 5.2 Hz, 2H), 7.03 (t, *J* = 8.8 Hz, 2H), 5.61 (ddt, *J* = 17.1, 10.0, 7.2 Hz, 2H), 5.08 (dt, *J* = 17.1, 1.4 Hz, 2H), 5.04 (dt, *J* = 10.2, 1.1 Hz, 2H), 3.78 (d, *J* = 5.6 Hz, 2H), 2.51 (dd, *J* = 14.0, 7.2 Hz, 2H), 2.44 (dd, *J* = 14.0, 7.3 Hz, 2H), 1.34 (t, *J* = 6.0 Hz, 1H(OH)); ^{13}C NMR (100 MHz, $CDCl_3$): δ = 161.3 (d, *J* = 243.8 Hz), 139.1 (d, *J* = 3.5 Hz), 134.1 (2C), 128.4 (d, *J* = 7.9 Hz, 2C), 118.1 (2C), 115.1 (d, *J* = 20.8 Hz, 2C), 67.8, 45.5, 39.9 (2C); IR (neat): $\tilde{\nu}$ = 3388 (br), 3076 (w), 2924 (m), 2855 (w), 1639 (m), 1604 (m), 1511 (s), 1445 (m), 1416 (w), 1378 (w), 1331 (w), 1234 (s), 1165 (s), 1051 (s), 1015 (s), 997 (s), 915 (s), 831 (s), 816 (s), 729 (m), 671 (w) cm^{-1}; MS (CI(NH_3)): *m*/*z* (rel. intensity): 238 (20) [M + NH_4], 203 (20), 189 (44), 126 (100); HRMS (CI(NH_3)) calcd for ($C_{14}H_{17}FO$ + NH_4): 238.1602; found: 238.1605.

Compound 1ec. Obtained from **1eb** (1.78 mmol, 490 mg) following the representative procedure.

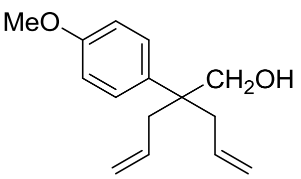

Colourless oil (409 mg, 99%); ^{1}H NMR (500 MHz, $CDCl_3$): δ = 7.26 (d, *J* = 8.8 Hz, 2H), 6.89 (d, *J* = 8.8 Hz, 2H), 5.71–5.59 (m, 2H), 5.09 (d, *J* = 16.9 Hz, 2H), 5.03 (d, *J* = 10.1 Hz, 2H), 3.80 (s, 3H), 3.75 (s, 2H), 2.51 (dd, *J* = 13.9, 7.2 Hz, 2H), 2.43 (dd, *J* = 13.9, 7.2 Hz, 2H); ^{13}C NMR (125 MHz, $CDCl_3$): δ = 157.8, 135.2, 134.4 (2C), 127.8 (2C), 117.7 (2C), 113.7 (2C), 67.9, 55.1, 45.2, 39.5 (2C); IR (neat): $\tilde{\nu}$ = 3437 (br), 3074 (w), 3004 (w), 2977 (w), 2933 (w), 2836 (w), 1638 (m), 1610 (m), 1580 (w), 1514 (s), 1464 (m), 1441 (m), 1415 (w), 1295 (m), 1247 (s), 1186 (s), 1148 (w), 1116 (w), 1034 (s), 998 (m), 912 (s), 827 (s), 801 (m), 737 (w), 716 (w), 672 (w) cm^{-1}; MS (CI(CH_4)): *m*/*z* (rel. intensity): 233 (30) [M + H], 121 (100); HRMS (CI) calcd for ($C_{15}H_{19}O_2$ + H): 233.1541; found: 233.1537.

Compound 1fc. Obtained from **1fb** (2.04 mmol, 593 mg) following the representative procedure.

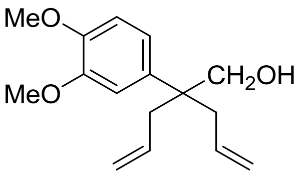

Colourless oil (519 mg, 97%); ^{1}H NMR (500 MHz, $CDCl_3$): δ = 6.89–6.83 (m, 3H), 5.65 (ddt, *J* = 17.1, 10.1, 7.1 Hz, 2H), 5.13–5.06 (m, 2H), 5.06–5.00 (m, 2H), 3.88 (s, 3H), 3.87 (s, 3H), 3.74 (s, 2H), 2.51 (dd, *J* = 13.8, 7.2 Hz, 2H), 2.44 (dd, *J* = 13.8, 7.2 Hz, 2H) and 1.37–1.27 (m, 1H(OH)); ^{13}C NMR (125 MHz, $CDCl_3$): δ = 148.8, 147.4, 135.9, 134.4 (2C), 119.2, 117.8 (2C), 110.9, 110.4, 68.1, 55.9, 55.8, 45.5, 39.5 (2C); IR (neat): $\tilde{\nu}$ = 3491 (br), 3074 (w), 3004 (w), 2933 (m), 2836 (w), 1638 (w), 1605 (w), 1588 (w), 1517 (s), 1464 (s), 1442 (s), 1411 (m), 1332 (w), 1252 (s), 1236 (s), 1185 (w), 1149 (s), 1026 (s), 998 (s), 912 (s), 827 (w), 805 (m), 768 (s), 733 (w), 688 (w) cm^{-1}; MS (CI(NH_3)): *m*/*z* (rel. intensity): 280 (32) [M + NH_4], 263 (29) [M + H], 245 (100), 221 (35), 151 (44); HRMS (CI) calcd for ($C_{16}H_{22}O_3$ + NH_4): 280.1907; found: 280.1898.

Compound 1gc. Obtained from **1gb** (1.05 mmol, 300 mg,) following the representative procedure.

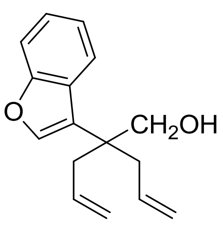

Colourless oil (245 mg, 96%); ^{1}H NMR (500 MHz, $CDCl_3$): δ = 7.73 (d, *J* = 7.9 Hz, 1H), 7.50 (d, *J* = 8.1 Hz, 1H), 7.43 (s, 1H), 7.30 (dd, *J* = 8.1, 7.3 Hz, 1H), 7.23 (dd, *J* = 7.9, 7.3 Hz, 1H), 5.68 (ddt, *J* = 17.2, 10.1, 7.3 Hz, 2H), 5.10 (d, *J* = 17.2 Hz, 2H), 5.04 (dd, *J* = 10.1, 1.0 Hz, 2H), 3.86 (d, *J* = 4.0 Hz, 2H), 2.65–2.56 (m, 4H), 1.46–1.41 (m, 1H(OH)); ^{13}C NMR (125 MHz, $CDCl_3$): δ = 155.8, 142.8, 133.8 (2C), 126.1, 124.2, 122.6, 122.4, 121.3, 118.2 (2C), 111.9, 66.3, 43.3, 38.0 (2C); IR (neat): $\tilde{\nu}$ = 3410 (br), 3074 (w), 3006, 2977 (w), 2925 (w), 1639 (w), 1453 (s), 1416 (w), 1386 (w), 1333 (w), 1284 (w), 1256 (w), 1197 (w), 1133 (w), 1111 (m), 1051 (s), 1034 (m), 1016 (m), 996 (s), 914 (s), 858 (s), 805 (w), 766 (w), 743 (s), 688 (w) cm^{-1}; MS (CI(NH_3)): *m*/*z* (rel. intensity): 260 (5) [M + NH_4], 243 (57) [M + H], 225 (100), 201 (24), 131 (22); HRMS (CI) calcd for ($C_{16}H_{18}O_2$ + H): 243.1385; found: 243.1377; calcd for ($C_{16}H_{18}O_2$ + NH_4): ; found: 260.1642.

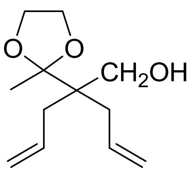

Compound 1hc. Under N_2, $TsOH.H_2O$ (49 mg, 0.25 mmol) was added to a solution of **1hb** (508 mg, 2.59 mmol), diethylene glycol (0.567 mmol, 10.19 mmol) and trimethyl orthoformate (0.56 mL, 5.10 mmol) at room temperature. After stirring overnight, the mixture was quenched with a saturated solution of $NaHCO_3$ and extracted with Et_2O (3 × 5 mL). The organic layer was washed with H_2O, then brine. It was then dried over Na_2SO_4, filtered and concentrated to give 622 mg of an oil which was dissolved in Et_2O (3 mL) under N_2 and added by cannula to a suspension of $LiAlH_4$ (53.0 mg, 1.40 mmol) in Et_2O (10 mL) at 0 °C. After 10 mins at 0 °C, another portion of $LiAlH_4$ (53.0 mg, 1.40 mmol) was added. After 2hours stirring at room temperature, the reaction mixture was quenched carefully at 0 °C with a saturated aqueous solution of Na_2SO_4. After filtration over Celite of the white precipitate thus formed, the solvent was evaporated under reduced pressure and the crude material was purified by flash chromatography (petroleum ether/EtOAc: 4/1) to afford **1hc** as a colourless oil (284 mg, 52 % over 2 steps). ^1H NMR (500 MHz, $CDCl_3$): δ = 5.92 (ddt, *J* = 17.1, 10.0, 7.3 Hz, 2H), 5.05 (d, *J* = 17.4 Hz, 2H), 5.04 (d, *J* = 10.0 Hz, 2H), 3.99–3.89 (m, 4H), 3.51 (d, *J* = 5.9 Hz, 2H), 3.08 (t, *J* = 5.9 Hz, 1H(OH)), 2.22 (dd, *J* = 14.4, 7.0 Hz, 2H), 2.15 (dd, *J* = 14.3, 7.6 Hz, 2H), 1.31 (s, 3H); ^{13}C NMR (125 MHz, $CDCl_3$): δ = 135.0 (2C), 117.3 (2C), 114.7, 66.0, 64.4 (2C), 47.9, 34.9 (2C), 20.0; IR (neat): $\tilde{\nu}$ = 3526 (br), 3074 (w), 2980 (w), 2941 (w), 2888 (m), 1638 (w), 1471 (w), 1436 (w), 1413 (w), 1377 (w), 1334 (w), 1204 (m), 1095 (m), 1038 (s), 998 (m), 950 (m), 912 (s), 874 (m), 765 (w), 693 (w) cm^{-1}; HRMS (CI(NH_3)): calcd for ($C_{12}H_{20}O_3$ + H): 213.1485; found: 213.1490

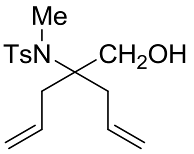

Compound 1ic. Obtained from **1ib** (1.48 mmol, 500 mg,) following the representative procedure. Colourless oil (440 mg, 98%); ^1H NMR (500 MHz, $CDCl_3$): δ = 7.72 (d, *J* = 8.4 Hz, 2H), 7.26 (d, *J* = 8.4 Hz, 2H), 5.73 (ddt, *J* = 16.7, 10.2, 7.5 Hz, 2H), 5.12–5.04 (m, 4H), 3.82 (d, *J* = 7.1 Hz, 2H), 2.89 (s, 3H), 2.74 (t, *J* = 7.1 Hz, 1H(OH)), 2.51 (dd, *J* = 14.3, 7.1 Hz, 2H), 2.41 (dd, *J* = 14.3, 7.4 Hz, 2H), 2.39 (s, 3H); ^{13}C NMR (125 MHz, $CDCl_3$): δ = 143.2, 138.7, 133.0 (2C), 129.5 (2C), 127.1 (2C), 118.8 (2C), 68.3, 65.7, 38.8 (2C), 33.1, 29.6, 21.4; IR (neat): $\tilde{\nu}$ = 3529 (br), 3075 (w), 2979 (w), 2925 (w), 1638 (w), 1598 (w), 1494 (w), 1446 (w), 1315 (m), 1304 (m), 1213 (w), 1151 (s), 1087 (m), 1061 (m), 1017 (w), 997 (m), 914 (s), 813 (s), 713 (m), 680 (w) cm^{-1}; MS (CI(CH_4)): *m*/*z* (rel. intensity): 209 (2) [M + H], 207 (4), 191 (14), 189 (18); MS (ESI): *m*/*z* (rel. intensity): 348 (15) [M + K], 332 (100) [M + Na]; HRMS (ESI) calcd for ($C_{16}H_{23}NO_3S$ + Na): 332.1296; found: 332.1287.

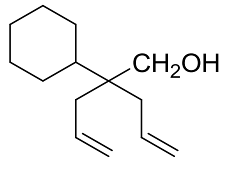

Compound 1jc. Obtained from **1jb** (0.868 mmol, 217 mg) following the representative procedure. Colourless oil (132 mg, 75%); ^1H NMR (500 MHz, $CDCl_3$): δ = 5.96 (ddt, *J* = 17.0, 10.1, 7.5 Hz, 2H), 5.11 (dd, *J* = 17.1, 1.5 Hz, 2H), 5.08 (dd, *J* = 10.1, 1.3 Hz, 2H), 3.56 (d, *J* = 5.3 Hz, 2H), 2.20–2.11 (m, 4H), 1.84–1.12 (m, 4H), 1.72–1.64 (m, 1H), 1.50–1.37 (m, 2H), 1.30–1.05 (m, 5H); ^{13}C NMR (125 MHz, $CDCl_3$): δ = 135.9 (2C), 117.1 (2C), 67.8, 43.1, 41.9, 38.0 (2C), 27.3 (2C), 27.2 (2C), 26.7; IR (neat): $\tilde{\nu}$ = 3393 (br), 3074 (w), 2924 (s), 2852 (m), 1637 (w), 1447 (w), 1044 (w), 995 (w), 910 (m) cm^{-1}; MS (CI(CH_4)): *m*/*z* (rel. intensity): 209 (2) [M + H], 207 (4), 191 (14), 189 (18); HRMS (CI(CH_4)) calcd for ($C_{14}H_{24}O$ + H): 209.1905; found: 209.1895.

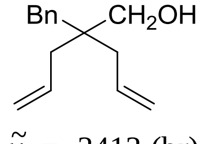

Compound 1kd. Obtained from **1kc** (1.23 mmol, 300 mg) following the representative procedure. Colourless oil (246 mg, 93%): ^1H NMR (500 MHz, $CDCl_3$): δ = 7.29–7.23 (m, 2H), 7.23–7.17 (m, 3H), 5.98–5.87 (m, 2H), 5.15–5.06 (m, 4H), 3.36 (d, *J* = 6.0 Hz, 2H), 2.63 (s, 2H), 2.04 (d, *J* = 7.4 Hz, 4H), 1.33, (t, *J* = 6.1 Hz, 1H(OH)); ^{13}C NMR (125 MHz, $CDCl_3$): δ = 138.1, 134.6, 130.6, 128.0, 126.1, 118.0, 66.8, 42.1, 40.4, 38.6; IR (neat): $\tilde{\nu}$ = 3413 (br), 3075 (w), 3029 (w), 2005 (w), 2977 (w), 2923 (m), 1638 (m), 1604 (w), 1496 (w), 1441 (m), 1415 (w), 1328 (w), 1230 (w), 1156 (w), 1048 (m), 1031 (m), 1016 (m), 995 (m), 911 (s), 861 (w), 812 (w), 783 (w), 740 (m), 702 (s), 665 (w) cm^{-1}; elemental analysis (%) calcd for $C_{15}H_{20}O$: C 83.28, H 9.32; found: C 83.37, H 9.35.

Representative procedure for the conversion of alcohols into compounds 1a–1l – Under N_2, DMSO (87 µL, 1.20 mmol) in CH_2Cl_2 (0.8 mL) was added to a solution of oxalyl chloride (53 µL, 0.61 mmol) in CH_2Cl_2 (3.9 mL) at –78 °C. After 10 minutes stirring at –78 °C, a solution of the **1ac** (95 mg, 0.47 mmol) in CH_2Cl_2 (0.8 mL) was added. After 20 minutes stirring at –78 °C, triethylamine (0.32 mL, 2.34 mmol) was added rapidly and the mixture was stirred at room temperature during 20 minutes. A saturated solution of NH_4Cl was added to the reaction mixture which was then extracted three times with diethyl ether. The organic layer was washed with H_2O and brine, dried over Na_2SO_4, filtered, and concentrated to give 93 mg of aldehyde which was used without further purification in the next step. Under N_2, nBuLi (0.32mL, 0.55 mmol, 1.7 M in hexanes) was added to a –78 °C solution of 2-Methylpyridine (59 µL, 0.60 mmol) in THF (2.5 mL). After 10 minutes of stirring at –78 °C, a solution of the aldehyde previously obtained (93 mg, 0.46 mmol) in THF (0.8 mL) was added by canula. After stirring for 10 minutes at –78 °C, the reaction was quenched with enough MeOH to turn the deep red solution to yellow and then allowed to warm to room temperature. The crude mixture was then partitioned between a saturated aqueous solution of $NaHCO_3$, water and Et_2O. After three extractions with Et_2O (5 mL), the combined organic layers were dried over Na_2SO_4, filtered and concentrated. Purification by flash column chromatography (petroleum ether/EtOAc: 7/1 → 6/1) afforded 133 mg of a clear oil which was dissolved in CH_2Cl_2 (0.4 mL) under N_2 and cooled to –40 °C. Et_3N (193 µL, 1.36 mmol) and methanesulfonyl chloride (53 µL, 0.68 mmol) were added dropwise. After stirring overnight at room temperature, the precipitate thus formed was then filtered off over Celite, washing with EtOAc, and the filtrate was concentrated under reduce pressure. Under N_2, the residue was dissolved in THF (2.4 mL) under N_2. At –20 °C, NaHMDS (0.6 mL, 0.61 mmol, 1M in THF) was added. After stirring at room temperature for 1 hour, the reaction mixture was quenched with a saturated solution of $NaHCO_3$ and partitioned between water and EtOAc. After three extractions with Et_2O (5 mL), the combined organic layers were dried over Na_2SO_4, filtered and concentrated. Purification by flash column chromatography (petroleum ether/Et_2O: 9/1 →8/1) afforded **1a** as a white solid (56 mg, 55% over four steps)

Compound 1a. mp.: 27–28 °C; ^{1}H NMR (500 MHz, $CDCl_3$): δ = 8.53 (d, J = 4.6 Hz, 1H), 7.62–7.56 (m, 1H), 7.35–7.26 (m, 5H), 7.22–7.16 (m, 1H), 7.12–7.07 (m, 1H), 6.85 (d, J = 16.4 Hz, 1H), 6.53 (d, J = 16.2 Hz, 1H), 5.61 (ddt, J = 17.2, 10.0, 7.1 Hz, 2H), 5.08–4.97 (m, 4H), 2.68 (d, J = 7.1 Hz, 4H); ^{13}C NMR (125 MHz, $CDCl_3$): δ = 156.0, 149.5, 144.8, 141.8, 136.4, 134.4 (2C), 128.7, 128.1, 127.4, 126.2, 121.8, 121.0, 117.8 (2C), 46.8, 41.9 (2C); IR (neat): $\tilde{\nu}$ = 3059 (w), 3034 (w), 3007 (w), 2922 (w), 2902 (w), 2841 (w), 1953 (w), 1892 (w), 1836 (w), 1650 (m), 1640 (m), 1598 (w), 1585 (m), 1563 (m), 1493 (m), 1469 (m), 1444 (m), 1429 (m), 1322 (w), 1302 (w), 1262 (w). 1243 (w), 1189 (w), 1150 (w), 1127 (w), 1097 (w), 1089 (w), 1049 (w), 1030 (w), 1009 (w), 990 (m), 979 (m), 943 (m), 935 (m), 915 (s), 889 (m), 856 (m), 779 (m), 766 (m), 749 (s), 701 (s), 671 (w), 654 (w) cm^{-1}; HRMS (ESI): calcd for ($C_{16}H_{27}NO_3$ + Na): 276.1752; found: 276.1750; elemental analysis (%) calcd for $C_{20}H_{21}N$: C 87.23, H 7.69, N 5.09; found: C 87.71, H 7.69, N 4.87.

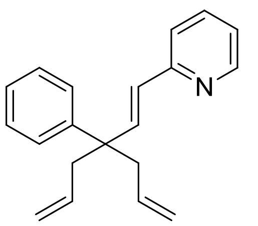

Compound 1a-D. Obtained from **1ac-D** (1.03 mmol, 211 mg) following the representative procedure. Colourless oil (202 mg, 71% over four steps); ^{1}H NMR (500 MHz, $CDCl_3$): δ = 8.53 (ddd, J = 4.8, 1.7, 0.8 Hz, 1H), 7.59 (dt, J = 7.7, 1.8 Hz, 1H), 7.35–7.26 (m, 5H), 7.21–7.16 (m, 1H), 7.09 (ddd, J = 7.5, 4.8, 1.1 Hz, 1H), 6.52 (s, 1H), 5.61 (ddt, J = 17.2, 10.1, 7.1 Hz, 2H), 5.07–4.97 (m, 4H), 2.70 – 2.66 (m, 4H); ^{13}C NMR (125 MHz, $CDCl_3$): δ = 156.0, 149.5, 144.8, 141.4 (t, J = 22.5 Hz), 136.4, 134.4 (2C), 128.6, 128.1 (2C), 127.4 (2C), 126.2, 121.8, 121.0, 117.8 (2C), 46.7, 41.9 (2C); IR (neat): $\tilde{\nu}$ = 3075 (w), 3003 (w), 2978 (w), 2930 (w), 1638 (m), 1586 (s), 1562 (w), 1494 (w), 1469 (m), 1445 (m), 1429 (m), 1149 (w), 1033 (w), 996 (m), 910 (s), 753 (m), 700 (s) cm^{-1}; HRMS (CI(NH_3)): calcd for ($C_{20}H_{20}DN$ + H): 277.1815; found: 277.1808.

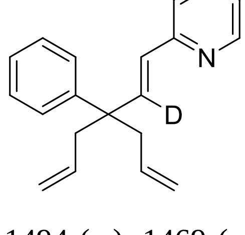

Compound 1b. Obtained from **1bc** (0.75 mmol, 190 mg) following the representative procedure. Yellow

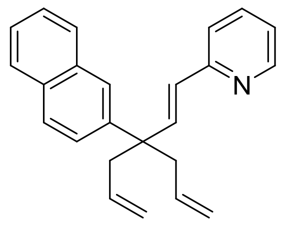

oil (120 mg, 49% over four steps); ^{1}H NMR (400 MHz, $CDCl_3$): δ = 8.55 (dq, *J* = 4.9, 0.7 Hz, 1H), 7.83–7.75 (m, 4H), 7.60 (td, *J* = 7.7, 1.8 Hz, 1H), 7.49 (dd, *J* = 8.6, 1.8 Hz, 1H), 7.47–7.43 (m, 2H), 7.30 (d, *J* = 7.8 Hz, 1H), 7.11 (ddd, *J* = 7.5, 4.8, 1.0 Hz, 1H), 6.94 (d, *J* = 16.3 Hz, 1H), 6.57 (d, *J* = 16.3 Hz, 1H), 5.65 (ddt, *J* = 17.1, 10.1, 7.1 Hz, 2H), 5.12 (dt, *J* = 17.0, 1.2 Hz, 2H), 5.05 (dt, *J* = 10.2, 1.0 Hz, 2H), 2.18 (dd, *J* = 7.1, 1.1 Hz, 4H); ^{13}C NMR (100 MHz, $CDCl_3$): δ = 156.0, 149.5, 142.3, 141.7, 136.4, 134.4 (2C), 133.2, 132.0, 129.1, 128.1, 127.7, 127.4, 126.2, 125.9, 125.8, 125.7, 121.9, 121.1, 117.9 (2C), 46.9, 41.9 (2C); IR (neat): $\tilde{\nu}$ = 3057 (w), 3004 (w), 2976 (w), 2927 (w), 2854 (w), 1638 (w), 1586 (m), 1563. (w), 1505 (w), 1468 (m), 1429 (m), 1351 (w), 1305 (w), 1274 (w), 1244 (w), 1202 (w), 1148 (w), 1131 (w), 1116 (w), 1049 (w), 1018 (w), 990 (m), 978 (m), 946 (w), 911 (s), 854 (m), 817 (s), 767 (s), 744 (s), 683 (w) cm^{-1}; MS (CI(CH_4)): *m*/*z* (rel. intensity): 326 (100) [M + H], 284 (22); HRMS (CI(CH_4)) calcd for ($C_{24}H_{23}N$ + H): 326.1909; found: 326.1900; elemental analysis (%) calcd for $C_{24}H_{23}N$: C 88.57, H 7.12, N 4.30; found: C 88.65, H 7.18, N 4.22.

Compound 1c. Obtained from **1cc** (0.88 mmol, 210 mg) following the representative procedure. Yellow

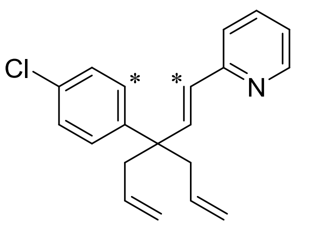

gum (126 mg, 46% over four steps); ^{1}H NMR (500 MHz, $CDCl_3$): δ = 8.57 (ddd, *J* = 4.8, 1.6, 0.6 Hz, 1H), 7.64 (td, *J* = 7.7, 1.8 Hz, 1H), 7.32–7.27 (m, 4H), 7.14 (ddd, *J* = 7.4, 4.8, 0.9 Hz, 1H), 6.87 (d, *J* = 16.2 Hz, 1H), 6.54 (d, *J* = 16.2 Hz, 1H), 5.63 (ddt, *J* = 17.1, 10.2, 7.2 Hz, 2H), 5.11–5.03 (m, 4H), 2.69 (d, *J* = 7.2 Hz, 4H); ^{13}C NMR (125 MHz, $CDCl_3$): δ = 155.7, 149.4, 143.3, 141.2, 136.5, 133.9 (2C), 132.0, 128.9 (3C),[8] 128.2, 121.9, 121.2, 118.2 (2C), 46.6, 41.9 (2C); IR (neat): $\tilde{\nu}$ = 3075 (w), 3005 (w), 2978 (w), 2925 (w), 1639 (m), 1586 (s), 1563 (m), 1489 (s), 1469 (s), 1429 (s), 1399 (w), 1322 (w), 1258 (w), 1149 (w), 1093 (s), 1050 (w), 1033 (w), 1012 (s), 990 (s), 980 (s), 913 (s), 826 (s), 771 (s), 739 (s) , 691 (w) cm^{-1}; MS (ESI+): *m*/*z* (rel. intensity): 334 (5) [$C_{20}H_{21}N^{37}Cl$ + Na], 332 (15) [$C_{20}H_{21}N^{35}Cl$ + Na], 312 (34) [$C_{20}H_{21}N^{37}Cl$ + H], 310 (100) [$C_{20}H_{21}N^{35}Cl$ + H]; HRMS (ESI) calcd for ($C_{20}H_{21}N^{37}Cl$ + H): 312.1333; found: 312.1332; calcd for ($C_{20}H_{21}N^{35}Cl$ + H): 310.1363; found: 310.1357; elemental analysis (%) calcd for $C_{20}H_{20}ClN$: C 77.53, H 6.51, N 4.52; found: C 77.07, H 6.61, N 4.33.

Compound 1d. Obtained from **1dc** (1.41 mmol, 309 mg,) following the representative procedure.

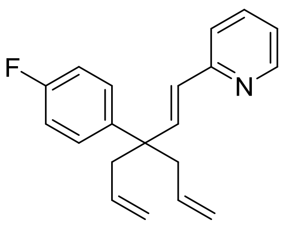

Colourless oil (198 mg, 48% over four steps); ^{1}H NMR (500 MHz, $CDCl_3$): δ = 8.53 (ddd, *J* = 4.9, 1.7, 0.9 Hz, 1H), 7.60 (td, *J* = 7.7, 1.8 Hz, 1H), 7.30–7.25 (m, 3H), 7.10 (ddd, *J* = 7.5, 4.9, 1.1 Hz, 1H), 7.01–7.94 (m, 2H), 6.84 (d, *J* = 16.3 Hz, 1H), 6.51 (d, *J* = 16.3 Hz, 1H), 5.61 (ddt, *J* = 17.2, 10.1,7.1 Hz, 2H), 5.08–4.98 (m, 4H), 2.65 (d, *J* = 7.1 Hz, 4H); ^{13}C NMR (125 MHz, $CDCl_3$): δ = 161.2 (d, *J* = 243.4 Hz), 155.8, 149.5, 141.6, 140.4 (d, *J* = 2.8 Hz), 136.5, 134.1 (2C), 129.0 (d, *J* = 7.5 Hz) (2C), 128.8, 122.0, 121.2, 118.1 (2C), 114.8 (d, *J* = 28.8 Hz) (2C), 46.4, 42.1 (2C); IR (neat): $\tilde{\nu}$ = 3074 (w), 3005 (w), 2978 (w), 2925 (w), 1638 (w), 1584 (m), 1563 (w), 1508 (s), 1469 (m), 1429 (m), 1323 (w), 1307 (w), 1230 (s), 1163 (m), 1149 (w), 1103 (w), 1049 (w), 1014 (w), 991 (m), 915 (s), 833 (s), 816 (m), 770 (m), 743 (m), 674 (w) cm^{-1}; MS (CI(CH_4)): *m*/*z* (rel. intensity): 294 (100) [M + H]; HRMS (CI(CH_4)) calcd for ($C_{20}H_{20}FN$ +H): 294.1653; found: 294.1660; elemental analysis (%) calcd for $C_{20}H_{20}FN$: C 81.88, H 6.87, N 4.77; found: C 82.14, H 7.17, N 4.50.

---

[8] The carbon atoms marked with an asterisk gave overlapped signals, as confirmed by HSQC.

Compound 1e. Obtained from **1ec** (1.23 mmol, 286 mg) following the representative procedure. Yellow oil (160 mg, 43% over four steps); ^{1}H NMR (500 MHz, $CDCl_3$): δ = 8.53 (dd, *J* = 5.0, 1.1 Hz, 1H), 7.60 (td, *J* = 7.8, 1.6 Hz, 1H), 7.30 (d, *J* = 7.6 Hz, 1H), 7.25 (d, *J* = 8.4 Hz, 2H), 7.10 (dd, *J* = 7.5, 5.5 Hz, 1H), 6.85–6.79 (m, 3H), 6.52 (d, *J* = 16.2 Hz, 1H), 5.63 (ddt, 17.1, 10.0, 7.1 Hz, 2H), 5.07–4.99 (m, 4H), 3.79 (s, 3H), 2.66 (d, *J* = 7.1 Hz, 4H); ^{13}C NMR (125 MHz, $CDCl_3$): δ = 157.8, 156.0, 149.4, 142.1, 136.7, 136.4, 134.5 (2C), 128.49, 128.46 (2C), 121.7, 120.9, 117.7 (2C), 113.4 (2C), 55.1, 46.1, 41.9 (2C); IR (neat): $\tilde{\nu}$ = 3073 (w), 3005 (w), 2975 (w), 2931 (w), 2835 (w), 1639 (w), 1608 (w), 1584 (m), 1563 (w), 1511 (s), 1467 (m), 1430 (m), 1292 (w), 1249 (s), 1182 (s), 1149 (w), 1115 (w), 1033(s), 992 (m), 914 (s), 829 (s), 802 (w), 770 (m), 744 (m) cm^{-1}; MS (CI(NH_3)): *m*/*z* (rel. intensity): 306 (100) [M + H]; HRMS (CI(NH_3)) calcd for ($C_{21}H_{23}NO$ + H): 306.1858; found: 306.1854.

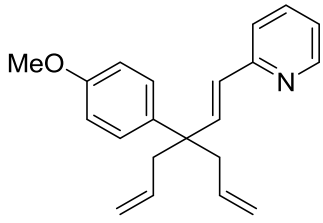

Compound 1f. Obtained from **1fc** (1.79 mmol, 469 mg) following the representative procedure. Yellow oil (316 mg, 52% over four steps); ^{1}H NMR (500 MHz, $CDCl_3$): δ = 8.50–8.48 (m, 1H), 7.55 (td, *J* = 7.6, 1.8 Hz, 1H), 7.25 (d, *J* = 6.4 Hz, 1H), 7.05 (dd, *J* = 7.3, 4.9 Hz, 1H), 6.85 (dd, *J* = 8.2, 2.1 Hz, 1H), 6.83 (s, 1H), 6.83–6.79 (m, 1H), 6.78 (d, J = 8.4 Hz), 6.48 (d, *J* = 16.3 Hz, 1H), 5.59 (ddt, *J* = 17.2, 10.2, 7.1 Hz, 2H), 5.08–5.00 (m, 4H), 3.81 (s, 3H), 3.79 (s, 3H), 2.62 (d, *J* = 7.1 Hz, 4H); ^{13}C NMR (125 MHz, $CDCl_3$): δ = 155.9, 149.4, 148.5, 147.3, 141.9, 137.3, 136.4, 134.5 (2C), 128.5, 121.7, 120.9, 119.6, 117.8 (2C), 111.1, 110.6, 55.9, 55.8, 46.4, 41.9 (2C); IR (neat): $\tilde{\nu}$ = 3073 (w), 3001 (w), 2975 (w), 2931 (w), 2834 (w), 1639 (w), 1584 (s), 1563 (w), 1515 (s), 1465 (s), 1441 (m), 1430 (m), 1408 (w), 1333 (w), 1256 (s), 1234 (s), 1149 (s), 1027(s), 991 (m), 914 (s), 854 (w), 807 (w), 770 (s), 744 (w) cm^{-1}; MS (CI(CH_4)): *m*/*z* (rel. intensity): 364 (12), 336 (100) [M + H], 294 (27); HRMS (CI(NH_3)) calcd for ($C_{22}H_{25}NO_2$ + H): 336.1958; found: 336.1971.

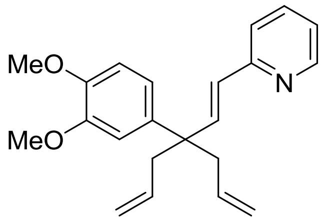

Compound 1g. Obtained from **1gc** (0.78 mmol, 193 mg) following the representative procedure. White waxy solid (120 mg, 48% over four steps); ^{1}H NMR (500 MHz, $CDCl_3$): δ = 8.52 (d, *J* = 4.7 Hz, 1H), 7.60 (d, *J* = 7.9 Hz, 1H), 7.56 (d, *J* = 7.6 Hz, 1H), 7.49 (s, 1H), 7.49–7.43 (m, 1H), 7.23 (t, *J* = 7.7 Hz, 1H), 7.19 (d, *J* = 7.8 Hz, 1H), 7.15–7.06 (m, 2H), 6.90 (d, *J* = 16.2 Hz, 1H), 6.48 (d, *J* = 16.0 Hz, 1H), 5.68 (ddt, *J* = 17.1, 10.0, 7.2 Hz, 2H), 5.05 (d, *J* = 16.8 Hz, 2H), 5.02 (d, *J* = 10.1 Hz, 2H), 2.82 (dd, *J* = 14.0, 7.0, 2H), 2.71 (dd, *J* = 13.9, 7.3 Hz, 2H); ^{13}C NMR (125 MHz, $CDCl_3$): δ = 155.8, 155.6, 149.5, 142.1, 139.6, 136.4, 133.8 (2C), 129.6, 126.5, 124.3, 124.0, 122.1, 122.0, 121.9, 121.4, 118.1 (2C), 42.9, 41.0 (2C); IR (neat): $\tilde{\nu}$ = 3076 (w), 3007 (w), 2978 (w), 2923 (w), 2845 (w), 1650 (w), 1637 (w), 1584 (s), 1562 (m), 1471 (m), 1453 (s), 1428 (s), 1337 (w), 1321 (w), 1307 (w), 1263 (w), 1253 (w), 1209 (w), 1147 (w), 1109 (s), 1093 (m), 1049 (w), 1033 (w), 991 (s), 981 (s), 921 (s), 858 (s), 809 (w), 767 (s), 753 (s), 743 (s) cm^{-1}; MS (CI(CH_4)): *m*/*z* (rel. intensity): 316 (100) [M + H]; HRMS (CI(CH_4)) calcd for ($C_{22}H_{21}NO$ + H): 316.1701; found: 316.1701.

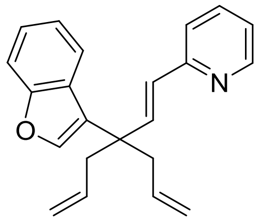

Compound 1h. Obtained from **1hc** (0.56 mmol, 119 mg) following the representative procedure. Colourless oil (86 mg, 52% over four steps); ^{1}H NMR (500 MHz, $CDCl_3$): δ = 8.53 (ddd, *J* = 4.8, 1.7, 0.8 Hz, 1H), 7.58 (dt, *J* = 7.7, 1.8 Hz, 1H), 7.32 (d, *J* = 7.9 Hz, 1H), 7.07 (ddd, *J* = 7.5, 4.8, 1.1 Hz, 1H), 6.82 (d, *J* = 16.5 Hz, 1H), 6.50 (d, *J* = 16.5 Hz, 1H), 5.94–5.84 (m, 2H), 5.04 (dt, *J* = 17.1, 1.5 Hz, 2H), 5.00 (dt, *J* = 10.2, 1.1 Hz, 2H), 3.97–3.88 (m, 4H), 2.49 (dd, *J* = 14.4, 7.7 Hz, 2H), 2.44 (dd, *J* = 14.4, 6.8 Hz, 2H), 1.28 (s, 3H); ^{13}C NMR (125 MHz, $CDCl_3$): δ = 156.1, 149.4, 139.2, 136.3, 135.5 (2C), 130.1, 121.7, 120.9, 116.5 (2C), 113.1, 64.8 (2C), 50.6, 36.6 (2C), 21.1; IR (neat): $\tilde{\nu}$ = 3073 (w), 2979 (m), 2939 (w), 2883 (w), 1638 (w), 1586 (s), 1563 (m), 1469 (m), 1430 (m), 1373 (m), 1304 (w), 1266 (w), 1198 (s), 1149 (m), 1127 (m), 1100 (m), 1083 (m), 1036 (s), 991 (s), 950 (m), 910 (s), 852 (w), 765 (m), 742 (w) cm^{-1}; MS (CI(CH_4)): *m*/*z* (rel. intensity): 286 (100) [M + H]; HRMS

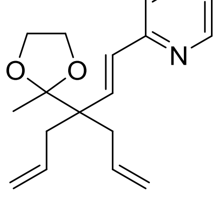

(CI(CH_4)): calcd for ($C_{18}H_{23}NO_2$ + H): 286.1802; found: 286.1804; elemental analysis (%) calcd for $C_{18}H_{23}NO_2$: C 75.76, H 8.12, N 4.91; found: C 76.44, H 8.33, N 4.93.

Compound 1i. Obtained from **1ic** (1.39 mmol, 430 mg) following the representative procedure. Yellow

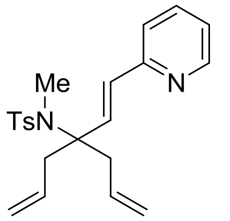

oil (320 mg, 60% over four steps); ^{1}H NMR (500 MHz, $CDCl_3$): δ = 8.50 (d, *J* = 4.6 Hz, 1H), 7.65 (d, *J* = 8.2 Hz, 2H), 7.60 (td, *J* = 7.7, 1.5 Hz, 1H), 7.21–7.15 (m, 3H), 7.11 (dd, *J* = 7.4, 4.9 Hz, 1H), 6.58 (d, *J* = 16.3 Hz, 1H), 6.44 (d, *J* = 16.3 Hz, 1H), 5.72 (ddt, *J* = 17.1, 10.1 and 7.0 Hz, 2H), 5.10 (d, *J* = 16.2 Hz, 2H), 5.09 (d, *J* = 9.7 Hz, 2H), 2.97 (s, 3H), 2.81 (dd, *J* = 14.2, 7.5 Hz, 2H), 2.64 (dd, *J* = 14.2, 6.4 Hz, 2H), 2.37 (s, 3H); ^{13}C NMR (125 MHz, $CDCl_3$): δ = 154.9, 149.4, 142.7, 139.8, 136.5, 136.4, 133.0 (2C), 130.0, 129.3 (2C), 127.2 (2C), 122.3, 121.6, 118.9 (2C), 66.3, 40.1 (2C), 33.4, 21.4; IR (neat): $\tilde{\nu}$ = 3075 (w), 2977 (w), 2925 (w), 1638 (w), 1584 (m), 1564 (w), 1494 (w), 1469 (m), 1430 (m), 1337 (s), 1304 (m), 1255 (w), 1211 (w), 1153 (s), 1117 (m), 1088 (s), 1049 (w), 1018 (w), 981 (s), 915 (s), 813 (s), 770 (m), 743 (m), 720 (s), 657 (s) cm^{-1}; MS (ESI): *m*/*z* (rel. intensity): 421 (10) [M + K], 405 (72) [M + Na], 383 (100) [M + H]; HRMS (ESI) calcd for ($C_{22}H_{27}N_2O_2S$ + Na): 383.1793; found: 383.1786.

Compound 1j. Obtained from **1jc** (0.649 mmol, 135 mg) following the representative procedure. Yellow

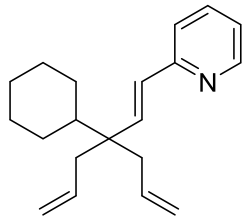

oil (82 mg, 45% over four steps); ^{1}H NMR (500 MHz, $CDCl_3$): δ = 8.53 (d, *J* = 4.7 Hz, 1H), 7.59 (td, *J* = 7.8, 1.8 Hz, 1H), 7.27 (d, *J* = 8.0 Hz, 1H), 7.08 (ddd, *J* = 7.6, 4.7, 0.8 Hz, 1H), 6.69 (d, *J* = 16.2 Hz, 1H), 6.36 (d, *J* = 16.2 Hz, 1H), 5.87–5.74 (m, 2H), 5.07 (d, *J* = 15.9 Hz, 2H), 5.05 (d, *J* = 9.6 Hz, 2H), 2.39 (dd, *J* = 14.2, 6.3 Hz, 2H), 2.77 (dd, *J* = 14.2, 8.3 Hz, 2H), 1.86–1.78 (m, 2H), 1.76–1.70 (m, 2H), 1.64–1.58 (m, 1H), 1.40 (tt, *J* = 11.8, 2.8 Hz), 1.24–0.98 (m, 5H); ^{13}C NMR (125 MHz, $CDCl_3$): δ = 156.1, 149.4, 140.5, 136.3, 135.0 (2C), 128.9, 121.5, 121.0, 117.2 (2C), 44.4, 44.2, 38.4 (2C), 27.3 (2C), 27.0 (2C), 26.5; IR (neat): $\tilde{\nu}$ = 3073 (w), 3004 (w), 2976 (w), 2923 (s), 2851 (s), 1638 (m), 1585 (s), 1563 (m), 1470 (s), 1449 (s), 1429 (s), 1367 (w), 1333 (w), 1148 (w), 1049 (w), 990 (s), 911 (s), 825 (w), 768 (s), 741 (m) cm^{-1}; MS (CI(CH_4)): *m*/*z* (rel. intensity): 282 (100) [M + H], 240 (53); HRMS (CI(CH_4)) calcd for ($C_{20}H_{27}N$ + H): 282.2221; found: 282.2211.

Compound 1k. Obtained from **1kd** (1.23 mmol, 300 mg) following the representative procedure. Yellow

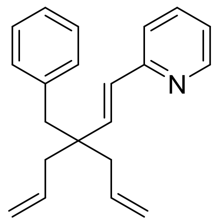

oil (199 mg, 64% over four steps); ^{1}H NMR (500 MHz, $CDCl_3$): δ = 8.56–8.51 (m, 1H), 7.58 (dt, *J* = 7.7, 1.8 Hz, 1H), 7.23–7.12 (m, 6H), 7.09 (ddd, *J* = 7.4, 4.9, 1.0 Hz, 1H), 6.69 (d, *J* = 16.2 Hz, 1H), 6.28 (d, *J* = 16.2 Hz, 1H), 5.92–5.82 (m, 2H), 5.13–5.05 (m, 4H), 2.78 (s, 2H), 2.31–2.20 (m, 4H); ^{13}C NMR (125 MHz, $CDCl_3$): δ = 155.9, 149.4, 141.6, 137.8, 136.4, 134.5 (2C), 130.8 (2C), 128.6, 127.7 (2C), 126.1, 121.7, 121.0, 117.9 (2C), 44.5, 43.0, 40.6 (2C); IR (neat): $\tilde{\nu}$ = 3074 (w), 3029 9 (w), 3005 (w), 2977 (w), 2922 (w), 2852 (w), 1638 (w)m 1603 (w), 1584 (m), 1563 (w), 1495 (w), 1469 (w), 1454 (w), 1429 (m), 1334 (w), 1304 (w), 1262 (w), 1149 (w), 1088 (w), 1049 (w), 1031 (w), 991 (m), 911 (s), 849 (w), 795 (w), 767 (m), 743 (s), 701 (s), 667 (w) cm^{-1}; elemental analysis (%) calcd for $C_{21}H_{23}N$: C 87.15, H 8.01, N 4.84; found: C 86.98, H 8.20, N 4.75.

Compound 1l. Obtained from **1lb** (0.80 mmol, 197 mg) following the representative procedure. Yellow

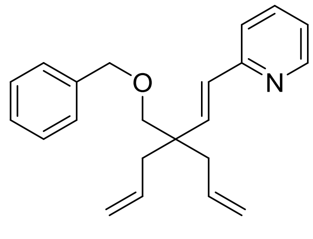

oil (146 mg, 57% over four steps); ^{1}H NMR (500 MHz, $CDCl_3$): δ = 8.54–8.50 (m, 1H), 7.58 (dt, *J* = 7.7, 1.8 Hz, 1H), 7.34 – 7.28 (m, 4H), 7.28 – 7.22 (m, 2H), 7.08 (ddd, *J* = 7.4, 4.9, 1.0 Hz, 1H), 6.67 (d, *J* = 16.3 Hz, 1H), 6.42 (d, *J* = 16.3 Hz, 1H), 5.77 (ddt, *J* = 17.1, 10.0, 7.3 Hz, 2H), 5.09–4.99 (m, 4H), 4.50 (s, 2H), 3.41 (s, 2H), 2.38–2.27 (m, 4H); ^{13}C NMR (125 MHz, $CDCl_3$): δ = 156.0, 149.4, 139.4, 138.7, 136.3, 134.4 (2C), 128.9, 128.3, 127.5 (2C), 127.4 (2C), 121.7, 121.1, 117.7 (2C), 74.1, 73.3, 43.5, 40.0 (2C); IR (neat): $\tilde{\nu}$ = 3074 (w), 3004 (w), 2911 (w), 2857 (m), 1649 (w), 1639 (w), 1585 (s), 1564 (m), 1497 (w), 1470 (m), 1454 (m), 1430 (m), 1363 (w), 1305 (w), 1206 (w), 1095 (s), 1029 (w), 992 (m), 976 (s), 914 (s), 767 (s), 739 (s),

698 (s) cm^{-1}; elemental analysis (%) calcd for $C_{22}H_{25}NO$: C 82.72, H 7.89, N 4.38; found: C 83.06, H 7.92, N 4.34.

Compound 1mb. Under N_2, a solution of LDA (1.41 mmol) {prepared from 0.67 mL of a 2.5 M of nBuLi and 0.20 mL of diisopropylamine in THF (1.8 mL)} at 0 °C, was added to a solution of **1ma** (155 mg, 1.00 mmol) in THF (0.7 mL) at -78°C. The resulting solution was stirred at -78°C for 1.5 hour then methyl iodide (94 µL, 1.51 mmol) was slowly added. The mixture was allowed to warm to room temperature overnight before being quenched with few drops of a saturated aqueous solution of NH_4Cl. The mixture was diluted with H_2O and extracted three times with Et_2O (5 mL). The organic layer was then washed with H_2O and brine, dried over Na_2SO_4, filtered and concentrated. Purification by flash chromatography (petroleum ether/ Et_2O: 29/1) afforded 148 mg of a colourless oil which was dissolved in Et_2O (0.8 mL) and added by cannula to a suspension of $LiAlH_4$ (19 mg, 0.49 mmol) in Et_2O (1.6 mL) at 0 °C. After 10 minutes at this temperature, another portion of $LiAlH_4$ (19 mg, 0.49 mmol) was added. After stirring for 1 hour at room temperature, the reaction mixture was quenched carefully at 0 °C with a saturated aqueous solution of Na_2SO_4. After filtration over Celite of the white precipitate thus formed, the solvent was evaporated under reduced pressure to give 107 mg of alcohol. Under N_2, DMSO (145 µL, 2.04 mmol) in CH_2Cl_2 (0.8 mL) was added to a solution of oxalyl chloride (88 µL, 1.02 mmol) in CH_2Cl_2 (3.9 mL) at –78 °C. After 10 minutes stirring at –78 °C, a solution of the alcohol (107 mg, 0.78 mmol) in CH_2Cl_2 (0.8 mL) was added. After 20 minutes stirring at –78 °C, triethylamine (0.55 mL, 3.92 mmol) was added rapidly and the mixture was stirred at room temperature during 20 minutes. A saturated solution of NH_4Cl was added to the reaction mixture which was then extracted three times with diethyl ether. The organic layer was washed with H_2O and brine, dried over Na_2SO_4, filtered, and concentrated to give 96 mg of aldehyde. Under N_2, nBuLi (0.39 mL, 0.81 mmol, 2.1 M in hexanes) was added to a -78 °C solution of 2-methylpyridine (80 µL, 0.81 mmol) in THF (2.0 mL). After 10 minutes of stirring at -78 °C, a solution of aldehyde (82 mg, 0.60 mmol) in THF (1 mL) was added by cannula. After stirring for 15 minutes at -78°C, the reaction was quenched with enough MeOH to turn the deep red solution to yellow and then allowed to warm to room temperature. The crude mixture was then partitioned between a saturated solution of $NaHCO_3$, water and ethyl acetate and then extracted three times with ethyl acetate (5 mL). The combined organic layers were dried over Na_2SO_4, filtered and concentrated. Purification by flash column chromatography (petroleum ether/EtOAc: 5/1) afforded **1mb** as a clear oil (98 mg, 42% over four steps): ^{1}H NMR (500 MHz, $CDCl_3$): δ = 8.48–8.43 (m, 1H), 7.59 (dt, *J* = 7.6, 1.8 Hz, 1H), 7.15–7.09 (m, 2H), 5.95–5.82 (m, 2H), 5.10–5.00 (m, 4H + 1H(OH)), 3.80 (dd, *J* = 10.5, 1.7 Hz, 1H), 2.90 (dd, *J* = 14.6, 1.7 Hz, 1H), 2.80 (d, *J* = 14.5, 10.4 Hz, 1H), 2.33–2.23 (m, 2H), 2.14 (dd, *J* = 13.9, 7.5 Hz, 1H), 2.03 (dd, *J* = 13.7, 7.8 Hz, 1H), 0.94 (s, 3H); ^{13}C NMR (125 MHz, $CDCl_3$): δ = 161.0, 148.6, 136.8, 135.4, 135.2, 123.7, 121.4, 117.24, 117.19, 75.7, 40.6, 40.4, 40.2, 37.7, 20.5; IR (neat): $\tilde{\nu}$ = 3351 (br w), 3074 (m), 2975 (m), 2917 (m), 1638 (m), 1597 (s), 1570 (m), 1475 (m), 1438 (s), 1375 (w), 1317 (w), 1145 (w), 1097 (w), 1049 (m), 999 (m), 912 (s), 756 (s) cm^{-1}; elemental analysis (%) calcd for $C_{15}H_{21}NO$: C 77.88, H 9.15, N 6.05; found: C 77.79, H 9.17, N 5.88.

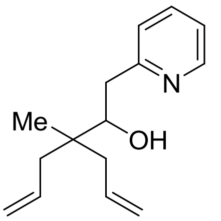

Compound 1m. At 0 °C, methanesulfonyl chloride (30 µL, 0.39 mmol) was added dropwise to a solution of **1mb** (75mg, 0.32 mmol) and Et_3N (68 µL, 0.49 mmol) in CH_2Cl_2 (0.64 mL). After stirring at room temperature overnight, the crude mixture was partitioned between a saturated solution of $NaHCO_3$ and ethyl acetate, and extracted three times with ethyl acetate (5 mL). The combined organic layers were washed with water, then brine, then dried over Na_2SO_4, filtered and concentrated. Under N_2, the residue was dissolved in THF (0.32 mL). At 0 °C, NaHMDS (0.5 mL, 0.49 mmol, 1M in THF) was added. After stirring for 2 hours at room temperature, the reaction mixture was quenched with a saturated solution of $NaHCO_3$ and extracted three times with ethyl acetate and the combined extracts were washed with water, then brine, then dried over Na_2SO_4, filtered and concentrated. Purification by flash column chromatography (petroleum ether/Et_2O: 6/1 → 4/1) afforded **1m** as a clear oil (47 mg, 68 %): ^{1}H NMR (500 MHz, $CDCl_3$): δ = 8.52 (ddd, *J* = 4.9, 1.7, 0.8 Hz, 1H), 7.59 (dt, *J* = 7.7, 1.8 Hz,

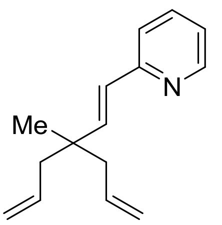

1H), 7.27–7.22 (m, 1H), 7.08 (ddd, *J* = 7.5, 4.9, 1.0 Hz, 1H), 6.69 (d, *J* = 16.2 Hz, 1H), 6.37 (d, *J* = 16.1 Hz, 1H), 5.82 – 5.72 (m, 2H), 5.06 – 4.99 (m, 4H), 2.22 (dd, *J* = 13.7, 7.0 Hz, 2H), 2.15 (dd, *J* = 13.7, 7.7 Hz, 2H), 1.08 (s, 3H); ^{13}C NMR (125 MHz, $CDCl_3$): δ = 156.1, 149.4, 143.0, 136.4, 134.8 (2C), 127.6, 121.6, 121.1, 117.4 (2C), 45.0 (2C), 39.3, 23.3; IR (neat): $\tilde{\nu}$ = 3075 (m), 3004 (w), 2976 (m), 2915 (m), 1640 (m), 1585 (s), 1564 (m), 1471 (m), 1430 (s), 1377 (w), 1321 (w), 1149 (w), 992 (m), 913 (s), 766 (m), 742 (w) cm^{-1}; elemental analysis (%) calcd for $C_{15}H_{19}N$: C 84.46, H 8.98, N 6.57; found: C 84.17, H 9.11, N 6.13.

Preparation of compounds 4a–4d and 4a-D

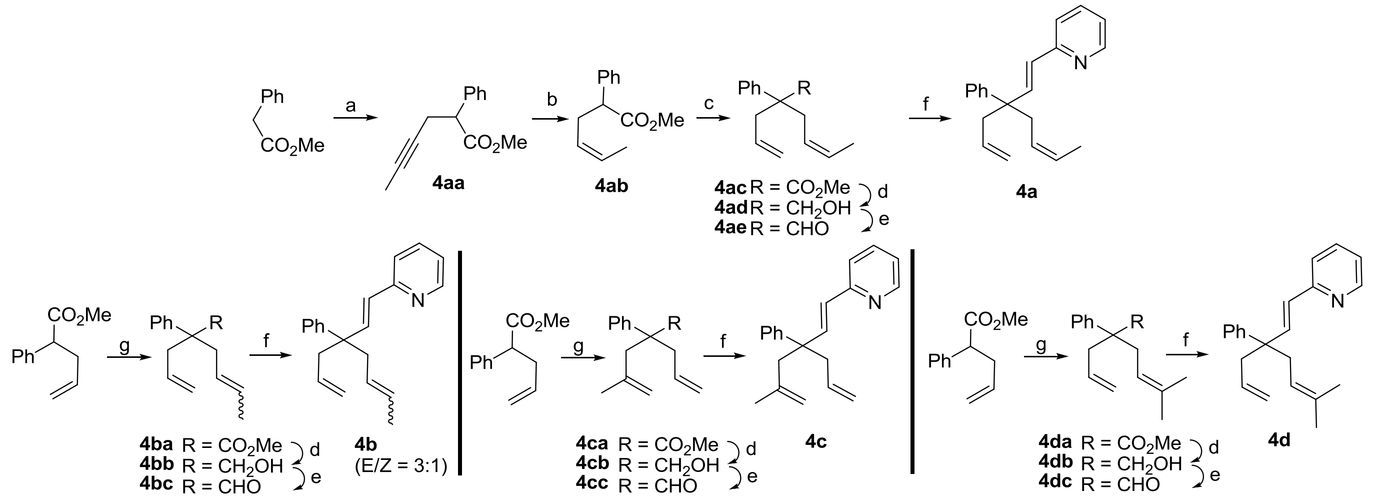

[a] i) LDA, THF, -78 °C; ii) 1-bromo-2-butyne, -78 °C to rt, 92%. [b] $Ni(OAc)_2$, H_2, $NaBH_4$, EtOH, 89%. [c] (i) LiHMDS, THF, -78 °C; ii) allyl bromide, -78 °C to rt, 89%. [d] $LiAlH_4$, THF, 93% (**4ad**), 80% (**4bb**), 93% (**4cb**), 90% (**4db**). (e) $(COCl)_2$, DMSO, Et_3N, CH_2Cl_2, 82% (**4ae**), 93% (**4bc**), 73% (**4cc**), 82% (**4dc**). [f] i) nBuLi, 2-picoline, THF, -78 °C, ii) **4ae** or **4bc–4dc**, -78 °C to rt, iii) MsCl, Et_3N, CH_2Cl_2, 0 °C to rt, iv) NaHMDS, THF, 0 °C, 79% (**4a**), 46% (**4b**), 76% (**4c**), 62% (**4d**). [g] i) LDA, THF, -78 °C; ii) 1-bromo-3-butene, -78 °C to rt, 87% (**4ba**), 89% (**4ca**), 85% (**4da**).

Compound 4aa. To a solution of $^{i}Pr_2NH$ (9.64 mmol, 1.36 ml) in THF (20 ml), nBuLi (2.5 M in hexane, 9.64 mmol, 3.85 ml) was added dropwise at 0˚C before stirring for 15 minutes 0˚C. Then, a solution of methyl phenylacetate (9.19 mmol, 1.37 g) in THF (10 ml) was slowly added at -78˚C before stirring for 45 minutes and then adding 1-bromo-2-butyne (9.64 mmol, 0.84 ml). After 30 minutes stirring at -78˚C, the mixture was stirred overnight warming to room temperature. The resulting mixture was quenched with a saturated solution of NH_4Cl (3 ml) and it was extracted with EtOAc (3x10 ml). The combined organic layers were dried over $MgSO_4$ and concentrated in vacuo. Purification by flash chromatography (petroleum ether/EtOAC, 90:1) afforded **4aa** as a colourless oil (1.3 g, 92%). ^1H NMR (500 MHz, $CDCl_3$): δ = 7.35–7.24 (m, 5H), 3.74 (dd, J = 8.4, 6.9 Hz, 1H), 3.67 (s, 3H), 2.86 (ddq, J = 16.4, 8.7, 2.5 Hz, 1H), 2.52 (ddq, J = 16.4, 6.8, 2.5 Hz, 1H), 1.71 (t, J = 2.5, 3H); ^{13}C NMR (100 MHz, $CDCl_3$): δ = 173.3, 137.9, 128.7 (2C), 127.7 (2C), 127.6, 76.1, 52.2, 51.3, 41.2, 23.5, 3.5; IR (neat): $\tilde{\nu}$ = 3065 (w), 3031 (w), 2952 (w), 2920 (w), 2845 (w), 1734 (s), 1603 (w), 1585 (w), 1496 (w), 1455 (m), 1435 (m), 1350 (m), 1314 (w), 1293 (m), 1269 (m), 1226 (m), 1197 (m), 1162 (s), 1079 (w), 1056 (w), 1031 (w), 1005 (w), 988 (w), 961 (w), 927 (w), 879 (w), 837 (w), 812 (w), 788 (w), 765 (w), 727 (m), 697 (s) cm^{-1}; MS (CI): m/z (rel. intensity): 220 (100) [M + NH_4], 203 (21) [M + H]; elemental analysis (%) calcd for $C_{13}H_{14}O_2$: C 77.20, H 6.98; found: C 77.29, H 7.07.

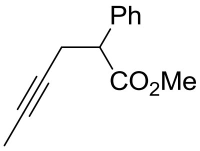

Compound 4ab. A solution of $Ni(OAc)_2$ (1.74 mmol, 0.38 g) in EtOH (2 ml) was purged with H_2. Then, a solution of $NaBH_4$ (4.19 mmol, 0.16 g) in EtOH (4 ml) was added and the mixture was stirred for 10 minutes. Ethylenediamine (19.32 mmol, 1.3 ml) was added before stirring for further 10 minutes. Then, a solution of **4aa** (5.44 mmol, 1.1 g) in EtOH (54 ml) was added. After 90 minutes stirring at room temperature, the suspension was filtered through celite and washed with Et_2O. After concentration of the solution, purification by flash chromatography (petroleum ether/EtOAC, 45:1) afforded **4ab** as a colourless oil (988 mg, 89%).^1H NMR (500 MHz, $CDCl_3$): δ = 7.32–7.28 (m, 4H), 7.26–7.22 (m, 1H), 5.47 (dqt, J = 10.9, 6.8, 1.3 Hz, 1H), 5.26 (dtq, J = 10.7, 7.3, 1.8 Hz, 1H), 3.64 (s, 3H), 3.56 (t, J = 7.8 Hz, 1H), 2.80 (dt, J = 14.3, 7.9 Hz, 1H), 2.49 (dt, J = 14.4, 7.3 Hz, 1H), 1.55 (d, J = 6.7 Hz, 3H); ^{13}C NMR (100 MHz, $CDCl_3$): δ = 174.1, 138.7, 128.5 (2C), 127.9 (2C), 127.2, 126.7, 126.3, 51.9, 51.5, 30.9, 12.8; IR (neat): $\tilde{\nu}$ = 3018 (w), 2951 (w), 1735 (s), 1602 (w), 1495 (w), 1454 (m), 1435 (m), 1405 (w), 1337 (w), 1270 (m), 1222 (m), 1193 (m), 1162 (s), 1107 (w), 1075 (w), 1033 (w), 929 (w), 842 (w), 773 (w), 733 (w), 699 (m) cm^{-1}; MS (CI): m/z (rel. intensity):

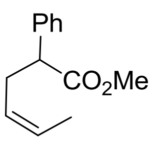

222 (100) [M + NH_4], 205 (41) [M + H]; elemental analysis (%) calcd for $C_{13}H_{16}O_2$: C 76.44, H 7.90; found: C 76.81, H 8.09.

Compound 4ac. LiHMDS (5.2 mmol, 0.86 g) was added as solid in one portion to a solution of **4ab** (4.4 mmol, 0.89 g) in THF (6 mL) at -78 °C under N_2. After 20 minutes stirring at this temperature, allyl bromide (5.2 mmol, 0.47 mL) was added via syringe and the mixture was stirred at room temperature for 1h. The mixture was then quenched with a saturated aqueous solution of NH_4Cl (3 mL) and extracted with EtOAc (3 × 10 mL). Combined organic layers were dried over $MgSO_4$ and concentrated in vacuo. Purification by flash chromatography (petroleum ether/EtOAc, 45:1) afforded 4c as a colourless oil (914 mg, 89%). ^{1}H NMR (500 MHz, $CDCl_3$): δ = 7.34–7.28 (m, 2H), 7.25–7.20 (m, 3H), 5.56–5.45 (m, 2H), 5.18-5.08 (m, 1H), 5.02 (d, *J* = 16.1 Hz, 1H), 5.01 (d, *J* = 11.1 Hz, 1H), 3.62 (s, 3H), 2.85–2.64 (m, 4H), 1.52 (d, *J* = 7.3 Hz, 3H); ^{13}C NMR (100 MHz, $CDCl_3$): δ = 175.8, 141.9, 133.6, 128.3 (2C), 127.1, 126.8, 126.4 (2C), 124.7, 118.5, 54.0, 52.0, 39.1, 31.9, 13.1; IR (neat): $\tilde{\nu}$ = 3063 (w), 3021 (w), 2980 (w), 2950 (w), 1730 (s), 1641 (w), 1600 (w), 1583 (w), 1497 (w), 1445 (w), 1372 (w), 1321 (w), 1275 (w), 1242 (w), 1205 (w), 1137 (w), 1036 (w), 997 (w), 917 (m), 847 (w), 802 (w), 779 (w), 736 (w), 698 (s), 671 (w) cm^{-1}; MS (CI): *m/z* (rel. intensity): 262 (33) [M + NH_4], 245 (100) [M + H]; elemental analysis (%) calcd for $C_{16}H_{20}O_2$: C 78.65, H 8.25; found C: 78.83, H 8.41.

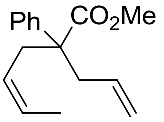

Compound 4ba. To a solution of $^iPr_2NH$ (5.5 mmol, 0.8 ml) in THF (8 ml), nBuLi (2.5 M in hexane, 5.3 mmol, 2.12 ml) was added dropwise at 0˚C before stirring for 15 minutes 0˚C. Then, a solution of methyl-2-(2-allyl)-phenylacetate (5.3 mmol, 1.01 g) in THF (4 ml) was slowly added -78˚C before stirring for 45 minutes and then adding 1-bromo-but-2-ene (5.5 mmol, 0.57 ml). After stirring for further 30 minutes, Bu_4NI (1.06 mmol, 0.39 g) was added in one portion and the mixture was stirred at room temperature for 12h. After quenching with a saturated aqueous solution of NH_4Cl and extractions with EtOAc, the combined organic layers were dried over $MgSO_4$ and concentrated in vacuo. Purification by flash chromatography (petroleum ether/EtOAc, 45:1) afforded **4ba** (*E*/*Z* = 3:1) as a colourless oil (1.21 g, 87%). Only the signals corresponding to the *E* isomer are listed below. ^{1}H NMR (500 MHz, $CDCl_3$): δ = 7.33–7.28 (m, 2H), 7.25–7.19 (m, 3H), 5.52–5.41 (m, 2H), 5.16–5.07 (m, 1H), 5.10–5.02 (m, 2H), 3.64 (s, 3H), 2.80–2.70 (m, 3H), 2.70–2.63 (m, 1H), 1.60 (d, *J* =6.4 Hz, 3H); ^{13}C NMR (100 MHz, $CDCl_3$): δ = 175.4, 141.8, 133.3, 129.0, 128.1 (2C), 126.6, 126.2 (2C), 125.4, 118.2, 53.6, 51.7, 38.8, 37.4, 17.9; IR (neat): $\tilde{\nu}$ = 3063 (w), 3026 (w), 2981 (w), 2950 (w), 1730 (s), 1640 (w), 1600 (w), 1583 (w), 1498 (w), 1444 (m), 1378 (w), 1320 (w), 1296 (w), 1264 (w), 1240 (w), 1205 (s), 1137 m), 1070 (w), 1035 (w), 997 (w), 969 (m), 943 (w), 916 (m), 846 (w), 778 (w), 758 (w), 736 (w), 698 (s) cm^{-1}; elemental analysis (%) calcd for $C_{16}H_{20}O_2$: C 78.65, H 8.25; found: C 79.95, H 8.41.

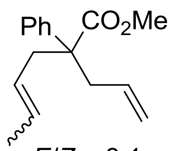

E/*Z* = 3:1

Compound 4ca. This compound was obtained from methyl-2-(2-allyl)-phenylacetate (5.62 mmol, 1.07 g), according to the same procedure described for the preparation of **4ba**. Colourless oil (1.22 g, 89%). ^{1}H NMR (500 MHz, $CDCl_3$): δ = 7.38–7.31 (m, 2H), 7.31-7.23 (m, 3H), 5.52 (ddt, *J* = 17.3, 10.1, 7.1 Hz, 1H), 5.07–4.99 (m, 2H), 4.83 (s, 1H), 4.66 (s, 1H), 3.61 (s, 3H), 2.90–2.78 (m, 3H), 2.72 (d, *J* = 13.9 Hz, 1H), 1.60 (s, 3H); ^{13}C NMR (100 MHz, $CDCl_3$): δ = 175.9, 142.3, 141.6, 133.7, 128.3 (2C), 126.8, 126.5 (2C), 118.2, 115.5, 53.3, 51.9, 42.4, 38.7, 23.8; IR (neat): $\tilde{\nu}$ = 3077 (w), 2950 (w), 1729 (s), 1641 (w), 1599 (w), 1583 (w), 1498 (w), 1446 (m), 1376 (w), 1327 (w), 1273 (w), 1262 (w), 1238 (w), 1203 (s), 1135 (w), 1115 (w), 1080 (w), 1067 (w), 1053 (w), 1034 (w), 991 (w), 915 (m), 896 (m), 858 (w), 824 (w), 802 (w), 771 (w), 734 (w), 698 (s) cm^{-1}; elemental analysis (%) calcd for $C_{16}H_{20}O_2$: C 78.65, H 8.25; found: C 77.82, H 8.33.

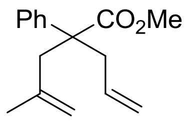

Compound 4da. This compound was obtained from methyl-2-(2-allyl)-phenylacetate (3.60 mmol, 684 mg), according to the same procedure described for the preparation of **4ba**. Colourless oil (789 mg, 85%). ^{1}H NMR (500 MHz, $CDCl_3$): δ = 7.32–7.27 (m, 2H), 7.25–7.20 (m, 3H), 5.53–5.44 (m, 1H), 5.07–4.99 (m, 2H), 4.87 (t, *J* = 6.6 Hz, 1H), 3.61 (s, 3H), 2.79–2.68 (m, 3H), 2.62 (dd, *J* = 14.4, 6.9 Hz, 1H), 1.63 (s, 3H), 1.50 (s, 3H); ^{13}C NMR (100 MHz, $CDCl_3$): δ = 175.8, 142.1, 134.7, 133.6, 128.2 (2C), 126.7 (2C), 126.4, 118.6, 118.3, 54.1, 51.9, 39.2, 33.1, 26.0, 17.9; IR (neat): $\tilde{\nu}$ = 3062 (w), 2950 (w), 2917 (w), 2858 (w), 1730 (s), 1641 (w), 1600 (w), 1583 (w), 1497 (w), 1446 (m), 1377 (w), 1321 (w), 1273 (w), 1215 (s), 1173 (m), 1138 (w), 1115 (w), 1077 (w), 1061 (w), 1035 (w), 996 (w), 916 (m), 892 (w), 852 (w), 773 (w), 737 (w), 698 (s) cm^{-1}; elemental analysis (%) calcd for $C_{17}H_{22}O_2$: C 79.03, H 8.58; found: C 78.87, H 8.69.

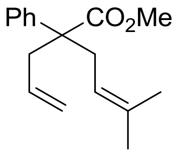

Compound 4ad. This compound was obtained from **4ac** (3.60 mmol, 880 mg), according to the same procedure described for the preparation of **1ac**. Colourless oil (726 mg, 93%). ^{1}H NMR (500 MHz, $CDCl_3$): δ = 7.35–7.23 (m, 4H), 7.23–7.18 (m, 1H), 5.64 (ddt, *J* = 17.3, 10.1, 7.2 Hz, 1H), 5.54–5.46 (m, 1H), 5.27–5.19 (m, 1H), 5.07 (d, *J*=17.0 Hz, 1H), 5.01 (d, *J* = 10.1 Hz, 1H), 3.78 (d, *J* = 6.4 Hz, 2H), 2.56 (dd, *J* = 14.0, 7.1 Hz, 1H), 2.52–2.40 (m, 3H), 1.58 (d, *J* = 7.0 Hz, 3H), 1.26 (t, *J* = 6.6 Hz, 1H(OH)); ^{13}C NMR (100 MHz, $CDCl_3$): δ = 143.6, 134.6, 128.4 (2C), 126.9 (2C), 126.5, 126.2, 125.7, 117.8, 68.1, 46.3, 39.7, 32.4, 13.1; IR (neat): $\tilde{\nu}$ = 3412 (br), 3060 (w), 3019 (w), 2977 (w), 2919 (w), 1638 (w), 1600 (w), 1580 (w), 1498 (w), 1445 (m), 1406 (w), 1371 (w), 1323 (w), 1045 (s), 1000 (w), 984 (w), 914 (s), 865 (w), 769 (w), 750 (w), 699 (s) cm^{-1}; MS (CI): *m/z* (rel. intensity): 234 (100) [M + NH_4], 161 (74), 91 (99); elemental analysis (%) calcd for $C_{15}H_{20}O$: C 83.28, H 9.32; found: C 83.55, H 9.59.

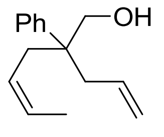

Compound 4bb. This compound was obtained as a *E*/*Z* mixture (3:1) from **4ba** according to the same procedure described for the preparation of **1ac**. (only the *E* isomer is described below) as colourless oil (549 mg, 80%). ^{1}H NMR (500 MHz, $CDCl_3$): δ = 7.37–7.31 (m, 4H), 7.24–7.19 (m, 1H), 5.65–5.55 (m, 1H), 5.54–5.46 (m, 1H), 5.29–5.20 (m, 1H), 5.06 (d, *J* = 16.8 Hz, 1H), 5.00 (d, *J* = 10.4 Hz, 1H), 3.77 (d, *J* = 6.6 Hz, 2H), 2.52–2.35 (m, 2H), 1.60 (d, *J* = 7.0 Hz, 3H), 1.25 (t, *J* = 6.7 Hz, 1H(OH)); ^{13}C NMR (100 MHz, $CDCl_3$): δ = 143.7, 134.5, 128.4 (2C), 126.9 (2C), 126.5, 126.2, 117.7, 68.0, 46.0, 39.7, 38.2, 18.1; IR (neat): $\tilde{\nu}$ = 3411 (br), 3060 (w), 3025 (w), 2917 (w), 2856 (w), 1735 (w), 1638 (w), 1601 (w), 1581 (w), 1498 (w), 1445 (m), 1415 (w), 1377 (w), 1338 (w), 1260 (w), 1140 (w), 1044 (m), 1000 (m), 970 (m), 913 (m), 860 (w), 804 (w), 764 (w), 697 (s) cm^{-1}; MS (CI): *m/z* (rel. intensity): 234 (100) [M + NH_4]; elemental analysis (%) calcd for $C_{15}H_{20}O$: C 83.28, H 9.32; found: C 83.80, H 9.42.

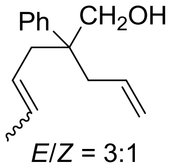

E/*Z* = 3:1

Compound 4cb. This compound was obtained from **4ca** (4.98 mmol, 1.21 g), according to the same procedure described for the preparation of **1ac**. Colourless oil (1 g, 93%). ^{1}H NMR (500 MHz, $CDCl_3$): δ = 7.34–7.25 (m, 4H), 7.22–7.18 (m, 1H), 5.72–5.62 (m, 1H), 5.10 (d, *J* = 17.2 Hz, 1H), 5.02 (d, *J* = 10.1 Hz, 1H), 4.76 (s, 1H), 4.60 (s, 1H), 3.86 (d, *J* = 6.5 Hz, 2H), 2.62 (ddt, *J* = 14.1, 6.6, 1.3 Hz, 1H), 2.51 (dd, *J* = 13.6, 7.8 Hz, 1H), 2.43 (s, 2H), 1.40 (t, *J* = 6.6 Hz, 1H(OH)), 1.29 (s, 3H); ^{13}C NMR (100 MHz, $CDCl_3$): δ = 143.9, 142.9, 134.8, 128.3 (2C), 126.8 (2C), 126.2, 117.9, 114.6, 67.0, 45.7, 44.7, 40.2, 24.4; IR (neat): $\tilde{\nu}$ = 3443 (br), 3024 (w), 2921 (w), 1639 (w), 1600 (w), 1580 (w), 1499 (w), 1445 (m), 1375 (w), 1331 (w), 1033 (m), 1023 (m), 970 (w), 914 (m), 894 (m), 759 (w), 749 (w), 699 (s) cm^{-1}; elemental analysis (%) calcd for $C_{15}H_{20}O$: C 83.28, H 9.32; found: C 83.26, H 9.52.

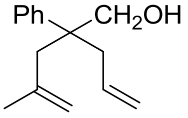

Compound 4db. This compound was obtained from **4ca** (3.55 mmol, 916 mg), according to the same procedure described for the preparation of **1ac**. Colourless oil (735 mg, 90%). ^{1}H NMR (500 MHz, $CDCl_3$): δ = 7.34–7.30 (m, 4H), 7.22–7.18 (m, 1H), 5.62 (ddt, *J* = 17.3, 10.1, 7.5 Hz, 1H), 5.05 (d, *J* = 17.2 Hz, 1H), 5.00 (d, *J* = 10.1 Hz, 1H), 4.96 (t, *J* = 7.2 Hz, 1H), 3.77 (d, *J* = 6.6 Hz, 2H), 2.52 (dd, *J* = 14.1, 7.2 Hz, 1H), 2.47–2.40 (m, 2H), 2.37 (dd, J = 14.6, 7.4 Hz, 1H), 1.63 (s, 3H), 1.56 (s, 3H), 1.27 (t, *J* = 6.6 Hz, 1H(OH)); ^{13}C NMR (100

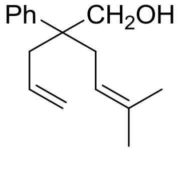

MHz, $CDCl_3$): δ = 43.8, 134.7, 134.1, 128.4 (2C), 127.0 (2C), 126.1, 119.6, 117.8, 68.2, 46.4, 39.8, 33.7, 26.0, 18.0; IR (neat): $\tilde{\nu}$ = 3402 (br), 3059 (w), 2975 (w), 2914 (w), 1639 (w), 1600 (w), 1580 (w), 1498 (w), 1445 (m), 1376 (w), 1321 (w), 1111 (w), 1046 (m), 999 (w), 914 (m), 867 (w), 782 (w), 739 (w), 697 (s) cm^{-1}; elemental analysis (%) calcd for $C_{16}H_{22}O$: C 83.43, H 9.63; found: C 83.17, H 9.77.

Compound 4ad-D. Obtained from **4ac** (0.80 mmol, 195 mg) following the same procedure but using $LiAlD_4$. Colourless oil (174 mg, 99%); ^{1}H NMR (500 MHz, $CDCl_3$): δ = 7.35–7.33 (m, 4H), 7.23–7.18 (m, 1H), 5.64 (ddt, *J* = 17.3, 10.1, 7.2 Hz, 1H), 5.54–5.46 (m, 1H), 5.27–5.19 (m, 1H), 5.07 (d, *J* = 17.0 Hz, 1H), 5.01 (d, *J* = 10.1 Hz, 1H), 2.56 (dd, *J* = 14.0, 7.1 Hz, 1H), 2.52–2.40 (m, 3H), 1.58 (d, *J* = 7.0 Hz, 3H), 1.25–1.21 (m, 1H(OH)); ^{13}C NMR (100 MHz, $CDCl_3$): δ = 143.7, 134.5, 128.3 (2C), 126.8 (2C), 126.1, 125.7, 117.6, 67.1 (quint, *J* = 21.7 Hz), 48.0, 39.5, 32.3, 13.0; IR (neat): $\tilde{\nu}$ = 3407 (br), 3060 (w), 3019 (w), 2977 (w), 2919 (w), 2206 (w), 2096 (w), 1638 (w), 1600 (w), 1580 (w), 1498 (w), 1445 (m), 1406 (w), 1371 (w), 1323 (w), 1045 (m), 1000 (w), 984 (w), 914 (m), 865 (w), 769 (w), 750 (w), 699 (s) cm^{-1}; MS (CI(NH_3)): *m*/*z* (rel. intensity): 236 (100) [M + NH_4]; HRMS (CI(NH_3)) calcd for ($C_{15}H_{18}D_2O$ + NH_4): 236.1983; found: 236.1975.

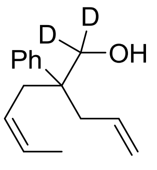

Compound 4ae. Under N_2, DMSO (0.60 mL, 8.40 mmol) in CH_2Cl_2 (3 mL) was added to a solution of oxalyl chloride (0.36 mL, 4.20 mmol) in CH_2Cl_2 (12 mL) at –78 °C. After 10 minutes stirring at –78 °C, a solution of the **4ad** (700 mg, 3.23 mmol) in CH_2Cl_2 (3 mL) was added. After 20 minutes stirring at –78 °C, triethylamine (2.2 mL, 16.2 mmol) was added rapidly and the mixture was stirred at room temperature during 20 minutes. A saturated solution of NH_4Cl was added to the reaction mixture which was then extracted three times with diethyl ether. The organic layer was washed with H_2O and brine, dried over Na_2SO_4, filtered, and concentrated. Purification by flash chromatography (petroleum ether/EtOAc, 75:1) gave **4ae** as colourless oil (568 mg, 82%). ^{1}H NMR (500 MHz, $CDCl_3$): δ = 9.52 (s, 1H), 7.37 (t, *J* = 7.7 Hz, 2H), 7.28 (t, *J* = 7.4 Hz, 1H), 7.20 (d, *J* = 7.8 Hz, 2H), 5.57–5.48 (m, 2H), 5.22–5.14 (m, 1H), 5.04 (d, *J* = 18.0 Hz, 1H), 5.03 (d, *J* = 10.0 Hz, 1H), 2.75–2.60 (m, 4H), 1.53 (d, *J* = 6.9 Hz, 3H); ^{13}C NMR (100 MHz, $CDCl_3$): δ = 202.1, 138.2, 132.9, 128.8 (2C), 127.6 (2C), 127.4 (2C)[9], 124.0, 118.8, 57.4, 36.9, 29.5, 13.1; IR (neat): $\tilde{\nu}$ = 3062 (w), 3022 (w), 2979 (w), 2919 (w), 2804 (w), 2711 (w), 1723 (s), 1640 (w), 1599 (w), 1582 (w), 1496 (w), 1446 (m), 1417 (w), 1384 (w), 1310 (w), 1079 (w), 1033 (w), 997 (w), 918 (m), 873 (w), 839 (w), 756 (m), 699 (s) cm^{-1}; MS (CI): *m*/*z* (rel. intensity): 232 (100) [M + NH_4]; elemental analysis (%) calcd for $C_{15}H_{18}O$: C 84.07, H 8.47; found: C 84.09, H 8.55.

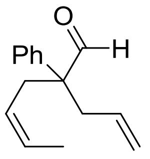

Compound 4bc. This compound was obtained as a *E*/*Z* mixture (3:1) from **4bb** using the same procedure (only the *E* isomer is described below) as colourless oil (313 mg, 93%).^{1}H NMR (500 MHz, $CDCl_3$): δ = 9.49 (s, 1H), 7.37 (t, *J* = 7.7 Hz, 2H), 7.30–7.26 (m, 1H), 7.18 (d, *J* = 8.0 Hz, 2H), 5.55–5.44 (m, 2H), 5.21–5.18 (m, 1H), 5.02 (d, *J* = 17.8 Hz, 1H), 5.01 (d, *J* = 9.2 Hz, 1H), 2.73–2.56 (m, 4H), 1.59 (d, *J* = 5.9 Hz, 3H); ^{13}C NMR (100 MHz, $CDCl_3$): δ = 202.0, 138.1, 132.8, 129.4, 128.6 (2C), 127.5 (2C), 127.2 (2C), 124.7, 118.7, 56.8, 36.7, 35.2, 17.9; IR (neat): $\tilde{\nu}$ = 3061 (w), 3026 (w), 2979 (w), 2918 (w), 2855 (w), 2710 (w), 1722 (s), 1640 (w), 1599 (w), 1582 (w), 1496 (w), 1446 (m), 1418 (w), 1379 (w), 1337 (w), 1192 (w), 1077 (w), 1034 (w), 997 (w), 968 (m), 916 (m), 865 (w), 844 (w), 759 (w), 734 (w), 698 (s) cm^{-1}; MS (CI): *m*/*z* (rel. intensity): 232 (100) [M + NH_4]; elemental analysis (%) calcd for $C_{15}H_{18}O$: C 84.07, H 8.47; found: C 84.35, H 8.56.

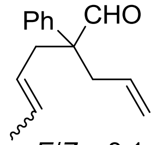

E/*Z* = 3:1

Compound 4cc. This compound was obtained from **4cb** (3.81 mmol, 823 mg), according to the same procedure described for the preparation of **4ae**. Colourless oil (595 mg, 73%). ^{1}H NMR (500 MHz, $CDCl_3$): δ = 9.54 (s, 1H), 7.38–7.34 (m, 2H), 7.30–7.25 (m, 1H), 7.23–7.20 (m, 2H), 5.56 (ddt, *J* = 17.3, 10.1, 7.4 Hz, 1H), 5.07–5.01 (m, 2H), 4.82 (s, 1H), 4.64 (s, 1H), 2.80 (dd, *J* = 13.7, 6.7 Hz, 1H), 2.76–2.65 (m, 3H), 1.40 (s, 3H); ^{13}C NMR (100 MHz,

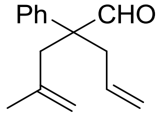

[9] Two overlapped carbons corresponding to the proton at 7.32 ppm (phenyl) and proton at 5.568 (double bond)

$CDCl_3$): δ = 202.0, 141.0, 138.1, 133.0, 128.7 (2C), 127.8 (2C), 127.5, 119.0, 115.7, 56.8, 40.6, 36.7, 24.3; IR (neat): $\tilde{\nu}$ = 3077 (w), 3026 (w), 2979 (w), 2945 (w), 2803 (w), 2713 (w), 1721 (s), 1641 (w), 1599 (w), 1582 (w), 1495 (w), 1446 (m), 1377 (w), 1316 (w), 1284 (w), 1231 (w), 1158 (w), 1090 (w), 1030 (w), 997 (w), 918 (m), 896 (w), 836 (w), 761 (w), 699 (s) cm^{-1}; HRMS (CI(NH_3)): calcd for ($C_{15}H_{18}O$ + H): 215.1430; found: 215.1439.

Compound 4dc. This compound was obtained from **4db** (2.93 mmol, 674 mg), according to the same

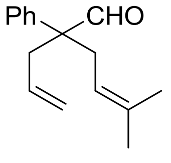

procedure described for the preparation of **4ae**. Colourless oil (568 mg, 82%). ^{1}H NMR (500 MHz, $CDCl_3$): δ = 9.50 (s, 1H), 7.39–7.34 (m, 2H), 7.30–7.25 (m, 1H), 7.21–7.17 (m, 2H), 5.50 (ddt, *J* = 17.3, 10.1, 7.2 Hz, 1H), 5.05–4.97 (m, 2H), 4.93 (t, *J* = 7.3 Hz, 1H), 2.70–2.63 (m, 3H), 2.60 (dd, *J* = 15.2, 7.3 Hz, 1H), 1.63 (s, 3H), 1.53 (s, 3H); ^{13}C NMR (100 MHz, $CDCl_3$): δ = 202.4, 138.4, 135.1, 133.1, 128.7 (2C), 127.7 (2C), 118.5, 117.9, 57.6, 37.1, 30.7, 25.9, 18.0; IR (neat): $\tilde{\nu}$ = 3062 (w), 2979 (w), 2915 (w), 2858 (w), 2711 (w), 1722 (s), 1640 (w), 1599 (w), 1582 (w), 1495 (w), 1446 (m), 1377 (w), 1264 (w), 1242 (w), 1205 (w), 1111 (w), 1030 (w), 997 (w), 913 (m), 880 (w), 760 (w), 698 (s) cm^{-1}; MS (CI(NH_3)): *m/z* (rel. intensity): 246 (100) [M + NH_4].

Compound 4ae-D. Obtained from **4ad-D** (0.77 mmol, 168 mg) following the same procedure. Colourless

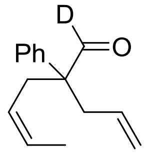

oil (131 mg, 79%); ^{1}H NMR (500 MHz, $CDCl_3$): δ = 7.40–7.33 (m, 2H), 7.30–7.26 (m, 1H), 7.23–7.18 (m, 2H), 5.58–5.48 (m, 2H), 5.21–5.18 (m, 1H), 5.09–4.99 (m, 2H), 2.76–2.60 (m, 4H), 1.53 (d, *J* = 5.9 Hz, 3H); ^{13}C NMR (100 MHz, $CDCl_3$): δ = 201.8, (t, *J* = 25.8 Hz), 138.3, 132.9, 128.8 (2C), 127.7 (2C), 127.4, 127.3, 124.0, 118.7, 57.2, 37.0, 29.7, 13.0; IR (neat): $\tilde{\nu}$ = 3078 (w), 3022 (w), 2979 (w), 2920 (w), 2858 (w), 2058 (w), 1710 (s), 1640 (w), 1599 (w), 1582 (w), 1496 (w), 1446 (w), 1417 (w), 1371 (w), 1309 (w), 1077 (w), 1032 (w), 996 (w), 917 (m), 833 (w), 751 (w), 698 (s) cm^{-1}; MS (CI(CH_4)): *m/z* (rel. intensity): 216 (100) [M + H], 146 (75), 132 (100), 121 (32), 108 (33), 105 (46), 95 (59); HRMS (CI(CH_4)) calcd for ($C_{15}H_{17}DO$ + H): 216.1499; found: 216.1501.

Compound 4a. Under N_2, nBuLi (2.5 mL, 6.32 mmol, 2.5 M in hexanes) was added to a -78 °C solution

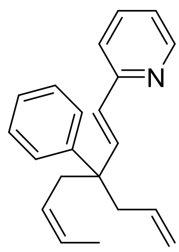

of 2-methylpyridine (0.40 mL, 4.23 mmol) in THF (16 mL). After 10 minutes of stirring at -78 °C, a solution of aldehyde (700 mg, 3.27 mmol) in THF (7 mL) was added by cannula. After stirring for 15 minutes at -78°C, the reaction was quenched with enough MeOH to turn the deep red solution to yellow and then allowed to warm to room temperature. The crude mixture was then partitioned between a saturated solution of $NaHCO_3$, water and EtOAc and then extracted three times with EtOAc (25 mL). The combined organic layers were dried over Na_2SO_4, filtered and concentrated. This material was then diluted in CH_2Cl_2 (7 mL) under N_2. Then, Et_3N (0.70 mL, 4.90 mmol) was added, followed by methanesulfonyl chloride (0.30 mL, 3.92 mmol) at 0 °C. After stirring at room temperature overnight, the crude mixture was partitioned between a saturated solution of $NaHCO_3$ and ethyl acetate, and extracted three times with EtOAc (25 mL). The combined organic layers were washed with water, then brine, then dried over Na_2SO_4, filtered and concentrated. Under N_2, the residue was dissolved in THF (7 mL). At 0 °C, NaHMDS (6.5 mL, 6.5 mmol, 1M in THF) was added. After stirring for 2 hours at room temperature, the reaction mixture was quenched with a saturated solution of $NaHCO_3$ and extracted three times with ethyl acetate and the combined extracts were washed with water, then brine, then dried over Na_2SO_4, filtered and concentrated. Purification by flash column chromatography (petroleum ether/EtOAc = 8:1) afforded **4** as yellow oil (765 mg, 79% over three steps). ^{1}H NMR (500 MHz, $CDCl_3$): δ = 8.53 (ddd, *J* = 4.9, 1.7, 0.8 Hz, 1H), 7.59 (td, *J* = 7.7, 1.8 Hz, 1H), 7.36–7.32 (m, 2H), 7.31–7.26 (m, 3H), 7.21–7.16 (m, 1H), 7.09 (ddd, *J* = 7.5, 4.9, 1.0 Hz, 1H), 6.86 (d, *J* = 16.3 Hz, 1H), 6.54 (d, *J* =16.4 Hz, 1H), 5.63 (ddt, *J* = 17.1, 10.2, 7.0 Hz, 1H), 5.51–5.43 (m, 1H), 5.31–5.23 (m, 1H), 5.06–4.97 (m, 2H), 2.68 (d, *J* =7.1 Hz, 2H), 2.65 (d, *J* =7.0 Hz, 2H), 1.53 (d, *J* = 6.6 Hz, 3H); ^{13}C NMR (100 MHz, $CDCl_3$): δ = 156.2, 149.5, 144.9, 142.0, 136.3, 134.7, 128.7, 128.1 (2C), 127.6 (2C), 126.1 (2C), 126.0, 121.7, 120.9, 117.7, 47.4, 42.3, 34.9, 13.2; IR (neat): $\tilde{\nu}$ = 3059 (w), 3020 (w), 2977 (w),

2916 (w), 2857 (w), 1948 (w), 1647 (w), 1585 (m), 1563 (w), 1495 (w), 1469 (m), 1445 (w), 1429 (m), 1370 (w), 1322 (w), 1255 (w), 1190 (w), 1149 (w), 1049 (w), 1034 (w), 990 (m), 979 (m), 913 (m), 842 (w), 815 (w), 764 (m), 699 (s) cm^{-1}; HRMS (CI(CH_4)) calcd for ($C_{21}H_{23}N$ + H): 290.1903; found: 290.1902.

Compound 4b. This compound was also obtained as a *E*/*Z* mixture (3:1) from **4bc** using the same procedure (only the *E* isomer is described below) as colourless oil (39 mg, 46%).^{1}H NMR (500 MHz, $CDCl_3$): δ = 8.53 (d, *J* = 4.6 Hz, 1H), 7.59 (td, *J* = 7.7, 1.7 Hz, 1H), 7.35–7.26 (m, 6H), 7.21–7.16 (m, 1H), 7.09 (dd, *J* = 7.3, 5.0 Hz, 1H), 6.82 (d, *J* = 16.3 Hz, 1H), 6.51 (d, *J* = 16.2 Hz, 1H), 5.60 (ddt, *J* = 17.1, 10.2, 7.0 Hz, 1H), 5.48–5.39 (m, 1H), 5.29–5.18 (m, 1H), 5.02 (d, *J* = 17.3 Hz, 1H), 4.98 (d, *J* = 11.2 Hz, 1H), 2.65 (d, *J* = 7.0 Hz, 2H), 2.60 (d, *J* = 7.2 Hz, 2H), 1.58 (d, *J* = 6.5 Hz, 3H); ^{13}C NMR (100 MHz, $CDCl_3$): δ = 156.1, 149.4, 145.0, 142.1, 136.4, 134.6, 128.6, 128.4, 128.0 (2C), 127.5 (2C), 126.5, 126.1, 121.7, 120.9, 117.7, 46.9, 41.8, 40.7, 18.1; HRMS (CI(CH_4)): calcd for ($C_{21}H_{23}N$ + H): 290.1903; found: 290.1907.

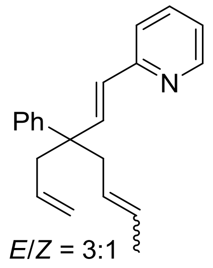

Compound 4a-D. Obtained from **4ae-D** following the same procedure. Yellow oil (70 mg, 80%);^{1}H NMR (500 MHz, $CDCl_3$): δ = 8.52 (d, *J* = 4.9 Hz, 1H), 7.59 (td, *J* = 7.7, 1.8 Hz, 1H), 7.36–7.32 (m, 2H), 7.31–7.26 (m, 3H), 7.20–7.16 (m, 1H), 7.10–7.06 (m, 1H), 6.56–6.51 (m, 1H), 5.63 (ddt, *J* = 17.1, 10.2, 7.0 Hz, 1H), 5.52–5.43 (m, 1H), 5.31–5.24 (m, 1H), 5.06–4.98 (m, 2H), 2.68 (d, *J* = 7.2 Hz, 2H), 2.65 (d, *J* = 7.0 Hz, 2H) 1.53 (d, *J* = 6.8 Hz, 3H); ^{13}C NMR (100 MHz, $CDCl_3$): δ = 156.2, 149.5, 145.0, 141.6 (t, *J* = 23.3 Hz), 136.4, 134.7, 128.7, 128.1 (2C), 127.6 (2C), 126.1, 126.0, 121.7, 120.9, 117.7, 47.3, 42.3, 34.9, 13.2; IR (neat): $\tilde{\nu}$ = 3059 (w), 3020 (w), 2977 (w), 2917 (w), 2856 (w), 1952 (w), 1637 (w), 1585 (m), 1562 (w), 1494 (w), 1468 (w), 1445 (w), 1428 (m), 1370 (w), 1321 (w), 1190 (w), 1149 (w), 1097 (w), 1049 (w), 1030 (w), 994 (w), 911 (m), 778 (w), 744 (m), 699 (s) cm^{-1}; HRMS (CI(CH_4)) calcd for ($C_{21}H_{22}DN$ + H): 291.1972; found: 291.1976.

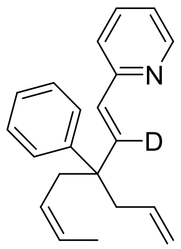

Compound 4c. Yellow oil (331 mg, 76%). ^{1}H NMR (500 MHz, $CDCl_3$): δ = 8.54–8.51 (m, 1H), 7.59 (td, *J* = 7.7, 1.8 Hz, 1H), 7.36–7.32 (m, 2H), 7.31–7.26 (m, 3H), 7.20–7.16 (m, 1H), 7.09 (ddd, *J* = 7.4, 4.9, 0.9 Hz, 1H), 6.89 (d, *J* = 16.4 Hz, 1H), 6.54 (d, *J* = 16.4 Hz, 1H), 5.66 (ddt, *J* = 17.1, 10.2, 6.9 Hz, 1H), 5.08–5.02 (m, 1H), 5.02–4.98 (m, 1H), 4.80 (s, 1H), 4.63 (s, 1H), 2.77 (d, *J* = 7.1 Hz, 2H), 2.68 (d, *J* = 13.4 Hz, 1H), 2.65 (d, *J* = 13.6 Hz, 1H), 1.36 (s, 3H); ^{13}C NMR (125 MHz, $CDCl_3$): δ = 156.1, 149.5, 145.0, 142.3, 142.3, 136.4, 134.9, 128.3, 128.0 (2C), 127.6 (2C), 126.1, 121.7, 120.9, 117.8, 115.2, 46.9, 16.1, 41.7, 24.8; IR (neat): $\tilde{\nu}$ = 3074 (w), 3004 (w), 2976 (w), 2926 (w), 1640 (m), 1585 (s), 1562 (m), 1494 (m), 1469 (m), 1445 (m), 1429 (s), 1374 (w), 1326 (w), 1304 (w), 1239 (w), 1149 (w), 1117 (w), 1049 (w), 1033 (w), 989 (m), 864 (s), 763 (s), 751 (m), 699 (s) cm^{-1}; HRMS (CI(CH_4)) calcd for ($C_{21}H_{23}N$ + H): 290.1909; found: 290.1901.

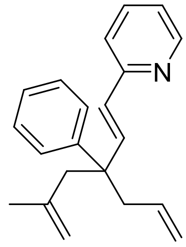

Compound 4d. Yellow oil (73 mg, 62%)^{1}H NMR (500 MHz, $CDCl_3$): δ = 8.55–8.51 (m, 1H), 7.59 (td, *J* = 7.7, 1.7 Hz, 1H), 7.35–7.26 (m, 5H), 7.21–7.15 (m, 1H), 7.09 (ddd, *J* = 7.4, 5.0, 0.8 Hz, 1H), 6.84 (d, *J* = 16.3 Hz, 1H), 6.52 (d, *J* = 16.5 Hz, 1H), 5.61 (ddt, *J* = 17.1, 10.1, 7.1 Hz, 1H), 5.06–4.94 (m, 3H), 2.66 (d, *J* = 7.2 Hz, 2H), 2.58 (d, *J* = 7.0 Hz, 2H), 1.61 (s, 3H), 1.50 (s, 3H); ^{13}C NMR (125 MHz, $CDCl_3$): δ = 156.2, 149.4, 145.0, 142.2, 136.4, 14.8, 133.7, 128.6, 128.0 (2C), 127.6 (2C), 126.0, 121.7, 120.9, 119.8, 117.8, 47.6, 42.2, 36.1, 26.0, 18.1; IR (neat): $\tilde{\nu}$ = 3058 (w), 2976 (w), 2913 (w), 2926 (w), 2855 (w), 1647 (e), 1585 (m), 1562 (m), 1494 (w), 1469 (m), 1445 (m), 1429 (m), 1376 (w), 1322 (w), 1305 (w), 1240 (w), 1149 (w), 1049 (w), 1033 (w), 989 (m), 979 (m), 912 (m), 854 (w), 761 (s), 742 (m), 699 (s) cm^{-1}; HRMS (ESI) calcd for ($C_{22}H_{26}N$ + H): 304.2065; found: 304.2067.

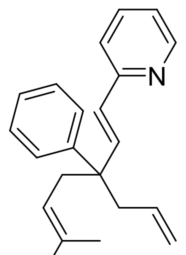

Preparation[10] of [Rh(coe)$_2$Cl]$_2$ – To a 3-neck round-bottomed flask containing $RhCl_3.3H_2O$ (175 mg, 0.84 mmol) were sequentially added degassed water (1 mL), degassed iPrOH (4 mL) and cyclooctene (0.56 mL, 4.30 mmol). The resulting dark red solution was heated under reflux for 2 hours. Afterwards, the resulting orange suspension was cooled to room temperature. The precipitate was collected by filtration, washed with cold ethanol, washed with petroleum ether and dried in vacuo to give [Rh(coe)$_2$Cl]$_2$ (165 mg, 28 %) as yellow solid. The purity of each batch of this pre-catalyst was assessed by elemental analysis (%) calcd for $C_{32}H_{50}Cl_2Rh_2$: C 53.57, H 7.87; found: C 52.23, H 7.62.

Representative procedure for the rhodium-catalysed carbocyclisation of 1,6-dienes – [Rh(coe)$_2$Cl]$_2$ (2.6 mg, 0.0036 mmol) and P(*p*MeOC$_6$H$_4$)$_3$ (5.1 mg, 0.0145 mmol) were added to a flame-dried J-Young Schlenk flask under N_2. THF (0.17 mL) was added and the red solution stirred at room temperature for 5 minutes. $AgBF_4$ (1.4 mg, 0.0073 mmol) in THF (0.1 mL) was added and stirred for 5 minutes. Then, under N_2, **1a** (20 mg, 0.0726 mmol) in THF (0.4 mL) was added via canula, the tube sealed and the reaction heated at 60 °C for 17 hours. The reaction mixture was cooled to room temperature, filtered through a small plug of silica (using CH_2Cl_2 to rinse) and the solvent was removed under reduced pressure. Purification by flash column chromatography (petroleum ether/Et_2O, 25:1 → 20:1) afforded **2a** as colourless oil (17.7 mg, 89%, **2a**/**3a** = 98:2).

Compound 2a. ^{1}H NMR (500 MHz, $CDCl_3$): δ = 8.48 (ddd, *J* = 4.8, 1.7, 0.8 Hz, 1H), 7.50 (dt, *J* = 7.8, 1.8 Hz, 1H), 7.36–7.31 (m, 4H), 7.25–7.21 (m, 1H), 7.10 (d, *J* = 8.1 Hz, 1H), 6.96 (ddd, *J* = 7.4, 4.9, 0.9 Hz, 1H), 5.75 (t, *J* = 2.3 Hz, 1H), 2.86 (dt, *J* = 17.2, 3.5 Hz, 1H), 2.67 (dt, *J* = 17.2, 2.5 Hz, 1H), 2.23 (d, *J* = 4.2 Hz, 1H), 2.19 (ddd, *J* = 11.6, 8.5, 2.5 Hz, 1H), 1.97 (ddt, *J* = 9.8, 2.1, 1.0 Hz, 1H), 1.93–1.84 (m, 1H), 1.82 (ddd, *J* = 9.7, 3.3, 1.7 Hz, 1H), 1.54 (dd, *J* = 11.8, 4.8 Hz, 1H), 1.02 (d, *J* = 7.0 Hz, 3H); ^{13}C NMR (125 MHz, $CDCl_3$): δ = 158.2, 157.6, 149.1, 142.8, 135.7, 128.19 (2C), 128.15 (2C), 126.3, 122.7, 120.1, 119.7, 59.6, 43.5, 42.6, 41.2, 40.9, 36.4, 22.3; IR (neat): $\tilde{\nu}$ = 3057 (w), 2951 (m), 2866 (w), 1655 (w), 1585 (s), 1559 (w), 1497 (w), 1471 (m), 1445 (w), 1427 (m), 1375 (w), 1346 (w), 1317 (w), 1260 (w), 1236 (w), 1220 (w), 1184 (w), 1148 (w), 1081 (w), 1061 (w), 1035 (w), 991 (w), 961 (w), 920 (w), 894 (w), 864 (w), 778 (w), 758 (m), 741 (m), 699 (s), 659 (w) cm^{-1}; HRMS (ESI): calcd for ($C_{20}H_{21}N$ + H): 276.1752; found: 276.1755.

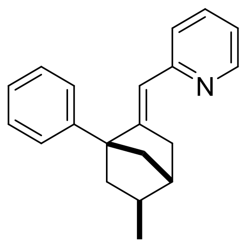

Compound 3a. This compound could be isolated by preparative TLC during the optimisation study. Colourless oil; ^{1}H NMR (500 MHz, $CDCl_3$): δ = 8.60 (ddd, *J* = 4.8, 1.7, 0.8 Hz, 1H), 7.61 (dt, *J* = 7.7, 1.8 Hz, 1H), 7.43–7.38 (m, 2H), 7.30–7.25 (m, 3H), 7.19–7.14 (m, 1H), 7.06 (ddd, *J* = 7.4, 4.9, 0.8 Hz, 1H), 6.44 (t, *J* = 2.5 Hz, 1H), 5.80–5.69 (m, 1H), 5.04 (ddt, *J* = 17.1, 1.9, 1.4 Hz, 1H), 5.00 (ddt, *J* = 10.2, 2.1, 1.0 Hz, 1H), 2.89–2.82 (m, 2H), 2.75 (ddt, *J* = 14.2, 7.4, 1.0 Hz, 1H), 2.64 (ddt, *J* = 14.2, 6.6, 1.3 Hz, 1H), 2.21 (ddd, *J* = 12.6, 6.5, 3.6 Hz, 1H), 1.87 (ddd, *J* = 12.7, 10.1, 7.1 Hz, 1H), 1.80–1.70 (m, 1H), 1.64–1.50 (m, 1H); ^{13}C NMR (125 MHz, $CDCl_3$): δ = 157.2, 156.1, 149.2, 146.1, 135.9, 135.6, 128.1 (2C), 127.1 (2C), 125.9, 123.5, 123.4, 120.5, 117.2, 55.8, 44.9, 37.7, 32.3, 22.1; HRMS (ESI): calcd for ($C_{20}H_{21}N$ + H): 276.1752; found: 276.1751.

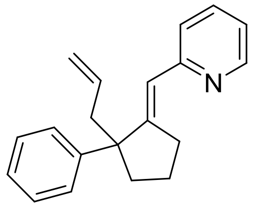

[10] Adapted from I. Ojima, A. T. Vu, D. Bonafoux, *Science of Synthesis* **2001**, Georg Thieme Verlag KG.

Compound 2a-D. Obtained from **1a-D** (0.071 mmol, 19.6 mg) following the representative procedure. Colourless oil (19.6 mg, quantitative); ^{1}H NMR (500 MHz, $CDCl_3$): δ = 8.48 (ddd, *J* = 4.8, 1.6, 0.7 Hz, 1H), 7.51 (dt, *J* = 7.8, 1.8 Hz, 1H), 7.37–7.31 (m, 4H), 7.26–7.21 (m, 1H), 7.09 (d, *J* = 8.0 Hz, 1H), 6.96 (ddd, *J* = 7.4, 4.8, 0.9 Hz, 1H), 5.77–5.74 (m, 1H), 2.86 (dt, *J* = 17.3, 3.4 Hz, 0.8H), 2.67 (dt, *J* = 17.3, 2.5 Hz, 0.8H), 2.25–2.21 (m, 1H), 2.18 (ddd, *J* = 11.6, 8.5, 2.5 Hz, 1H), 1.99–1.94 (m, 1H), 1.93–1.84 (m, 1H), 1.82 (ddd, *J* = 9.7, 3.1, 1.5 Hz, 1H), 1.54 (dd, *J* = 11.8, 4.9 Hz, 1H), 1.05–0.98 (m, 2.4H); ^{2}D NMR (77 MHz, $CDCl_3$): δ = 2.86 (s, 0.2D), 2.78 (s, 0.2D), 1.04 (s, 0.6D); ^{13}C NMR (125 MHz, $CDCl_3$):[11] δ = 158.20, [158.16], 157.6, 149.1, 142.7, 135.7, 128.17 (2C), 128.14 (2C), 126.3, 122.6, 120.1, [119.71], 119.66, 59.6, [43.47], 43.43, 42.51, [42.45], 41.2, 40.9, [40.5 (t, *J* = 20.3 Hz)], [36.32], 36.26, [22.3], 22.0 (t, *J* = 19.2 Hz); IR (neat): $\tilde{\nu}$ = 3057 (w), 3002 (w), 2949 (s), 2866 (w), 2165 (w), 1655 (m), 1602 (w), 1585 (s), 1559 (w), 1497 (w), 1471 (m), 1446 (w), 1427 (s), 1375 (w), 1318 (w), 1257 (w), 1234 (w), 1220 (w), 1148 (w), 1086 (w), 1060 (w), 1036 (w), 990 (w), 958 (w), 890 (w), 864 (w), 776 (w), 758 (s), 741 (m), 699 (s) cm^{-1}; MS (CI(CH_4)): *m/z* (rel. intensity): 277 (70) [M + H], 169 (100); HRMS (CI(CH_4)): calcd for ($C_{20}H_{20}DN$ + H): 277.1815; found: 277.1805.

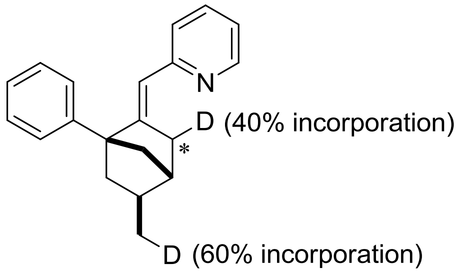

Hydrochloride salt of compound 2a. Compound **2a** (20 mg, 0.073 mmol) was dissolved in Et_2O (0.2 mL). Then 3 drops of conc. HCl solution were added to give a white suspension. After mixing vigorously for 5 minutes, all volatiles were removed. The residue was dissolved in a vial with CH_2Cl_2 (1 mL) and then petroleum ether (5 mL) so that the two layers do not mix. All volatiles evaporated slowly at room temperature and atmospheric pressure to give brown crystals which were washed with diethyl ether to give brown crystals which were suitable for X-ray crystalography (15 mg, 66 %). mp.: 167–174°C (decomposition); ^{1}H NMR (500 MHz, $CDCl_3$): δ = 8.71–8.54 (m, 1H), 8.19–8.06 (m, 1H), 7.67–7.56 (m, 1H), 7.53–7.44 (m, 1H), 7.41 (t, *J* = 7.4 Hz, 2H), 7.34 (d, *J* = 7.6 Hz, 2H), 7.30 (t, *J* = 7.4 Hz, 3H), 6.37–6.29 (m, 1H), 2.94 (d, *J* = 16.1 Hz, 1H), 2.66 (d, *J* = 16.1 Hz, 1H), 2.34 (s, 1H), 2.28–2.19 (m, 1H), 2.06 (d, *J* = 9.7 Hz, 1H), 1.97–1.86 (m, 2H), 1.61 (dd, *J* = 12.0, 4.5 Hz, 1H), 1.05 (d, *J* = 6.8 Hz, 3H); ^{13}C NMR (125 MHz, $CDCl_3$): δ = 170.3, 151.6, 144.2, 141.5 (br), 140.1, 128.7 (2C), 128.0 (2C), 127.3, 124.8, 122.7, 111.9, 61.0, 43.3, 42.2, 41.3, 41.1, 35.9, 22.0; IR (neat): $\tilde{\nu}$ = 3086 (w), 3040 (w), 2958 (w), 2916 (w), 2862 (w), 2266 (m), 2209 (m), 2041 (m), 1980 (m), 1933 (m), 1650 (m), 1608 (s), 1528 (m), 1496 (m), 1456 (m), 1444 (m), 1399 (w), 1371 (m), 1323 (w), 1290 (m), 1251 (w), 1233 (w), 1155 (m), 1141 (w), 1096 (w), 1064 (w), 1038 (w), 1025 (w), 988 (m), 961 (w), 950 (w), 923 (w), 896 (w), 882 (w), 871 (w), 847 (w), 827 (w), 814 (w), 772 (s), 763 (s), 702 (s), 659 (w) cm^{-1}; elemental analysis (%) calcd for $C_{20}H_{22}ClN$: C 77.03, H 7.11, N 4.49; found: C 76.49, H 7.06, N 4.41.

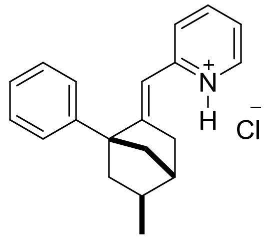

[11] As easily established by comparison of ^{13}C NMR APT, HSQC and HMBC NMR of **2a** and **2a-D**, the chemical shifts in brackets correspond to the mono-deuterated isomer for which the deuterium atom is incorporated at the position indicated by a star.

Compound 2b. Obtained from **1b** (0.067 mmol, 22 mg) following the representative procedure. Colourless oil (16 mg, 73%, **2b**/**3b** = 97:3); ^{1}H NMR (500 MHz, $CDCl_3$): δ = 8.49–8.46 (m, 1H), 7.87–7.77 (m, 4H), 7.53–7.43 (m, 4H), 7.04 (d, *J* = 8.2 Hz, 1H), 6.94 (dd, *J* = 7.4, 4.9 Hz, 1H), 5.74 (t, *J* = 2.4 Hz, 1H), 2.92 (dt, *J* = 17.1, 3.3 Hz, 1H), 2.74 (dt, *J* = 17.4, 2.5 Hz, 1H), 2.35–2.28 (m, 2H), 2.08 (d, J = 9.6 Hz, 1H), 1.95 (hex, *J* = 6.8 Hz, 1H), 1.89 (ddd, *J* = 9.6, 3.1, 1.5 Hz, 1H), 1.68 (dd, *J* = 11.5, 4.8 Hz, 1H), 1.07 (d, *J* = 7.0 Hz, 3H); ^{13}C NMR (100 MHz, $CDCl_3$): δ = 158.3, 157.5, 149.1, 140.4, 135.7, 133.4, 132.3, 127.8, 127.6, 127.5, 127.0, 126.5, 125.8, 125.4, 122.7, 120.1, 119.9, 59.8, 43.4, 42.8, 41.6, 41.0, 36.4, 22.3; IR (neat): $\tilde{\nu}$ = 3055 (w), 2951 (s), 2865 (w), 1655 (m), 1600 (w), 1585 (s), 1558 (w), 1506 (w), 1470 (s), 1428 (s), 1375 (w), 1309 (w), 1260 (w), 1220 (w), 1198 (w), 1148 (w), 1128 (w), 1089 (w), 1018 (w), 992 (w), 908 (s), 891 (m), 854 (m), 816 (s), 778 (m), 734 (s) cm^{-1}; MS (ESI): *m*/*z* (rel. intensity): 326 (100) [M + H], 284 (22); HRMS (ESI) calcd for ($C_{24}H_{23}N$ + H): 326.1909; found: 326.1903; elemental analysis (%) calcd for $C_{24}H_{23}N$: C 88.57, H 7.12, N 4.30; found: C 88.35, H 7.33, N 4.17.

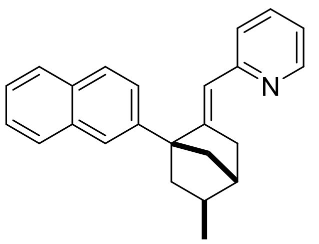

Compound 2c. Obtained from **1c** (0.126 mmol, 39 mg) following the representative procedure. Colourless oil (38 mg, 97%, **2c**/**3c** = 96:4); ^{1}H NMR (400 MHz, $CDCl_3$): δ = 8.51–8.47 (m, 1H), 7.52 (td, *J* = 7.8, 1.7 Hz, 1H), 7.31 (d, *J* = 8.8 Hz, 2H), 7.25 (d, *J* = 8.8 Hz, 2H), 7.10 (d, *J* = 7.9 Hz, 1H), 6.97 (ddd, *J* = 7.3, 5.0, 0.8 Hz, 1H), 5.71 (t, *J* = 1.5 Hz, 1H), 2.85 (dt, *J* = 17.2, 3.3 Hz, 1H), 2.67 (dt, *J* = 17.4, 2.6 Hz, 1H), 2.23 (d, *J* = 4.0 Hz, 1H), 2.15 (ddd, *J* = 11.6, 8.3, 2.3 Hz, 1H), 1.92 (d, *J* = 10.0 Hz, 1H), 1.91–1.85 (m, 1H), 1.84 (ddd, *J* = 9.7, 3.0, 1.5 Hz, 1H), 1.49 (dd, *J* = 11.6, 4.8 Hz, 1H), 1.02 (d, *J* = 7.0 Hz, 3H); ^{13}C NMR (100 MHz, $CDCl_3$): δ = 157.7, 157.3, 149.1, 141.3, 135.8, 132.0, 129.6 (2C), 128.2 (2C), 122.6, 120.3, 119.8, 59.1, 43.4, 42.5, 41.3, 40.8, 36.3, 22.3; IR (neat): $\tilde{\nu}$ = 3051 (w), 2951 (s), 2866 (w), 1655 (m), 1585 (s), 1559 (m), 1493 (s), 1470 (s), 1427 (s), 1399 (w), 1375 (w), 1346 (w), 1316 (w), 1299 (w), 1259 (w), 1237 (w), 1189 (w), 1148 (w), 1093 (s), 1062 (w), 1014 (s), 1000 (w), 990 (m), 961 (w), 908 (m), 894 (w), 875 (m), 864 (m), 835 (s), 813 (s), 777 (s), 717 (s) cm^{-1}; MS (ESI+): *m*/*z* (rel. intensity): 334 (5) [$C_{20}H_{21}N^{37}Cl$ + Na], 332 (15) [$C_{20}H_{21}N^{35}Cl$ + Na], 312 (34) [$C_{20}H_{21}N^{37}Cl$ + H], 310 (100) [$C_{20}H_{21}N^{35}Cl$ + H]; HRMS (ESI) calcd for ($C_{20}H_{21}N^{37}Cl$ + H): 312.1333; found: 312.1321; calcd for ($C_{20}H_{21}N^{35}Cl$ + H): 310.1363; found: 310.1359.

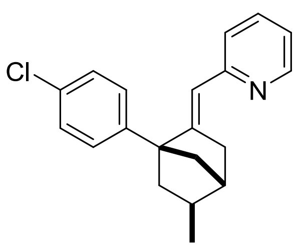

Compound 2d. Obtained from **1d** (0.116 mmol, 34 mg) following the representative procedure. Colourless oil (28 mg, 82%, **2d**/**3d** = 96:4); ^{1}H NMR (500 MHz, $CDCl_3$): δ = 8.51–8.46 (m, 1H), 7.52 (td, *J* = 7.7 and 1.9 Hz, 1H), 7.28 (dd, *J* = 8.8, 5.5 Hz, 2H), 7.10 (d, *J* = 8.0 Hz, 1H), 7.04 (t, *J* = 8.8 Hz, 2H), 6.97 (ddd, *J* = 7.4, 4.9, 0.9 Hz, 1H), 5.71 (t, *J* = 2.2 Hz, 1H), 2.85 (dt, *J* = 17.2, 3.4 Hz, 1H), 2.67 (dt, *J* = 17.3, 2.5 Hz, 1H), 2.23 (d, *J* = 4.1 Hz, 1H), 2.16 (ddd, *J* = 10.9, 8.4, 2.4 Hz, 1H), 1.95–1.89 (m, 1H), 1.92–1.84 (m, 1H), 1.80 (ddd, *J* = 9.7, 3.2, 1.6 Hz, 1H), 1.50 (dd, *J* = 11.7, 4.9 Hz, 1H), 1.03 (d, *J* = 7.0 Hz, 3H); ^{13}C NMR (125 MHz, $CDCl_3$): δ = 161.5 (d, *J* = 242.8 Hz), 158.0, 157.4, 149.0, 138.4 (d, *J* = 2.7 Hz), 135.8, 129.6 (d, *J* = 7.9 Hz, 2C), 122.6, 120.3, 119.7, 114.9 (d, J_F = 20.9 Hz, 2C), 59.0, 43.6, 42.6, 41.4, 40.8, 36.4, 22.3; IR (neat): $\tilde{\nu}$ = 3046 (w), 2951 (m), 2867 (w), 1655 (w), 1585 (m), 1559 (w), 1510 (s), 1470 (m), 1428 (m), 1375 (w), 1346 (w), 1301 (w), 1259 (w), 1221 (m), 1159 (m), 1149 (m), 1089 (w), 1061 (w), 1014 (w), 1001 (w), 991 (w), 961 (w), 913 (w), 895 (w), 876 (w), 864 (w), 837 (m), 820 (m), 811 (m), 777 (m), 757 (w), 739 (m) cm^{-1}; MS ($CI(CH_4)$): *m*/*z* (rel. intensity): 322 (25) [M + C_2H_6], 282 (100) [M + H], 274 (24); HRMS ($CI(CH_4)$) calcd for ($C_{20}H_{21}FN$ + H): 294.1653; found: 294.1664; elemental analysis (%) calcd for $C_{20}H_{20}N$: C 81.88, H 6.87, N 4.77; found: C 81.75, H 7.05, N 4.63.

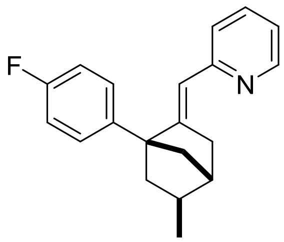

Compound 2e. Obtained from **1d** (0.120 mmol, 37 mg) following the representative procedure. Colourless oil (28 mg, 76%, **2e**/**3e** = 95:5); ^{1}H NMR (500 MHz, $CDCl_3$): δ = 8.48 (dd, *J* = 4.7, 1.0, 0.8 Hz, 1H), 7.51 (t, *J* = 7.7 Hz, 1H), 7.25 (d, *J* = 8.8 Hz, 2H), 7.11 (d, *J* = 8.0 Hz, 1H), 6.96 (dd, *J* = 7.4, 4.7 Hz, 1H), 6.89 (d, *J* = 8.8 Hz, 2H), 5.79–5.73 (m, 1H), 3.81 (s, 3H), 2.84 (dt, *J* = 17.1, 3.5 Hz, 1H), 2.68–2.61 (m, 1H), 2.21 (d, *J* = 4.2 Hz, 1H), 2.17–2.12 (m, 1H), 1.95–1.90 (m, 1H), 1.88 (hex, *J* = 6.6 Hz, 1H), 1.81–1.74 (m, 1H), 1.55 (dd, *J* = 11.7, 4.8 Hz, 1H), 1.02 (d, *J* = 6.9 Hz, 3H); ^{13}C NMR (125 MHz, $CDCl_3$): δ = 158.5, 158.0, 157.6, 149.1, 135.7, 134.8, 129.1 (2C), 122.6, 120.1, 119.6, 113.6 (2C), 59.0, 55.2, 43.6, 42.5, 41.4, 40.8, 36.4, 22.3; IR (neat): $\tilde{\nu}$ = 3038 (w), 2999 (w), 2949 (s), 2865 (m), 2834 (w), 1655 (m), 1612 (m), 1585 (s), 1558 (w), 1513 (s), 1463 (s), 1442 (m), 1427 (s), 1374 (w), 1345 (w), 1318 (w), 1289 (m), 1245 (s), 1178 (s), 1148 (w), 1118 (w), 1091 (w), 1061 (w), 1038 (s), 990 (w), 960 (w), 914 (w), 894 (w), 875 (w), 864 (w), 835 (s), 819 (m), 794 (w), 777 (m), 757 (w), 741 (m) cm^{-1}; MS (ESI): *m*/*z* (rel. intensity): 306 (100) [M + H]; HRMS (ESI) calcd for ($C_{21}H_{23}NO$ + H): 306.1858; found: 306.1859.

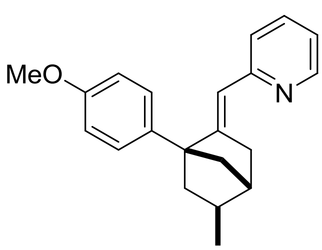

Compound 2f. Obtained from **1e** (0.098 mmol, 33 mg) following the representative procedure. Colourless oil (26 mg, 79%, **2f**/**3f** = 96:4); ^{1}H NMR (500 MHz, $CDCl_3$): δ = 8.49 (d, *J* = 4.7, 1.0 Hz, 1H), 7.54 (td, *J* = 7.8, 1.3 Hz, 1H), 7.12 (d, *J* = 7.9 Hz, 1H), 6.98 (dd, *J* = 6.8, 5.3 Hz, 1H), 6.90–6.82 (m, 3H), 5.83–5.79 (m, 1H), 3.87 (s, 3H), 3.86 (s, 3H), 2.85 (dt, *J* = 17.3, 3.5 Hz, 1H), 2.86 (dt, *J* = 17.3, 2.2 Hz, 1H), 2.22 (d, *J* = 4.1 Hz, 1H), 2.15 (ddd, *J* = 11.3, 8.7, 2.5 Hz, 1H), 1.92 (d, *J* = 9.6, 1H), 1.88 (hex, *J* = 6.9 Hz, 1H), 1.81 (ddd, *J* = 9.6, 2.9, 1.4 Hz, 1H), 1.53 (dd, *J* = 11.5, 4.8 Hz, 1H), 1.02 (d, *J* = 7.0 Hz, 3H); ^{13}C NMR (125 MHz, $CDCl_3$): δ = 158.5, 157.3, 148.8, 148.5, 147.4, 135.8, 135.3, 122.6, 120.2, 120.1, 119.2, 111.5, 110.9, 59.2, 55.8, 43.5, 42.5, 41.4, 40.8, 36.3, 22.2; IR (neat): $\tilde{\nu}$ = 2951 (s), 2867 (w), 2833 (w), 1655 (m), 1585 (s), 1559 (w), 1516 (s), 1463 (s), 1427 (s), 1410 (s), 1374 (w), 1362 (w), 1346 (w), 1322 (w), 1251 (s), 1238 (s), 1215 (s), 1170 (s), 1144 (s), 1110 (w), 1028 (s), 974 (w), 914 (w), 894 (w), 851 (w), 802 (m), 778 (m), 764 (s), 732 (s) cm^{-1}; MS ($CI(CH_4)$): *m*/*z* (rel. intensity): 364 (10) [M + C_2H_6], 336 (100) [M + H]; HRMS ($CI(CH_4)$) calcd for ($C_{22}H_{25}NO_2$ + H): 336.1958; found: 336.1957.

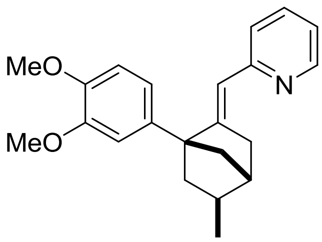

Compound 2g. Obtained from **1g** (0.126 mmol, 40 mg) following the representative procedure. Crystalline needles (33 mg, 84%, **2g**/**3g** = 85:15); ^{1}H NMR (500 MHz, $CDCl_3$): δ = 8.48 (ddd, *J* = 4.7, 1.9, 0.9 Hz, 1H), 7.84-7.46 (m, 4H), 7.25 (ddd, *J* = 8.5, 7.0, 1.2 Hz, 1H), 7.15 (ddd, *J* = 8.0, 7.0, 0.8 Hz, 1H), 7.05 (d, *J* = 8.0 Hz, 1H), 6.96 (ddd, *J* = 7.5, 4.8, 1.0 Hz, 1H), 5.95 (t, *J* = 2.3 Hz, 1H), 2.94 (dt, *J* = 17.4, 3.6 Hz, 1H), 2.75 (dt, *J* = 17.4, 2.8 Hz, 1H), 2.29 (d, *J* = 4.0Hz, 1H), 2.16 (ddd, J = 11.2, 8.3, 2.4 Hz, 1H), 2.14–2.10 (m, 1H), 1.95–1.87 (m, 1H), 1.74 (ddd, *J* = 9.7, 3.3, 1.6 Hz, 1H), 1.61 (dd, *J* = 11.6, 4.9 Hz, 1H), 1.05 (d, *J* = 6.9 Hz, 3H); ^{13}C NMR (125 MHz, $CDCl_3$): δ = 157.4, 155.8, 154.3, 149.1, 142.1, 135.7, 127.4, 123.9, 122.8, 122.13, 122.08, 121.8, 120.2, 119.2, 111.5, 52.9, 43.5, 43.0, 41.4, 40.8, 35.6, 22.3; IR (neat): $\tilde{\nu}$ = 3056 (w), 2951 (m), 2866 (w), 1661 (m), 1584 (m), 1559 (m), 1470 (m), 1451 (s), 1427 (m), 1375 (w), 1340 (w), 1309 (w), 1295 (w), 1272 (w), 1261 (w), 1247 (w), 1215 (m), 1187 (w), 1148 (w), 1100 (s), 1088 (m), 1071 (w), 1011 (w), 988 (w), 964 (w), 930 (w), 908 (m), 894 (w), 857 (m), 827 (w), 777 (m), 768 (m), 741 (s) cm^{-1}; MS (ESI): *m*/*z* (rel. intensity): 338 (12) [M + Na], 316 (100) [M + Na]; HRMS (ESI): calcd for ($C_{22}H_{21}NO$ + H): 316.1701; found: 316.1693.

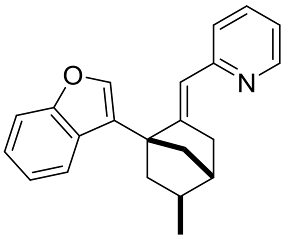

Compound 2h. Obtained from **1h** (0.070 mmol, 20 mg) following the representative procedure. Colourless oil (18 mg, 88%); ^{1}H NMR (500 MHz, $CDCl_3$): δ = 8.54 (ddd, *J* = 4.8, 1.8, 0.8 Hz, 1H), 7.55 (dt, *J* = 7.7, 1.9 Hz, 1H), 7.22 (d, *J* = 8.1 Hz, 1H), 6.98 (ddd, *J* = 7.4, 4.9, 1.0 Hz, 1H), 6.79 (t, *J* = 2.3 Hz, 1H), 4.07–3.88 (m, 4H), 2.71 (dt, *J* = 17.1, 3.4 Hz, 1H), 2.61 (dt, *J* = 17.1, 2.5 Hz, 1H), 2.04 (d, *J* = 3.9 Hz, 1H), 1.74 (ddd, *J* = 9.6, 3.4, 1.8 Hz, 1H), 1.73–1.67 (m, 1H), 1.62 (ddd, *J* = 11.4, 8.4, 2.4 Hz, 1H), 1.45 (s, 3H), 1.44–1.38 (m, 2H), 0.97 (d, *J* = 6.9 Hz, 3H); ^{13}C NMR (125 MHz, $CDCl_3$): δ = 158.1, 152.8, 149.0, 135.7, 123.1, 120.2, 120.0, 110.9, 65.2, 64.5, 63.0, 42.3, 42.0, 38.9, 38.6, 35.8, 22.2, 21.7; IR (neat): $\tilde{\nu}$ = 3052 (w), 2950 (s), 2871 (m), 1650 (m), 1585 (s), 1558 (w), 1470 (m), 1428 (s), 1371 (m), 1321 (w), 1287 (w), 1264 (m), 1239 (w), 1216 (w), 1198 (s), 1161 (s), 1111 (m), 1090 (m), 1075 (w), 1063 (m), 1040 (s), 992 (w), 943 (w), 920 (w), 899 (m), 870 (m), 812 (w), 779 (m), 761 (w), 741 (m), 658 (w) cm^{-1}; HRMS (CI(CH_4)): calcd for ($C_{18}H_{23}NO_2$ + H): 286.1802; found: 286.1000.

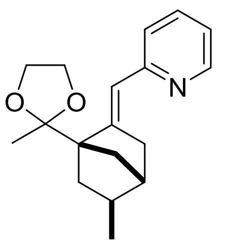

Compound 2i. Obtained from **1i** (0.146 mmol, 56 mg) following the representative procedure. Colourless gum (43 mg, 76%);[12] ^{1}H NMR (500 MHz, $CDCl_3$): δ = 8.54 (dd, *J* = 4.8 and 1.0 Hz, 1H), 7.73 (d, *J* = 8.1 Hz, 2H), 7.55 (td, *J* = 7.8, 1.8 Hz, 1H), 7.26 (d, *J* = 8.1 Hz, 2H), 7.13 (d, *J* = 7.9 Hz, 1H), 7.02 (ddd, *J* = 7.3, 4.8, 0.6 Hz, 1H), 6.38 (t, *J* = 2.2 Hz, 1H), 3.11 (s, 3H), 2.77–2.62 (m, 2H), 2.39 (s, 3H), 2.19 (ddd, *J* = 11.2, 8.4, 2.4 Hz, 1H), 1.97 (d, *J* = 2.6 Hz, 1H), 1.86 (d, *J* = 9.5 Hz, 1H), 1.81–1.72 (m, 1H), 1.58 (dd, *J* = 8.8, 5.0 Hz, 1H), 1.57–1.53 (m, 1H), 0.88 (d, *J* = 6.9 Hz, 3H); ^{13}C NMR (125 MHz, $CDCl_3$): δ = 159.9, 149.7, 149.0, 142.8, 139.5, 135.9, 129.5 (2C), 126.8 (2C), 123.6, 120.4, 118.4, 74.9, 43.2, 39.81, 39.76, 38.9, 35.7, 35.4, 22.1, 21.4; IR (neat): $\tilde{\nu}$ = 2954 (w), 2923 (w), 2868 (w), 1666 (w), 1585 (m), 1560 (w), 1494 (w), 1470 (m), 1428 (m), 1377 (w), 1336 (s), 1306 (s), 1280 (w), 1225 (w), 1151 (s), 1119 (w), 1089 (s), 1042 (s), 1016 (w), 982 (w), 902 (m), 842 (s), 812 (s), 778 (m), 759 (w), 741 (m), 706 (w), 661 (s) cm^{-1}; HRMS (ESI): calcd for ($C_{22}H_{27}N_2O_2S$ + H): 383.1793; found: 383.1783.

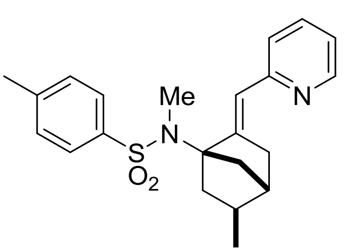

Compound 2j. Obtained from **1j** (0.071 mmol, 20 mg) following the representative procedure. Colourless oil (15 mg, 75%, **2j**/**3k** = 96:4); ^{1}H NMR (500 MHz, $CDCl_3$): δ = 8.52 (ddd, *J* = 4.7, 1.6, 0.7 Hz, 1H), 7.55 (td, *J* = 7.8, 1.8 Hz, 1H), 7.25 (d, *J* = 8.0 Hz, 1H), 6.98 (ddd, *J* = 7.2, 4.7, 0.7 Hz, 1H), 6.32–6.28 (m, 1H), 2.58 (dt, *J* = 16.8, 3.2 Hz, 1H), 2.52 (dt, *J* = 16.8, 2.2 Hz, 1H), 2.01 (d, *J* = 3.2 Hz, 1H), 1.99–1.89 (m, 2H), 1.85–1.78 (m, 2H), 1.85–1.69 (m, 4H), 1.45 (ddd, *J* = 9.5, 2.8, 1.5 Hz, 1H), 1.40–1.24 (m, 4H), 1.23–1.18 (m, 1H), 1.18–1.12 (m, 1H), 1.12–1.03 (m, 1H), 1.05–0.93 (m, 1H), 0.93 (d, *J* = 7.0 Hz, 3H); ^{13}C NMR (125 MHz, $CDCl_3$): δ = 158.0, 156.2, 149.1, 135.7, 122.4, 120.0, 118.1, 58.6, 42.03, 41.95, 41.1, 37.7, 36.1, 35.6, 29.7, 29.2, 27.11, 27.09, 26.8, 22.3; IR (neat): $\tilde{\nu}$ = 2923 (s), 2851 (s), 1650 (m), 1585 (s), 1558 (w), 1470 (s), 1462 (s), 1449 (m), 1426 (s), 1373 (w), 1347 (w), 1308 (w), 1268 (w), 1221 (w), 1147 (w), 1105 (w), 1089 (w), 1061 (w), 1020 (w), 988 (w), 893 (w), 858 (w), 775 (m), 762 (m), 739 (s) cm^{-1}; HRMS (CI(CH_4)): calcd for ($C_{20}H_{27}N$ + H): 282.2216; found: 282.2212.

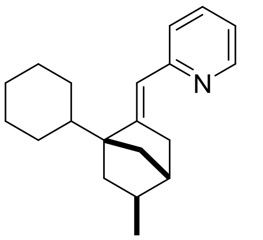

---

[12] Over time, we observed a small doublet appearing at 2.64 ppm (*J* = 5.4 Hz) in ^{1}H NMR which could correspond to the *Me*-N of a minor impurity where the tosyl group is cleaved. A small peak (ESI) with the expected *m*/*z* = 228 [M – Ts + H] also confirms this analysis.

Compound 2k. Obtained from **1k** (0.070 mmol, 20.3 mg) following the representative procedure, except that P(*p*MeOC_6H_4)$_3$ (3.6 mg, 0.0101 mmol) was used. Colourless oil. (17 mg, 83%, **2k**/**3k** = 95:5); ^{1}H NMR (500 MHz, $CDCl_3$): δ = 8.55 (ddd, *J* = 4.9, 1.7, 0.8 Hz, 1H), 7.59 (dt, *J* = 7.7, 1.9 Hz, 1H), 7.29–7.24 (m, 3H), 7.21–7.16 (m, 3H), 7.02 (ddd, *J* = 7.4, 4.9, 1.0 Hz, 1H), 6.44–6.40 (m, 1H), 3.05 (d, *J* = 13.4 Hz, 1H), 2.99 (d, *J* = 13.4 Hz, 1H), 2.67 (dt, *J* = 17.1, 3.4 Hz, 1H), 2.57 (dt, *J* = 16.9, 2.5 Hz, 1H), 1.98 (d, *J* = 4.1 Hz, 1H), 1.72–1.62 (m, 1H), 1.50 (ddd, *J* = 11.7, 8.5, 2.5 Hz, 1H), 1.33 (ddd, *J* = 9.8, 3.1, 1.7 Hz, 1H), 1.26–1.21 (m, 1H), 1.06 (dd, *J* = 11.8, 4.9 Hz, 1H), 0.77 (d, *J* = 6.9 Hz, 3H); ^{13}C NMR (125 MHz, $CDCl_3$): δ = 157.7, 156.9, 149.2, 139.7, 135.8, 130.3 (2C), 127.8 (2C), 125.8, 122.5, 120.2, 117.7, 55.3, 42.7, 41.8, 41.7, 39.3, 38.2, 36.0, 22.0; IR (neat): $\tilde{\nu}$ = 3061 (w), 3027 (w), 3002 (w), 2950 (m), 2866 (w), 1655 (m), 1603 (w), 1585 (s), 1559 (w), 1496 (w), 1471 (m), 1454 (w), 1428 (m), 1375 (w), 1346 (w), 1308 (w), 1276 (w), 1221 (w), 1149 (w), 1096 (w), 1062 (w), 1031 (w), 991 (w), 889 (w), 858 (w), 804 (w), 771 (w), 754 (m), 741 (w), 702 (s), 658 (w) cm^{-1}; HRMS (ESI): calcd for ($C_{21}H_{24}N$ + H): 290.1911; found: 290.1909; elemental analysis (%) calcd for $C_{21}H_{23}N$: C 87.15, H 8.01, N 4.84; found: C 86.55, H 8.13, N 4.63.

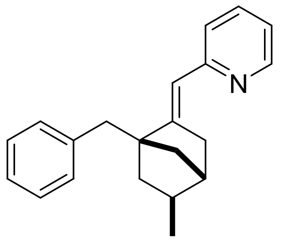

Compound 2l. Obtained from **1l** (0.070 mmol, 22.4 mg) following the representative procedure (15.9 mg, 71%, **2l**/**3l** = 2:1). Isomers **2l** and **3l** could be separated by preparative TLC (petroleum ether/EtOAc = 14:1). Colourless oil; ^{1}H NMR (500 MHz, $CDCl_3$): δ = 8.54–8.49 (m, 1H), 7.56 (dt, *J* = 7.7, 1.8 Hz, 1H), 7.38–7.30 (m, 4H), 7.29–7.24 (m, 1H), 7.21 (d, *J* = 8.0 Hz, 1H), 6.99 (ddd, *J* = 7.4, 4.9, 0.7 Hz, 1H), 6.25–6.23 (m, 1H), 4.60 (s, 2H), 3.77 (d, *J* = 9.6 Hz, 1H), 3.73 (d, *J* = 9.6 Hz, 1H), 2.68 (dt, *J* = 17.1, 3.5 Hz, 1H), 2.53 (dt, *J* = 17.1, 2.5 Hz, 1H), 2.08 (d, *J* = 4.1Hz, 1H), 1.77–1.68 (m, 1H), 1.64 (ddd, *J* = 9.7, 3.2, 1.6 Hz, 1H), 1.56 (ddd, *J* = 11.9, 8.5, 2.4 Hz, 1H), 1.41–1.36 (m, 1H), 1.26 (dd, *J* = 11.9, 4.9 Hz, 1H), 0.98 (d, *J* = 7.0 Hz, 3H); ^{13}C NMR (125 MHz, $CDCl_3$): δ = 157.6, 154.1, 149.1, 138.8, 135.8, 128.3 (2C), 127.5 (2C), 127.4, 122.5, 120.2, 117.7, 73.4, 71.2, 55.3, 42.5, 41.4, 41.2, 38.8, 36.0, 22.2; IR (neat): $\tilde{\nu}$ = 3058 (w), 303 (w), 3002 (w), 2950 (s), 2864 (m), 1720 (w), 1658 (m), 1585 (s), 1559 (w), 1495 (w), 1470 (m), 1454 (m), 1428 (s), 1364 (w), 1271 (w), 1205 (w), 1148 (w), 1093 (s), 1028 (w), 927 (w), 889 (w), 857 (w), 739 (s), 697 (m) ($C_{22}H_{25}NO$ + H): 320.2014; found: 320.2013.

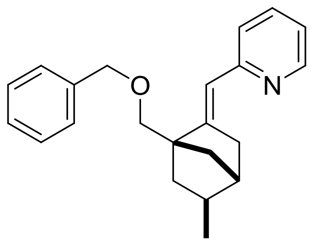

Compound 3l. Colourless oil; ^{1}H NMR (500 MHz, $CDCl_3$): δ = 8.55 (ddd, *J* = 4.7, 1.6, 0.8 Hz, 1H), 7.58 (dt, *J* = 7.8, 1.8 Hz, 1H), 7.33–7.28 (m, 4H), 7.27–7.20 (m, 2H), 7.02 (ddd, *J* = 7.5, 4.8, 1.0 Hz, 1H), 6.37 (t, *J* = 2.4 Hz, 1H), 5.85 – 5.73 (m, 1H), 5.08 – 4.97 (m, 2H), 4.53 (d, *J* = 12.4 Hz, 1H), 4.49 (d, *J* = 12.4 Hz, 1H), 3.36 (s, 2H), 2.90–2.81 (m, 1H), 2.80–2.70 (m, 1H), 2.47 (dd, *J* = 13.8, 8.0 Hz, 1H), 2.35 (dd, *J* = 13.8, 6.6 Hz, 1H), 1.84–1.62 (m, 4H); ^{13}C NMR (125 MHz, $CDCl_3$): δ = 157.5, 155.2, 149.1, 138.8, 135.8, 135.4, 128.3 (2C), 127.4 (2C), 127.3, 123.2, 122.0, 120.4, 117.3, 76.4, 73.3, 51.5, 41.5, 33.5, 33.4, 23.5; IR (neat): $\tilde{\nu}$ = 3065 (w), 2020 (w), 3004 (w), 2951 (w), 2856 (w), 1647 (w), 1584 (m), 1559 (w), 1496 (w), 1471 (m), 1454 (w), 1427 (m), 1360 (w), 1287 (w), 1218 (w), 1204 (w), 1148 (w), 1094 (s), 1028 (w), 991 (w), 911 (m), 890 (w), 864 (w), 818 (w), 775 (w), 736 (s), 696 (s) cm^{-1}; elemental analysis (%) calcd for $C_{22}H_{25}NO$: C 82.72, H 7.89, N 4.38; found: C 82.64, H 7.99, N 4.12.

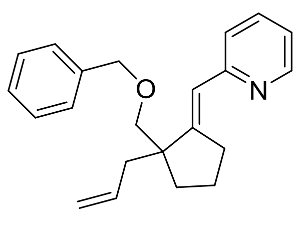

Compound 2m. Obtained from **1m** (0.080 mmol, 17.1 mg) following the representative procedure, except that P(*p*MeOC_6H_4)$_3$ (3.6 mg, 0.0101 mmol) was used. Colourless oil (12.3 mg, 72%, **2m**/**3m** = 3:1, not separable);[13] ^{1}H NMR (500 MHz, $CDCl_3$): δ = 8.53–8.50 (m, 1H), 7.56 (dt, *J* = 7.8, 1.8 Hz, 1H), 7.27–7.22 (m, 1H), 6.98 (ddd, *J* = 7.4, 4.9, 0.9 Hz, 1H), 6.27–6.24 (m, 1H), 2.64 (dt, *J* = 17.1, 3.5 Hz, 1H), 2.50 (dt, *J* = 17.1, 2.5 Hz, 1H), 2.04 (d, *J* = 4.2 Hz, 1H), 1.76–1.62 (m, 1H), 1.57 (ddd, *J* = 11.8, 8.5, 2.5, 1H), 1.52 (ddd, *J* = 9.6, 3.1, 1.5 Hz, 1H), 1.31 (s, 3H), 1.27–1.22 (m, 1H), 1.04 (dd, *J* = 11.9, 4.9 Hz, 1H), 0.96 (d, *J* = 7.1 Hz, 3H); ^{13}C NMR (125 MHz, $CDCl_3$): δ = 157.8, 157.5, 149.1, 135.8, 122.3, 120.0, 117.0, 51.0, 46.0, 42.7, 42.6, 41.0, 36.8, 22.3, 18.1; IR (neat): $\tilde{\nu}$ = 3073 (w), 3003 (w), 2950 (s), 2868 (w), 1731 (w), 1657 (m), 1585 (s), 1559 (w), 1471 (m), 1463 (m), 1427 (s), 1375 (w), 1316 (w), 1279 (w), 1265 (w), 1227 (w), 1149 (w), 1088 (w), 1061 (w), 989 (w), 966 (w), 912 (w), 887 (w), 858 (w), 823 (w), 775 (m), 740 (m) cm^{-1}; HRMS (CI(CH_4)): calcd for ($C_{15}H_{19}N$ + H): 214.1590; found: 214.1594.

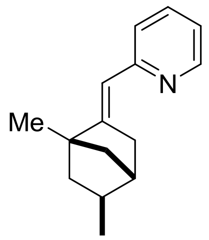

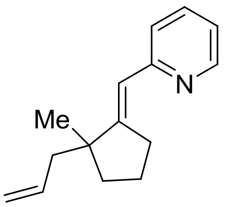

Compound 3m. ^{1}H NMR (500 MHz, $CDCl_3$): δ = 8.57–8.53 (m, 1H), 7.61 – 7.54 (m, 1H), 7.27 – 7.22 (m, 1H), 7.00 (ddd, *J* = 7.5, 5.0, 0.9 Hz, 1H), 6.29 (t, *J* = 2.4 Hz, 1H), 5.84–5.73 (m, 1H), 5.06–4.98 (m, 2H), 2.92–2.82 (m, 1H), 2.80–2.69 (m, 1H), 2.26 – 2.15 (m, 2H), 1.80–1.63 (m, 3H), 1.48–1.41 (m, 1H), 1.13 (s, 3H); ^{13}C NMR (125 MHz, $CDCl_3$): δ = 159.3, 157.7, 149.1, 135.8, 135.6, 122.9, 120.5, 120.2, 116.9, 47.1, 45.4, 38.0, 32.6, 26.6, 23.0.

Compound 5. This compound was obtained from **4** following the representative procedure after separation from **6** and **7** by preparative TLC. Colourless oil; ^{1}H NMR (500 MHz, $CDCl_3$): δ = 8.49 (d, *J* = 4.9 Hz, 1H), 7.48 (td, *J* = 7.7, 1.9 Hz, 1H), 7.38–7.30 (m, 4H), 7.26–7.22 (m, 1H), 7.06 (d, *J* = 8.0 Hz, 1H), 6.95 (ddd, *J* = 7.5, 4.8, 0.8 Hz, 1H), 5.70–5.67 (m, 1H), 3.23 (q, *J* = 6.9 Hz, 1H), 2.18–2.11 (m, 2H), 1.99–1.91 (m, 1H), 1.87 (s, 1H), 1.68 (d, *J* = 10.1 Hz, 1H), 1.51 (dd, *J* = 11.8, 4.9 Hz, 1H), 1.08 (d, *J* = 6.9 Hz, 3H), 1.03 (d, *J* = 6.9 Hz, 3H); ^{13}C NMR (100 MHz, $CDCl_3$): δ = 163.5, 157.2, 149.1, 142.8, 135.6, 128.3 (2C), 128.1 (2C), 126.2, 123.3, 120.2, 119.8, 59.7, 49.9, 43.9, 43.8, 37.5, 36.5, 22.1, 17.3; IR (neat): $\tilde{\nu}$ = 3059 (w), 2953 (s), 2865 (m), 1652 (m), 1584 (s), 1560 (m), 1496 (w), 1471 (s), 1445 (m), 1426 (s), 1374 (w), 1311 (w), 1254 (w), 1236 (w), 1218 (w), 1148 (m), 1118 (w), 1096 (w), 1063 (m), 1035 (w), 991 (m), 946 (w), 931 (m), 890 (m), 865 (m), 778 (m), 763 (s), 753 (s), 741 (s), 698 (s) cm^{-1}; MS (CI(CH_4)): *m/z* (rel. intensity): 290 (100) [M + H]; HRMS (CI(CH_4)) calcd for ($C_{21}H_{23}N$ + H): 290.1903; found: 290.1909.

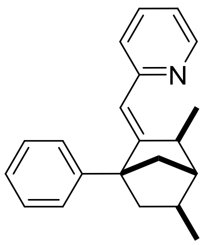

Compound 5-D. This compound was obtained from **4-D** following the representative procedure after separation from **6-D** and **7-D** by preparative TLC. Colourless oil; ^{1}H NMR (500 MHz, $CDCl_3$): δ = 8.49 (ddd, *J* = 4.9, 2.0, 0.9 Hz, 1H), 7.48 (td, *J* = 7.7, 1.9 Hz, 1H), 7.38–7.30 (m, 4H), 7.26–7.22 (m, 1H), 7.06 (d, *J* = 8.0 Hz, 1H), 6.95 (ddd, *J* = 7.5, 4.8, 0.8 Hz, 1H), 5.70–5.67 (m, 1H), 3.23 (qt, *J* = 6.9, 1.9 Hz, 0.88H), 2.18–2.11 (m, 2H), 1.99–1.91 (m, 1H), 1.87 (s, 1H), 1.68 (dt, *J* = 10.1, 1.8 Hz, 1H), 1.51 (dd, *J* = 11.8, 4.9 Hz, 1H), 1.08 (d, *J* = 6.9 Hz, 3H), 1.03 (m, 2.1H); ^{13}C NMR (100 MHz, $CDCl_3$): δ = 163.5, 157.1, 149.1, 142.8, 135.6, 128.3 (2C), 128.1 (2C), 126.2, 123.3, 120.2, 119.8, 59.7, 49.83, 43.9, 43.8, 37.4, 36.4, 21.8 (t, *J* = 19.3 Hz), 17.3 (signals from the minor monodeuterated isomer were visible at 49.79 and 22.1); IR (neat): $\tilde{\nu}$ = 3058 (w), 2958 (m), 2923 (m), 2858 (w), 2170 (w), 1734 (w), 1651 (w), 1584 (m), 1559 (w), 1456 (w), 1471 (m), 1445 (w), 1426 (m), 1374 (w), 1316 (w), 1260 (w), 1234 (w), 1218 (w), 1147 (w), 1092 (w), 1057 (w), 1033 (w), 991 (w), 943 (m), 928 (w), 850

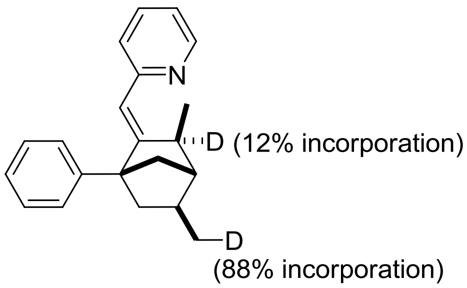

---

[13] IR and HRMS were recorded on the **2m**/**3m** mixture

(w), 865 (w), 801 (w), 777 (w), 762 (m), 752 (m), 740 (m), 698 (s) cm^{-1}; MS (CI(NH_3)): *m*/*z* (rel. intensity): 291 (100) [M + H]; HRMS (CI(NH_3)) calcd for ($C_{21}H_{22}DN$ + H): 290.1966; found: 291.1975.

Compound 6. This compound was obtained from **4** following the representative procedure after separation from **5** and **7** by preparative TLC (mixture of *E* and *Z* isomers (55:45), the sample was contaminated with grease). Colourless oil; ^{1}H NMR (500 MHz, $CDCl_3$):[14] δ = <u>8.59 (ddd, *J* = 4.9, 1.8, 0.9 Hz, 1H)</u>, <u>7.63–7.57 (td, *J* = 7.8, 1.9 Hz, 1H)</u>, <u>7.44–7.38 (m, 2H)</u>, <u>7.30–7.25 (m, 3H)</u>, <u>7.18–7.13 (m, 1H)</u>, <u>7.05 (ddd, *J* = 7.5, 4.9, 1.0 Hz, 1H)</u>, [6.44 (t, *J* = 2.6 Hz, 0.45H)], 6.40 (t, *J* = 2.5 Hz, 0.55H), <u>5.53–5.33 (m, 2H)</u>, <u>2.92–2.82 (m, 2H)</u>, [2.79 (dd, *J* = 14.4, 7.6 Hz, 0.45H)], 2.66 (dd, *J* = 14.0, 7.5 Hz, 0.55H), <u>2.59–2.52 (m, 1H)</u>, [2.23 (ddd, *J* = 12.9, 6.6, 3.4 Hz, 0.45H)], 2.16 (ddd, *J* = 12.6, 6.6, 3.8 Hz, 0.55H), <u>1.90–1.81 (m, 1H)</u>, <u>1.80–1.70 (m, 1H)</u>, <u>1.60–1.55 (m, 1H)</u>, 1.60 (dq, *J* = 6.1, 1.1 Hz, 1.65H), 1.56–1.52 (m, 1.35H); ^{13}C NMR (125 MHz, $CDCl_3$): δ = <u>157.4</u>, <u>156.4</u>, <u>149.2</u>, 146.5, [146.2], <u>135.8</u>, [128.08 (2C)], 128.05 (2C), 127.84, 127.79, [127.2], <u>127.1 (2C)</u>, [125.9], 125.8, [125.7], 123.41, [123.39], 123.37, [123.33], [120.48], 120.45, 56.2, [56.1], 43.6, 37.71, [37.67], [37.48], 32.4, [32.2], <u>22.5</u>, 17.8, [13.1]; IR (neat): $\tilde{\nu}$ = 3055 (w), 3022 (w), 2954 (w), 2920 (w), 2853 (w), 1943 (w), 1731 (w), 1647 (w), 1584 (m), 1558 (w), 1492 (w), 1471 (m), 1444 (m), 1427 (m), 1376 (w), 1286 (w), 1219 (w), 1149 (w), 1092 (w), 1048 (w), 1031 (w), 969 (w), 930 (w), 890 (w), 864 (w), 758 (m), 741 (m), 700 (s) cm^{-1}; MS (CI(CH_4)): *m*/*z* (rel. intensity): 290 (100) [M + H], 234 (58); HRMS (CI(CH_4)) calcd for ($C_{21}H_{23}N$ + H): 290.1903; found: 290.1909.

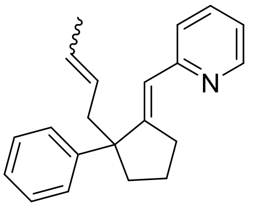

Compound 6-D. This compound was obtained from **4-D** following the representative procedure after separation from **5-D** and **7-D** by preparative TLC (mixture of *E* and *Z* isomers (50:50), the sample was contaminated with grease).[15] Colourless oil; ^{1}H NMR (500 MHz, $CDCl_3$):[13] δ = <u>8.59 (ddd, *J* = 4.9, 1.8, 0.9 Hz, 1H)</u>, <u>7.63–7.57 (td, *J* = 7.8, 1.9 Hz, 1H)</u>, <u>7.44–7.38 (m, 2H)</u>, <u>7.30–7.25 (m, 3H)</u>, <u>7.18–7.13 (m, 1H)</u>, <u>7.05 (ddd, *J* = 7.5, 4.9, 1.0 Hz, 1H)</u>, [6.46–6.42 (m, 0.50H)], 6.41–6.37 (m, 0.50H), <u>5.53–5.33 (m, 1.84H)</u>, <u>2.92–2.82 (m, 1.39H)</u>, [2.79 (dd, *J* = 14.4, 7.6 Hz, 0.50H)], 2.66 (dd, *J* = 14.0, 7.5 Hz, 0.50H), <u>2.59–2.52 (m, 1H)</u>, [2.26–2.20 (m, 0.50H)], 2.21–2.14 (m, 0.50H), <u>1.90–1.81 (m, 1H)</u>, <u>1.80–1.70 (m, 0.88H)</u>, <u>1.60–1.55 (m, 0.84H)</u>, 1.60 (d, *J* = 6.0 Hz, 1.50H), 1.55 (d, *J* = 6.8 Hz, 1.50H); ^{2}D NMR (77 MHz, $CDCl_3$): δ = 5.51 (s, 0.15D), 2.89 (s, 0.60D), 1.78 (s, 0.12D), 1.61 (s, 0.13D); IR (neat): $\tilde{\nu}$ = 3055 (w), 3022 (w), 2954 (w), 2920 (w), 2853 (w), 1943 (w), 1731 (w), 1647 (w), 1584 (m), 1558 (w), 1492 (w), 1471 (m), 1444 (m), 1427 (m), 1376 (w), 1286 (w), 1219 (w), 1149 (w), 1092 (w), 1048 (w), 1031 (w), 969 (w), 930 (w), 890 (w), 864 (w), 758 (m), 741 (m), 700 (s) cm^{-1}; MS (CI(CH_4)): *m*/*z* (rel. intensity): 291 (100) [M + H], 235 (49); HRMS (CI(CH_4)) calcd for ($C_{21}H_{22}DN$ + H): 291.1966; found: 291.1969.

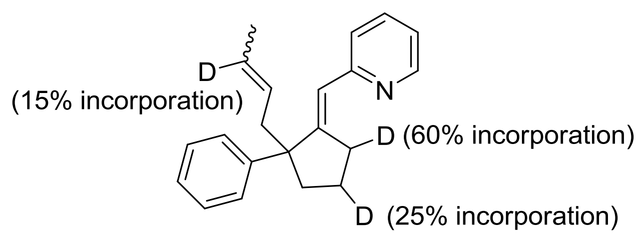

6-D
(*E*/*Z* = 1:1)

---

[14] The signals indicated in brackets correspond to the *Z* isomer, those underlined both to the *E* and *Z* isomers, the others to the *E* isomer.
[15] The percentage of deuterium incorporation at the indicated positions was determined by ^{2}H NMR.

Compound 7. This compound was obtained from **4** following the representative procedure after separation from **5** and **6** by preparative TLC (the sample was contaminated with grease). Colourless oil; ^{1}H NMR (500 MHz, $CDCl_3$): δ = 8.49 (d, *J* = 4.7 Hz, 1H), 7.56 (t, *J* = 7.7 Hz, 1H), 7.35–7.26 (m, 4H), 7.22–7.14 (m, 2H), 7.07–7.02 (m, 1H), 6.91 (d, *J* = 15.9 Hz, 1H), 6.24 (d, *J* = 15.9 Hz, 1H), 5.78 (ddd, *J* = 17.5, 10.1, 7.7 Hz, 1H), 4.98 (d, *J* = 17.0 Hz, 1H), 4.94 (d, *J* = 10.2 Hz, 1H), 2.55–2.47 (m, 1H), 2.44 (dd, *J* = 12.3, 6.3 Hz, 1H), 2.21–2.11 (m, 2H), 2.00–1.92 (m, 1H), 1.87 (t, *J* = 11.7 Hz, 1H), 0.98 (d, *J* = 6.3 Hz, 3H); ^{13}C NMR (100 MHz, $CDCl_3$): δ = 156.1, 149.4, 148.2, 145.0, 142.5, 136.3, 128.2 (2C), 127.0 (2C), 126.3, 125.9, 121.6, 114.0, 52.1, 51.8, 47.4, 44.6, 39.4, 18.0; IR (neat): $\tilde{\nu}$ = 3061 (w), 2953 (s), 2924 (s), 2867 (m), 1641 (m), 1585 (s), 1563 (m), 1494 (m), 1469 (s), 1446 (m), 1429 (s), 1375 (w), 1306 (w), 1148 (w), 1049 (w), 1033 (w), 991 (m), 977 (m), 910 (m), 758 (s), 741 (s), 700 (s) cm^{-1}; MS (CI(CH_4)): *m*/*z* (rel. intensity): 290 (100) [M + H]; HRMS (CI(CH_4)) calcd for ($C_{21}H_{23}N$ + H): 290.1903; found: 290.1908.

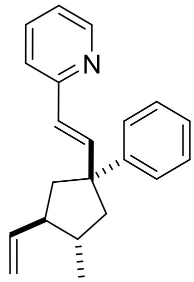

Compound 7-D. This compound was obtained from **4-D** following the representative procedure after separation from **5-D** and **6-D** by preparative TLC (the sample was contaminated with grease).[16] Colourless oil; ^{1}H NMR (500 MHz, $CDCl_3$): δ = 8.49 (ddd, *J* = 4.8, 1.5, 0.7 Hz, 1H), 7.55 (td, *J* = 7.7, 1.9 Hz, 1H), 7.35–7.26 (m, 4H), 7.22–7.14 (m, 2H), 7.05 (ddd, *J* = 7.5, 4.9, 1.1 Hz, 1H), 6.91 (d, *J* = 15.9 Hz, 1H), 6.24 (d, *J* = 15.9 Hz, 1H), 5.78 (ddd, *J* = 17.5, 10.1, 7.7 Hz, 1H), 4.98 (dd, *J* = 17.1, 1.4 Hz, 1H), 4.94 (dd, *J* = 10.2, 1.9 Hz, 1H), 2.55–2.47 (m, 1H), 2.44 (dd, *J* = 12.6, 6.2 Hz, 1H), 2.21–2.11 (m, 2H), 2.00–1.92 (m, 1H), 1.87 (t, *J* = 11.2 Hz, 1H), 1.00–0.95 (m, 2H); ^{2}D NMR (77 MHz, $CDCl_3$): δ = 5.86 (s, 0.11D), 1.02 (s, 0.89D); ^{13}C NMR (100 MHz, $CDCl_3$): δ = 156.1, 149.4, 148.2, 145.0, 142.5, 136.3, 128.2 (2C), 127.0 (2C), 126.3, 125.9, 121.6, 114.0, 52.1, 51.78, 47.4, 44.58, 39.36, 17.8 (t, *J* = 19.1 Hz) (signals of the minor mono-deuterated isomer were also visible: 51.69, 44.59, 39.42, 18.1); IR (neat): $\tilde{\nu}$ = 3058 (w), 2924 (w), 2856 (w), 2165 (w), 1726 (w), 1641 (w), 1585 (m), 1563 (w), 1494 (w), 1469 (m), 1446 (w), 1429 (m), 1282 (w), 1148 (w), 1091 (w), 1049 (w), 1033 (w), 991 (w), 977 (w), 910 (w), 844 (w), 757 (m), 742 (w), 700 (m) cm^{-1}; MS (CI(CH_4)): *m*/*z* (rel. intensity): 291 (100) [M + H]; HRMS (CI(CH_4)) calcd for ($C_{21}H_{23}DN$ + H): 291.1966; found: 291.1956.

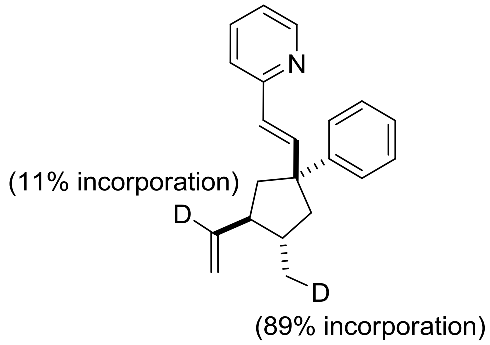

[16] The percentage of deuterium incorporation at the indicated positions was determined by ^{2}H NMR.

Compound 8. Under N_2, a stock solution of $RuCl_3.xH_2O$ (7 mg in 0.7 mL of degassed H_2O) was added by syringe to a J-Young Schlenk flask containing a degassed suspension of **2a** (47mg, 0.17 mmol) and sodium periodate (146 mg, 0.68 mmol) in 1,2-dichloroethane (0.5 mL) and CH_3CN (0.5 mL). The flask was sealed and the suspension was stirred vigorously at 40 °C overnight. At room temperature, a saturated solution of $Na_2S_2O_3$ and a saturated solution of NH_4Cl were added, and the mixture was extracted 3 times with dichloromethane (5 mL). The combined organic layers were dried over Na_2SO_4, filtered and concentrated. Purification by flash chromatography (petroleum ether/Et_2O, 10/1 → 7/1) gave **8** as a white solid (23 mg, 68%). mp.: 28–31°C ;^{1}H NMR (500 MHz, $CDCl_3$): δ = 7.36–7.31 (m, 2H), 7.27–7.23 (m, 3H), 2.37–2.28 (m, 2H), 2.21 (ddd, *J* = 12.8, 8.3, 2.1 Hz, 1H), 2.18–2.08 (m, 3H), 2.06–1.98 (m, 1H), 1.48 (dd, *J* = 12.8, 4.7 Hz, 1H), 1.12 (d, *J* = 7.0 Hz, 3H); ^{13}C NMR (125 MHz, $CDCl_3$): δ = 215.7, 138.1, 128.1 (2C), 127.5 (2C), 126.8, 62.3, 46.2, 40.1, 39.9, 38.7, 36.0, 22.1; IR (neat): $\tilde{\nu}$ = 3059 (w), 3028 (w), 2955 (m), 1741 (s), 1604 (w), 1499 (m), 1471 (w), 1446 (m), 1408 (w), 1377 (w), 1349 (w), 1332 (w), 1291 (w), 1257 (w), 1220 (w), 1185 (w), 1159 (w), 1140 (w), 1109 (w), 1067 (m), 1056 (w), 1035 (w), 1021 (w), 1005 (w), 954 (m), 938 (w), 926 (w), 915 (w), 882 (w), 851 (w), 807 (w), 757 (s), 696 (s) cm^{-1}; elemental analysis (%) calcd for $C_{14}H_{16}O$: C 83.96, H 8.05; found: C 83.95, H 8.05.

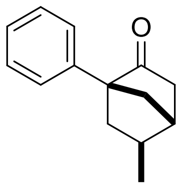

Compound 9. The same procedure was applied to **2a** (20mg, 0.0726 mmol) but the crude material was dissolved in toluene (0.8 mL) under N_2. At -78°C, DIBAL (0.29 ml, 0.29 mmol, 1M in heptane) was added. After stirring at -78°C for 1 hour, MeOH (0.3 mL) was added, followed by a saturated aqueous solution of NH_4Cl (0.1 mL) before stirring at room temperature for 30 minutes. Et_2O was added (10 mL) and the suspension was dried over $MgSO_4$, filtered over a Celite, rinsing with Et_2O. The filtrate was concentrated. Purification by flash column chromatography (petroleum ether/EtOAc: 12/1) afforded compound **8** as a white solid (11 mg, 70 % over 2 steps, d.r. > 20:1). mp.: 67-71°C ;^{1}H NMR (500 MHz, $CDCl_3$): δ = 7.31–7.28 (m, 4H), 7.23–7.17 (m, 1H), 4.08–4.01 (m, 1H), 2.67 (ddd, *J* = 12.5, 8.5, 2.0 Hz, 1H), 2.23 (ddd, *J* = 13.0, 10.3, 4.8 Hz, 1H), 1.94–1.85 (m, 2H), 1.81–1.72 (m, 2H), 1.53 (d, *J* = 4.0 Hz, 1H(OH)), 1.12 (dt, *J* = 13.0, 3.4 Hz, 1H), 1.07 (ddd, *J* = 12.4, 4.5, 1.9 Hz, 1H), 0.96 (d, *J* = 7.0 Hz, 3H); ^{13}C NMR (125 MHz, $CDCl_3$): δ = 144.0, 128.3 (2C), 126.7 (2C), 126.1, 78.1, 56.1, 42.8, 40.6, 38.8, 37.4, 35.6, 22.4; IR (neat): $\tilde{\nu}$ = 3267 (br), 3086 (w), 3059 (w), 3025 (w), 2951 (s), 2865 (m), 2737 (w), 1603 (w), 1494 (w), 1445 (w), 1418 (w), 1372 (w), 1352 (w), 1330 (w), 1279 (w), 1247 (w), 1198 (w), 1149 (w), 1124 (w), 1099 (w), 1083 (w), 1056 (s), 1034 (w), 1006 (w), 974 (w), 941 (w), 911 (w), 900 (w), 855 (w), 804 (w), 755 (m), 695 (s), 659 (w) cm^{-1}; MS (CI(CH_4)): *m/z* (rel. intensity): 201 (5) [M], 185 (100) [M – H_2O + H], 157 (24), 81 (45); HRMS (CI(CH_4)): calcd for (($C_{14}H_{18}O$ – H_2O) + H): 185.1325; found: 185.1327.

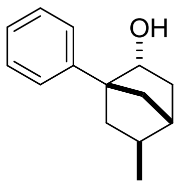

Compound 10. From a solution of tBuOK (0.143 mmol, 16 mg) in DMSO (1.6 mL), 60 µL was added via syringe to a solution of **8** (0.055 mmol, 11 mg) and para-anisaldehyde (0.055 mmol, 6.7 µL) in DMSO (0.1 mL) at 10 °C under N_2. After stirring for 16 hours at room temperature, the mixture was partitioned between Et_2O and water and the organic layer was dried over Na_2SO_4, filtered and concentrated. The solid thus obtained was triturated and washed with pentane to give **10** as a white solid (11.9 mg, 68%). mp.: 120–124°C;^{1}H NMR (500 MHz, $CDCl_3$): δ = 7.45 (d, *J* = 8.8 Hz, 2H), 7.39–7.34 (m, 2H), 7.33–7.29 (m, 2H), 7.29–7.25 (m, 1H), 7.18 (s, 1H), 6.94 (d, *J* = 8.8 Hz, 2H), 3.84 (s, 3H), 3.32 (s, 1H), 2.35 (ddd, *J* = 12.5, 8.3, 2.2 Hz, 1H), 2.32–2.25 (m, 1H), 2.28 (dd, *J* = 10.2, 1.7 Hz, 1H), 2.09 (dq, *J* = 10.3, 1.5 Hz, 1H), 1.61 (dd, *J* = 12.5, 4.0 Hz, 1H), 1.27 (d, *J* = 6.8 Hz, 3H); ^{13}C NMR (125 MHz, $CDCl_3$): δ = 205.0, 160.3, 139.8, 138.6, 131.5 (2C), 128.2 (2C), 127.9, 127.7 (2C), 127.5, 126.9, 114.3 (2C), 61.3, 55.4, 45.3, 40.5, 38.6, 35.9, 22.2; IR (neat): $\tilde{\nu}$ = 3057 (w), 3027 (w), 2961 (w), 2923 (w), 2866 (w), 2837 (w), 1716 (s), 1641 (s), 1602 (s), 1510 (s), 1458 (m), 1446 (m), 1373 (w), 1334 (w), 1248 (s), 1176 (s), 1152 (s), 1116 (w), 1088 (w), 1069 (s), 1020 (s), 981 (w), 965 (w), 949 (w), 932 (s), 912 (s), 866 (m), 848

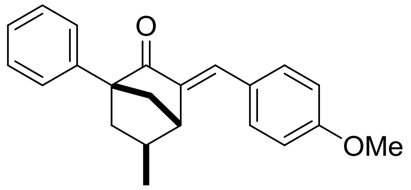

(m), 835 (s), 823 (m), 809 (m), 763 (m), 752 (m), 736 (w), 695 (s), 656 (m) cm^{-1}; MS (CI(NH_3)): *m/z* (rel. intensity): 319 (100) [M]; HRMS (CI(NH_3)): calcd for ($C_{22}H_{22}O_2$+ H): 319.1693; found: 319.1698.

Compound 11. Compound **8** (0.109 mmol, 21.7 mg) was added to a solution of mCPBA (0.271 mmol, 61 mg (77%)) and $NaHCO_3$ (0.543 mmol, 46 mg) in dichloromethane (2 mL) at 0 °C under N_2. After stirring for 40 hours at room temperature, the mixture was partitioned between water and dichloromethane and the organic layer was dried over Na_2SO_4, filtered and concentrated. Purification by flash chromatography (petroleum ether/EtOAc, 15/1 → 4/1) gave **11** as a colourless oil (22 mg, 95%). ^{1}H NMR (500 MHz, $CDCl_3$): δ = 7.50–7.45 (m, 2H), 7.39–7.33 (m, 2H), 7.32–7.27 (m, 1H), 2.76 (ddd, *J* = 15.0, 8.6, 2.7 Hz, 1H), 2.75 (dd, *J* = 18.5, 4.9 Hz, 1H), 2.64 (dt, *J* = 18.4, 2.0 Hz, 1H), 2.37 (ddd, *J* = 12.9, 5.4, 2.0 Hz, 1H), 2.26–2.17 (m, 2H), 2.03 (dd, *J* = 12.9, 2.4 Hz, 1H), 1.69 (dd, *J* = 14.8, 5.1 Hz, 1H), 1.12 (d, *J* = 7.1 Hz, 3H); ^{13}C NMR (125 MHz, $CDCl_3$): δ = 170.4, 140.3, 128.4 (2C), 128.0, 125.3 (2C), 92.2, 48.9, 39.3, 38.6, 37.8, 22.6; IR (neat): $\tilde{\nu}$ = 3060 (w), 2954 (w), 2869 (w), 1731 (s), 1497 (w), 1149 (w), 1438 (w), 1415 (w), 1378 (w), 1361 (w), 1325 (w), 1276 (m), 1261 (m), 1239 (m), 1198 (s), 1164 (w), 1143 (w), 1086 (s), 1049 (s), 1020 (w), 1000 (s), 959 (s), 938 (m), 925 (w), 887 (w), 850 (w), 757 (s), 696 (s), 657 (w) cm^{-1}; MS (CI(NH_3)): *m/z* (rel. intensity): 234 (24) [M + NH_4], 217 (100) [M + H]; HRMS (CI(NH_3)): calcd for (($C_{14}H_{16}O_2$) + H): 217.1223; found: 217.1229.

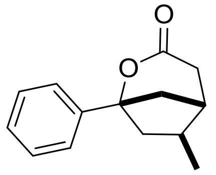

Compound 12. Obtained from **1i** (0.117 mmol, 45 mg) using the representative procedure for the Rh(I)-catalysed cycloisomerisation, except that the reaction was stopped after 30 minutes heating. Purification by flash column chromatography (petroleum ether/EtOAc, 10:1) yielded the title compound as colourless oil (15 mg, 33%). ^{1}H NMR ($CDCl_3$, 500 MHz): δ = 8.53 (ddd, *J* = 4.7, 1.6 and 0.7 Hz, 1H), 7.72 (d, *J* = 8.2 Hz, 2H), 7.59 (td, *J* = 7.7, 1.6 Hz, 1H), 7.25 (d, *J* = 8.0 Hz, 2H), 7.18 (d, *J* = 7.8 Hz, 1H), 7.06 (ddd, *J* = 7.5, 4.7, 0.8 Hz, 1H), 6.50 (d, *J* = 2.2 Hz, 1H), 5.90–5.79 (m, 1H), 5.18–5.12 (m, 1H), 5.09 (dt, *J* = 10.2, 0.8 Hz, 1H), 3.26–3.15 (m, 1H), 2.92 (s, 3H), 2.94–2.86 (m, 1H), 2.77 (dd, *J* = 13.9, 8.5 Hz, 1H), 2.55 (dd, *J* = 12.8, 9.0 Hz, 1H), 2.38 (s, 3H), 2.27 (dd, *J* = 12.8, 5.3 Hz, 1H), 1.12 (d, *J* = 7.0 Hz, 3H); ^{13}C NMR ($CDCl_3$, 125 MHz): δ = 155.2, 152.0, 149.5, 143.0, 138.6, 136.1, 133.8, 129.4 (2C), 127.3 (2C), 127.1, 123.0, 121.4, 118.9, 68.6, 42.5, 37.0, 34.6, 33.6, 21.5, 18.5; IR (neat) $\tilde{\nu}$ = 3074 (w), 2958 (w), 2869 (w), 1665 (w), 1638 (w), 1584 (m), 1563 (w), 1494 (w), 1469 (m), 1430 (m), 1329 (s), 1304 (m), 1289 (m), 1270 (w), 1214 (w), 1184 (w), 1150 (s), 1086 (s), 1018 (w), 993 (w), 893 (s), 846 (m), 813 (s), 743 (s), 707 (m), 688 (w), 658 (s) cm^{-1}; MS (ESI): *m/z* (rel. intensity): 421 (12) [M + K], 405 (27) [M + Na], 383 (100) [M + H]; HRMS (ESI) calcd for ($C_{22}H_{26}N_2O_2S$ + H): 383.1793, found: 383.1792.

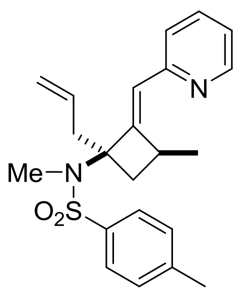

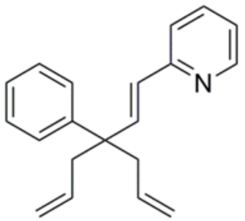

1a

```
Current Data Parameters
NAME        KHO-07-134-01
EXPNO                   1
PROCNO                  1

F2 - Acquisition Paramet
Date_            20120425
Time                15.11
INSTRUM            DRX500
PROBHD   5 mm PABBO BB-
PULPROG              zg30
TD                  65536
SOLVENT             CDCl3
NS                     16
DS                      2
SWH             10288.065
FIDRES           0.156983
AQ              3.1850996
RG                  203.2
DW                 48.600
DE                   6.00
TE                  293.7
D1             1.00000000
TD0                     1

======== CHANNEL f1 ====
NUC1                   1H
P1                   9.80
PL1                 -3.60
SFO1          500.1330885

F2 - Processing paramete
SI                  32768
SF            500.1300228
WDW                    EM
SSB                     0
LB                   0.30
GB                      0
PC                   1.00
```

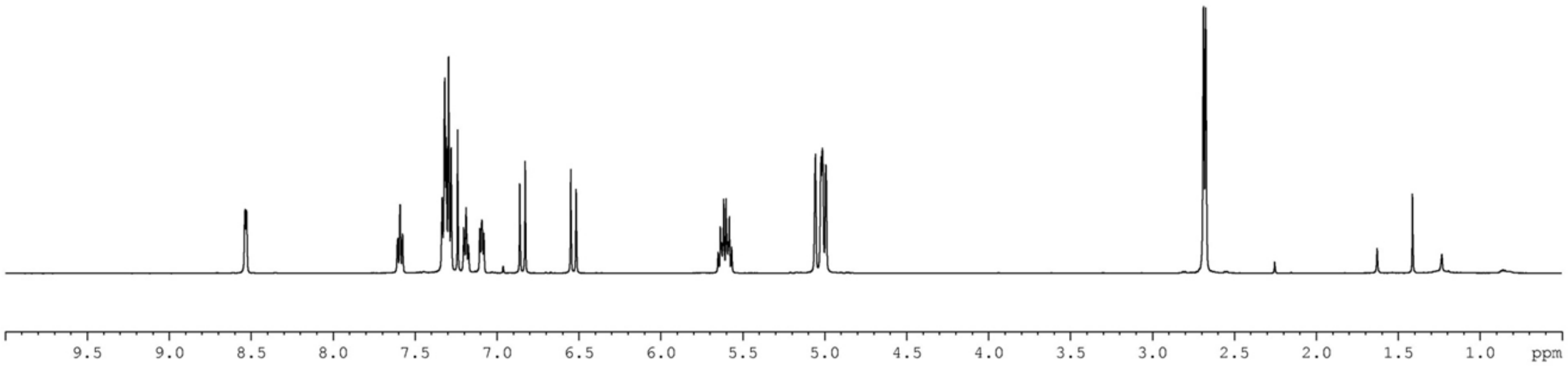

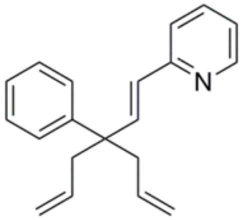

1a

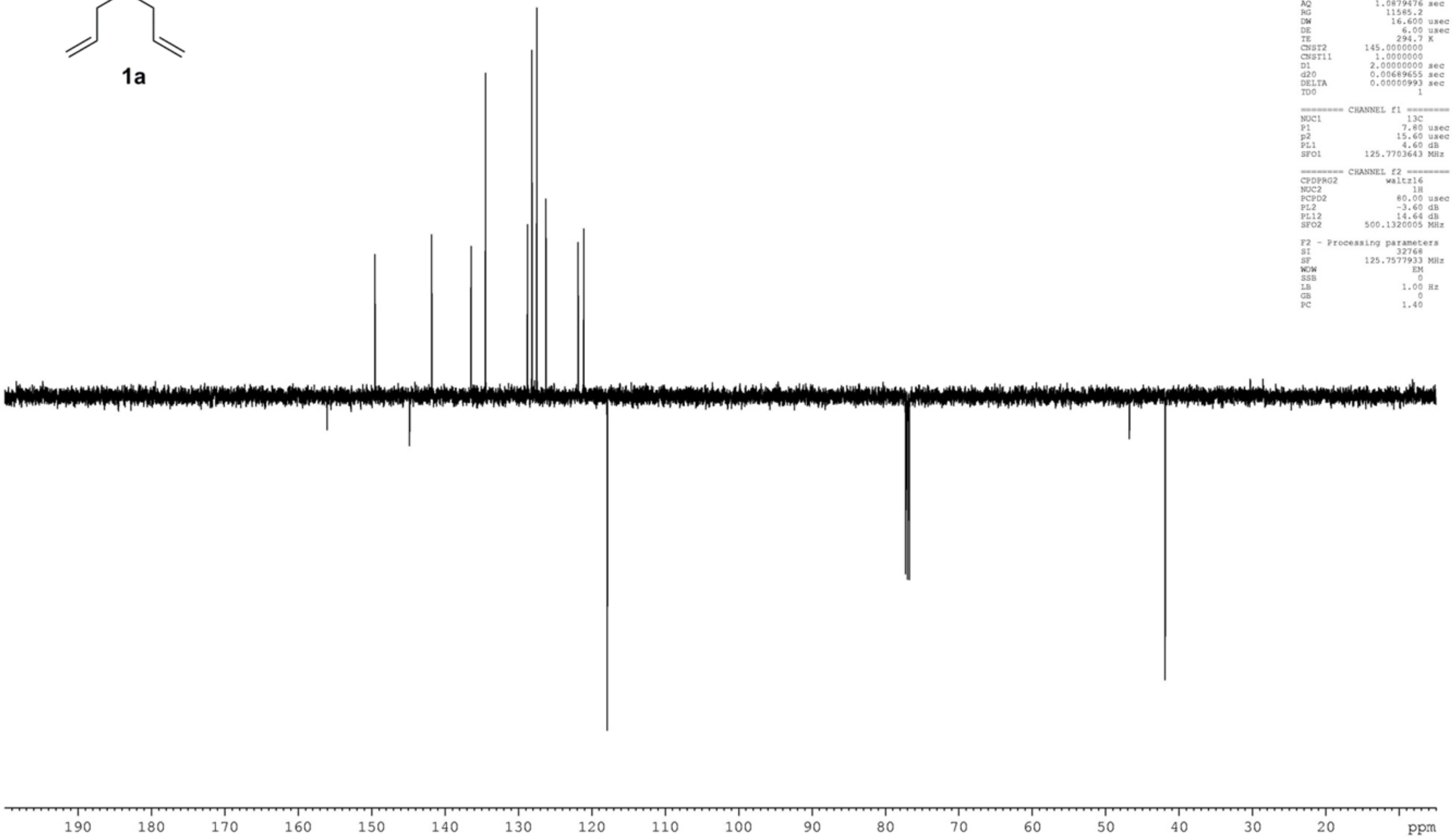
190
180
170
160
150
140
130
120
110
100
90
80
70
60
50
40
30
20
ppm

Current Data Parameters
NAME KHO-07-134-01
EXPNO 2
PROCNO 1

F2 - Acquisition Parameters
Date_ 20120425
Time 15.14
INSTRUM DRX500
PROBHD 5 mm PABBO BB-
PULPROG jmod
TD 65536
SOLVENT CDCl3
NS 132
DS 4
SWH 30120.482 Hz
FIDRES 0.459602 Hz
AQ 1.0879476 sec
RG 11585.2
DW 16.600 usec
DE 6.00 usec
TE 294.7 K
CNST2 145.0000000
CNST11 1.0000000
D1 2.00000000 sec
d20 0.00689655 sec
DELTA 0.00000993 sec
TD0 1

======== CHANNEL f1 ========
NUC1 13C
P1 7.80 usec
p2 15.60 usec
PL1 4.60 dB
SFO1 125.7703643 MHz

======== CHANNEL f2 ========
CPDPRG2 waltz16
NUC2 1H
PCPD2 80.00 usec
PL2 -3.60 dB
PL12 14.64 dB
SFO2 500.1320005 MHz

F2 - Processing parameters
SI 32768
SF 125.7577933 MHz
WDW EM
SSB 0
LB 1.00 Hz
GB 0
PC 1.40

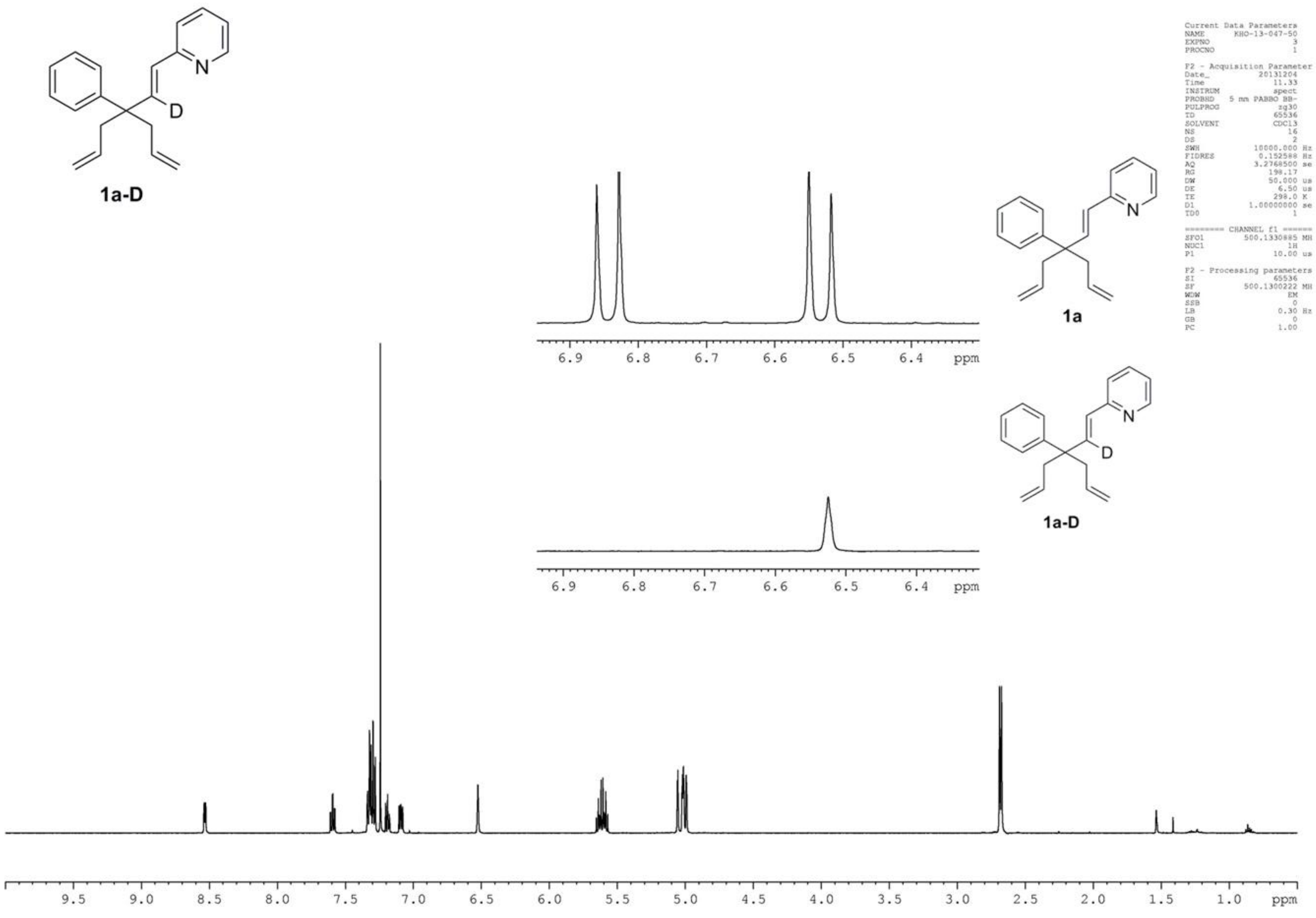
1a-D
D
N
1a
Current Data Parameters
NAME KHO-13-047-50
EXPNO 3
PROCNO 1
F2 - Acquisition Parameter
Date_ 20131204
Time 11.33
INSTRUM spect
PROBHD 5 mm PABBO BB-
PULPROG zg30
TD 65536
SOLVENT CDCl3
NS 16
DS 2
SWH 10000.000 Hz
FIDRES 0.152588 Hz
AQ 3.2768500 se
RG 198.17
DW 50.000 us
DE 6.50 us
TE 298.0 K
D1 1.00000000 se
TD0 1
======== CHANNEL f1 ======
SFO1 500.1330885 MH
NUC1 1H
P1 10.00 us
F2 - Processing parameters
SI 65536
SF 500.1300222 MH
WDW EM
SSB 0
LB 0.30 Hz
GB 0
PC 1.00
6.9 6.8 6.7 6.6 6.5 6.4 ppm
9.5 9.0 8.5 8.0 7.5 7.0 6.5 6.0 5.5 5.0 4.5 4.0 3.5 3.0 2.5 2.0 1.5 1.0 ppm

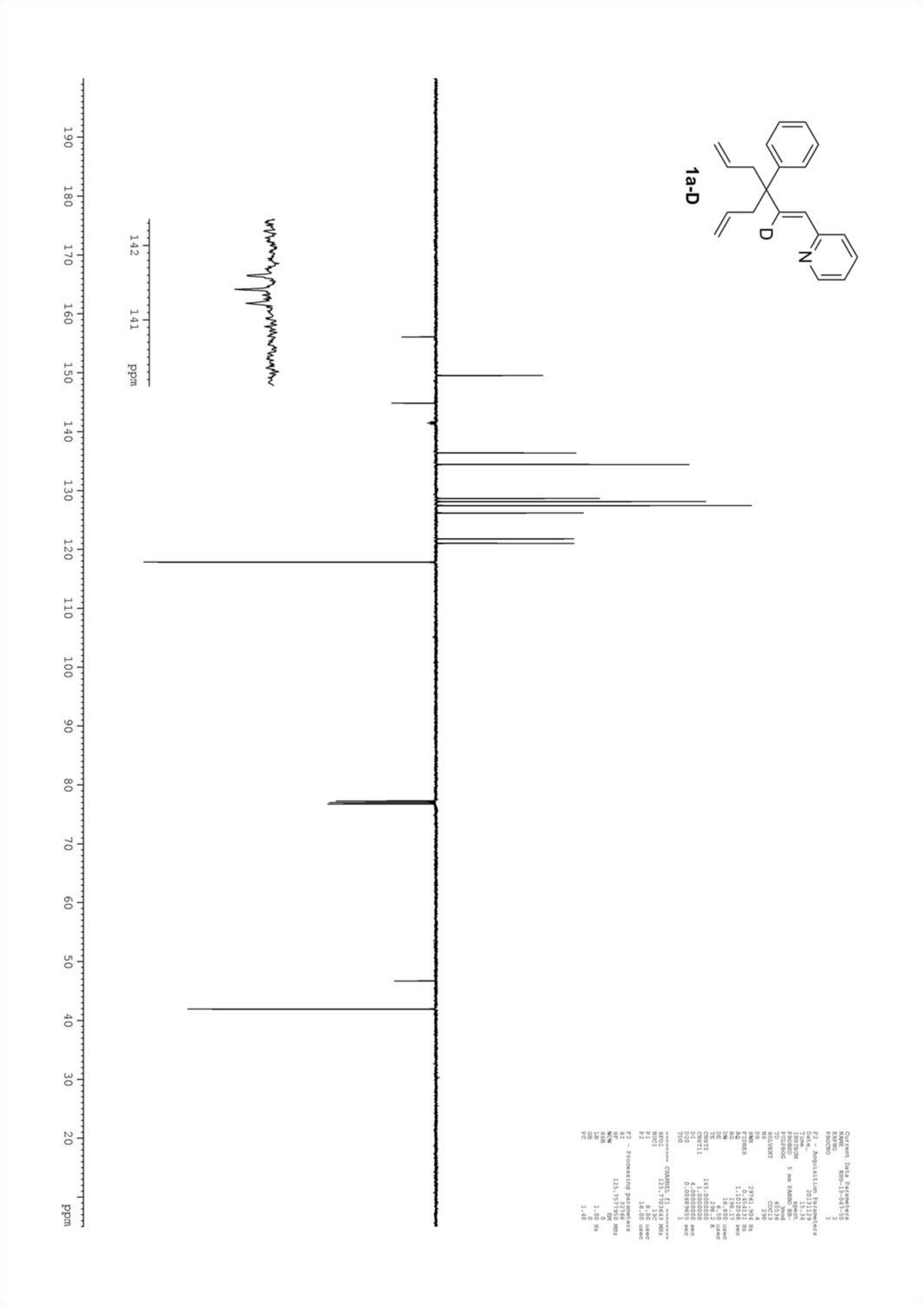
1a-D
D
N
190 180 170 160 150 140 130 120 110 100 90 80 70 60 50 40 30 20 ppm
142 141 ppm

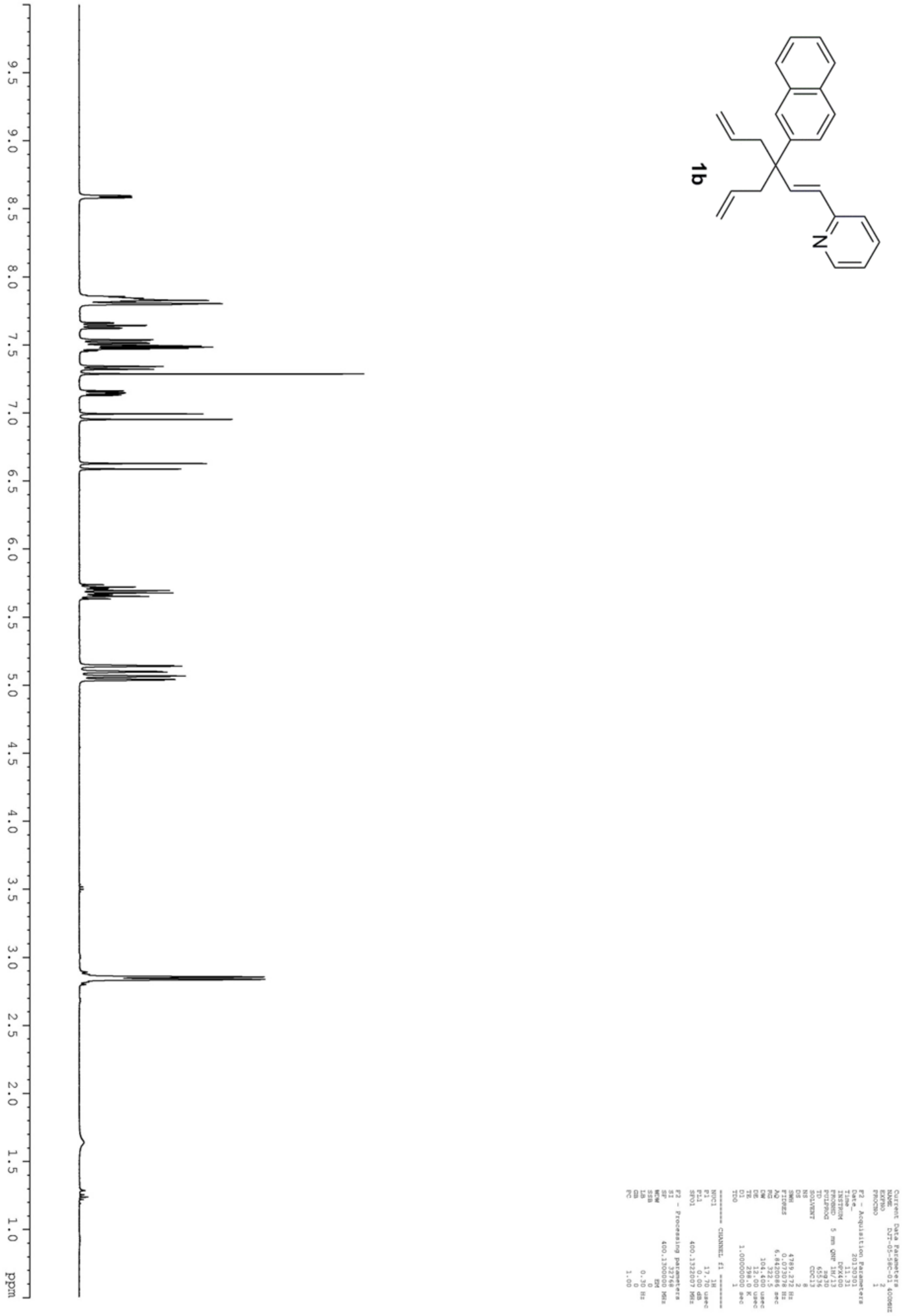
1b
ppm

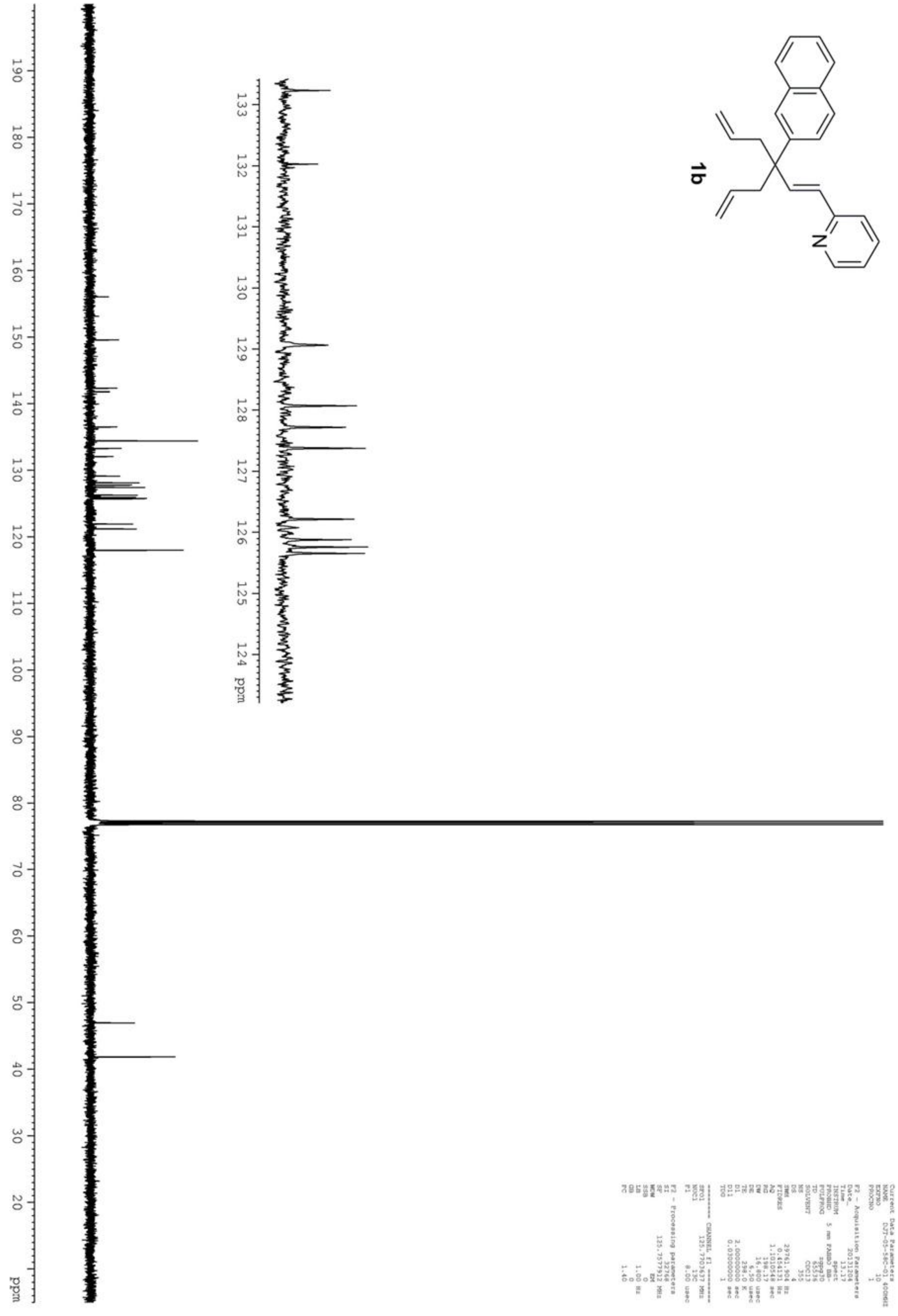
1b
ppm

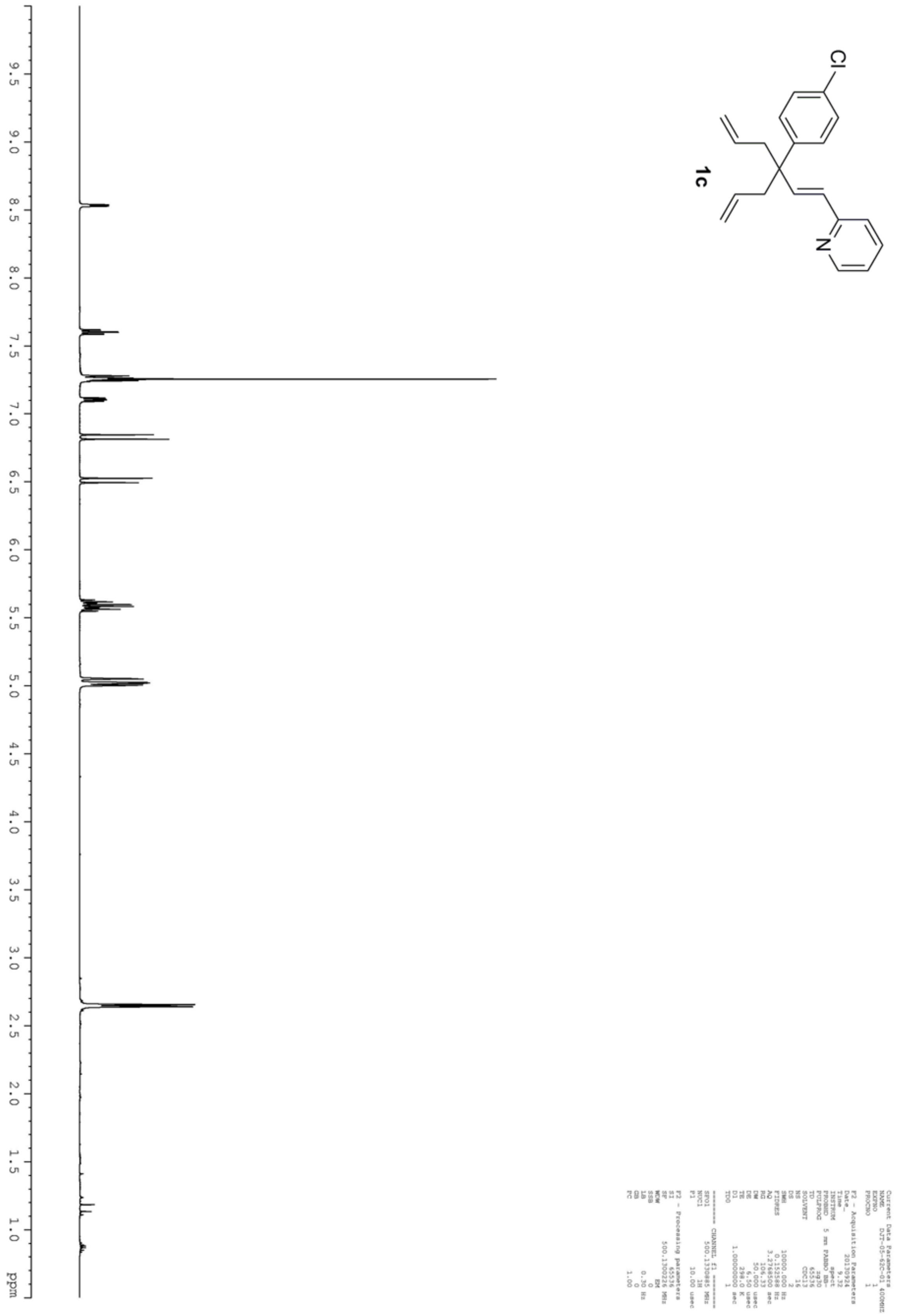
Cl
N
1c
9.5
9.0
8.5
8.0
7.5
7.0
6.5
6.0
5.5
5.0
4.5
4.0
3.5
3.0
2.5
2.0
1.5
1.0
ppm

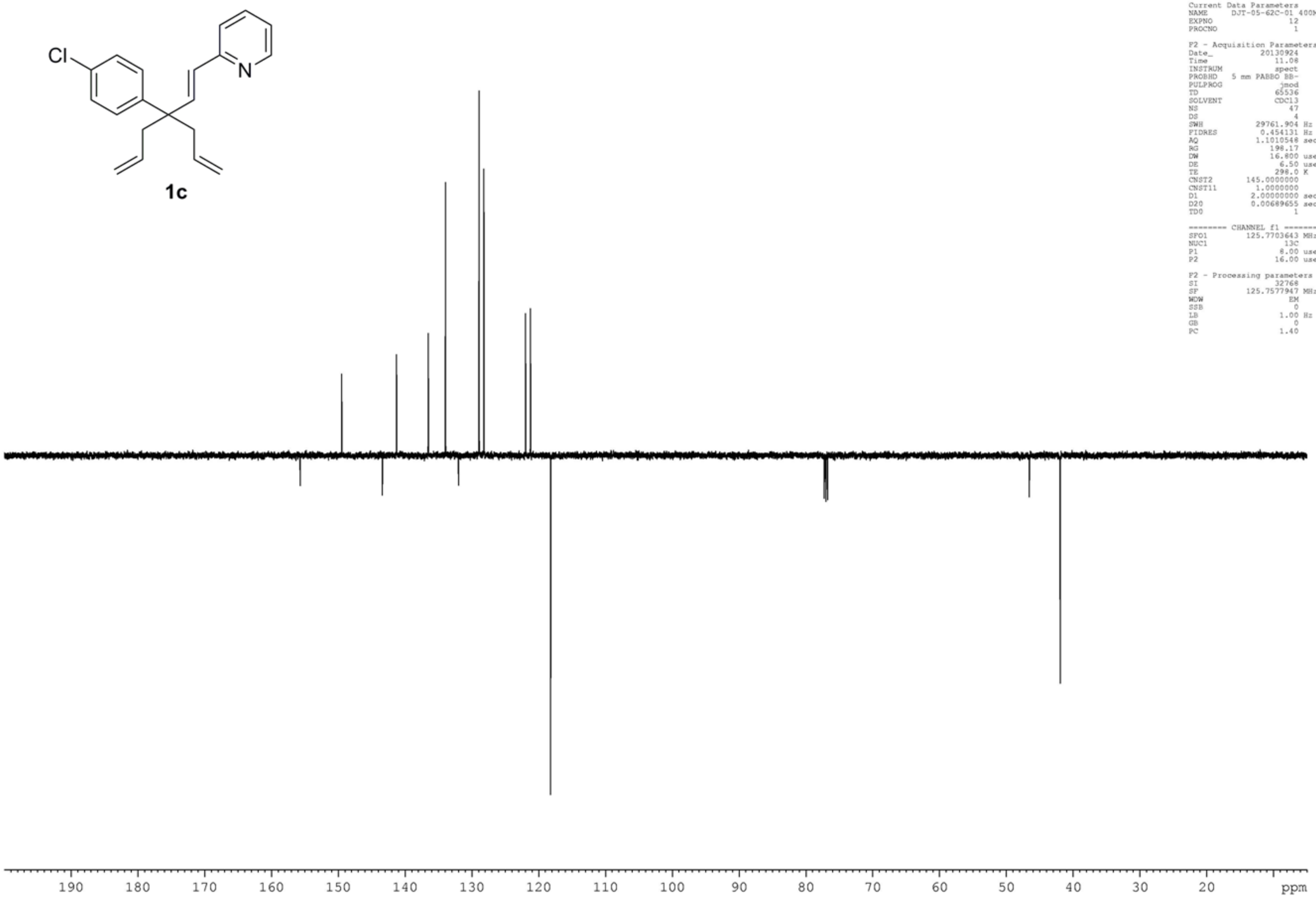

1c

Current Data Parameters		
NAME	DJT-05-62C-01 400MH	
EXPNO	12	
PROCNO	1	
F2 - Acquisition Parameters		
Date_	20130924	
Time	11.08	
INSTRUM	spect	
PROBHD	5 mm PABBO BB-	
PULPROG	jmod	
TD	65536	
SOLVENT	CDCl3	
NS	47	
DS	4	
SWH	29761.904	Hz
FIDRES	0.454131	Hz
AQ	1.1010548	sec
RG	198.17	
DW	16.800	usec
DE	6.50	usec
TE	298.0	K
CNST2	145.0000000	
CNST11	1.0000000	
D1	2.00000000	sec
D20	0.00689655	sec
TD0	1	
======== CHANNEL f1 ========		
SFO1	125.7703643	MHz
NUC1	13C	
P1	8.00	usec
P2	16.00	usec
F2 - Processing parameters		
SI	32768	
SF	125.7577947	MHz
WDW	EM	
SSB	0	
LB	1.00	Hz
GB	0	
PC	1.40	

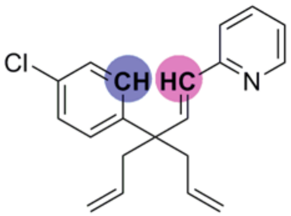

Cl
CH
HC
N

1c
key HSQC

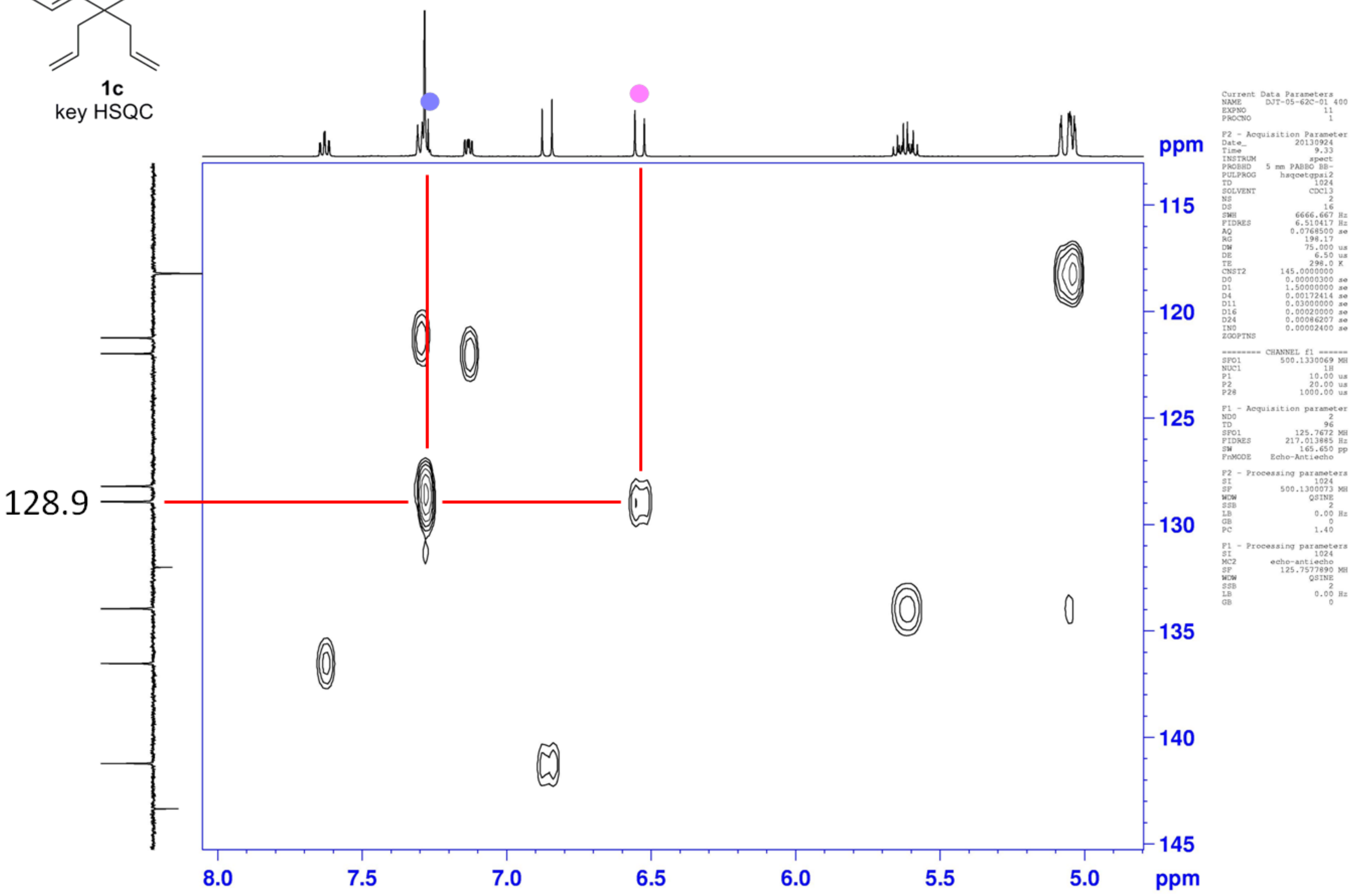

128.9
ppm
115
120
125
130
135
140
145
8.0
7.5
7.0
6.5
6.0
5.5
5.0
ppm

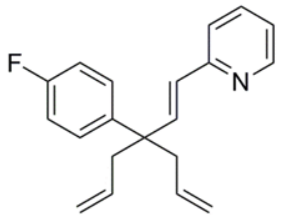

1d

Current Data Parameters
NAME DJT-07-36-01TEST
EXPNO 1
PROCNO 1

F2 - Acquisition Parameters
Date_ 20130924
Time 13.07
INSTRUM spect
PROBHD 5 mm PABBO BB-
PULPROG zg30
TD 65536
SOLVENT CDCl3
NS 8
DS 2
SWH 10000.000 Hz
FIDRES 0.152588 Hz
AQ 3.2768500 sec
RG 134.89
DW 50.000 usec
DE 6.50 usec
TE 298.0 K
D1 1.00000000 sec
TD0 1

======== CHANNEL f1 ========
SFO1 500.1330885 MHz
NUC1 1H
P1 10.00 usec

F2 - Processing parameters
SI 65536
SF 500.1300000 MHz
WDW EM
SSB 0
LB 0.30 Hz
GB 0
PC 1.00

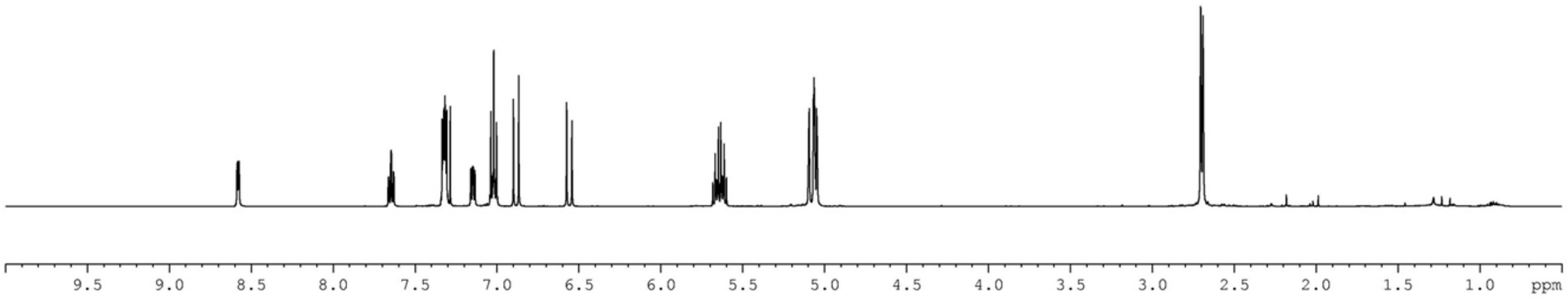

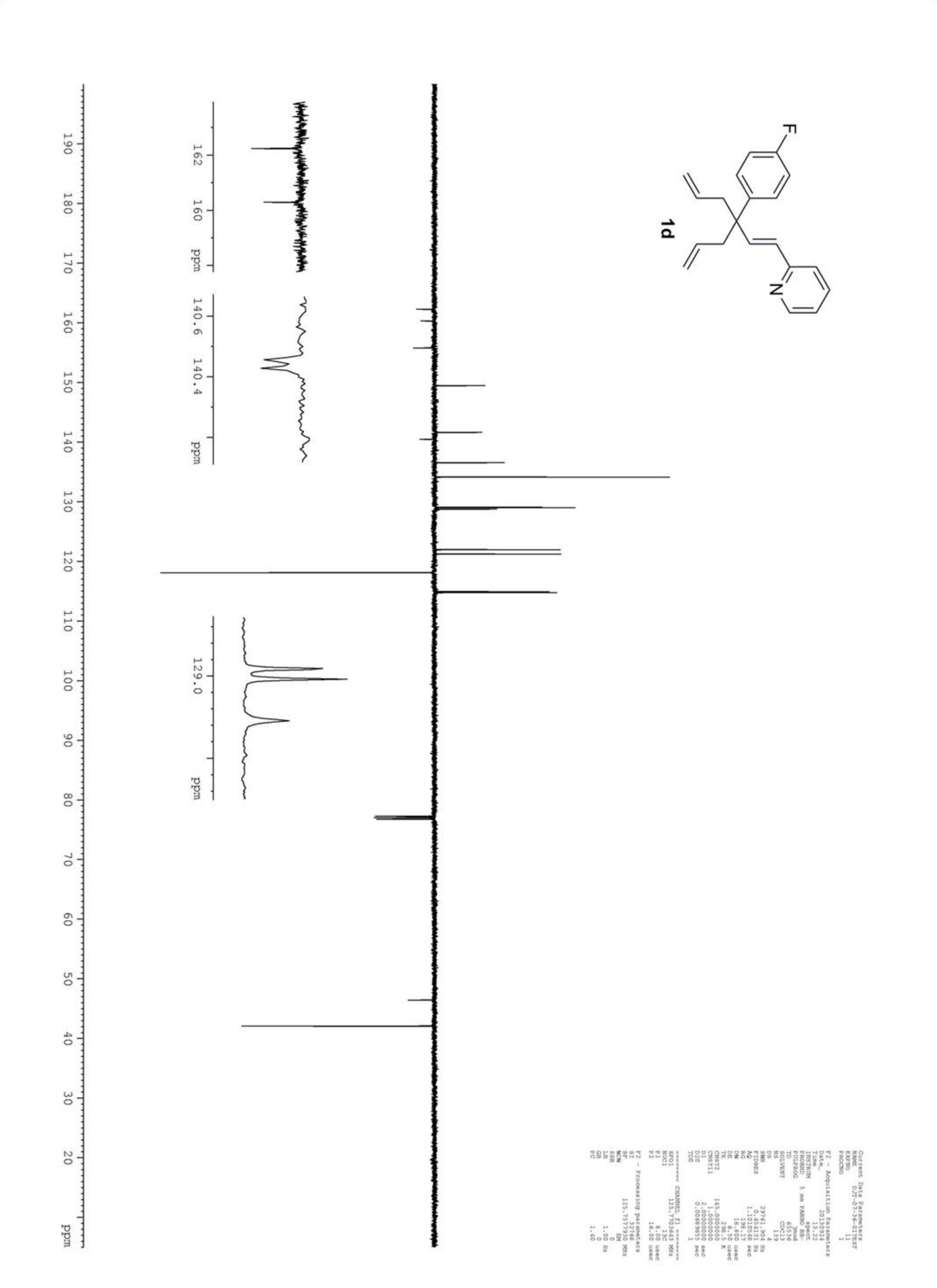
1d
ppm
190
180
170
160
150
140
130
120
110
100
90
80
70
60
50
40
30
20
162
160
140.6
140.4
129.0

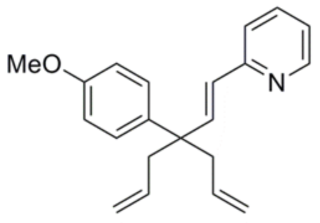

1e

```
Current Data Parameters
NAME       DJT-05-81B-01TEST
EXPNO                      2
PROCNO                     1

F2 - Acquisition Parameters
Date_               20131209
Time                   13.58
INSTRUM                spect
PROBHD    5 mm PABBO BB-
PULPROG                 zg30
TD                     65536
SOLVENT                CDCl3
NS                         8
DS                         2
SWH             10000.000 Hz
FIDRES           0.152588 Hz
AQ              3.2768500 sec
RG                    155.66
DW                 50.000 usec
DE                   6.50 usec
TE                  298.0 K
D1             1.00000000 sec
TD0                        1

======== CHANNEL f1 ========
SFO1          500.1330885 MHz
NUC1                      1H
P1                  10.00 usec

F2 - Processing parameters
SI                     65536
SF            500.1300226 MHz
WDW                       EM
SSB                        0
LB                     0.30 Hz
GB                         0
PC                      1.00
```

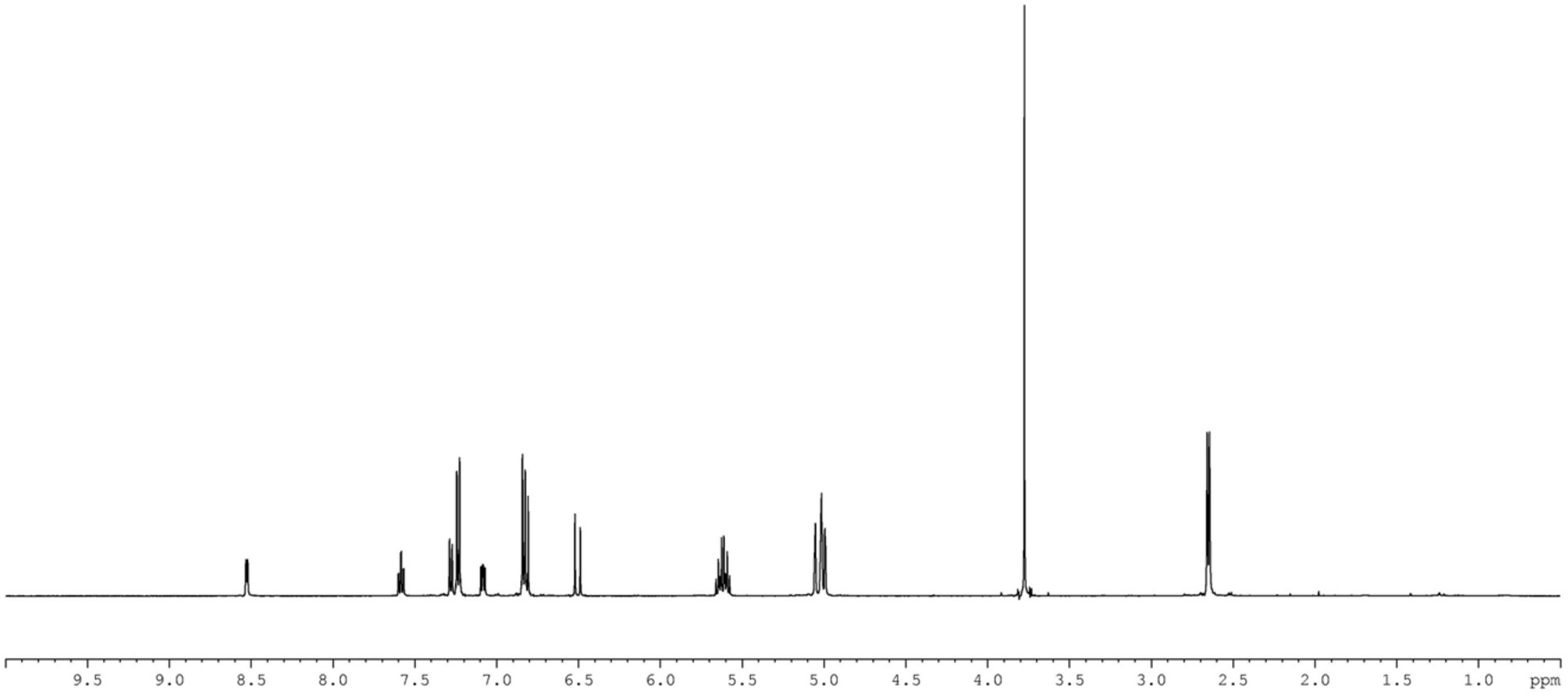

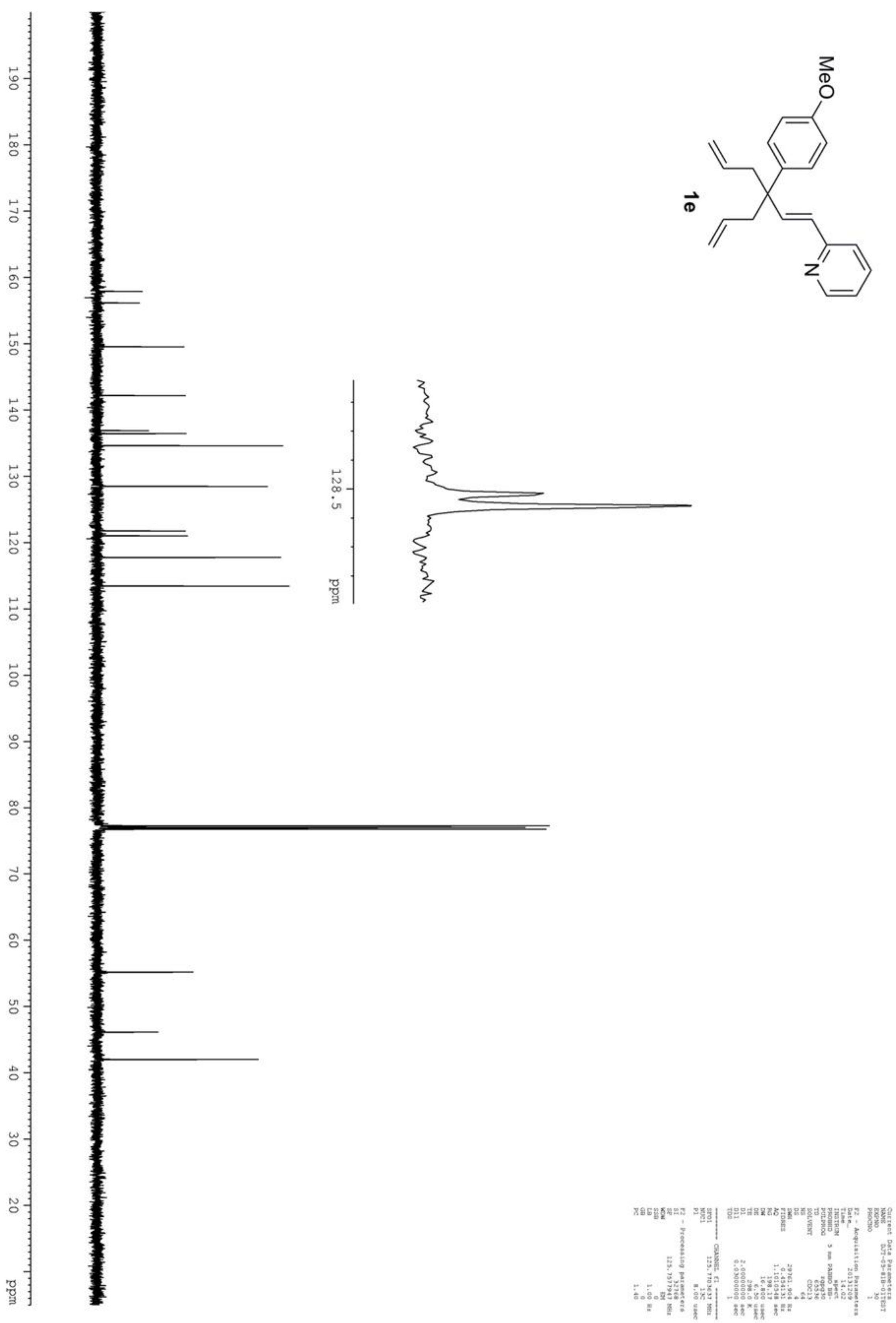
MeO
N
1e
128.5
ppm

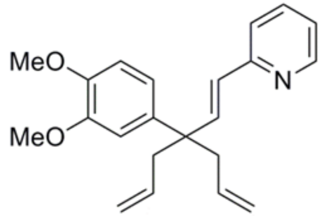

1f

```
Current Data Parameters
NAME     DJT-07-35-01TEST
EXPNO                   2
PROCNO                  1

F2 - Acquisition Parameters
Date_            20131105
Time                13.09
INSTRUM             spect
PROBHD   5 mm PABBO BB-
PULPROG              zg30
TD                  65536
SOLVENT             CDCl3
NS                     32
DS                      2
SWH             10000.000 Hz
FIDRES           0.152588 Hz
AQ              3.2768500 sec
RG                 198.17
DW                 50.000 usec
DE                   6.50 usec
TE                  298.0 K
D1            1.00000000 sec
TD0                     1

======== CHANNEL f1 ========
SFO1          500.1330885 MHz
NUC1                   1H
P1                  10.00 usec

F2 - Processing parameters
SI                  65536
SF            500.1300226 MHz
WDW                    EM
SSB                     0
LB                   0.30 Hz
GB                      0
PC                   1.00
```

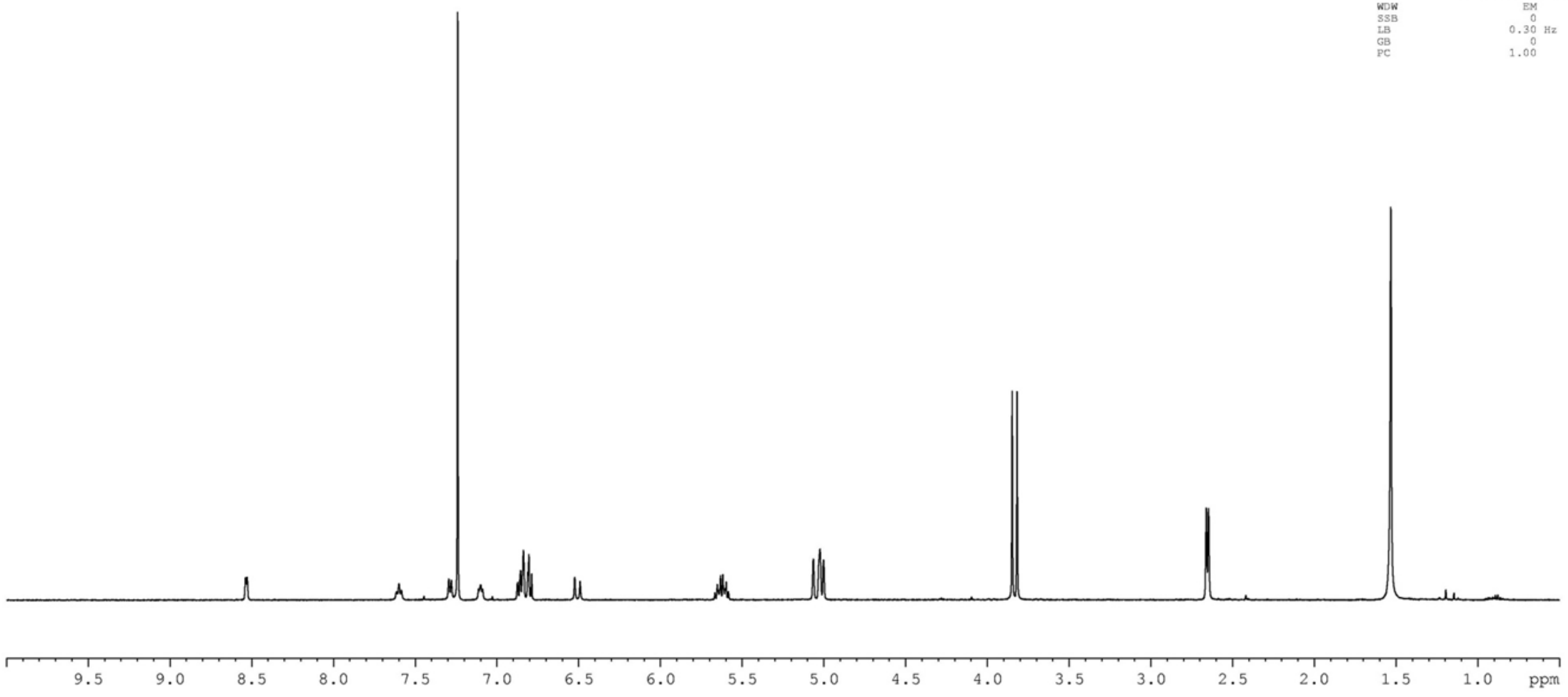

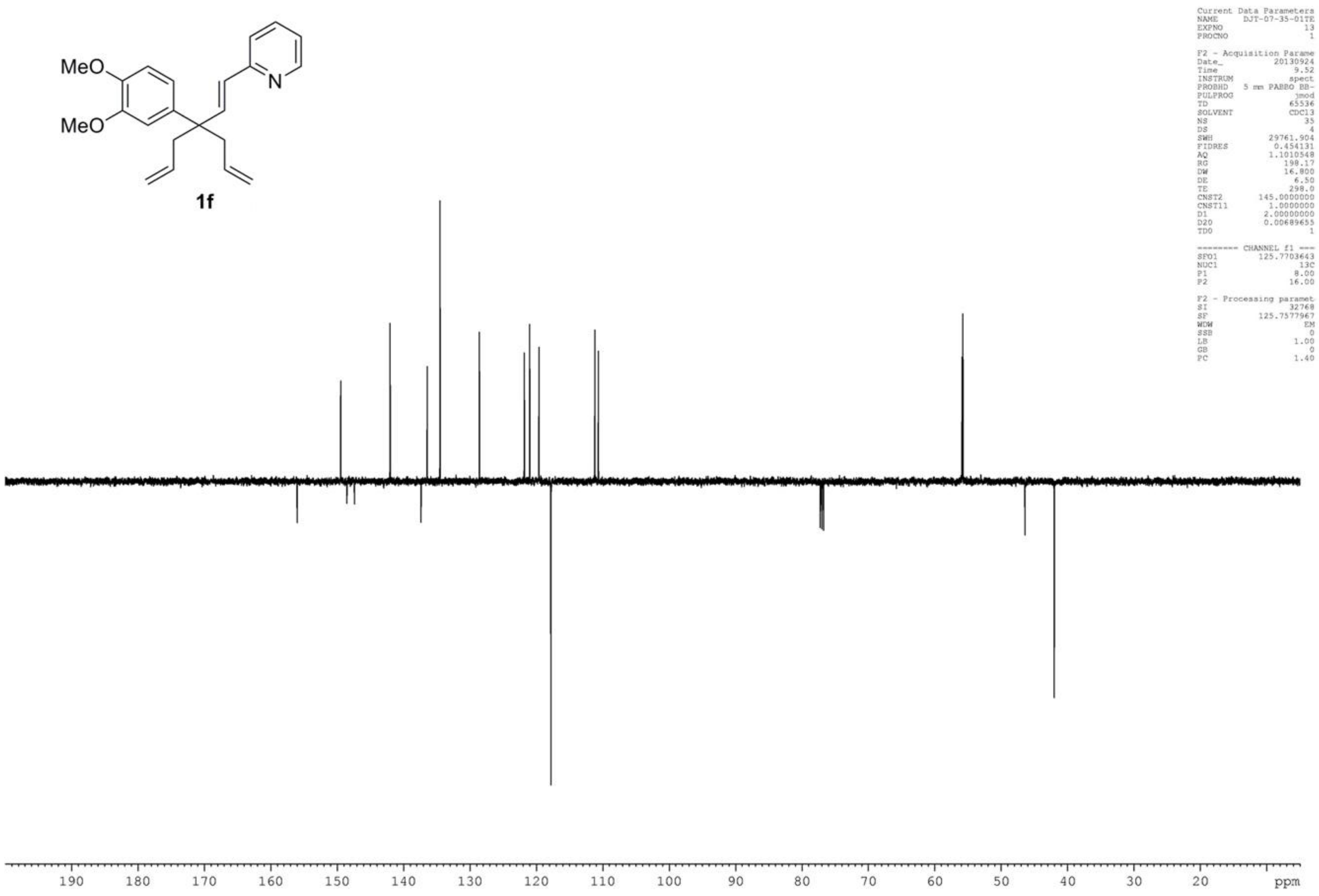
MeO
MeO
N
1f
Current Data Parameters
NAME DJT-07-35-01TE
EXPNO 13
PROCNO 1
F2 - Acquisition Parame
Date_ 20130924
Time 9.52
INSTRUM spect
PROBHD 5 mm PABBO BB-
PULPROG jmod
TD 65536
SOLVENT CDCl3
NS 35
DS 4
SWH 29761.904
FIDRES 0.454131
AQ 1.1010548
RG 198.17
DW 16.800
DE 6.50
TE 298.0
CNST2 145.0000000
CNST11 1.0000000
D1 2.00000000
D20 0.00689655
TD0 1
======== CHANNEL f1 ===
SFO1 125.7703643
NUC1 13C
P1 8.00
P2 16.00
F2 - Processing paramet
SI 32768
SF 125.7577967
WDW EM
SSB 0
LB 1.00
GB 0
PC 1.40
190 180 170 160 150 140 130 120 110 100 90 80 70 60 50 40 30 20 ppm

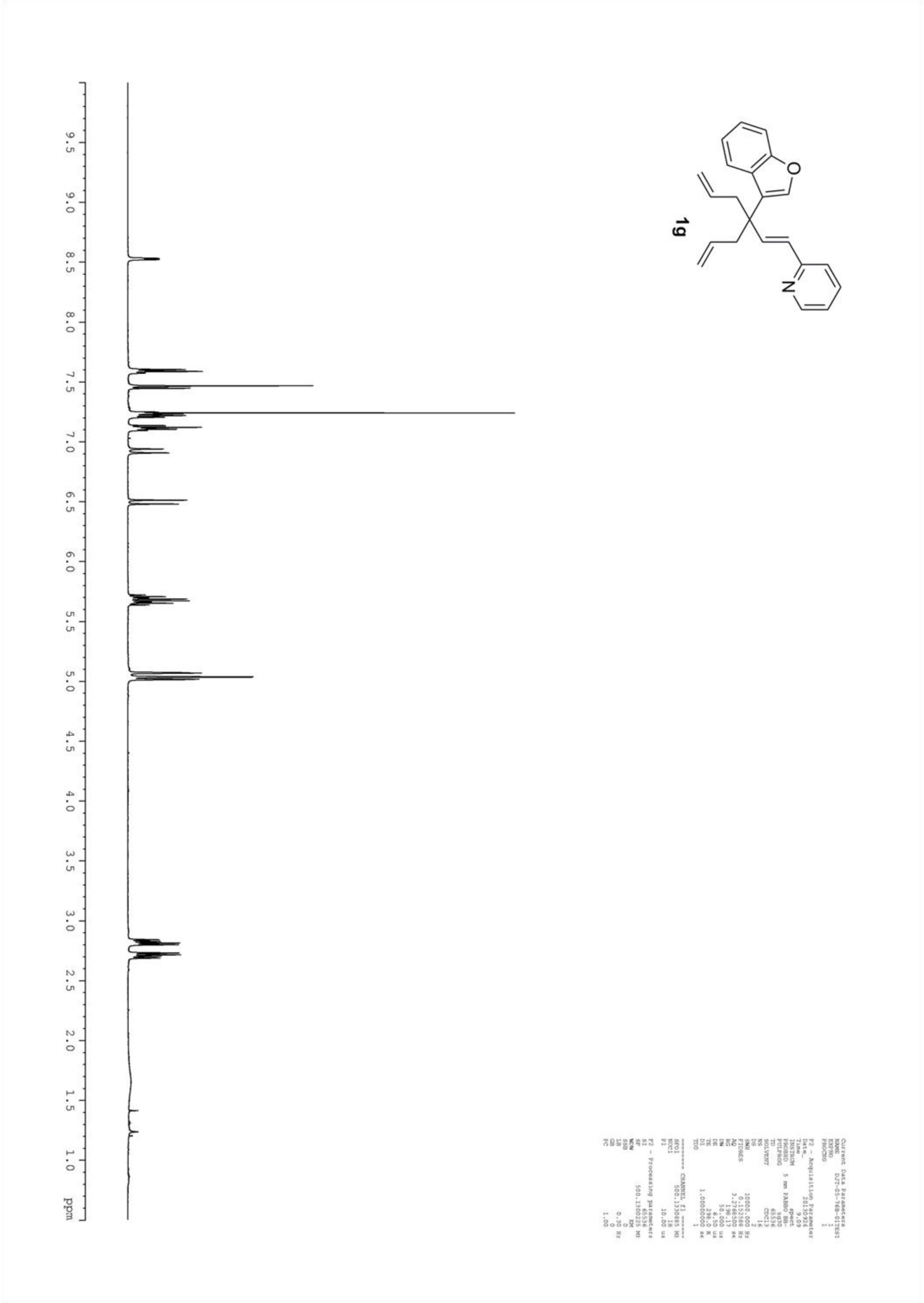
1g
9.5
9.0
8.5
8.0
7.5
7.0
6.5
6.0
5.5
5.0
4.5
4.0
3.5
3.0
2.5
2.0
1.5
1.0
ppm

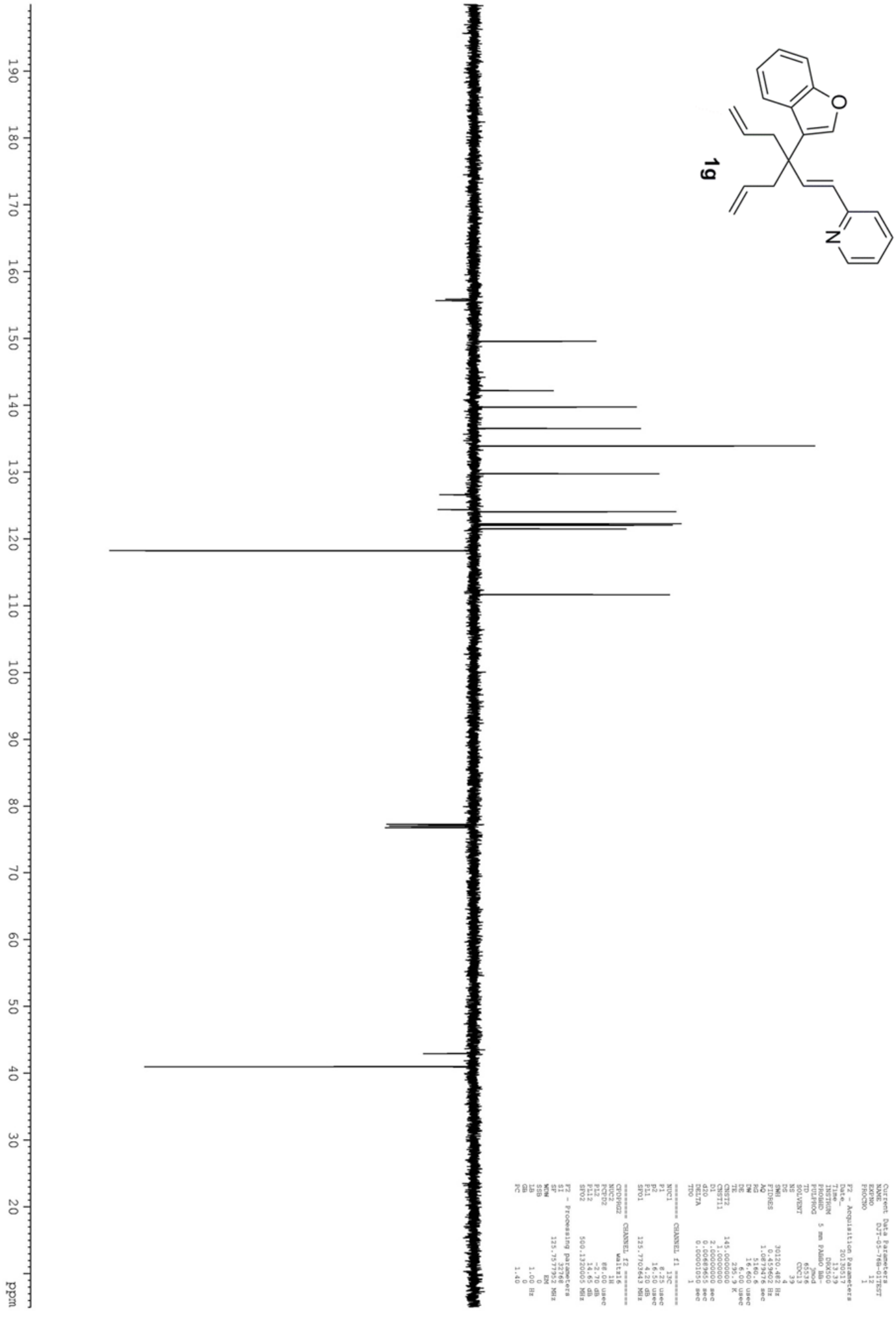

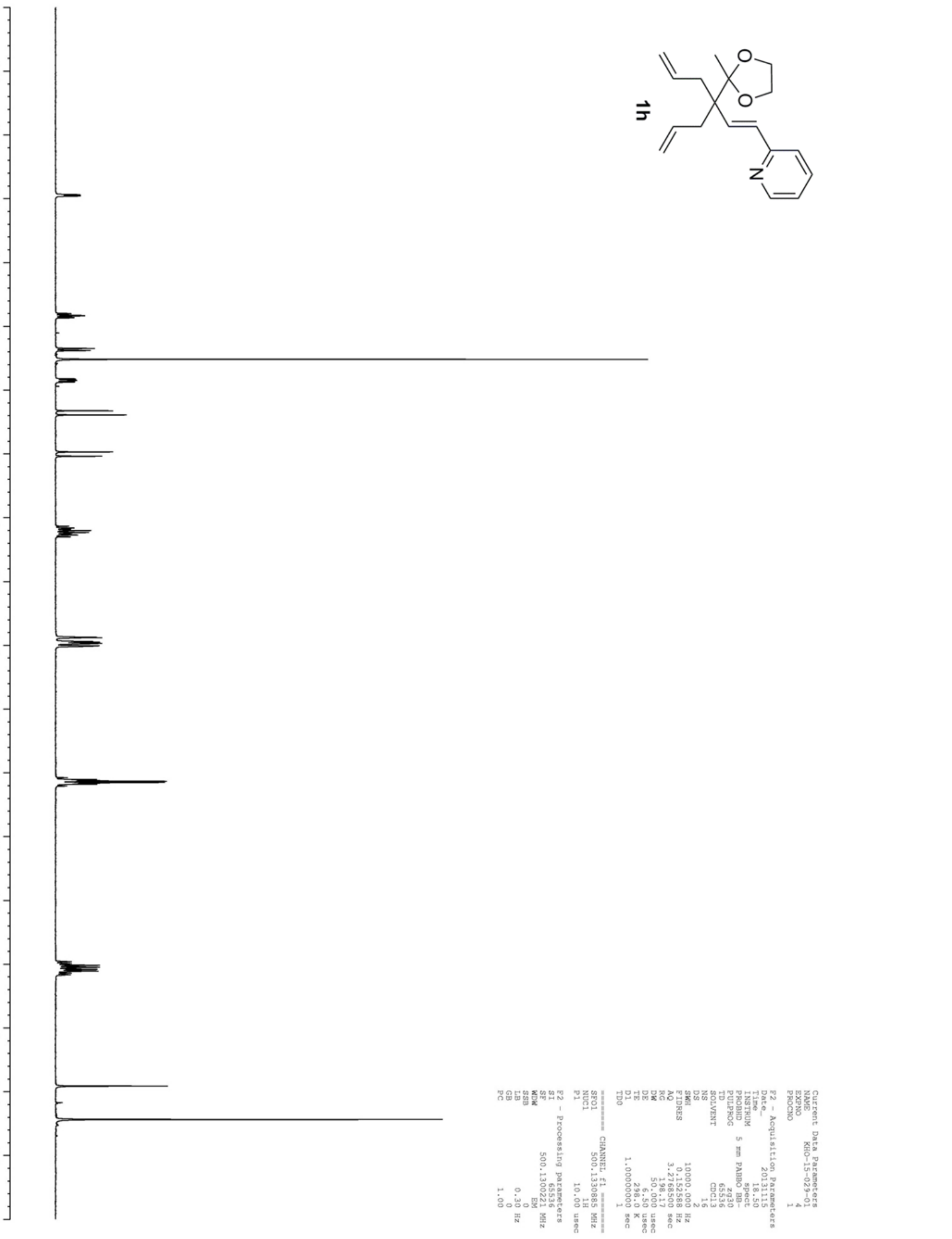
1h
Current Data Parameters
NAME KHO-15-029-01
EXPNO 4
PROCNO 1
F2 - Acquisition Parameters
Date_ 20131115
Time 18.50
INSTRUM spect
PROBHD 5 mm PABBO BB-
PULPROG zg30
TD 65536
SOLVENT CDCl3
NS 16
DS 2
SWH 10000.000 Hz
FIDRES 0.152588 Hz
AQ 3.2768500 sec
RG 198.17
DW 50.000 usec
DE 6.50 usec
TE 298.0 K
D1 1.00000000 sec
TD0 1
======== CHANNEL f1 ========
SFO1 500.1330885 MHz
NUC1 1H
P1 10.00 usec
F2 - Processing parameters
SI 65536
SF 500.1300221 MHz
WDW EM
SSB 0
LB 0.30 Hz
GB 0
PC 1.00
9.5 9.0 8.5 8.0 7.5 7.0 6.5 6.0 5.5 5.0 4.5 4.0 3.5 3.0 2.5 2.0 1.5 1.0 ppm

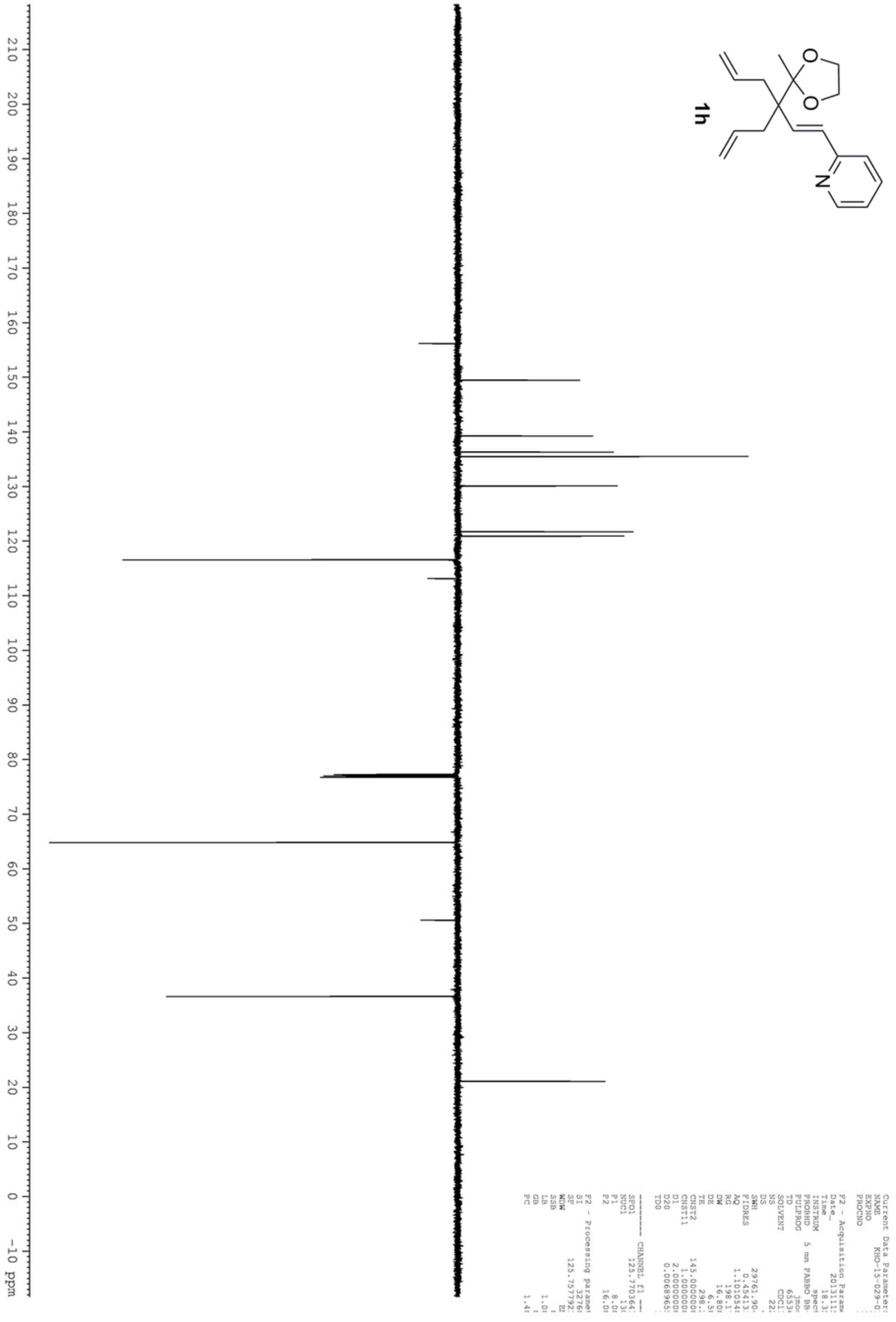
1h
ppm

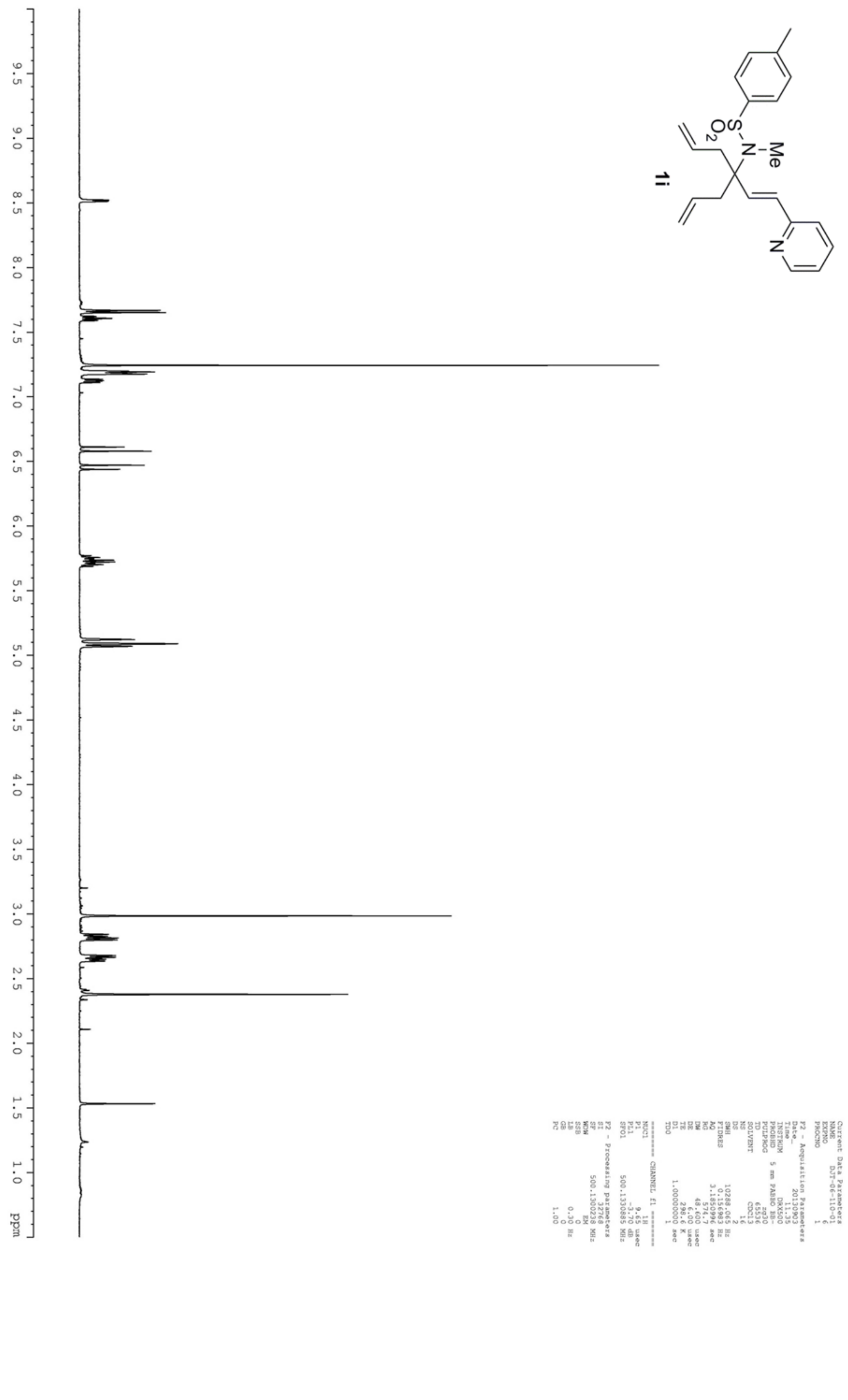

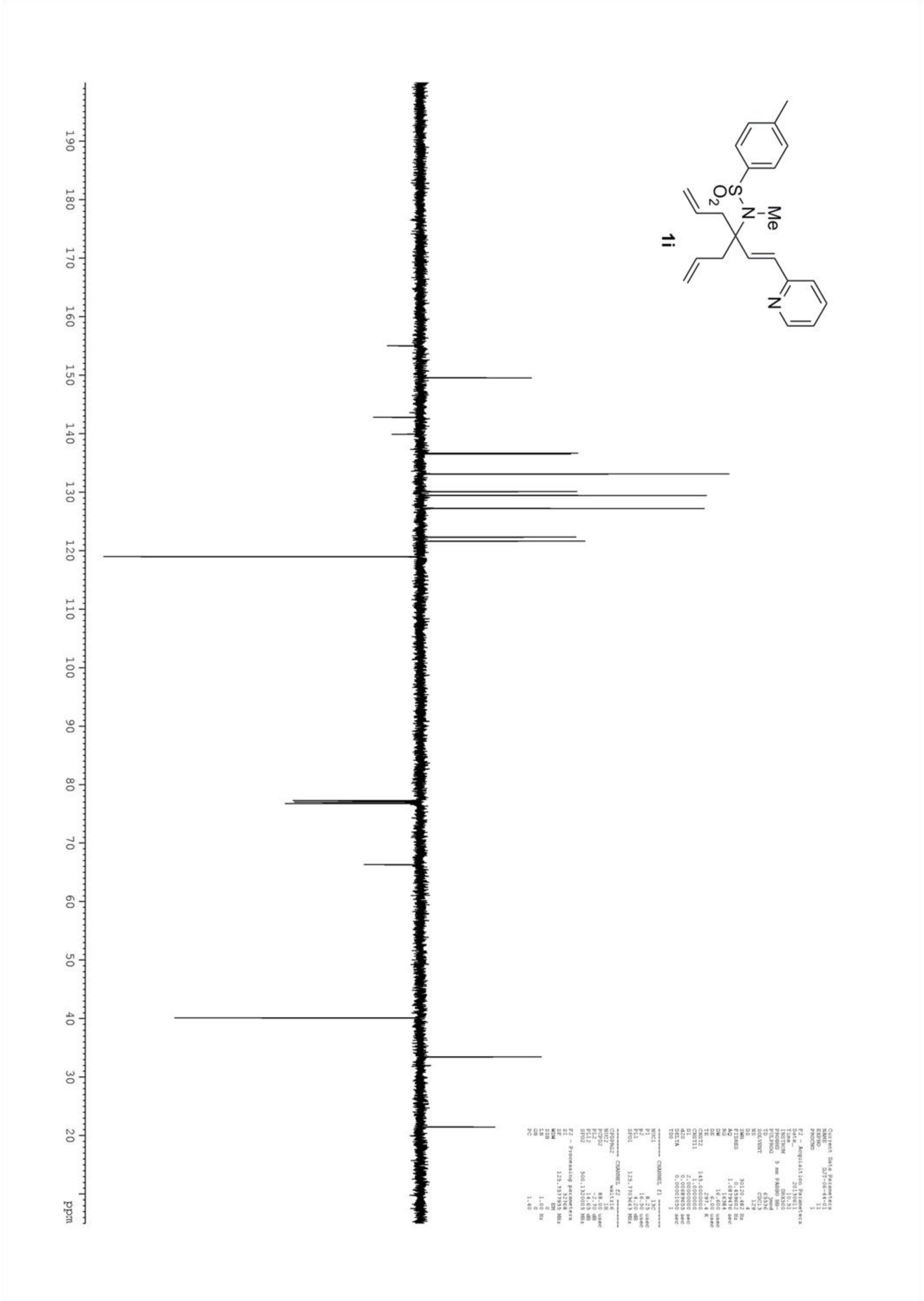

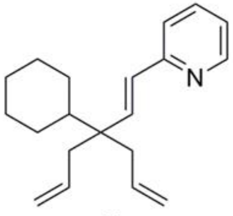

1j

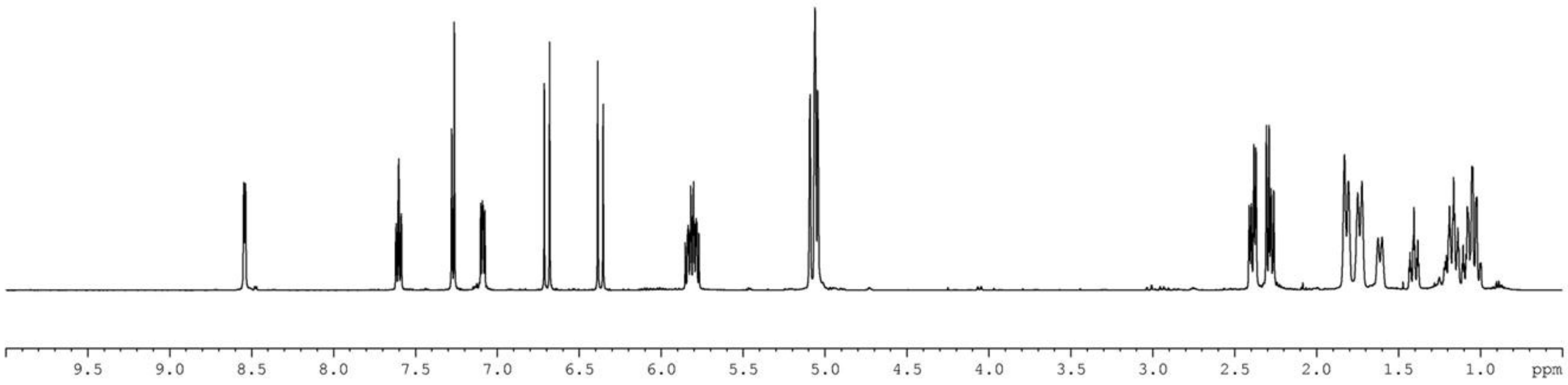
Current Data Parameters
NAME DJT-06-34-01
EXPNO 2
PROCNO 1
F2 - Acquisition Paramete
Date_ 20130603
Time 10.06
INSTRUM DRX500
PROBHD 5 mm PABBO BB-
PULPROG zg30
TD 65536
SOLVENT CDCl3
NS 8
DS 2
SWH 10288.065 H
FIDRES 0.156983 H
AQ 3.1850996 s
RG 80.6
DW 48.600 u
DE 6.00 u
TE 297.1 K
D1 1.00000000 s
TD0 1
======== CHANNEL f1 =====
NUC1 1H
P1 9.65 u
PL1 -3.70 d
SFO1 500.1330885 M
F2 - Processing parameter
SI 32768
SF 500.1305114 M
WDW EM
SSB 0
LB 0.30 H
GB 0
PC 1.00
9.5
9.0
8.5
8.0
7.5
7.0
6.5
6.0
5.5
5.0
4.5
4.0
3.5
3.0
2.5
2.0
1.5
1.0
ppm

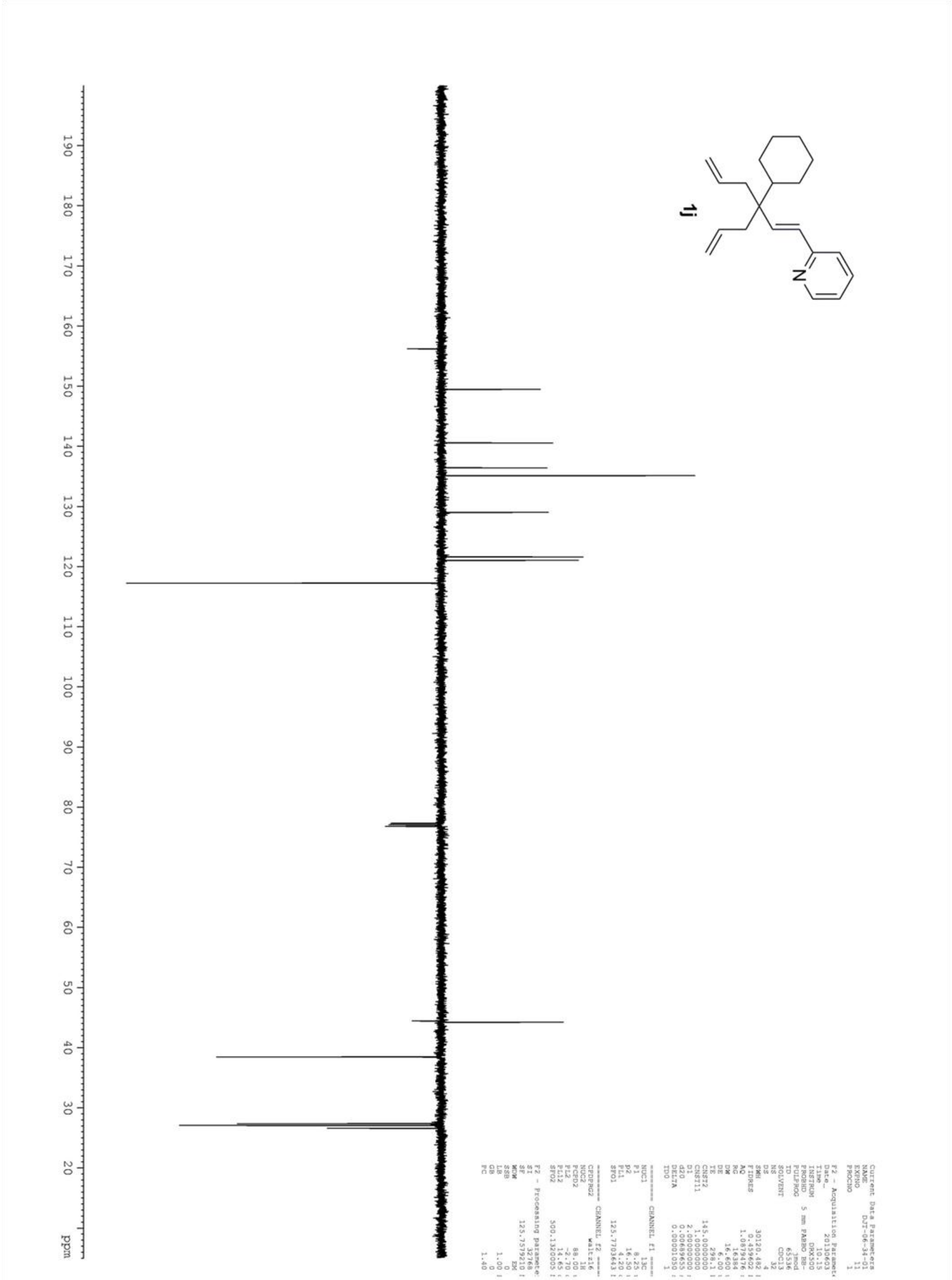
1j
ppm

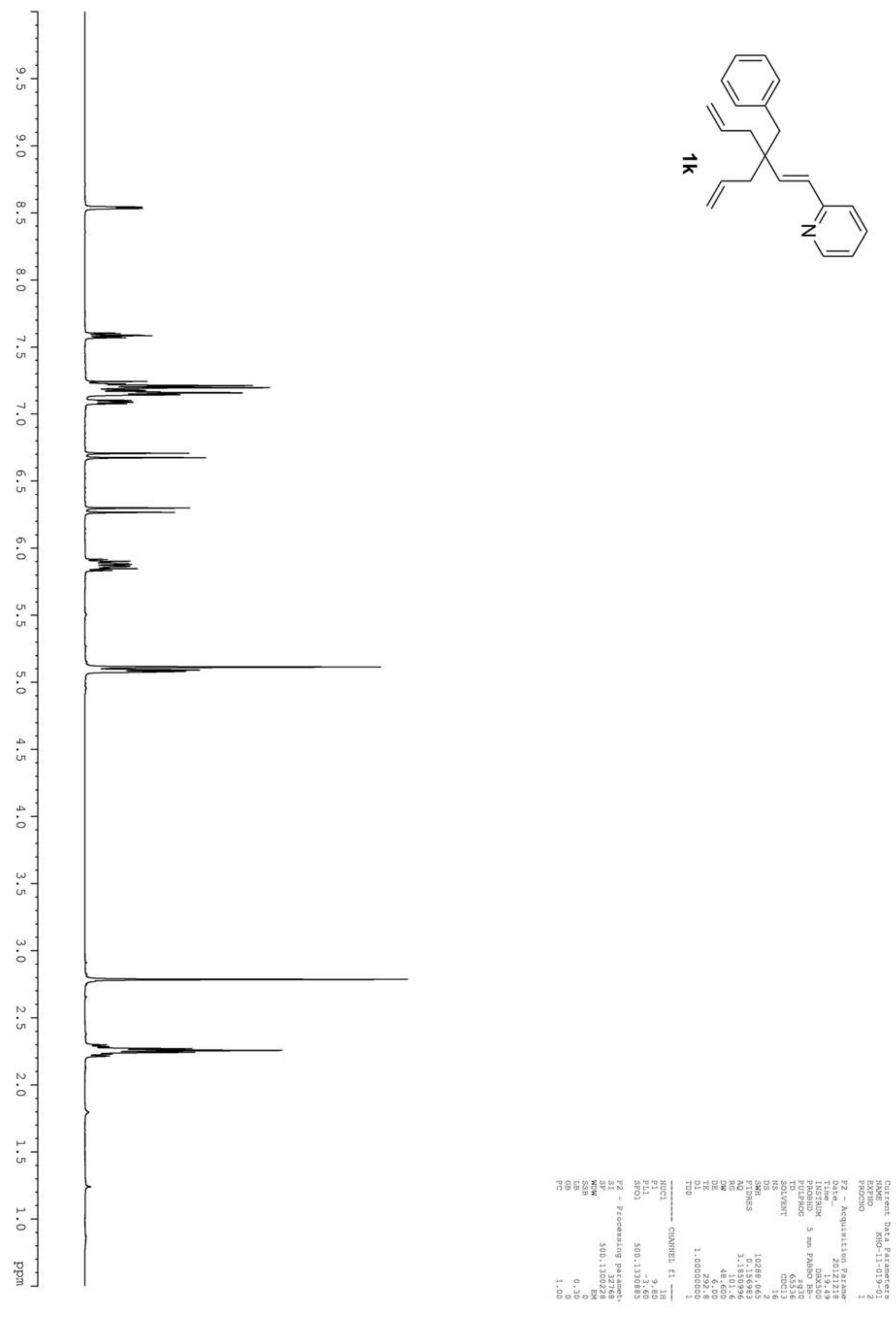

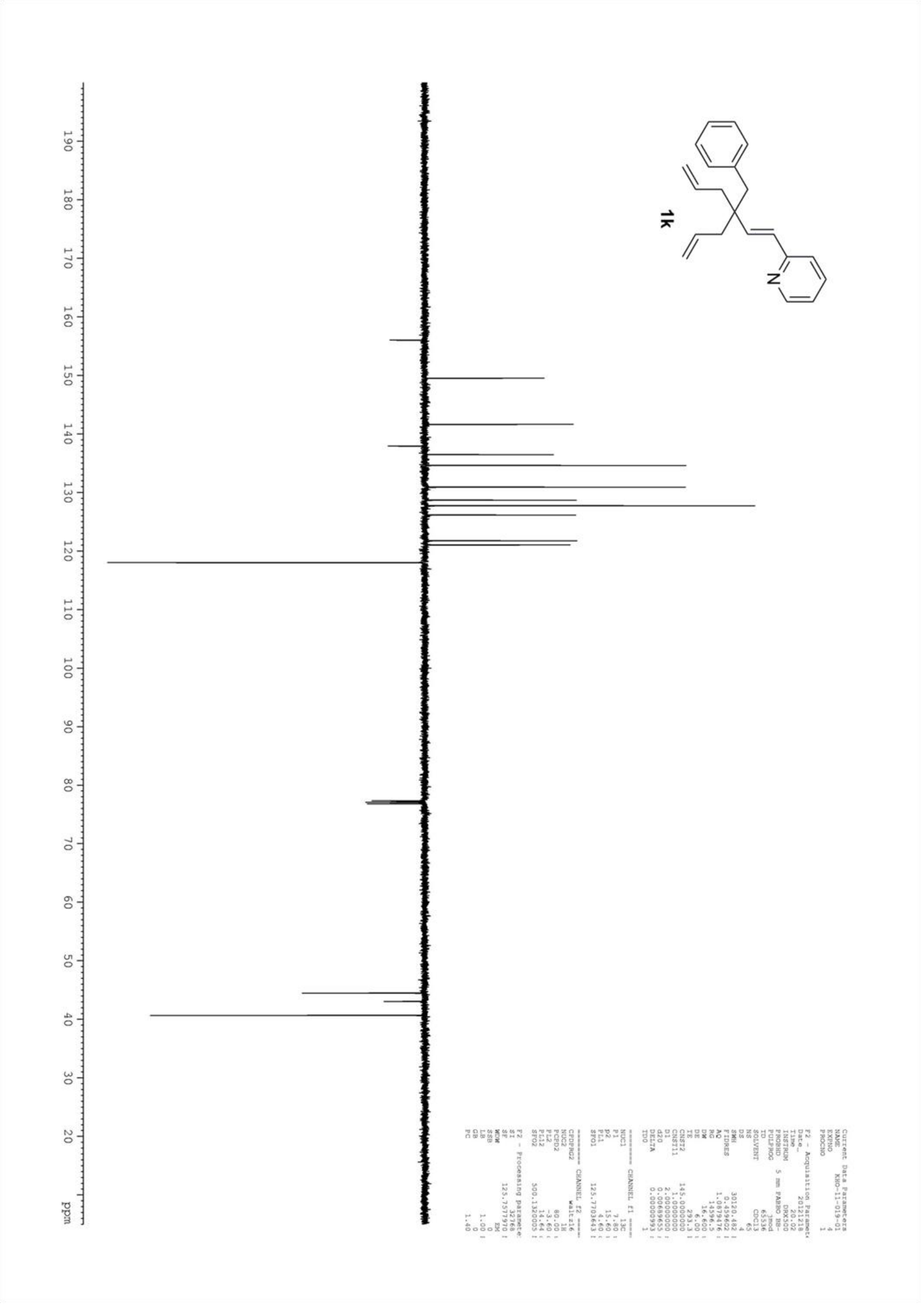
1k

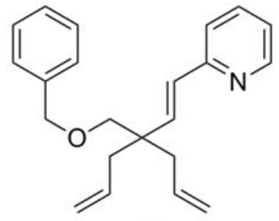

1l

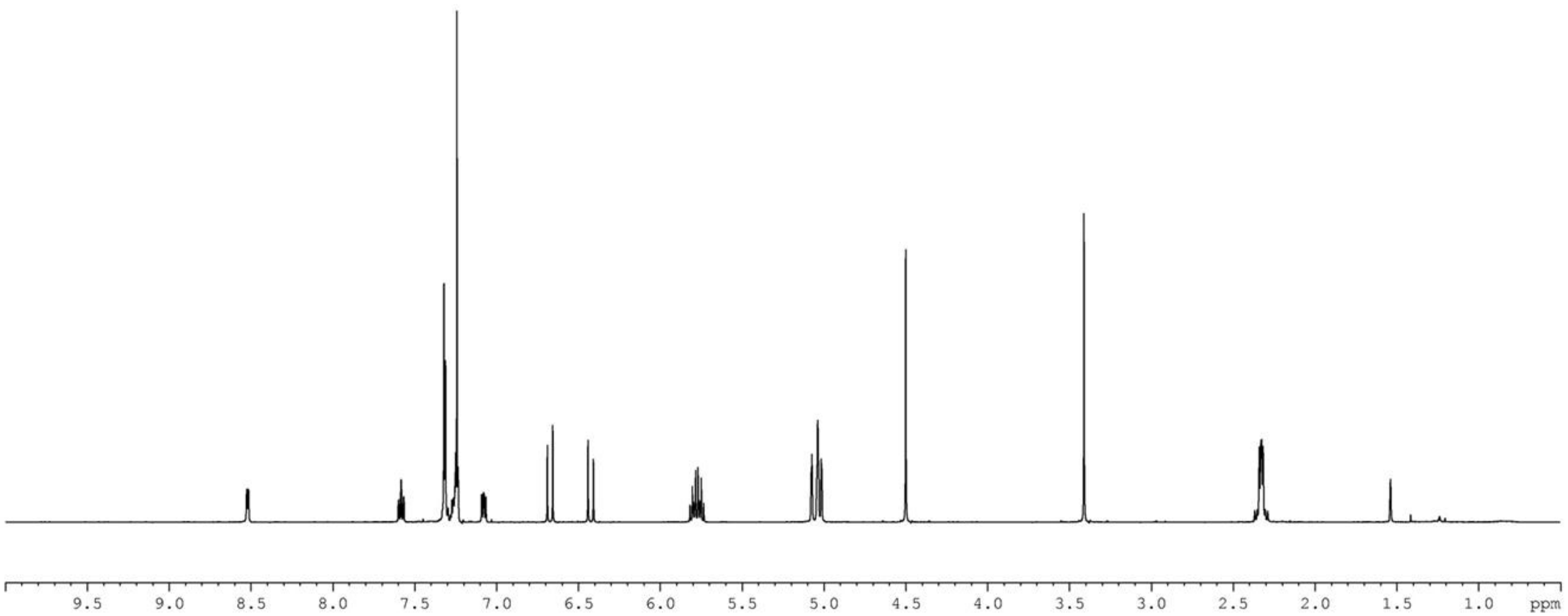

Current Data Paramete
NAME KHO-13-034-
EXPNO
PROCNO
F2 - Acquisition Para
Date_ 201306
Time 18.
INSTRUM DRX5
PROBHD 5 mm PABBO B
PULPROG zg
TD 655
SOLVENT CDC
NS
DS
SWH 10288.0
FIDRES 0.1569
AQ 3.18509
RG 3
DW 48.6
DE 6.
TE 299
D1 1.000000
TD0
======== CHANNEL f1 ==
NUC1
P1 9.
PL1 -3.
SFO1 500.13308
F2 - Processing param
SI 327
SF 500.13002
WDW
SSB
LB 0.
GB
PC 1.
9.5
9.0
8.5
8.0
7.5
7.0
6.5
6.0
5.5
5.0
4.5
4.0
3.5
3.0
2.5
2.0
1.5
1.0
ppm

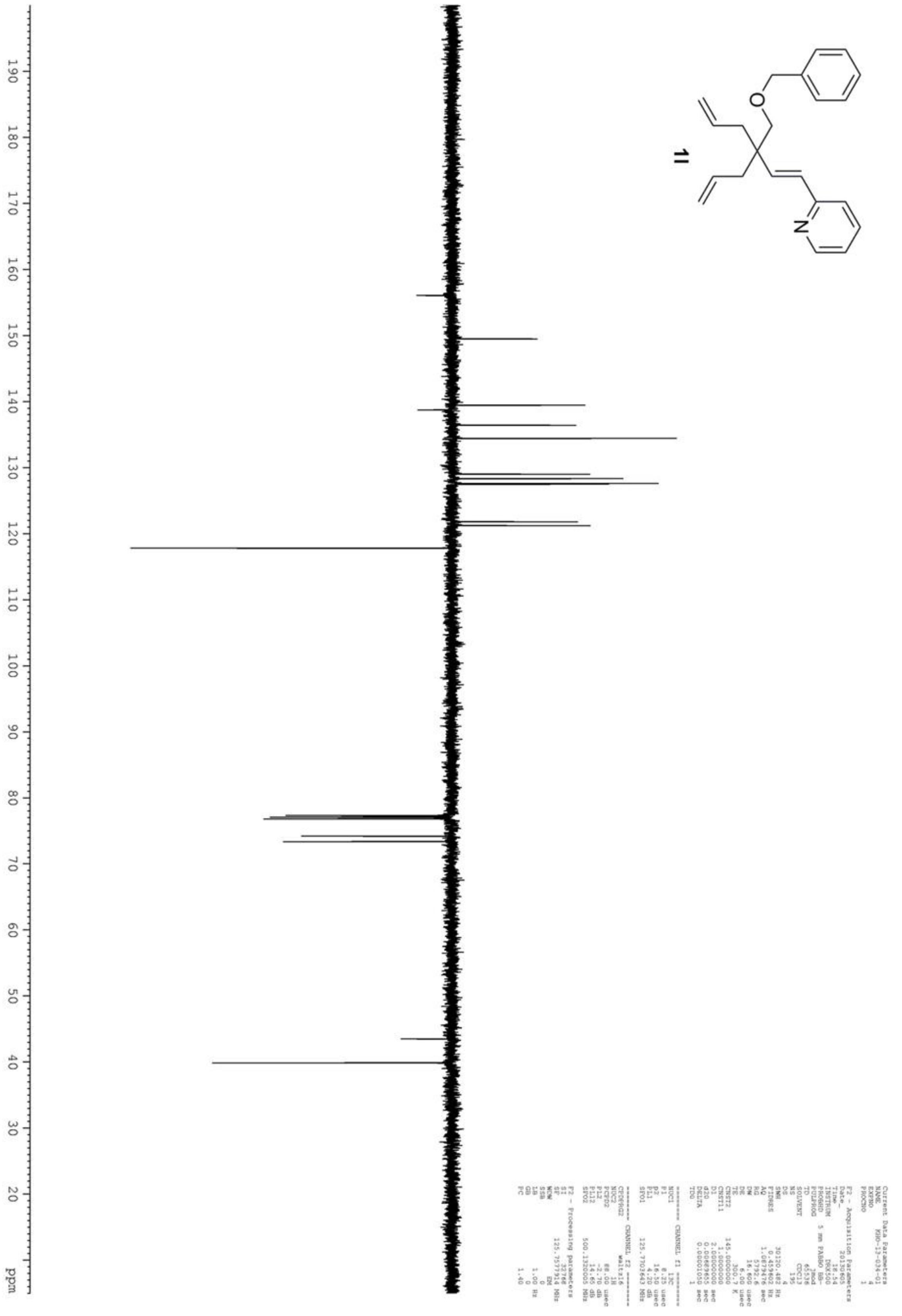

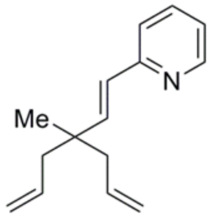

1m

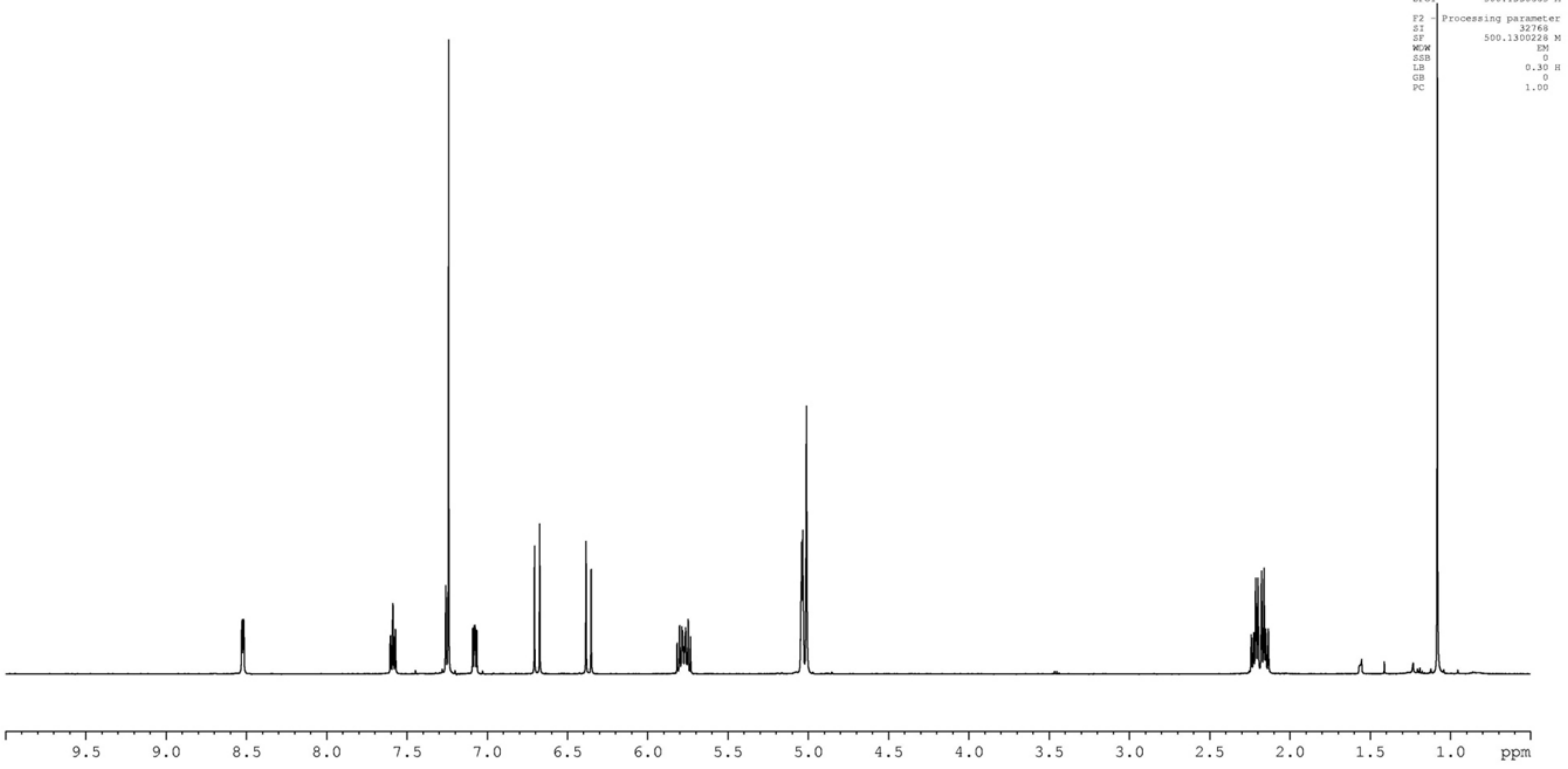
Current Data Parameters
NAME KHO-11-104-01
EXPNO 12
PROCNO 1
F2 - Acquisition Paramete
Date_ 20130218
Time 19.41
INSTRUM DRX500
PROBHD 5 mm PABBO BB-
PULPROG zg30
TD 65536
SOLVENT CDCl3
NS 16
DS 2
SWH 10288.065 H
FIDRES 0.156983 H
AQ 3.1850996 s
RG 362
DW 48.600 u
DE 6.00 u
TE 293.8 K
D1 5.00000000 s
TD0 1
======== CHANNEL f1 =====
NUC1 1H
P1 9.80 u
PL1 -3.60 d
SFO1 500.1330885 M
F2 - Processing parameter
SI 32768
SF 500.1300228 M
WDW EM
SSB 0
LB 0.30 H
GB 0
PC 1.00
9.5
9.0
8.5
8.0
7.5
7.0
6.5
6.0
5.5
5.0
4.5
4.0
3.5
3.0
2.5
2.0
1.5
1.0
ppm

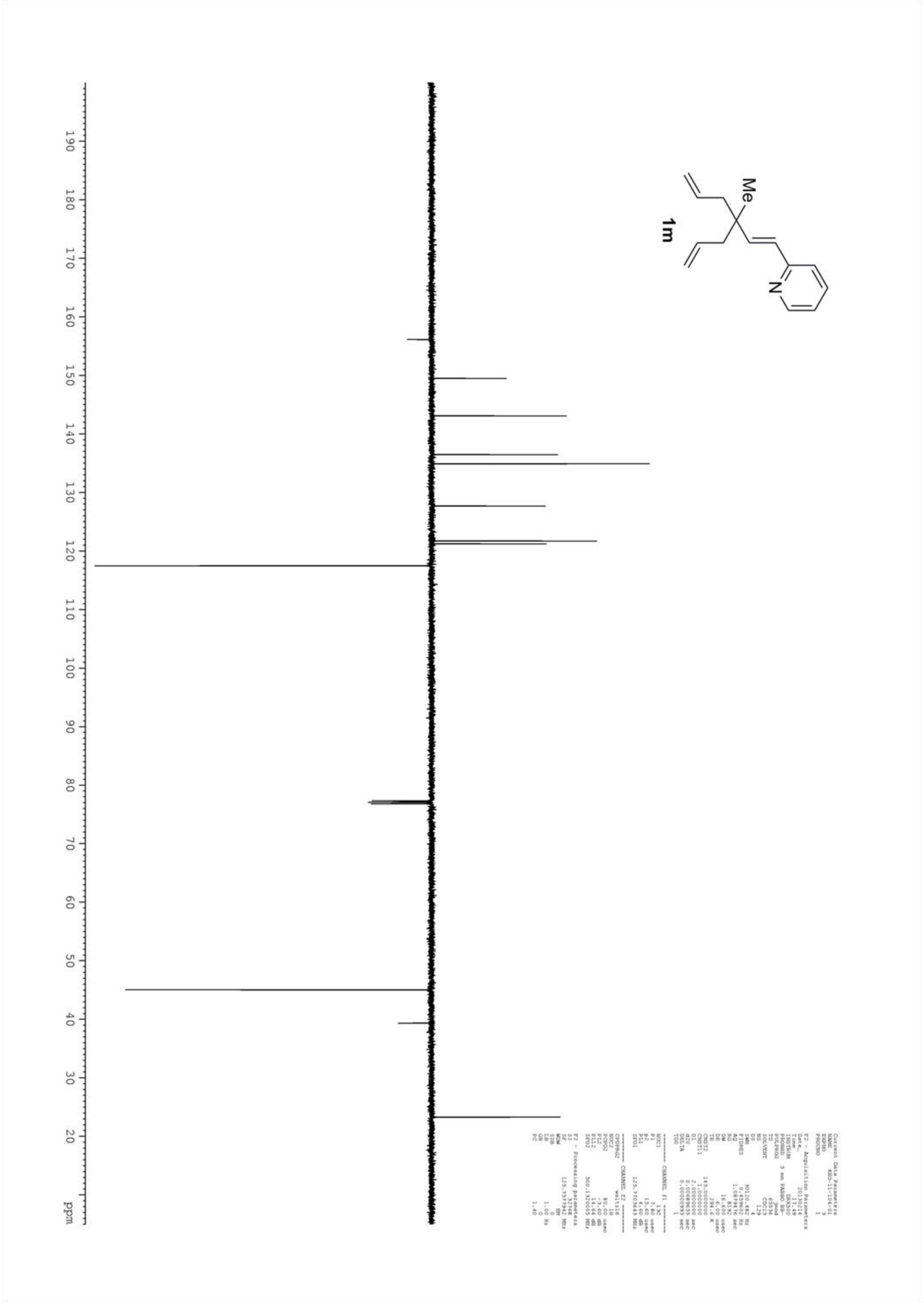
1m
Me
N
ppm

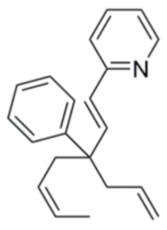

4a

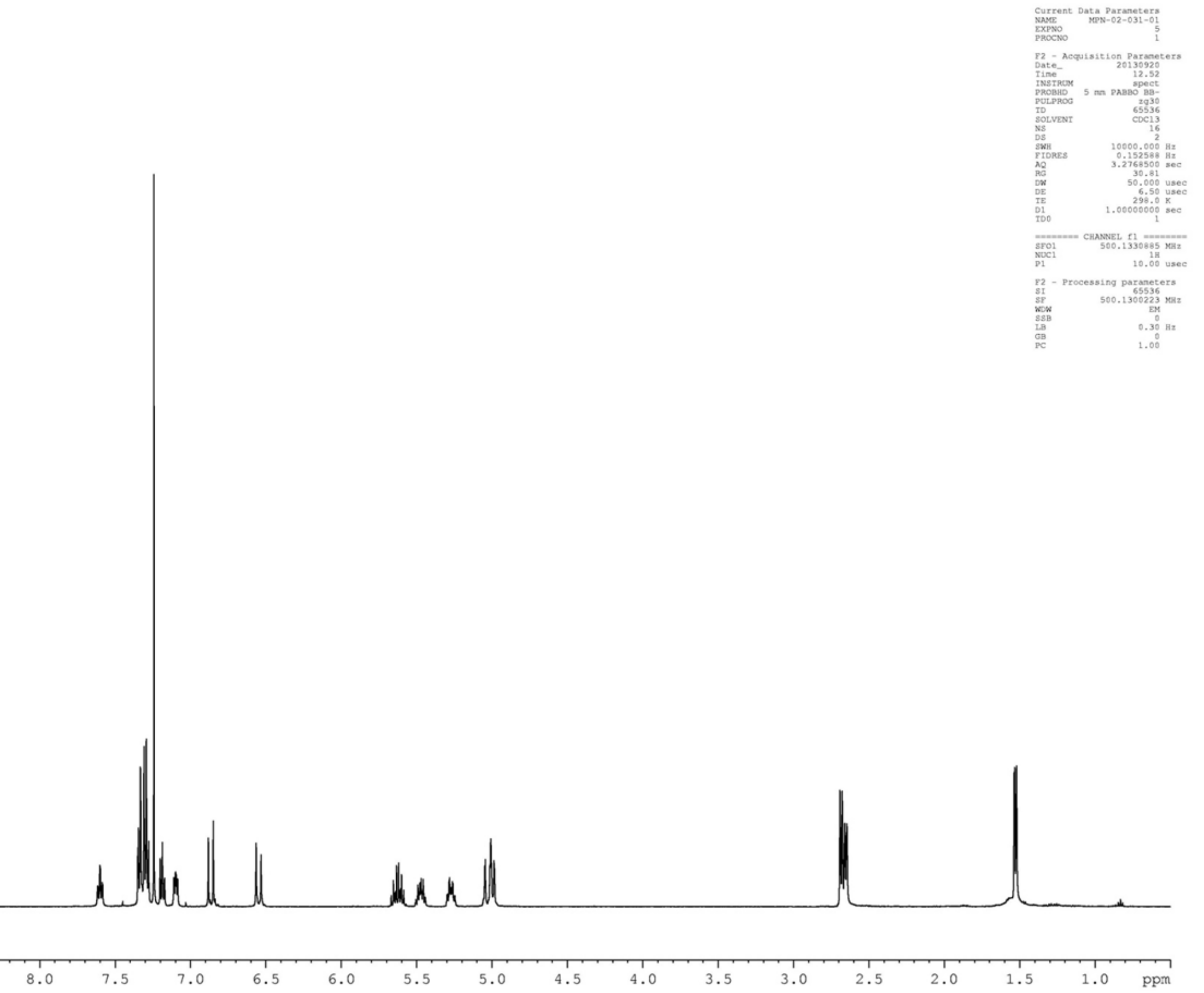
Current Data Parameters
NAME MPN-02-031-01
EXPNO 5
PROCNO 1
F2 - Acquisition Parameters
Date_ 20130920
Time 12.52
INSTRUM spect
PROBHD 5 mm PABBO BB-
PULPROG zg30
TD 65536
SOLVENT CDCl3
NS 16
DS 2
SWH 10000.000 Hz
FIDRES 0.152588 Hz
AQ 3.2768500 sec
RG 30.81
DW 50.000 usec
DE 6.50 usec
TE 298.0 K
D1 1.00000000 sec
TD0 1
======== CHANNEL f1 ========
SFO1 500.1330885 MHz
NUC1 1H
P1 10.00 usec
F2 - Processing parameters
SI 65536
SF 500.1300223 MHz
WDW EM
SSB 0
LB 0.30 Hz
GB 0
PC 1.00
9.5 9.0 8.5 8.0 7.5 7.0 6.5 6.0 5.5 5.0 4.5 4.0 3.5 3.0 2.5 2.0 1.5 1.0 ppm

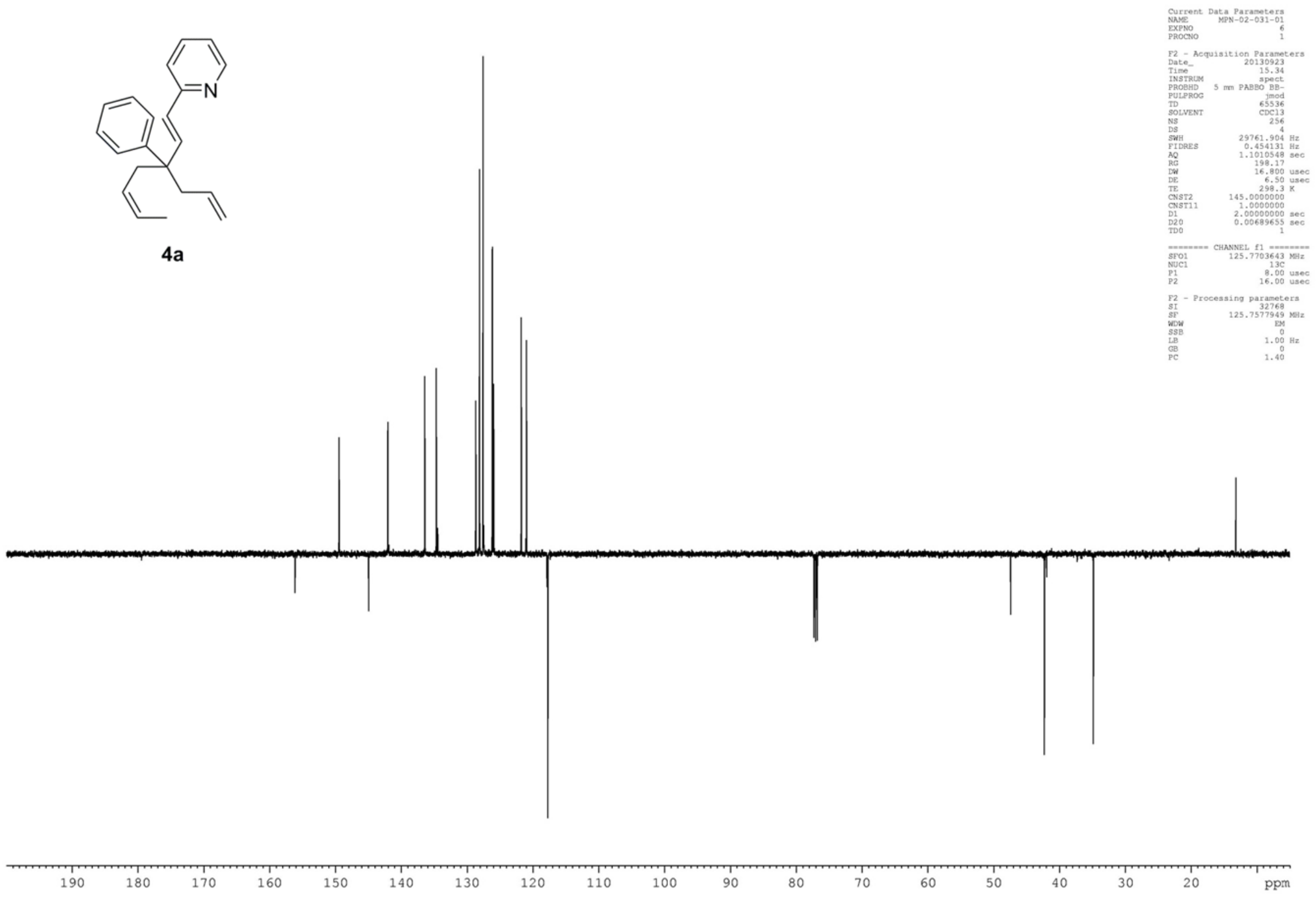
N
4a
Current Data Parameters
NAME MPN-02-031-01
EXPNO 6
PROCNO 1
F2 - Acquisition Parameters
Date_ 20130923
Time 15.34
INSTRUM spect
PROBHD 5 mm PABBO BB-
PULPROG jmod
TD 65536
SOLVENT CDCl3
NS 256
DS 4
SWH 29761.904 Hz
FIDRES 0.454131 Hz
AQ 1.1010548 sec
RG 198.17
DW 16.800 usec
DE 6.50 usec
TE 298.3 K
CNST2 145.0000000
CNST11 1.0000000
D1 2.00000000 sec
D20 0.00689655 sec
TD0 1
======== CHANNEL f1 ========
SFO1 125.7703643 MHz
NUC1 13C
P1 8.00 usec
P2 16.00 usec
F2 - Processing parameters
SI 32768
SF 125.7577949 MHz
WDW EM
SSB 0
LB 1.00 Hz
GB 0
PC 1.40
190
180
170
160
150
140
130
120
110
100
90
80
70
60
50
40
30
20
ppm

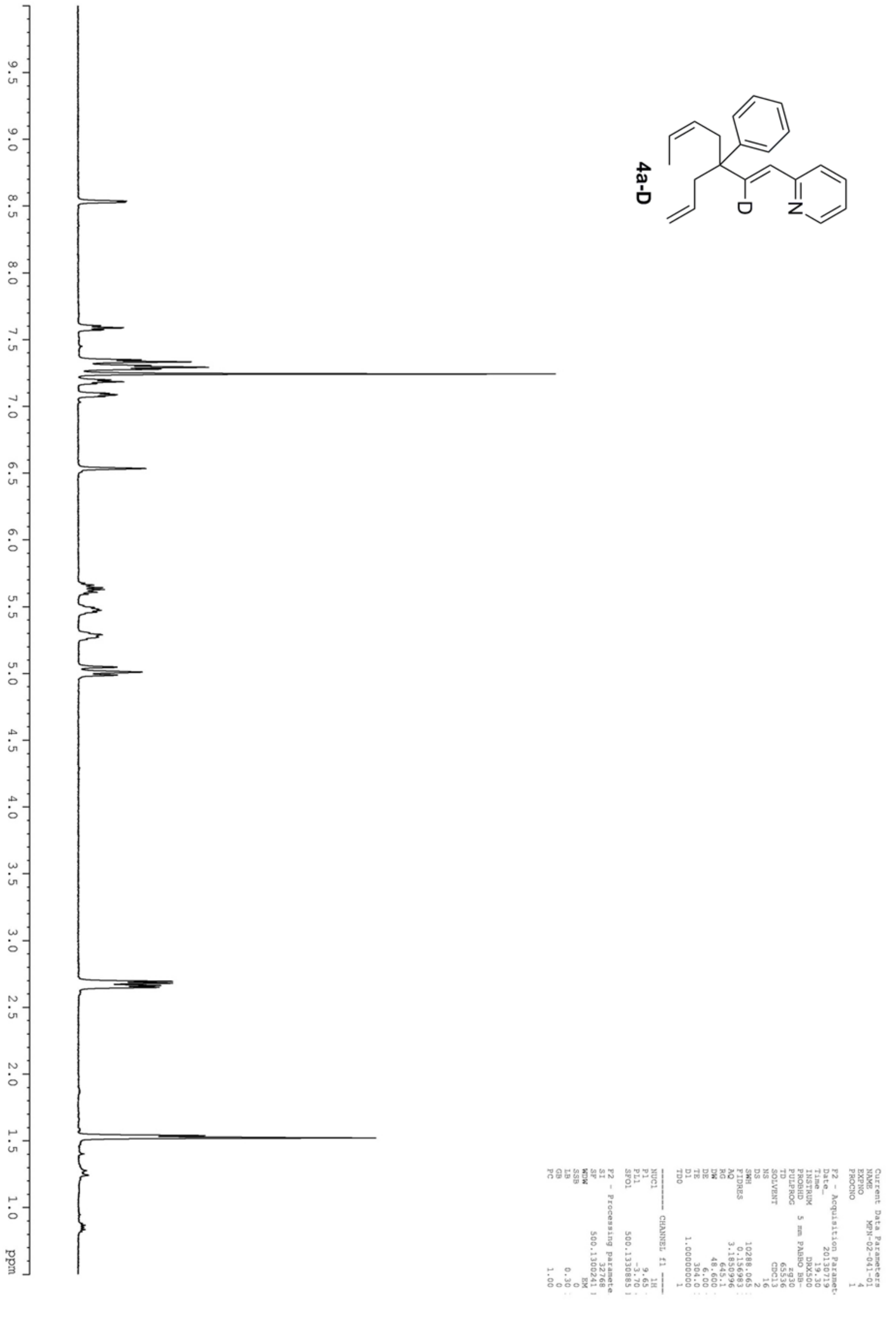
4a-D
ppm
9.5
9.0
8.5
8.0
7.5
7.0
6.5
6.0
5.5
5.0
4.5
4.0
3.5
3.0
2.5
2.0
1.5
1.0

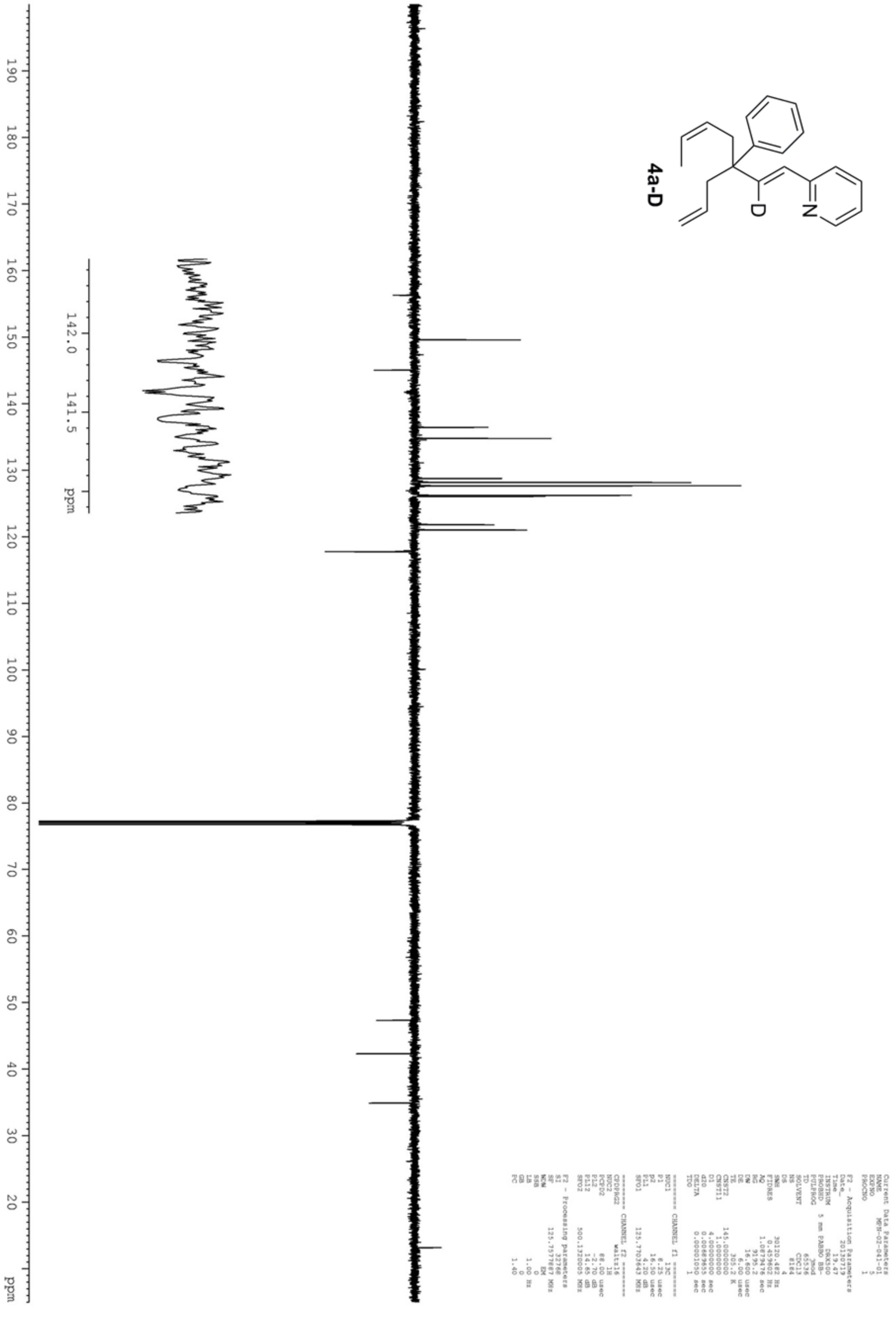
4a-D
142.0
141.5
ppm
ppm

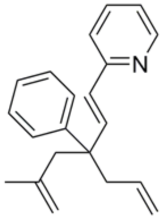

4c

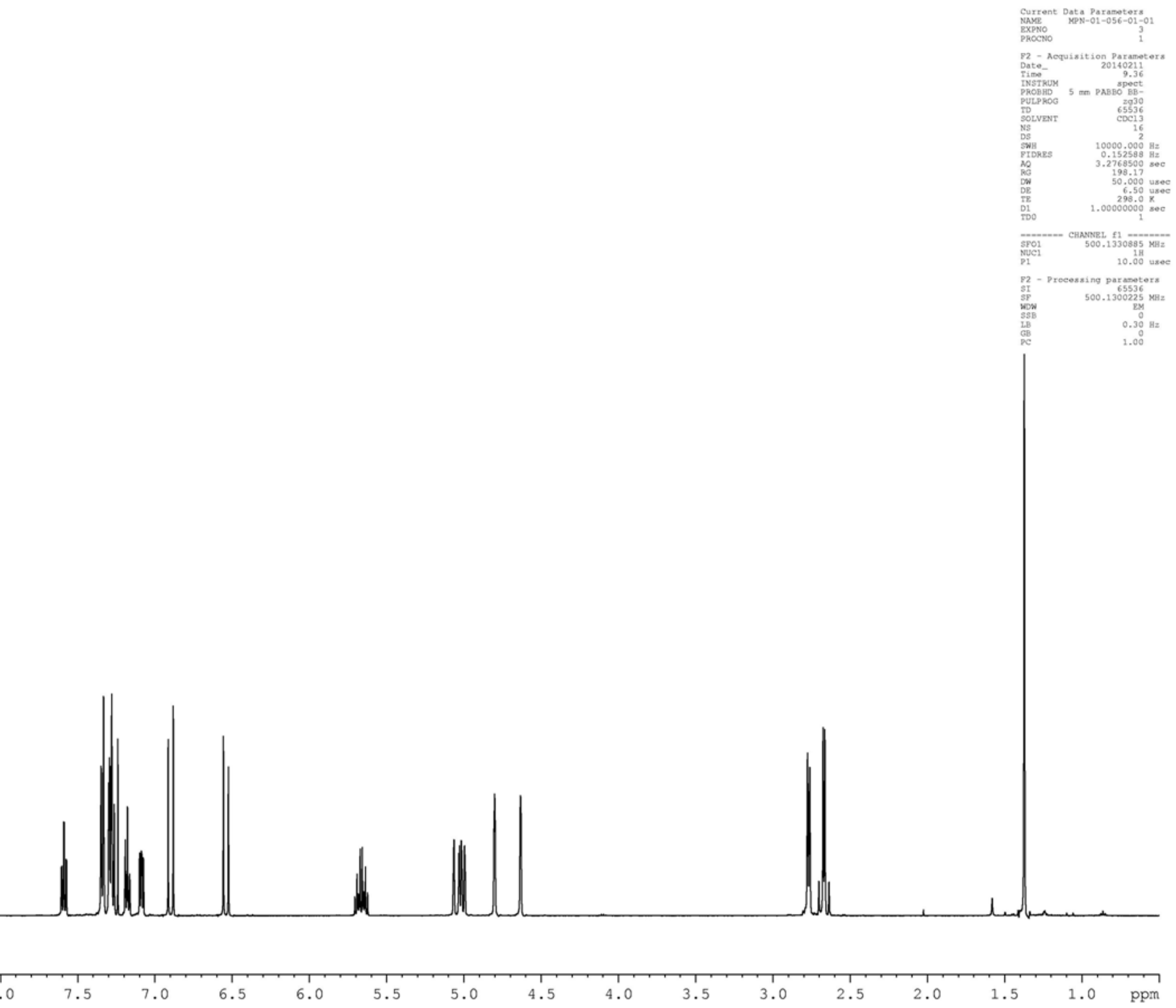

Current Data Parameters
NAME MPN-01-056-01-01
EXPNO 3
PROCNO 1
F2 - Acquisition Parameters
Date_ 20140211
Time 9.36
INSTRUM spect
PROBHD 5 mm PABBO BB-
PULPROG zg30
TD 65536
SOLVENT CDCl3
NS 16
DS 2
SWH 10000.000 Hz
FIDRES 0.152588 Hz
AQ 3.2768500 sec
RG 198.17
DW 50.000 usec
DE 6.50 usec
TE 298.0 K
D1 1.00000000 sec
TD0 1
======== CHANNEL f1 ========
SFO1 500.1330885 MHz
NUC1 1H
P1 10.00 usec
F2 - Processing parameters
SI 65536
SF 500.1300225 MHz
WDW EM
SSB 0
LB 0.30 Hz
GB 0
PC 1.00
9.5 9.0 8.5 8.0 7.5 7.0 6.5 6.0 5.5 5.0 4.5 4.0 3.5 3.0 2.5 2.0 1.5 1.0 ppm

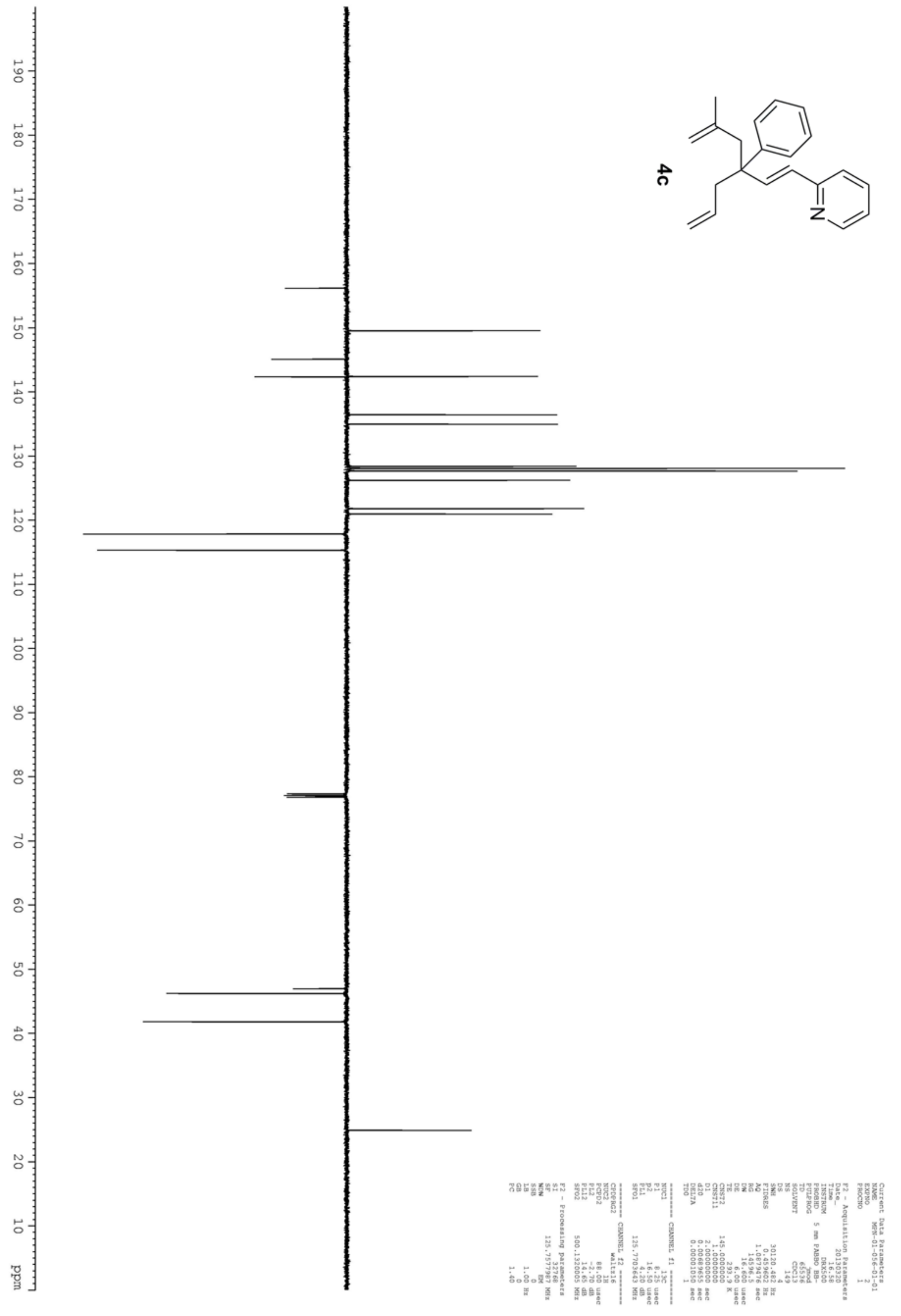
4c

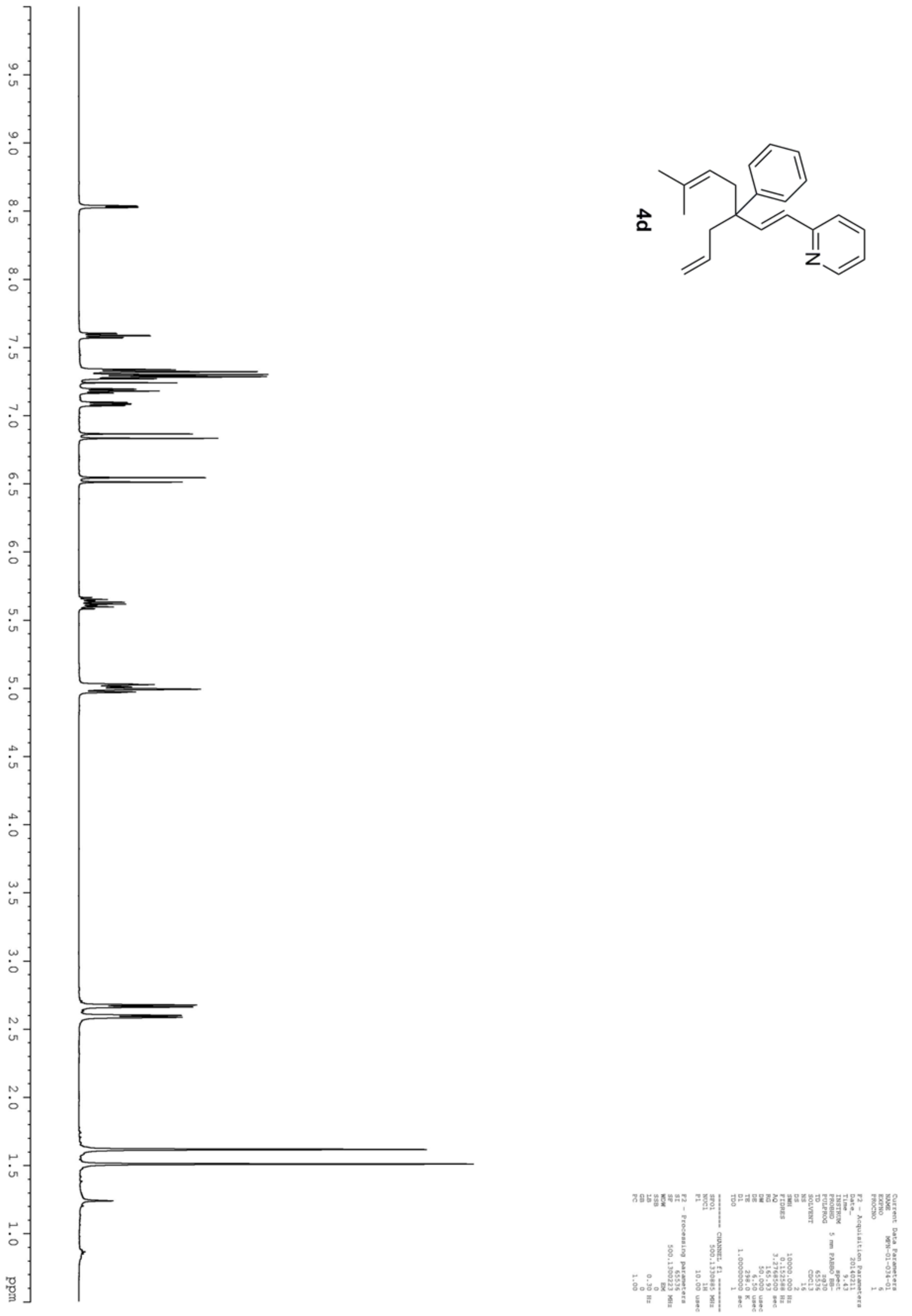
4d
ppm

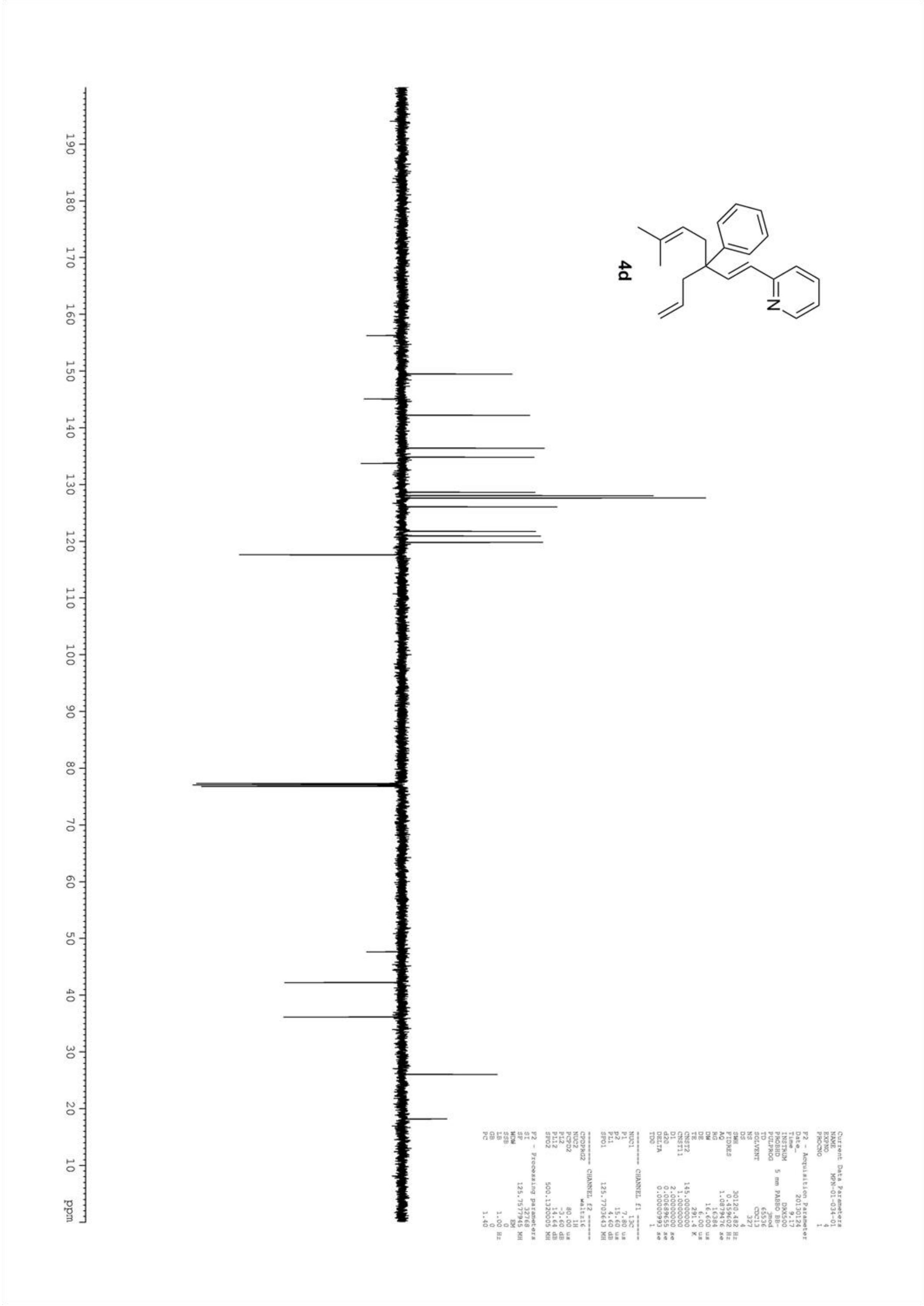
4d
ppm

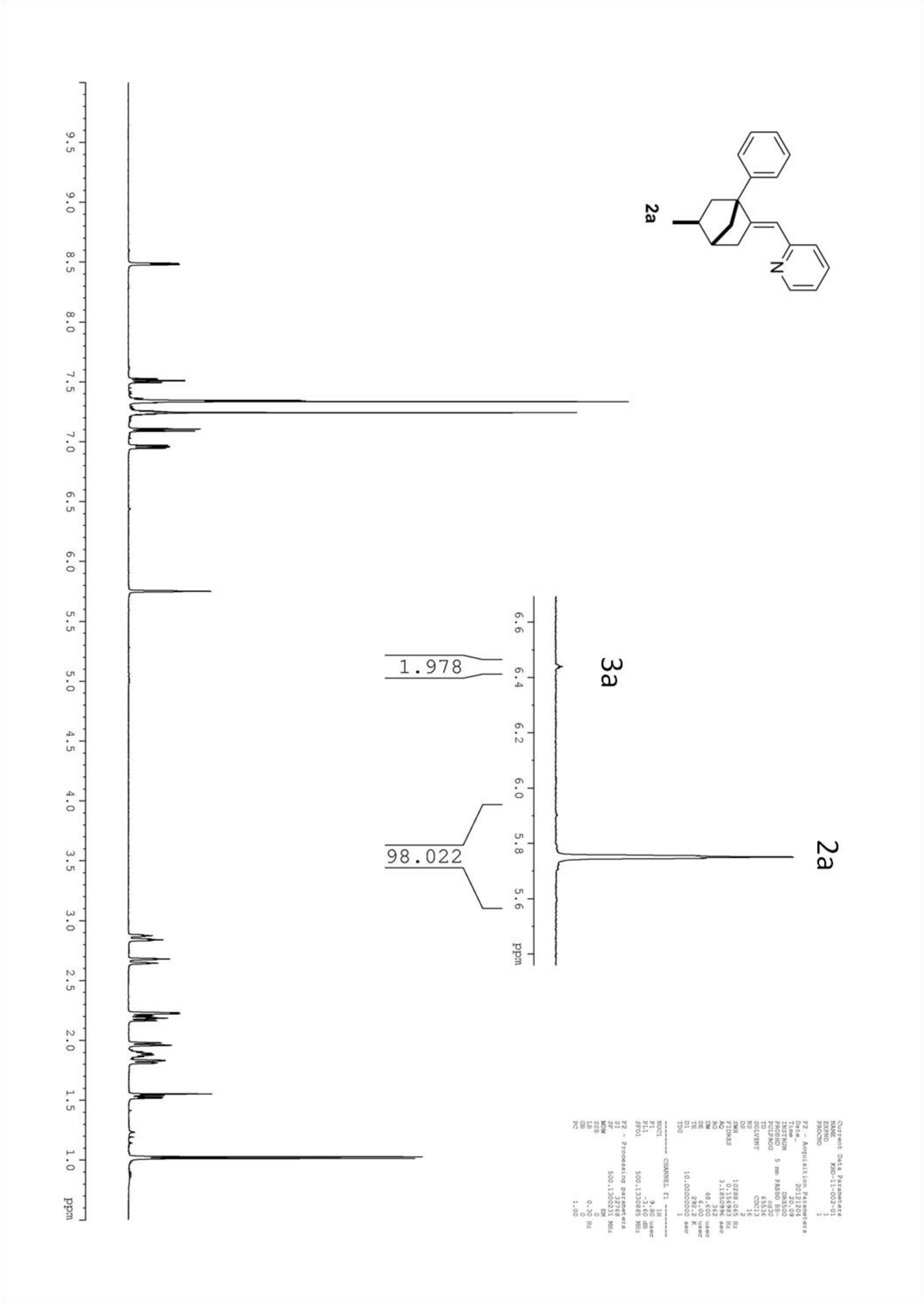
2a
3a
2a
1.978
98.022
ppm
ppm

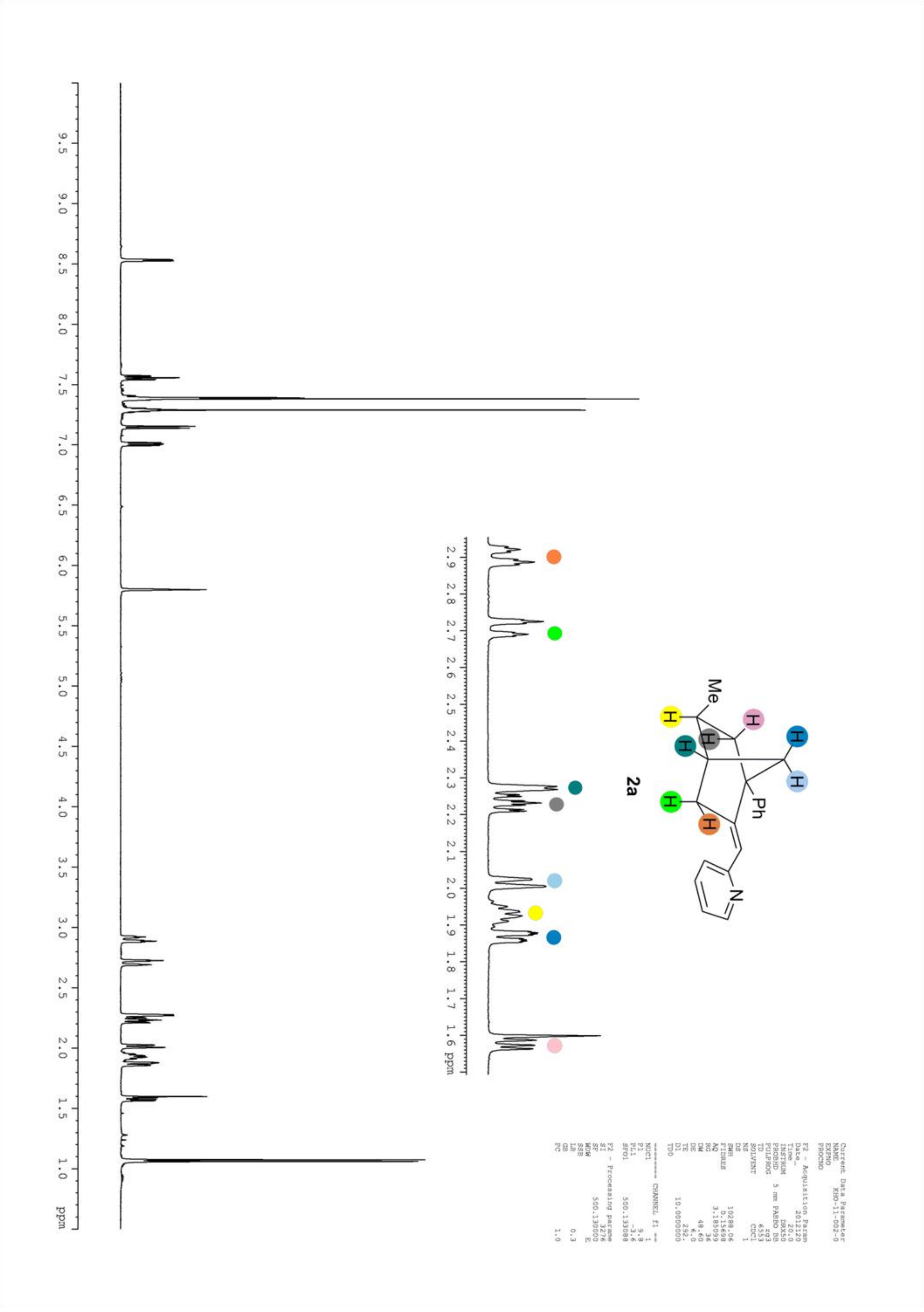
2a
Me
Ph
N
ppm

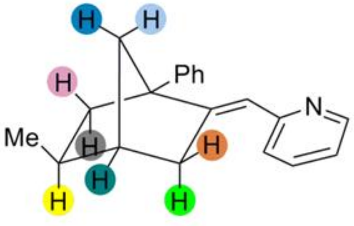

Me
Ph
N
H

2a

COSY

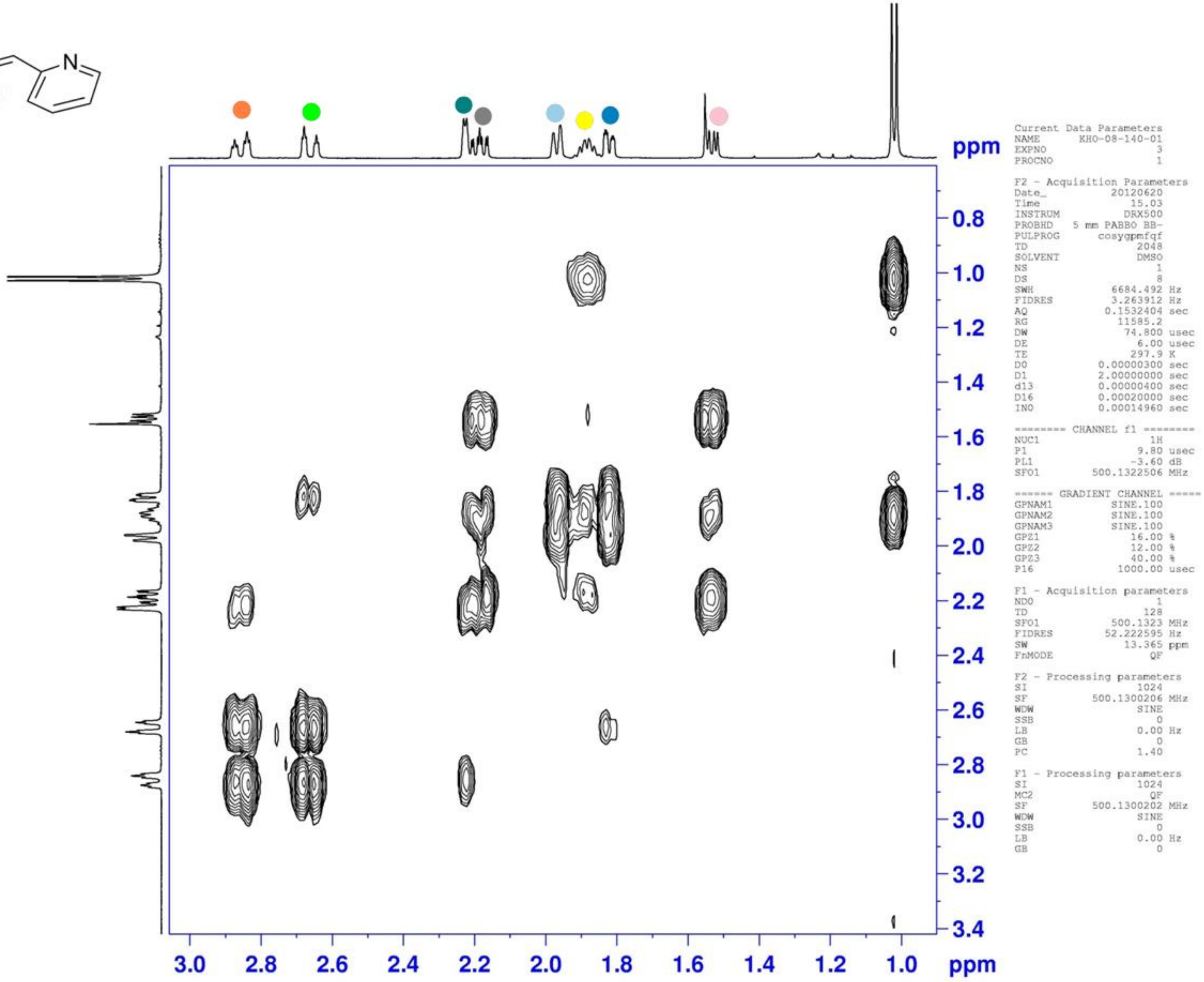

ppm
3.0 2.8 2.6 2.4 2.2 2.0 1.8 1.6 1.4 1.2 1.0
0.8 1.0 1.2 1.4 1.6 1.8 2.0 2.2 2.4 2.6 2.8 3.0 3.2 3.4
Current Data Parameters
NAME KHO-08-140-01
EXPNO 3
PROCNO 1
F2 - Acquisition Parameters
Date_ 20120620
Time 15.03
INSTRUM DRX500
PROBHD 5 mm PABBO BB-
PULPROG cosygpmfqf
TD 2048
SOLVENT DMSO
NS 1
DS 8
SWH 6684.492 Hz
FIDRES 3.263912 Hz
AQ 0.1532404 sec
RG 11585.2
DW 74.800 usec
DE 6.00 usec
TE 297.9 K
D0 0.00000300 sec
D1 2.00000000 sec
d13 0.00000400 sec
D16 0.00020000 sec
IN0 0.00014960 sec
======== CHANNEL f1 ========
NUC1 1H
P1 9.80 usec
PL1 -3.60 dB
SFO1 500.1322506 MHz
====== GRADIENT CHANNEL =====
GPNAM1 SINE.100
GPNAM2 SINE.100
GPNAM3 SINE.100
GPZ1 16.00 %
GPZ2 12.00 %
GPZ3 40.00 %
P16 1000.00 usec
F1 - Acquisition parameters
ND0 1
TD 128
SFO1 500.1323 MHz
FIDRES 52.222595 Hz
SW 13.365 ppm
FnMODE QF
F2 - Processing parameters
SI 1024
SF 500.1300206 MHz
WDW SINE
SSB 0
LB 0.00 Hz
GB 0
PC 1.40
F1 - Processing parameters
SI 1024
MC2 QF
SF 500.1300202 MHz
WDW SINE
SSB 0
LB 0.00 Hz
GB 0

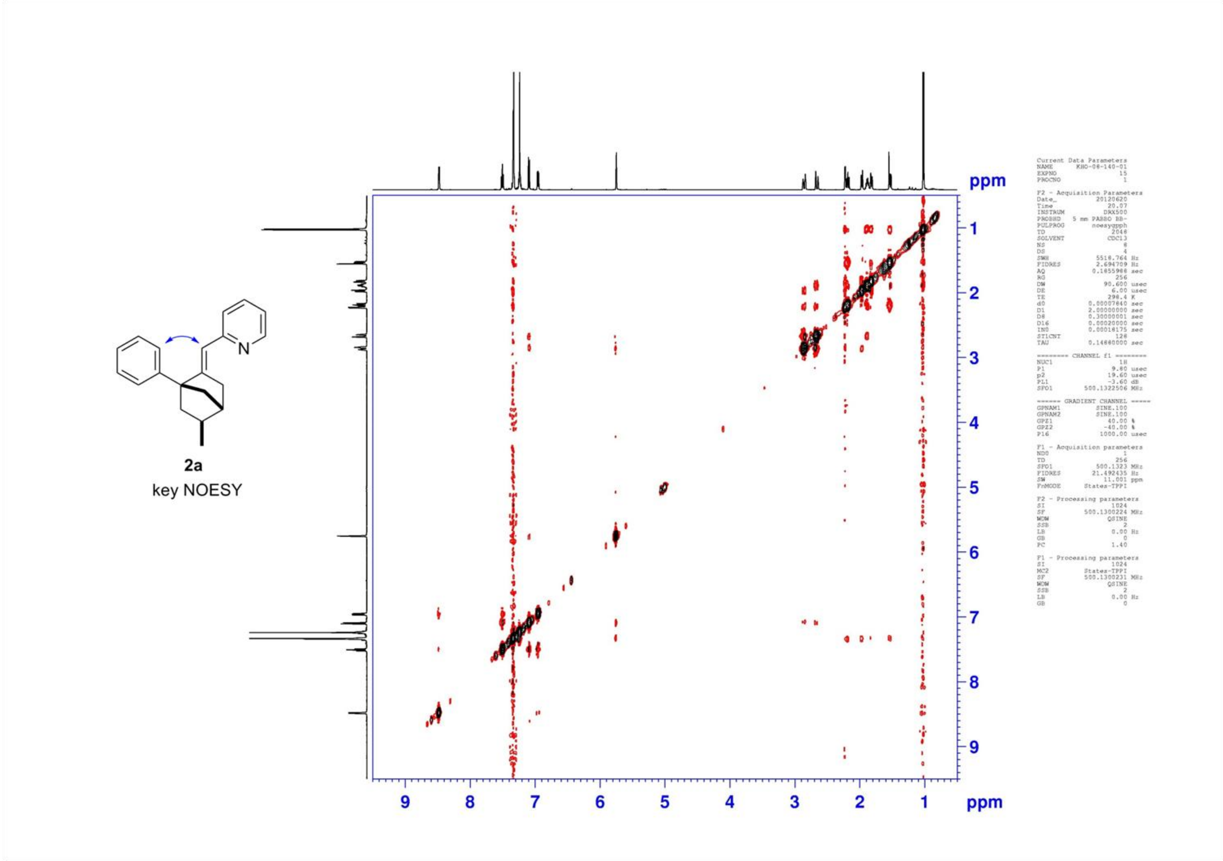

2a
key NOESY
ppm
ppm

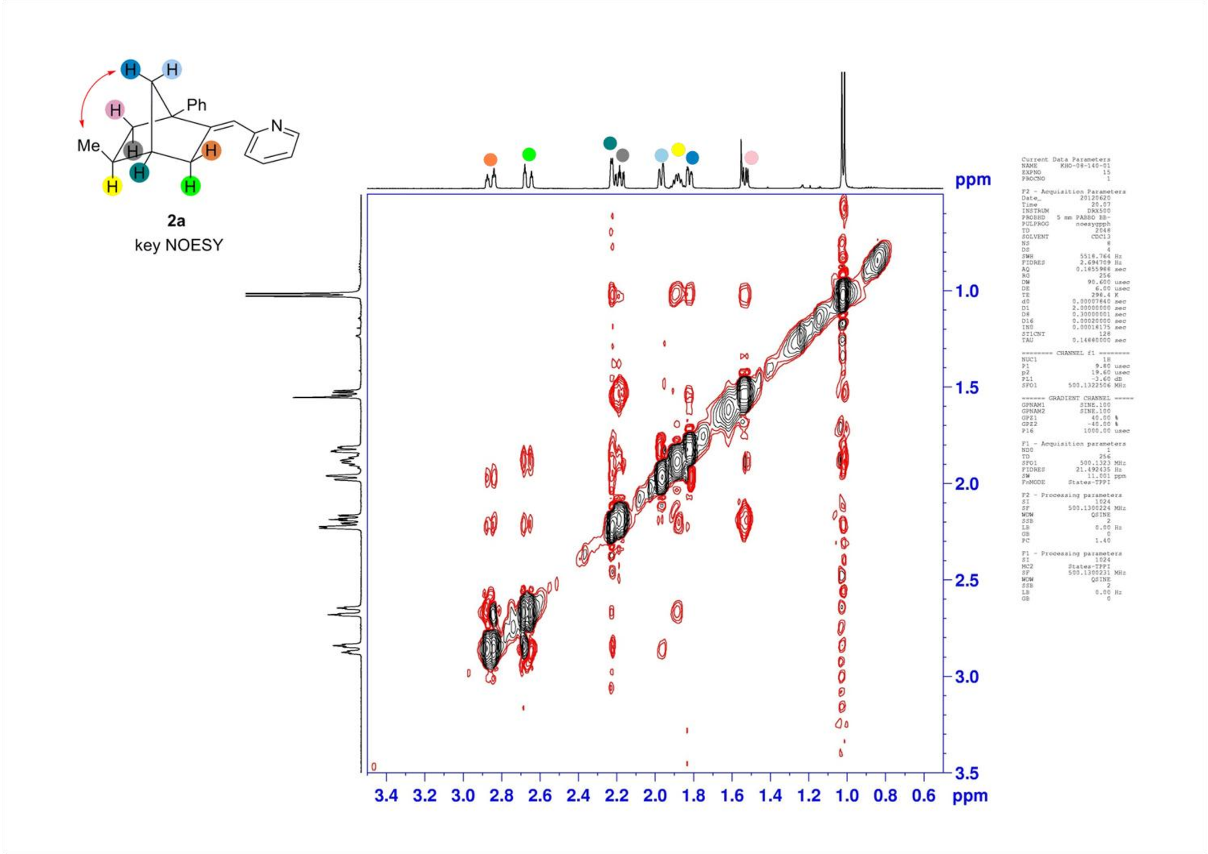

Ph
Me
N
2a
key NOESY
ppm
ppm

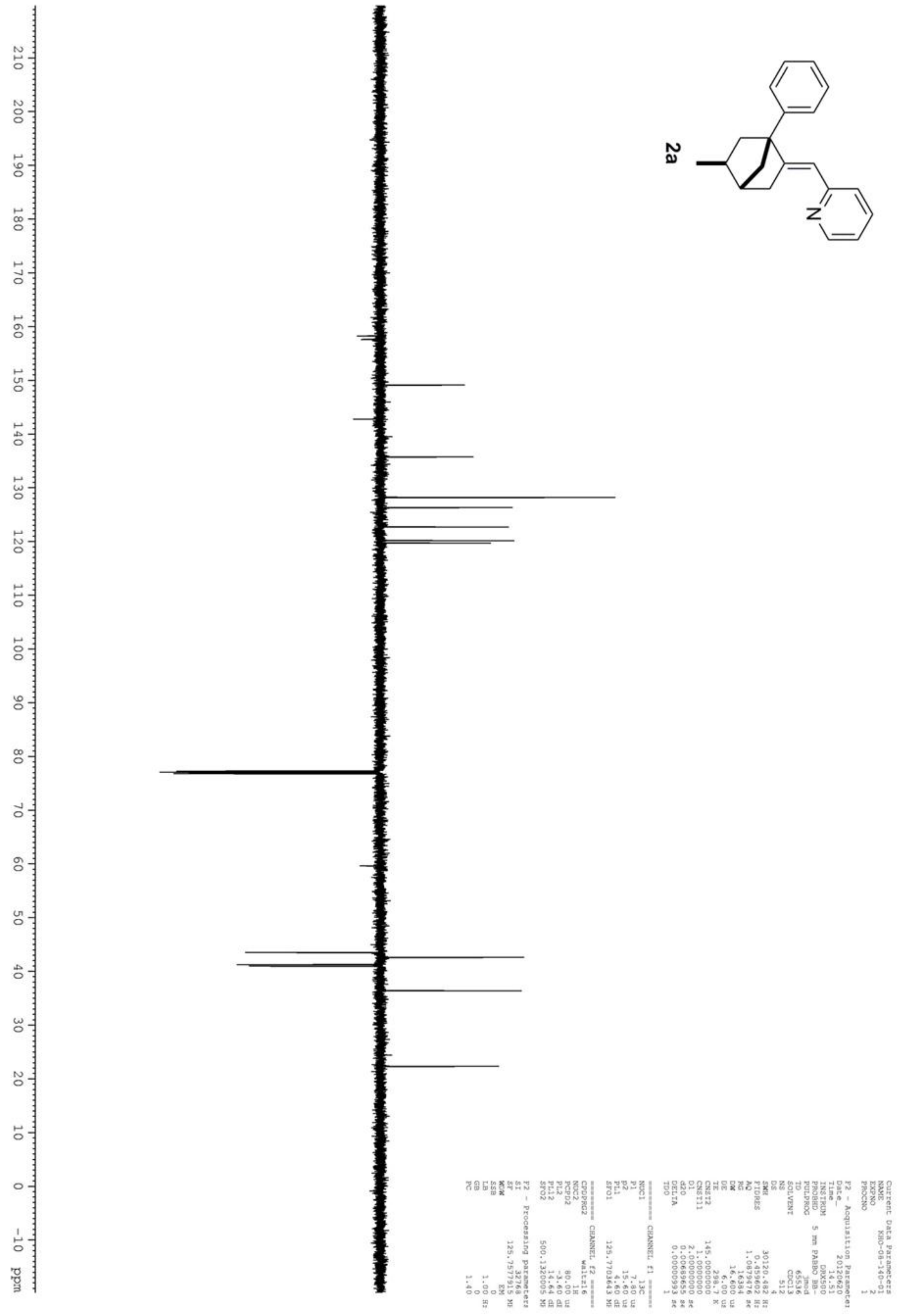
2a
210 200 190 180 170 160 150 140 130 120 110 100 90 80 70 60 50 40 30 20 10 0 -10 ppm
Current Data Parameters

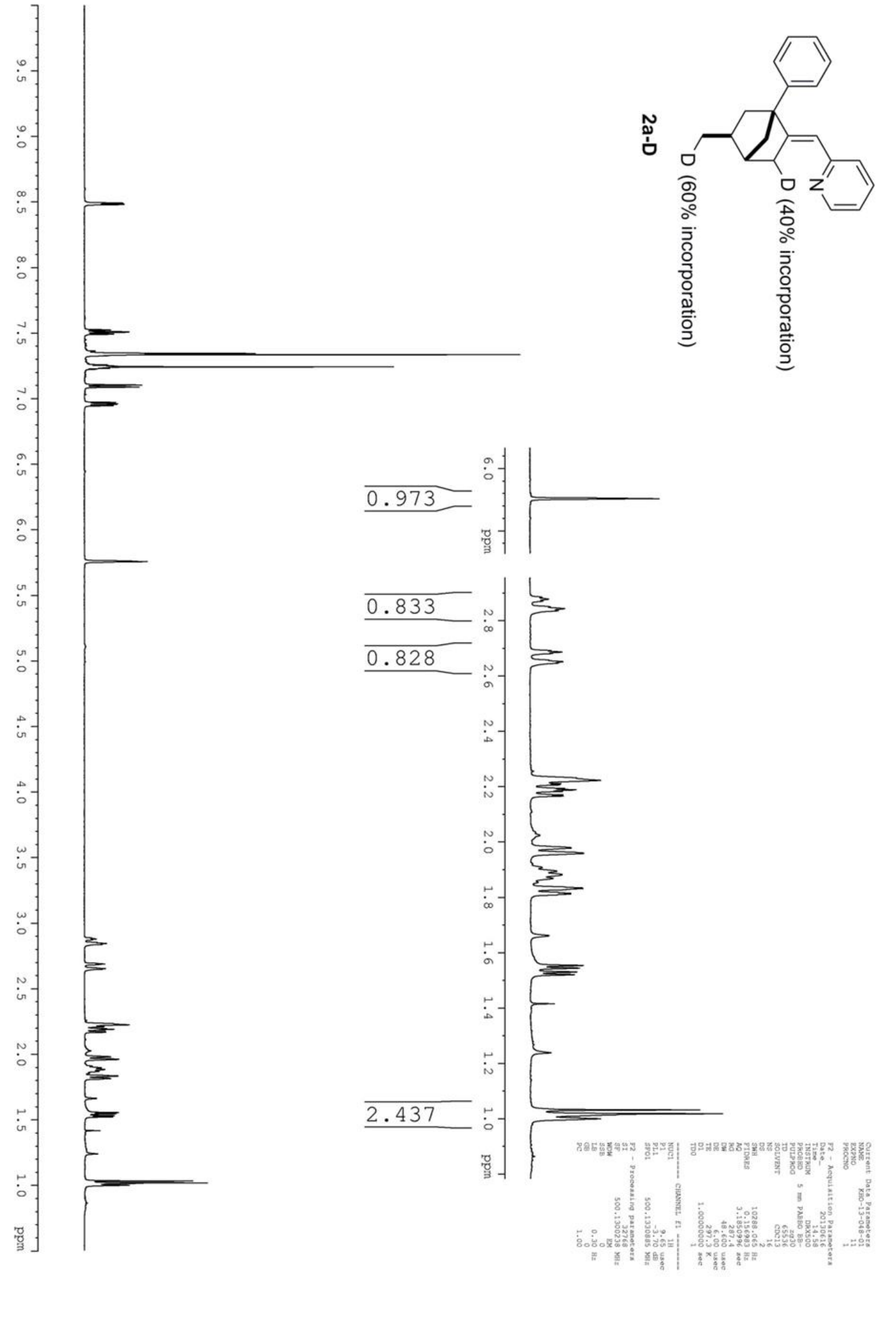
2a-D
D (40% incorporation)
D (60% incorporation)
0.973
0.833
0.828
2.437
ppm

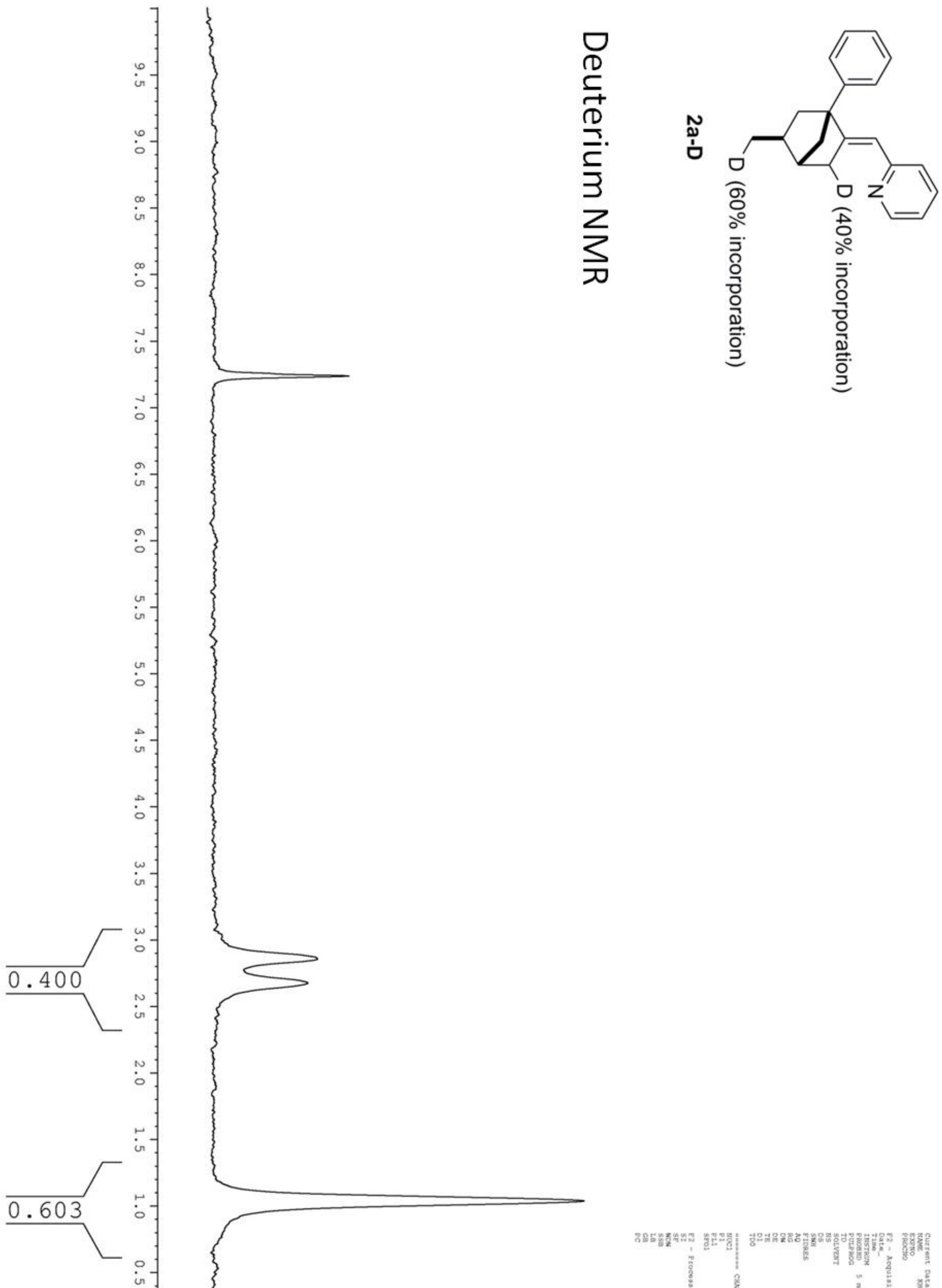

Deuterium NMR
2a-D
D (40% incorporation)
D (60% incorporation)
0.400
0.603
ppm

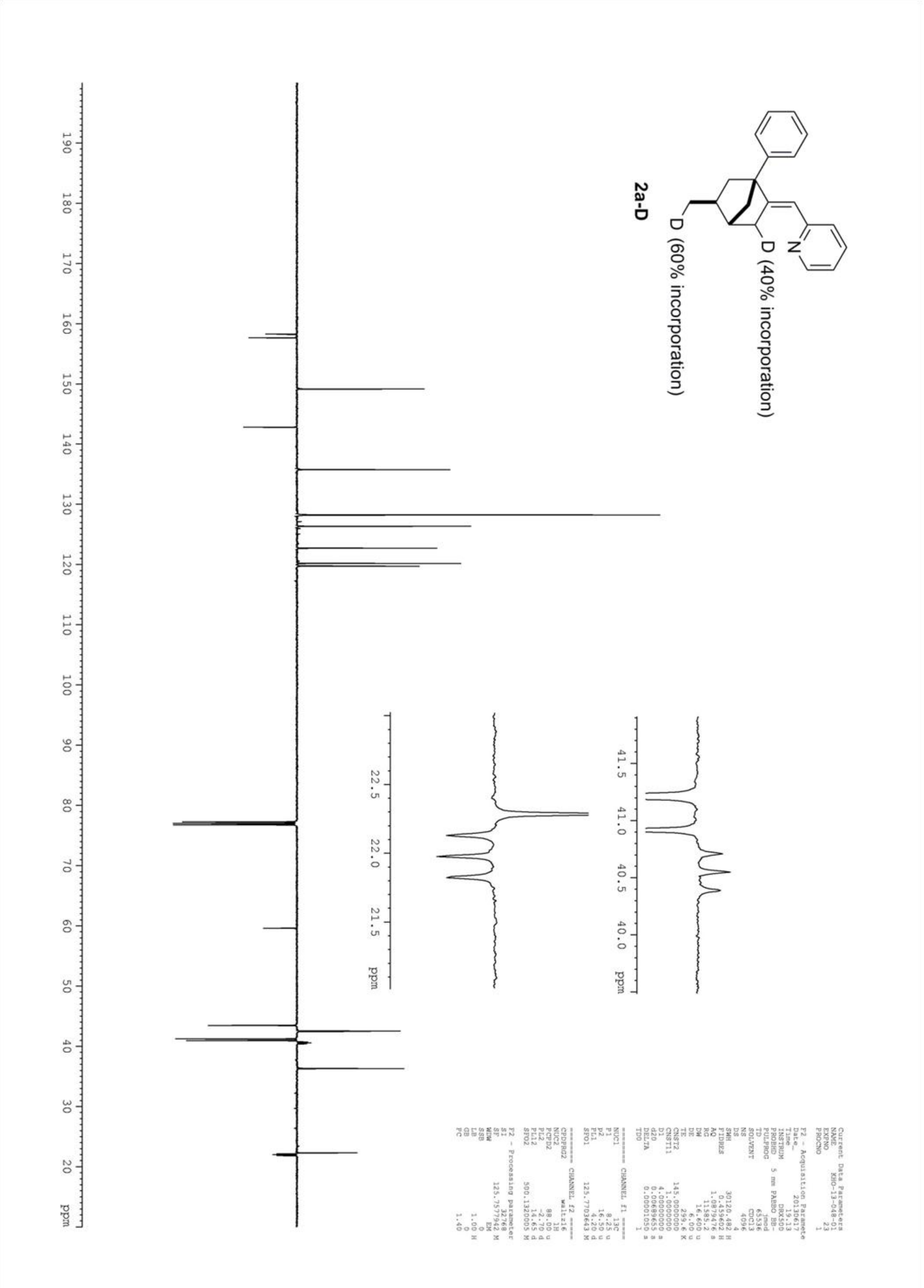
2a-D
D (40% incorporation)
D (60% incorporation)

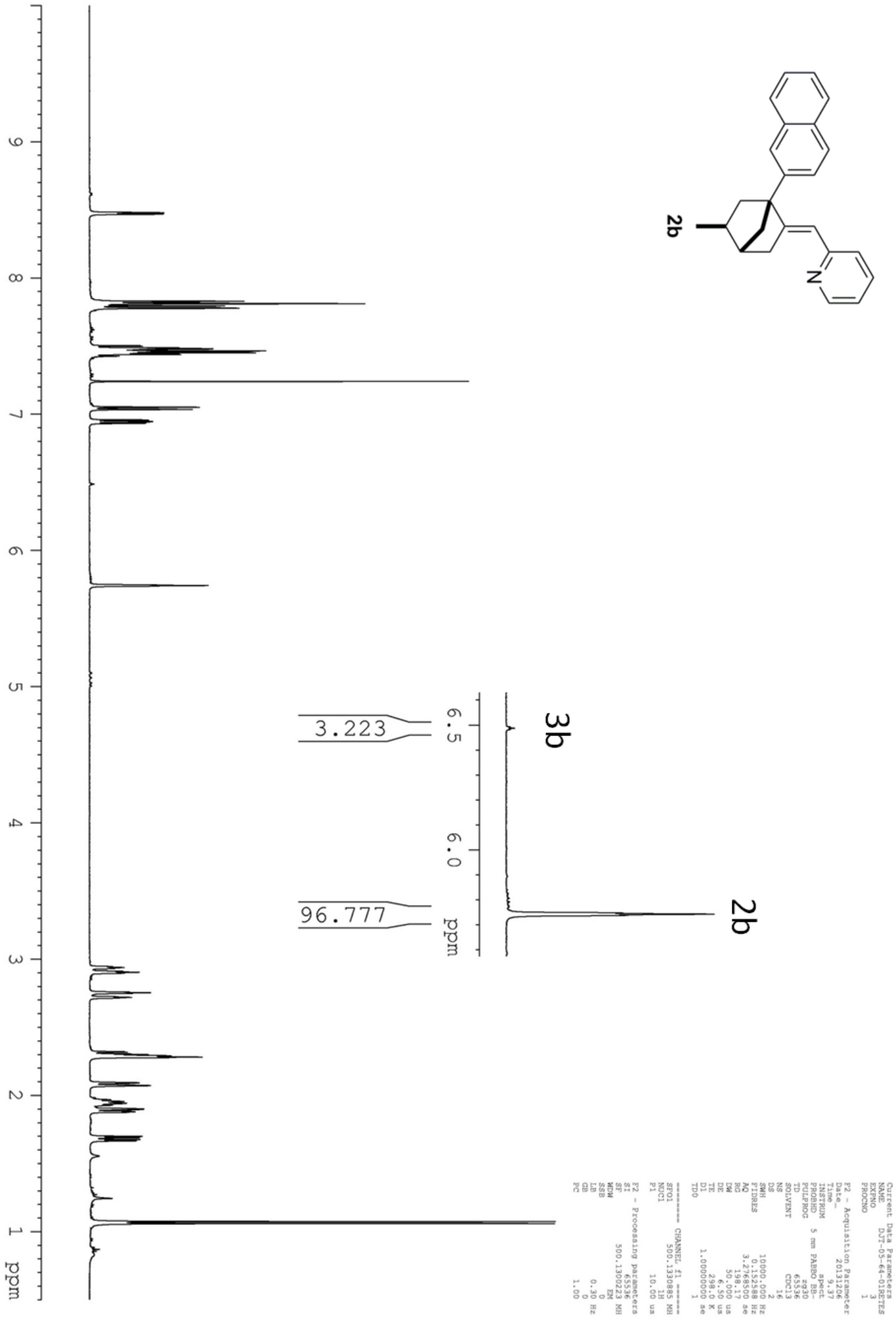
2b
3b
3.223
96.777
6.5
6.0
ppm
2b
9
8
7
6
5
4
3
2
1
ppm

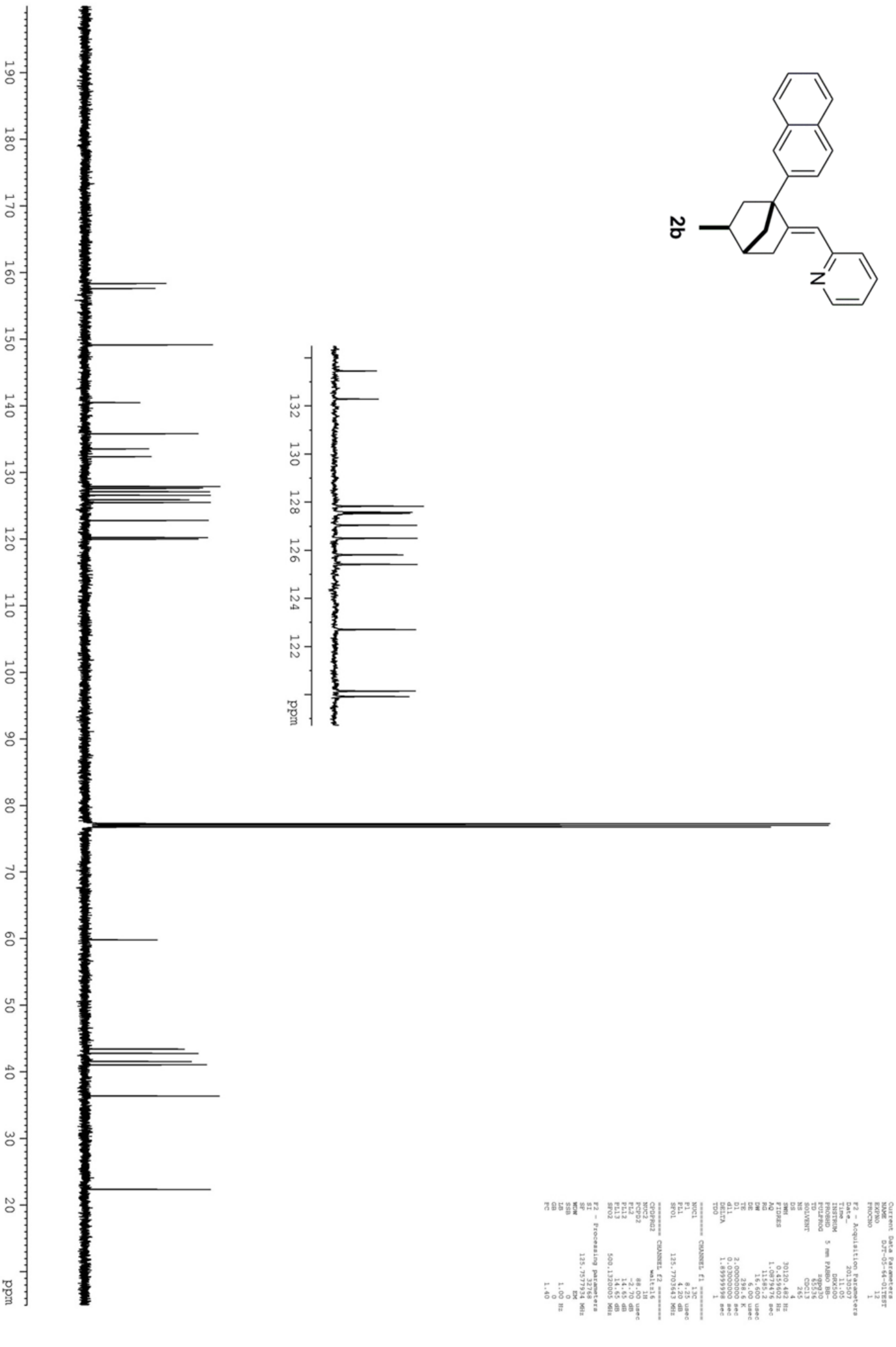
2b
ppm

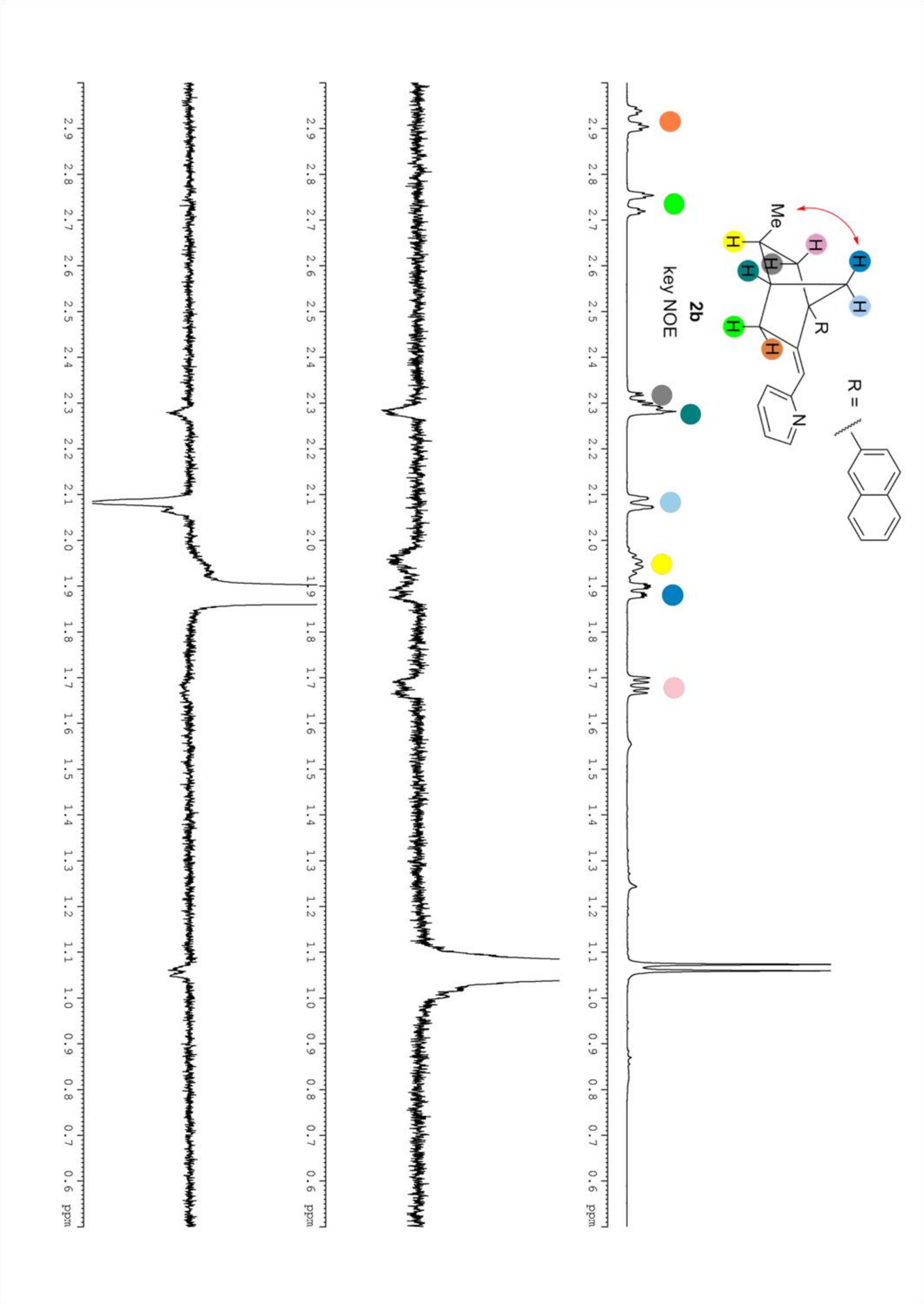

R =
Me
R
N
2b
key NOE
ppm

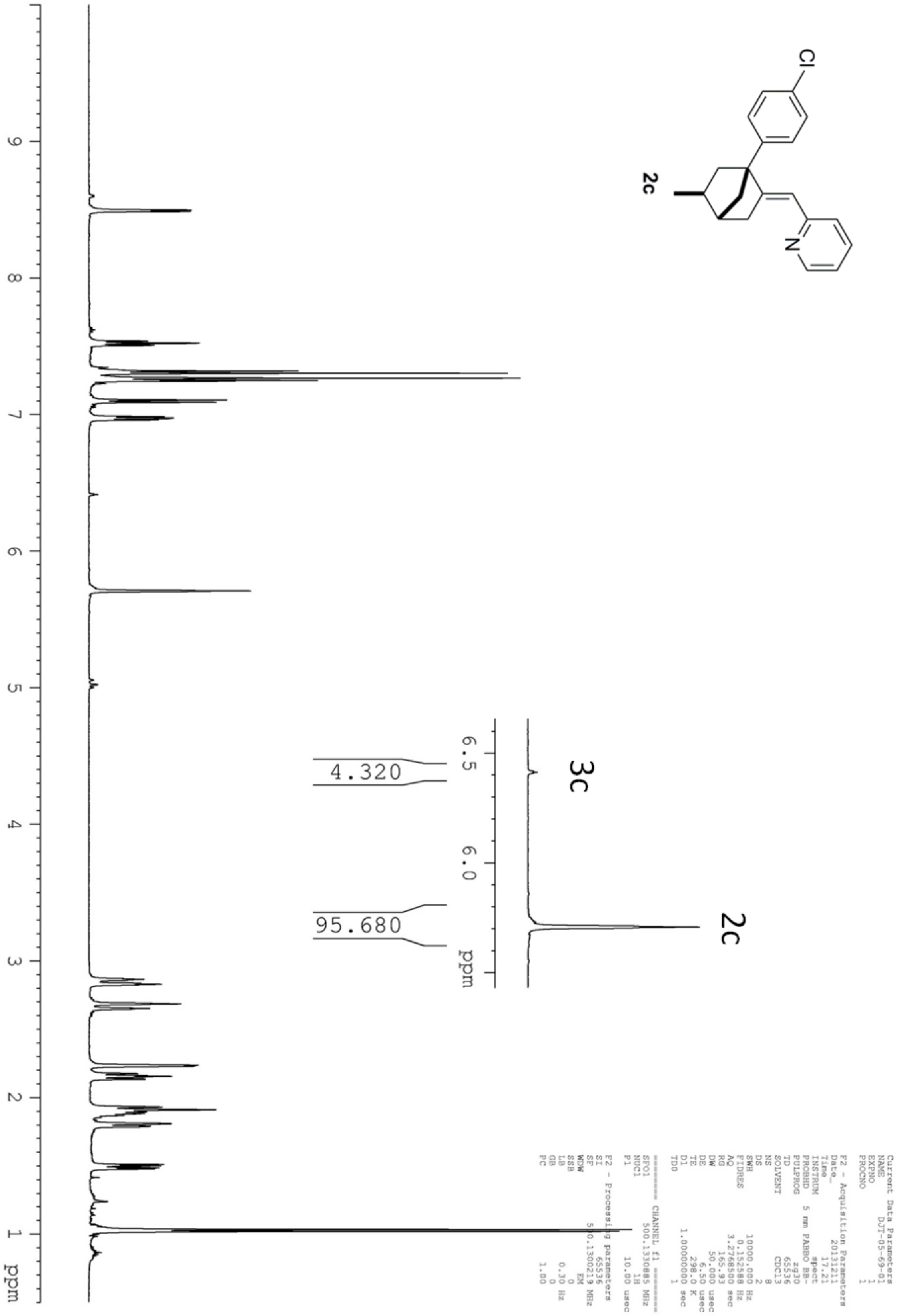
2c
Cl
N
3c
2c
4.320
95.680
6.5
6.0
ppm
9
8
7
6
5
4
3
2
1
ppm
Current Data Parameters
NAME DJT-05-69-01
EXPNO 1
PROCNO 1
F2 - Acquisition Parameters
Date_ 20131211
Time 17.21
INSTRUM spect
PROBHD 5 mm PABBO BB-
PULPROG zg30
TD 65536
SOLVENT CDCl3
NS 8
DS 2
SWH 10000.000 Hz
FIDRES 0.152588 Hz
AQ 3.2768500 sec
RG 165.93
DW 50.000 usec
DE 6.50 usec
TE 298.0 K
D1 1.00000000 sec
TD0 1
======== CHANNEL f1 ========
SFO1 500.1330885 MHz
NUC1 1H
P1 10.00 usec
F2 - Processing parameters
SI 65536
SF 500.1300219 MHz
WDW EM
SSB 0
LB 0.30 Hz
GB 0
PC 1.00

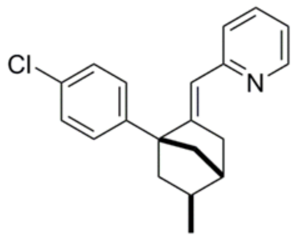

2c

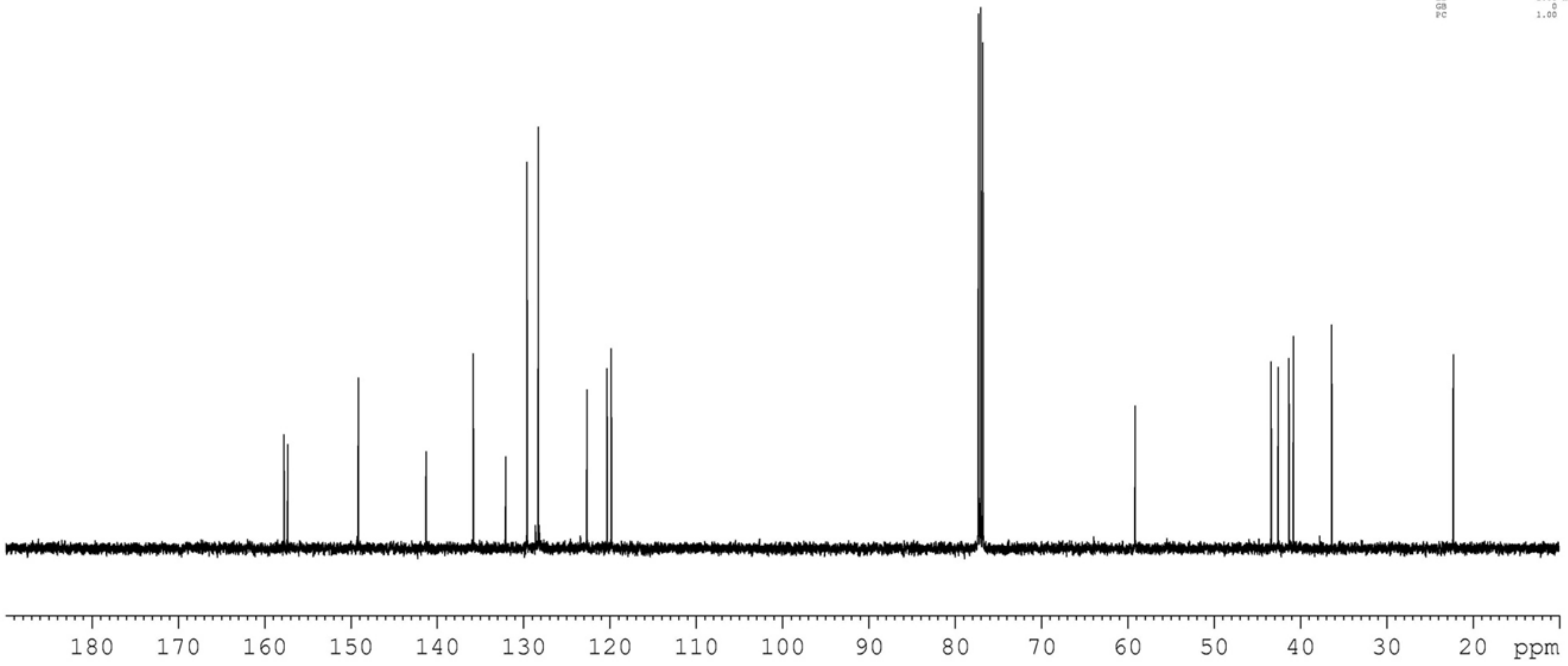


```
Current Data Parameters
NAME     DJT-05-69-01TEST
EXPNO                  10
PROCNO                  1

F2 - Acquisition Parameters
Date_            20130307
Time                13.48
INSTRUM            DRX500
PROBHD   5 mm PABBO BB-
PULPROG            zgpg30
TD                  65536
SOLVENT             CDCl3
NS                     91
DS                      4
SWH             30120.482 Hz
FIDRES           0.459602 Hz
AQ              1.0879476 sec
RG                11585.2
DW                 16.600 usec
DE                   6.00 usec
TE                  293.6 K
D1             2.00000000 sec
d11            0.03000000 sec
DELTA          1.89999998 sec
TD0                     1

======== CHANNEL f1 ========
NUC1                  13C
P1                   8.25 usec
PL1                  4.20 dB
SFO1          125.7703643 MHz

======== CHANNEL f2 ========
CPDPRG2           waltz16
NUC2                   1H
PCPD2               88.00 usec
PL2                 -2.70 dB
PL12                14.65 dB
PL13                14.65 dB
SFO2          500.1320005 MHz

F2 - Processing parameters
SI                  32768
SF            125.7577970 MHz
WDW                    EM
SSB                     0
LB                   1.00 Hz
GB                      0
PC                   1.00
```

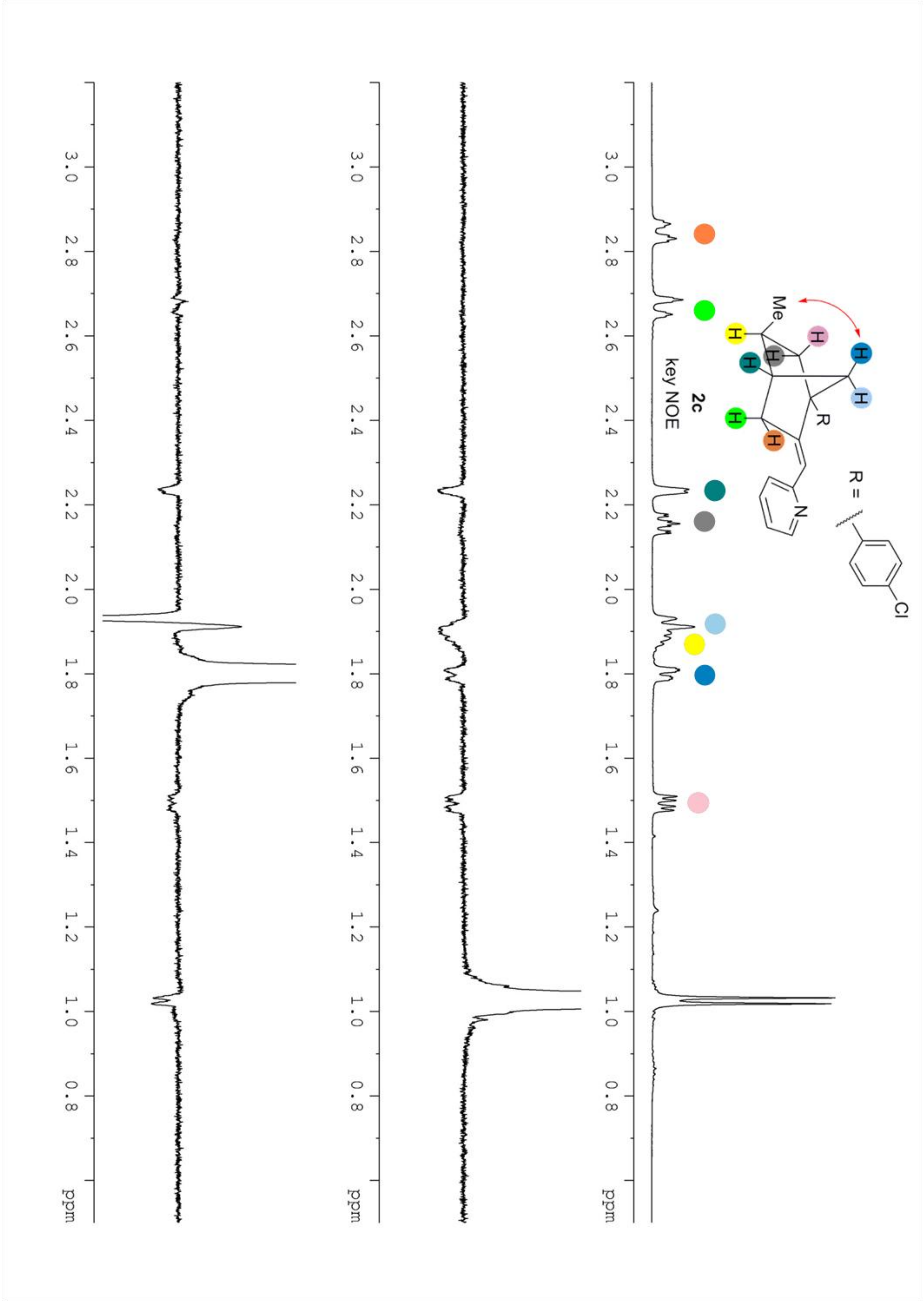

R =
Cl
Me
R
N
2c
key NOE
3.0
2.8
2.6
2.4
2.2
2.0
1.8
1.6
1.4
1.2
1.0
0.8
ppm

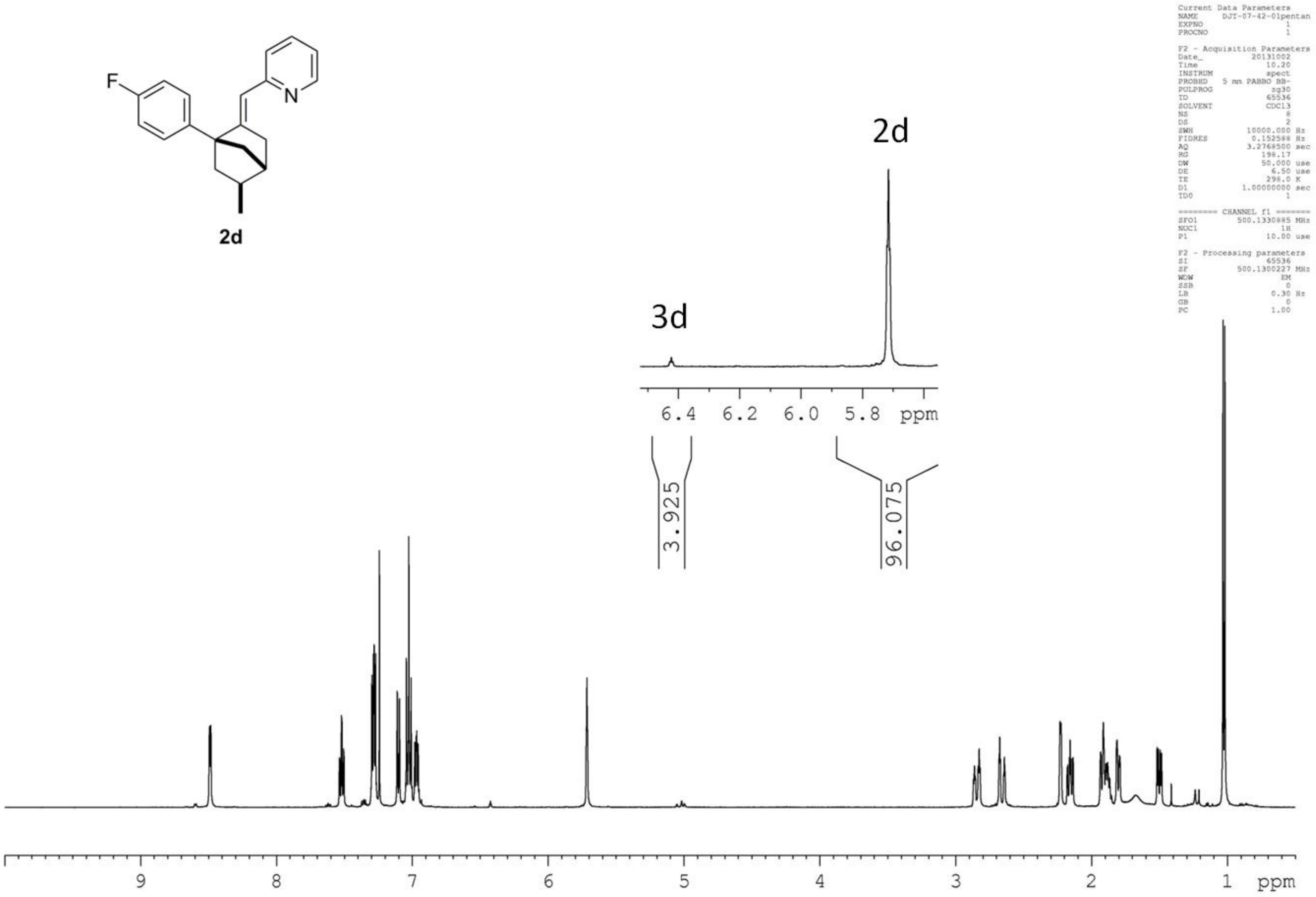
F
N
2d
2d
3d
6.4
6.2
6.0
5.8
ppm
3.925
96.075
9
8
7
6
5
4
3
2
1
ppm
Current Data Parameters
NAME DJT-07-42-01pentan
EXPNO 1
PROCNO 1
F2 - Acquisition Parameters
Date_ 20131002
Time 10.20
INSTRUM spect
PROBHD 5 mm PABBO BB-
PULPROG zg30
TD 65536
SOLVENT CDCl3
NS 8
DS 2
SWH 10000.000 Hz
FIDRES 0.152588 Hz
AQ 3.2768500 sec
RG 198.17
DW 50.000 use
DE 6.50 use
TE 298.0 K
D1 1.00000000 sec
TD0 1
======== CHANNEL f1 ========
SFO1 500.1330885 MHz
NUC1 1H
P1 10.00 use
F2 - Processing parameters
SI 65536
SF 500.1300227 MHz
WDW EM
SSB 0
LB 0.30 Hz
GB 0
PC 1.00

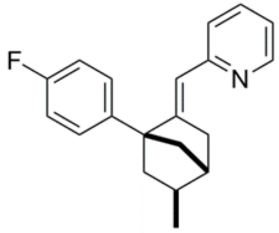

2d

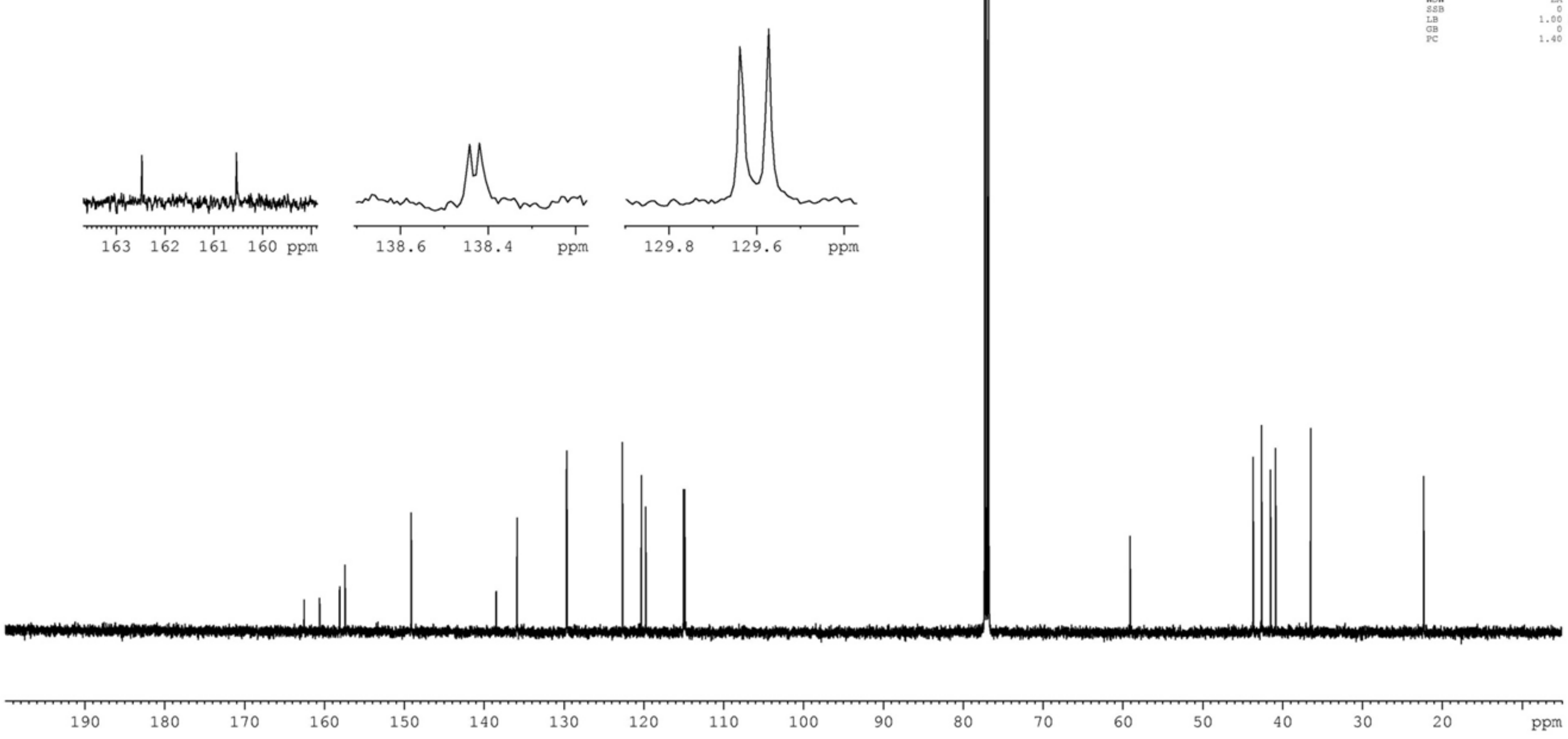

Current Data Parameters
NAME DJT-07-42-01pentan
EXPNO 10
PROCNO 1

F2 - Acquisition Parameters
Date_ 20131002
Time 10.43
INSTRUM spect
PROBHD 5 mm PABBO BB-
PULPROG zgpg30
TD 65536
SOLVENT CDCl3
NS 379
DS 4
SWH 29761.904 Hz
FIDRES 0.454131 Hz
AQ 1.1010548 sec
RG 198.17
DW 16.800 use
DE 6.50 use
TE 298.0 K
D1 2.00000000 sec
D11 0.03000000 sec
TD0 1

======== CHANNEL f1 ========
SFO1 125.7703637 MHz
NUC1 13C
P1 8.00 use

F2 - Processing parameters
SI 32768
SF 125.7577924 MHz
WDW EM
SSB 0
LB 1.00 Hz
GB 0
PC 1.40

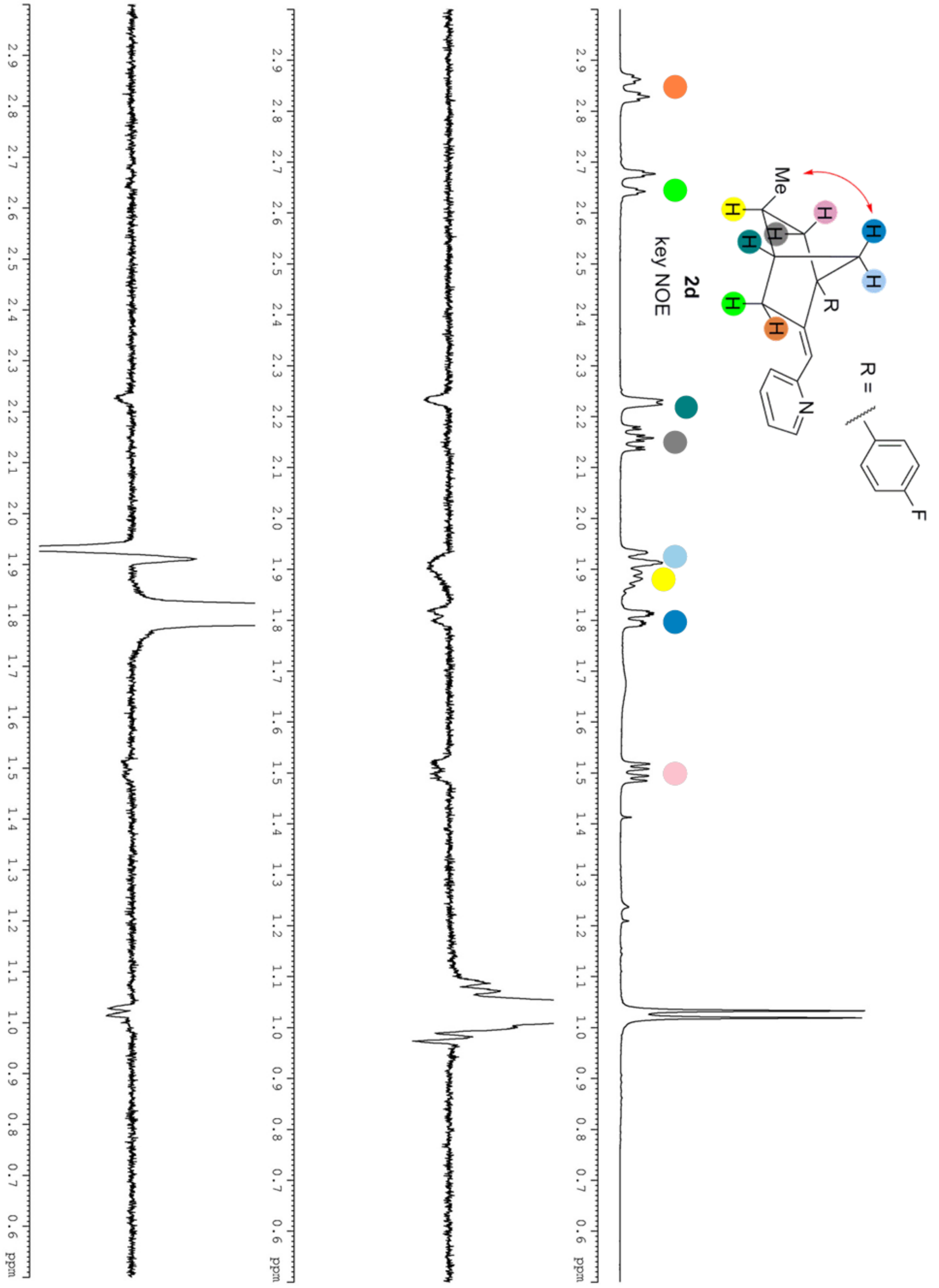

2d
key NOE
R =
ppm

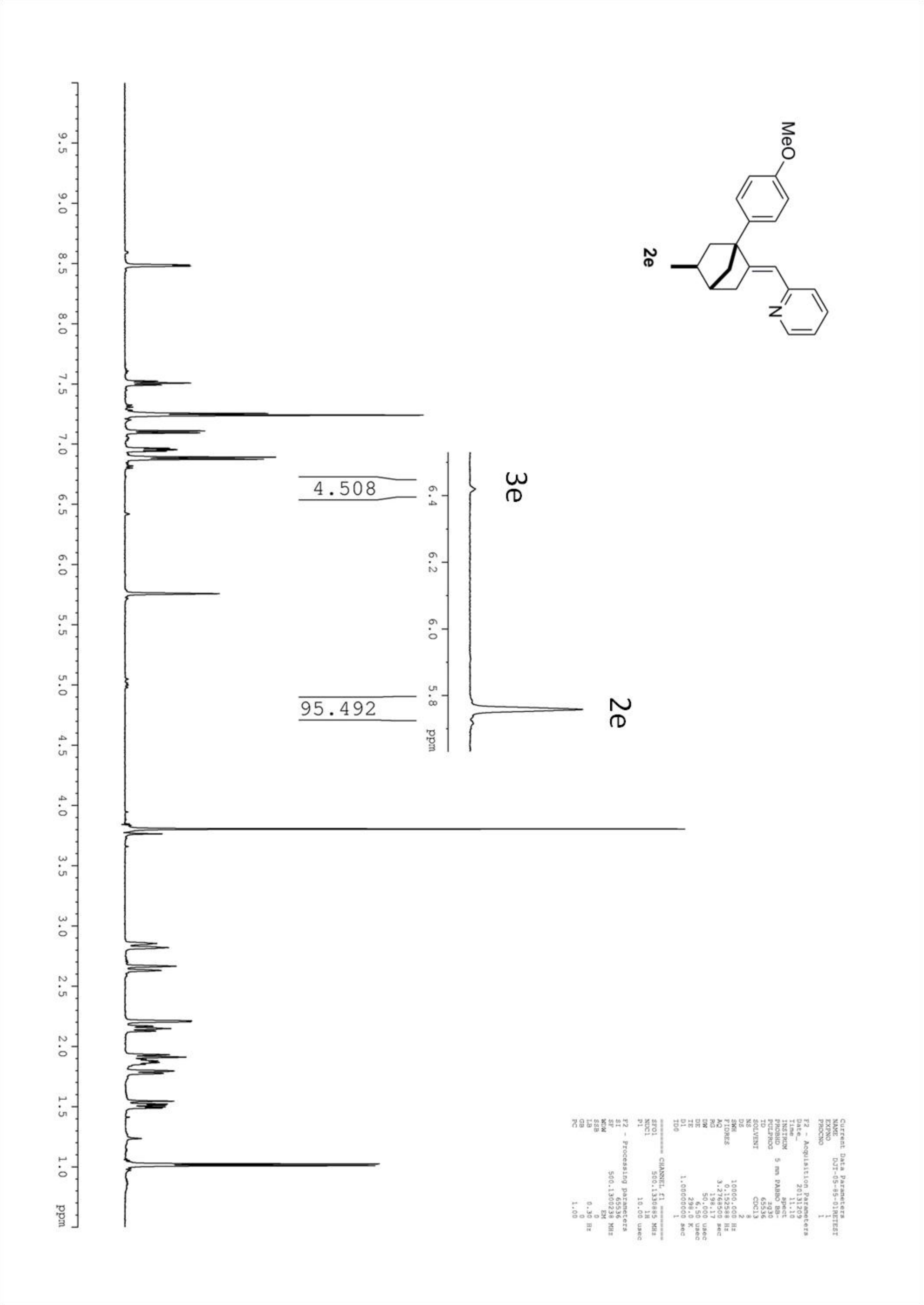
MeO
N
2e
3e
4.508
95.492
ppm

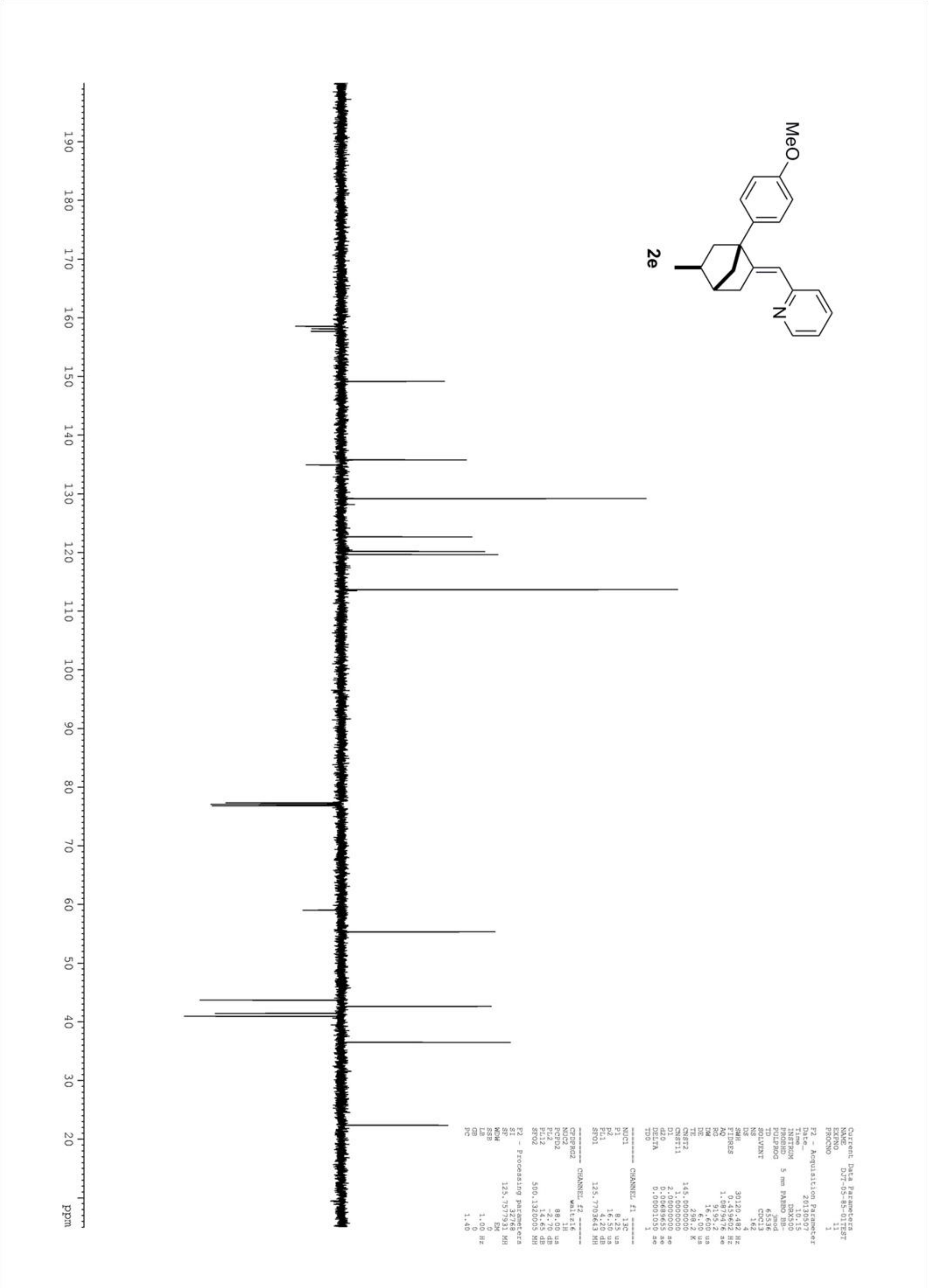
MeO
N
2e
ppm

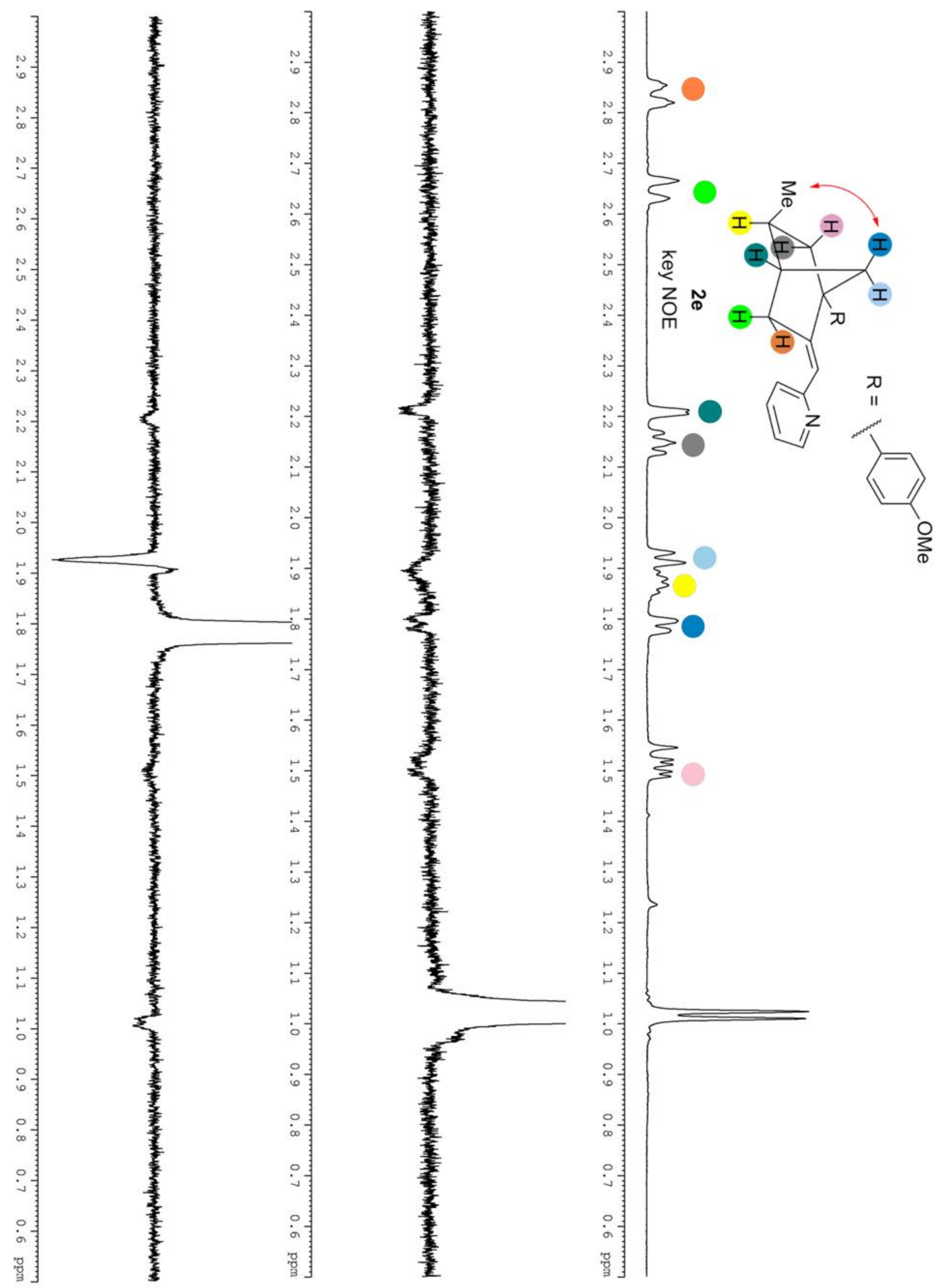
R =
OMe
Me
R
N
2e
key NOE
ppm

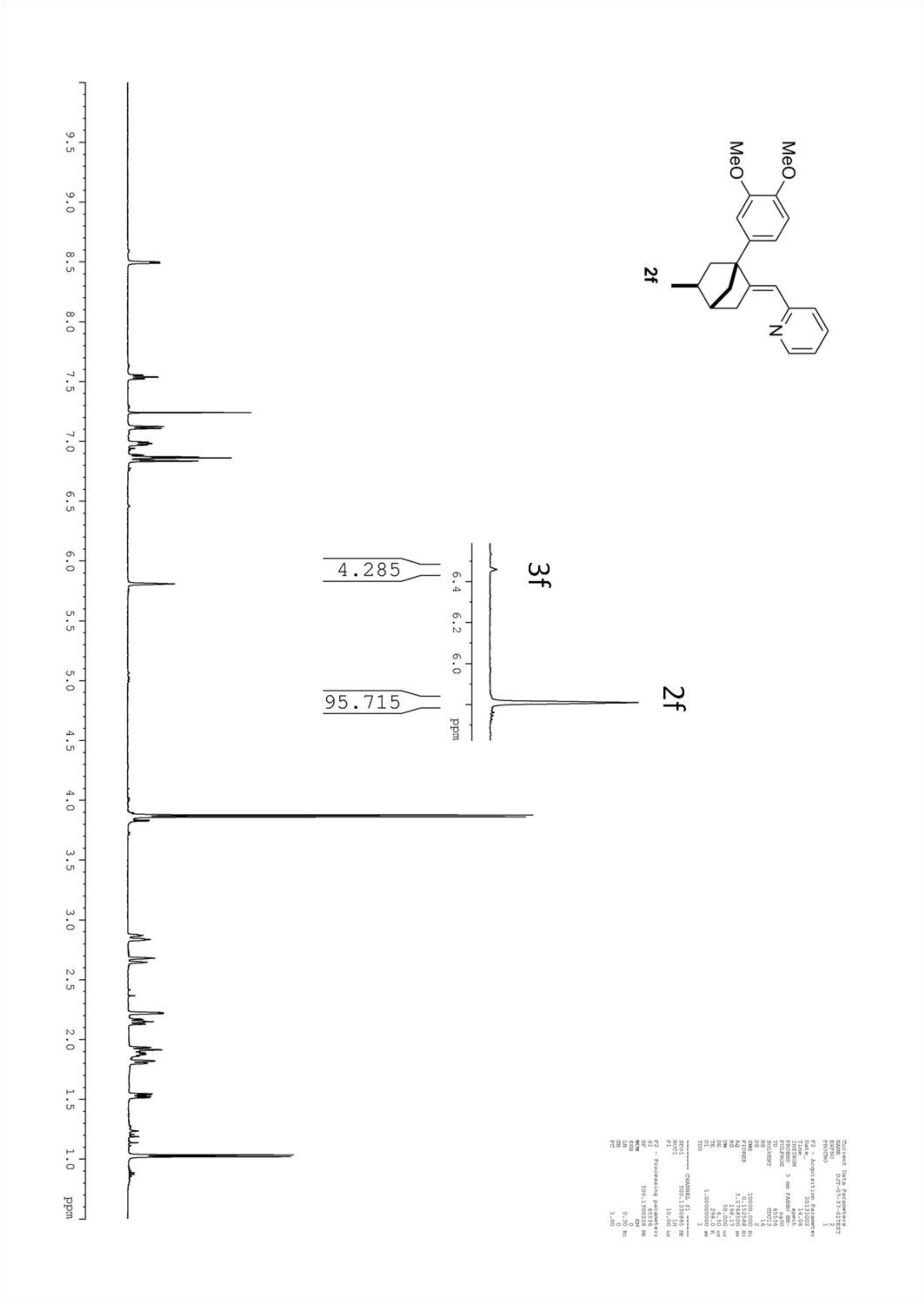
MeO
MeO
N
2f
3f
2f
4.285
95.715
ppm

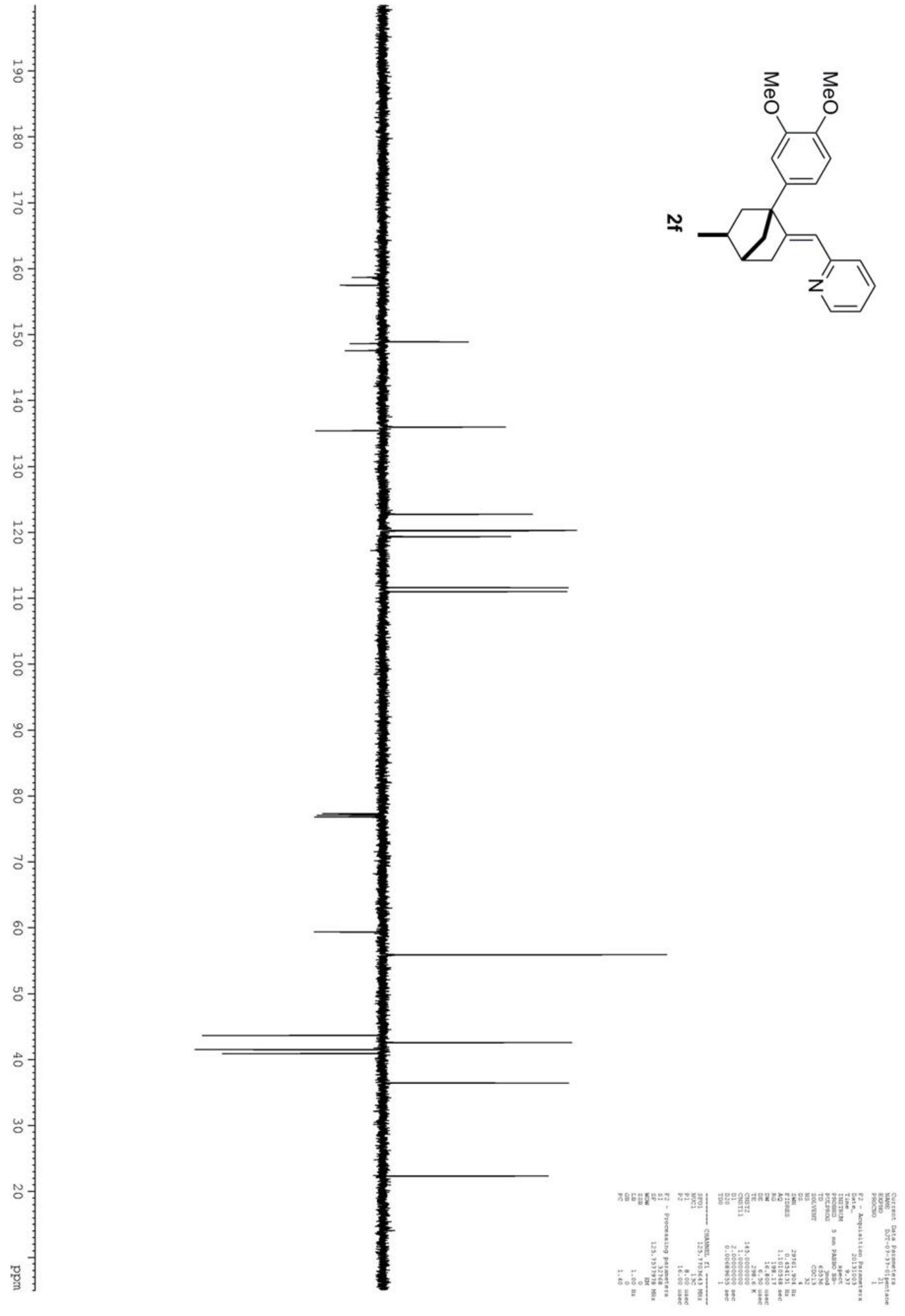

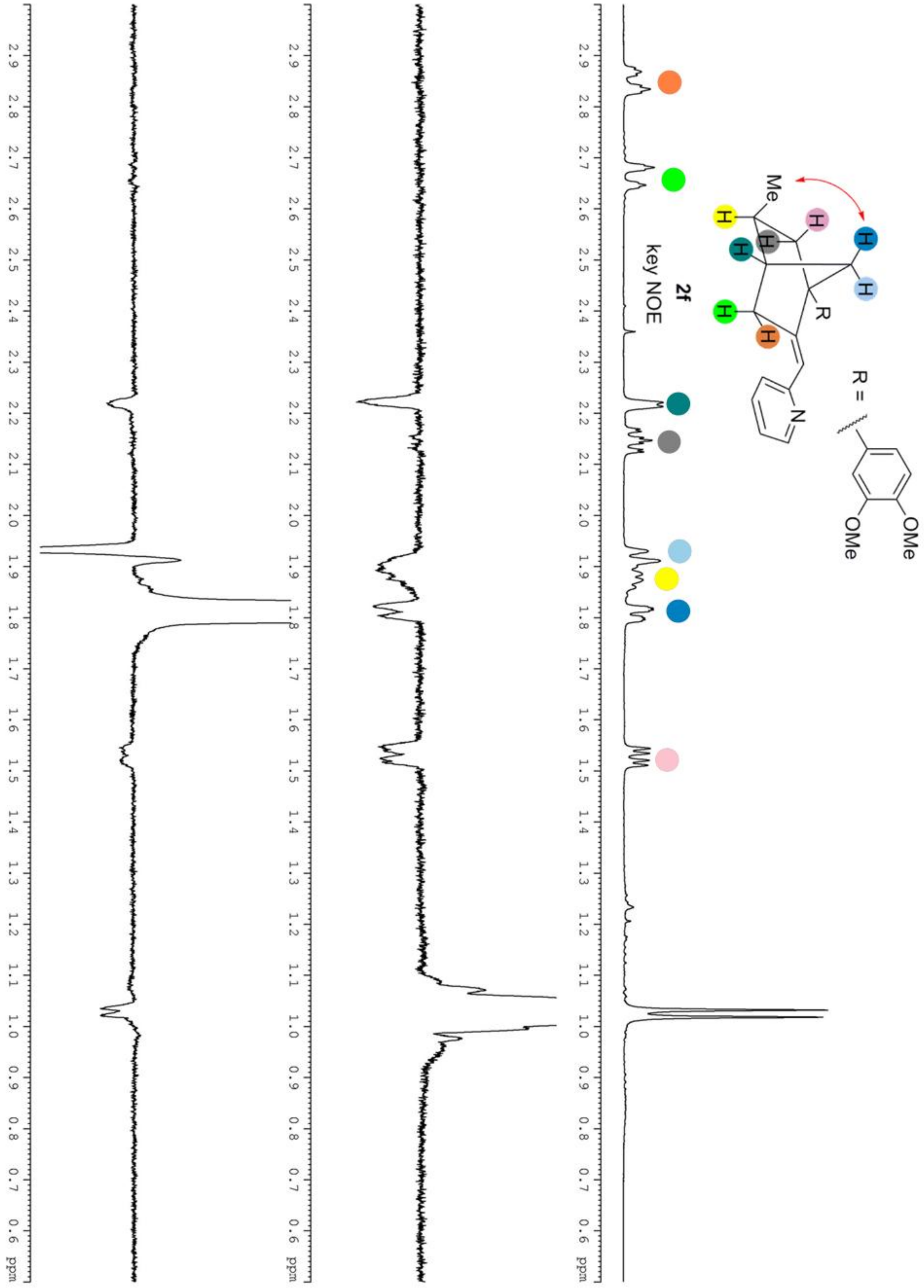

2f
key NOE
R =
OMe
OMe
Me
R
N
ppm

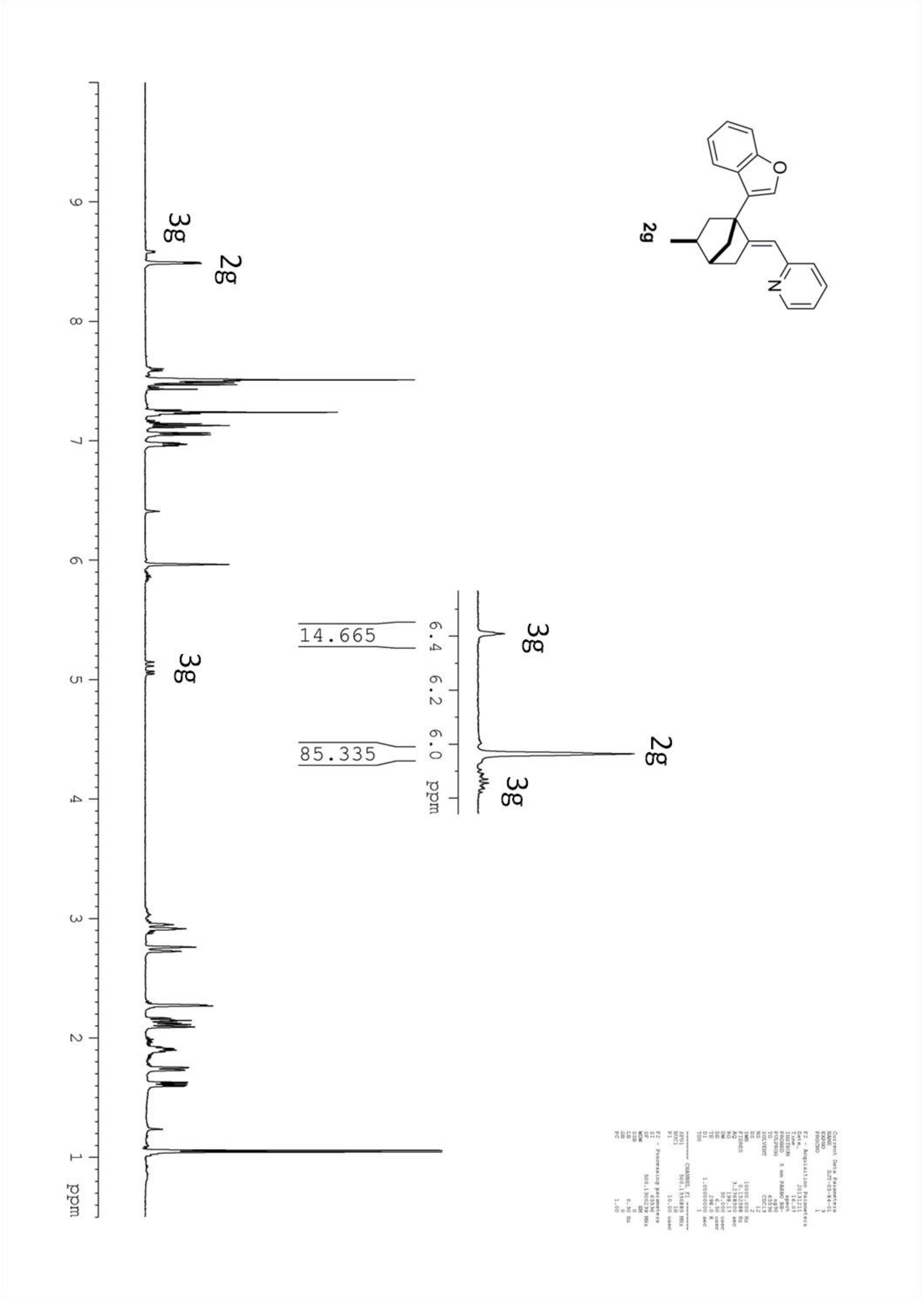
2g
3g
2g
3g
2g
3g
3g
14.665
85.335
ppm
ppm

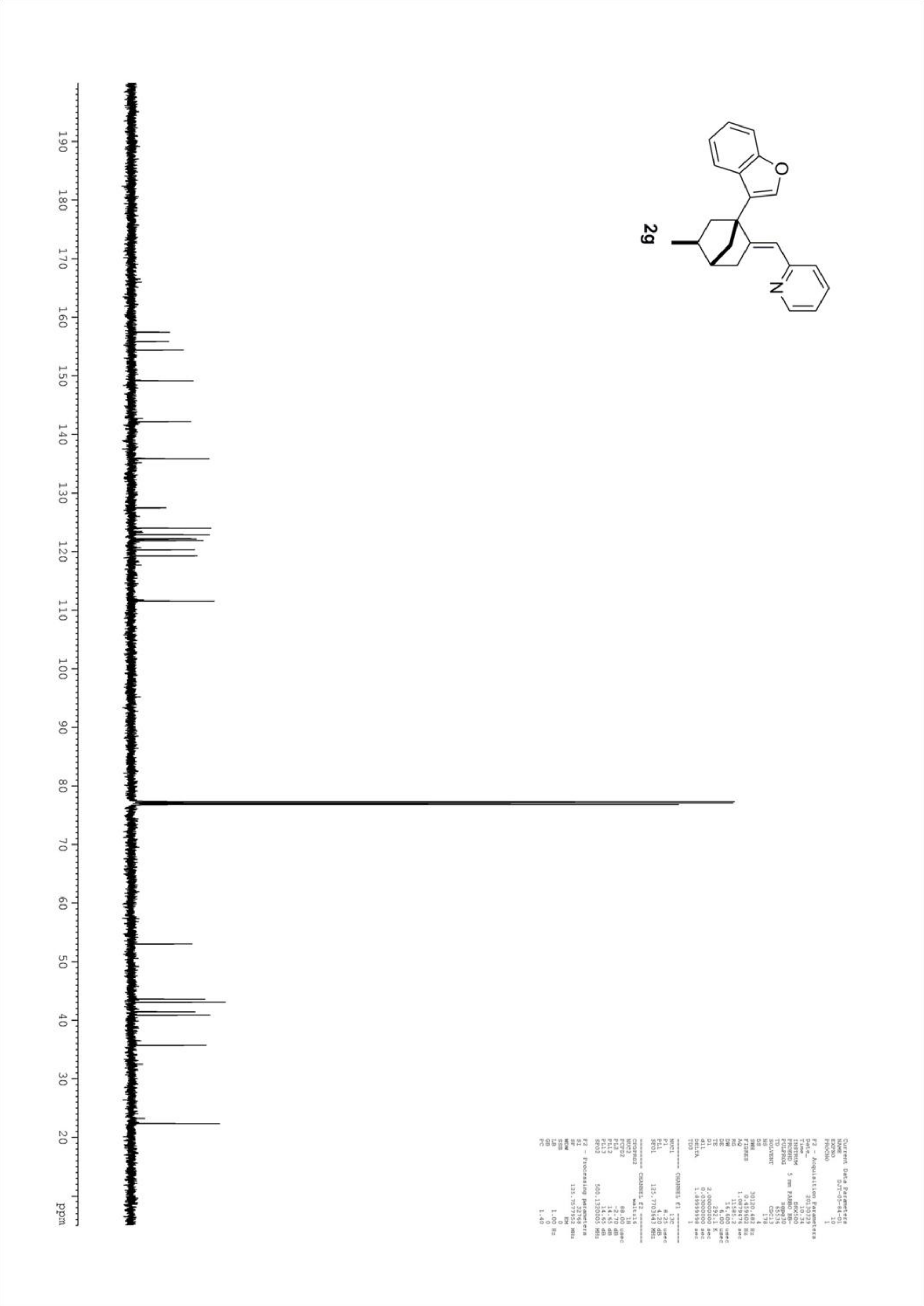
2g
190
180
170
160
150
140
130
120
110
100
90
80
70
60
50
40
30
20
ppm

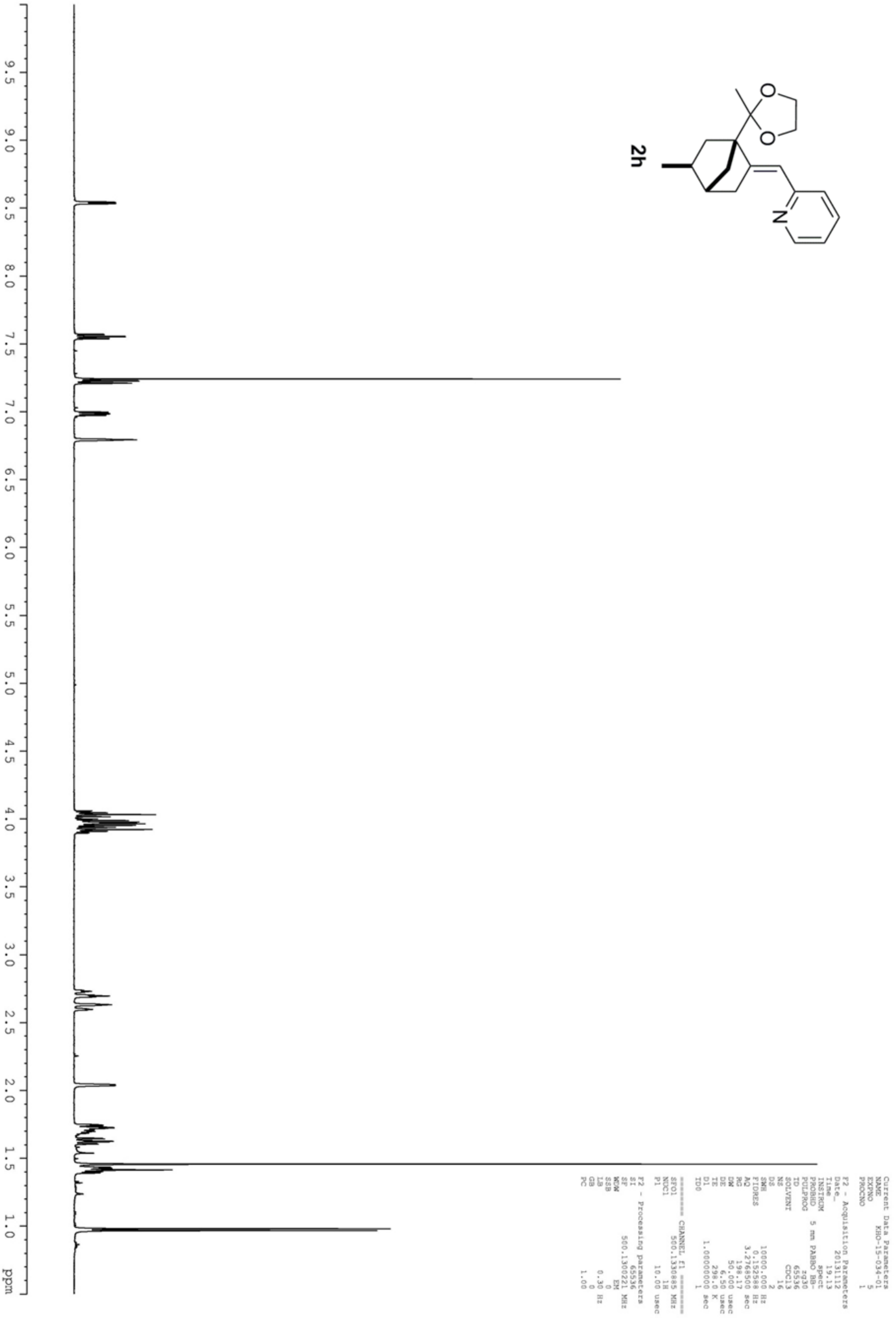
2h
ppm
9.5
9.0
8.5
8.0
7.5
7.0
6.5
6.0
5.5
5.0
4.5
4.0
3.5
3.0
2.5
2.0
1.5
1.0
Current Data Parameters
NAME KHO-15-034-01
EXPNO 5
PROCNO 1
F2 - Acquisition Parameters
Date_ 20131112
Time 19.13
INSTRUM spect
PROBHD 5 mm PABBO BB-
PULPROG zg30
TD 65536
SOLVENT CDCl3
NS 16
DS 2
SWH 10000.000 Hz
FIDRES 0.152588 Hz
AQ 3.2768500 sec
RG 198.17
DW 50.000 usec
DE 6.50 usec
TE 298.0 K
D1 1.00000000 sec
TD0 1
======== CHANNEL f1 ========
SFO1 500.1330885 MHz
NUC1 1H
P1 10.00 usec
F2 - Processing parameters
SI 65536
SF 500.1300221 MHz
WDW EM
SSB 0
LB 0.30 Hz
GB 0
PC 1.00

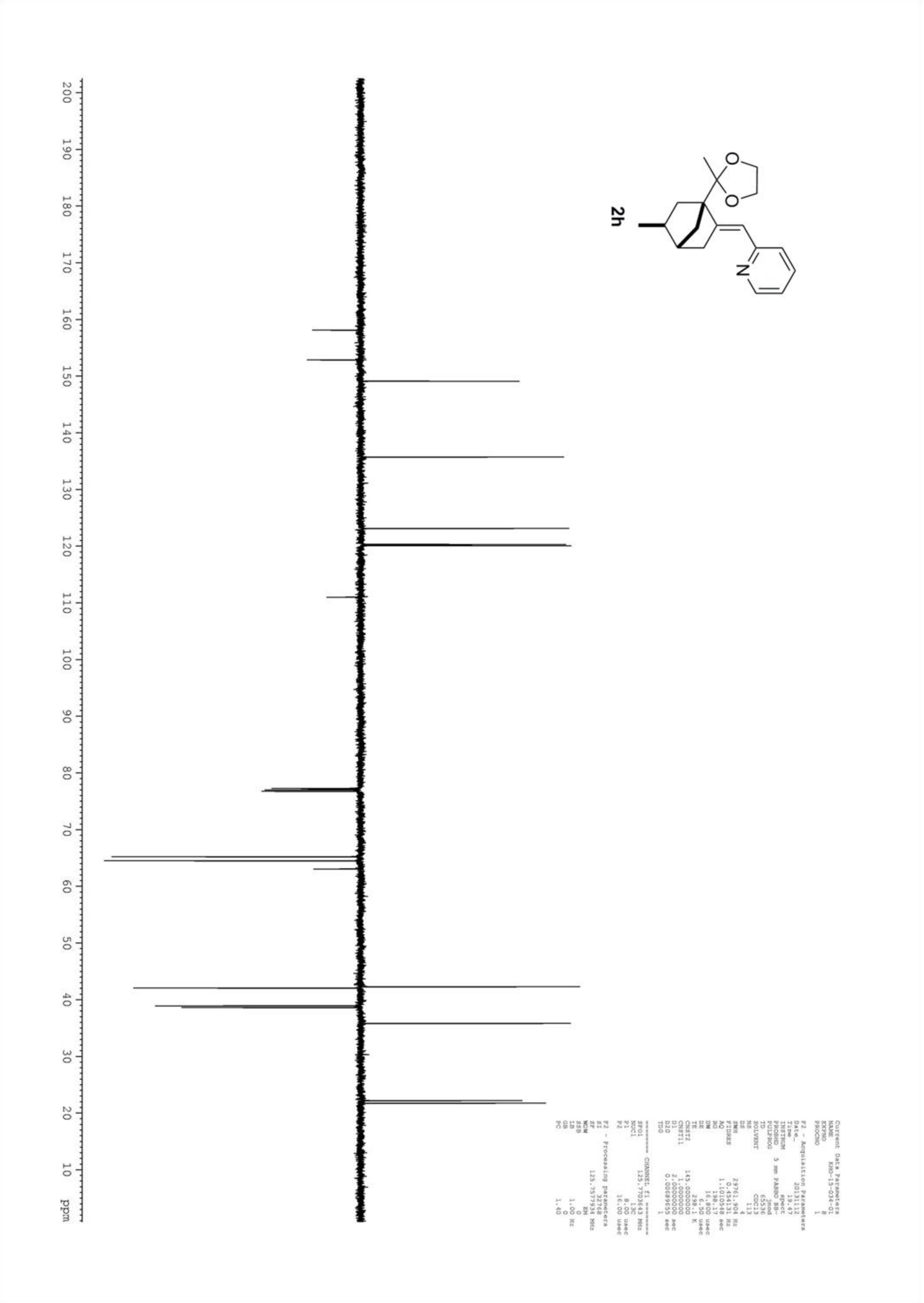
2h
ppm
200
190
180
170
160
150
140
130
120
110
100
90
80
70
60
50
40
30
20
10

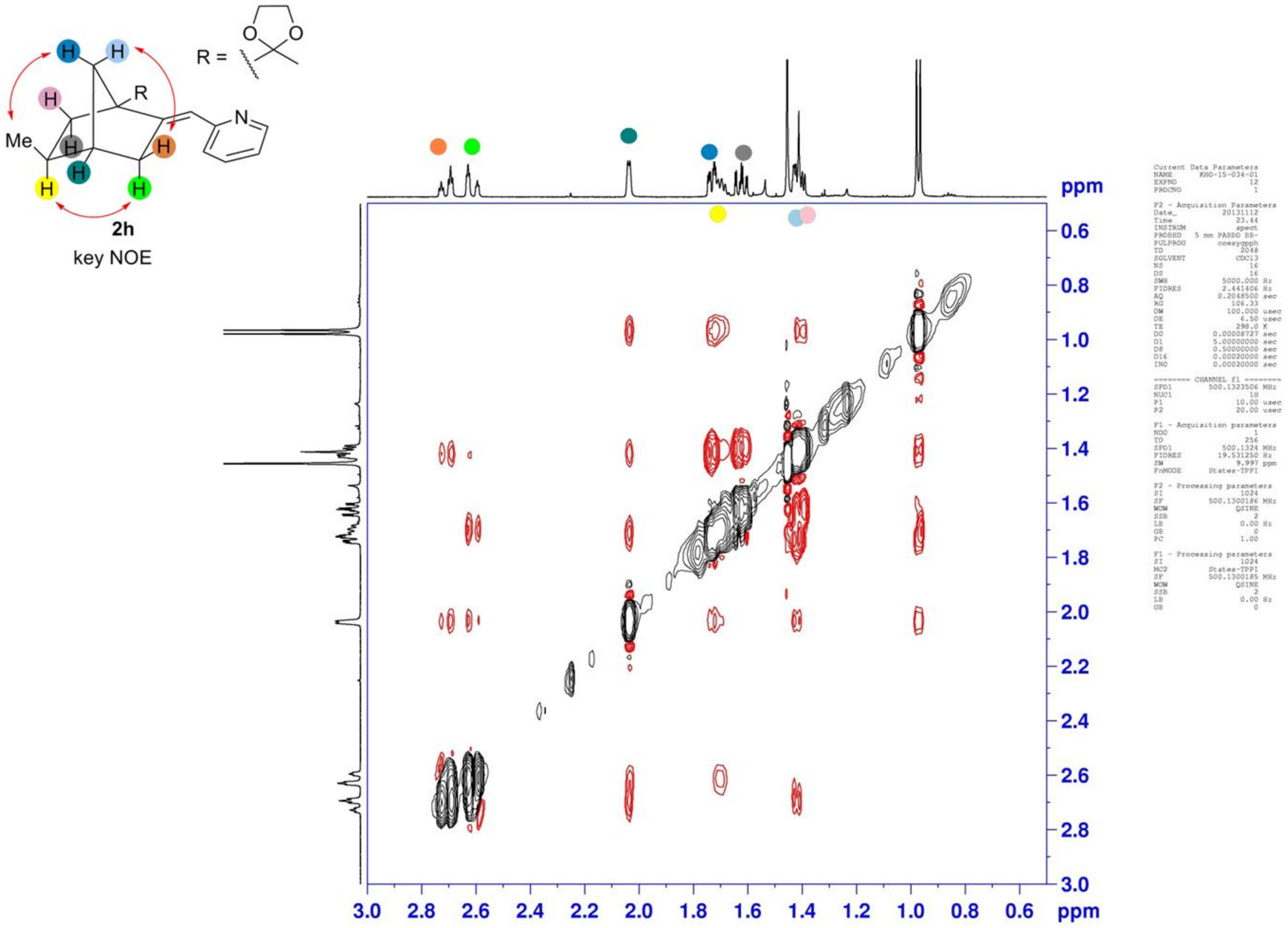
R =
O
O
N
R
Me
H
2h
key NOE
ppm
0.6
0.8
1.0
1.2
1.4
1.6
1.8
2.0
2.2
2.4
2.6
2.8
3.0
Current Data Parameters
NAME KHO-15-034-01
EXPNO 12
PROCNO 1
F2 - Acquisition Parameters
Date_ 20131112
Time 23.44
INSTRUM spect
PROBHD 5 mm PABBO BB-
PULPROG noesygpph
TD 2048
SOLVENT CDCl3
NS 16
DS 16
SWH 5000.000 Hz
FIDRES 2.441406 Hz
AQ 0.2048500 sec
RG 106.33
DW 100.000 usec
DE 6.50 usec
TE 298.0 K
D0 0.00008727 sec
D1 5.00000000 sec
D8 0.50000000 sec
D16 0.00020000 sec
IN0 0.00020000 sec
======== CHANNEL f1 ========
SFO1 500.1323506 MHz
NUC1 1H
P1 10.00 usec
P2 20.00 usec
F1 - Acquisition parameters
ND0 1
TD 256
SFO1 500.1324 MHz
FIDRES 19.531250 Hz
SW 9.997 ppm
FnMODE States-TPPI
F2 - Processing parameters
SI 1024
SF 500.1300186 MHz
WDW QSINE
SSB 2
LB 0.00 Hz
GB 0
PC 1.00
F1 - Processing parameters
SI 1024
MC2 States-TPPI
SF 500.1300185 MHz
WDW QSINE
SSB 2
LB 0.00 Hz
GB 0

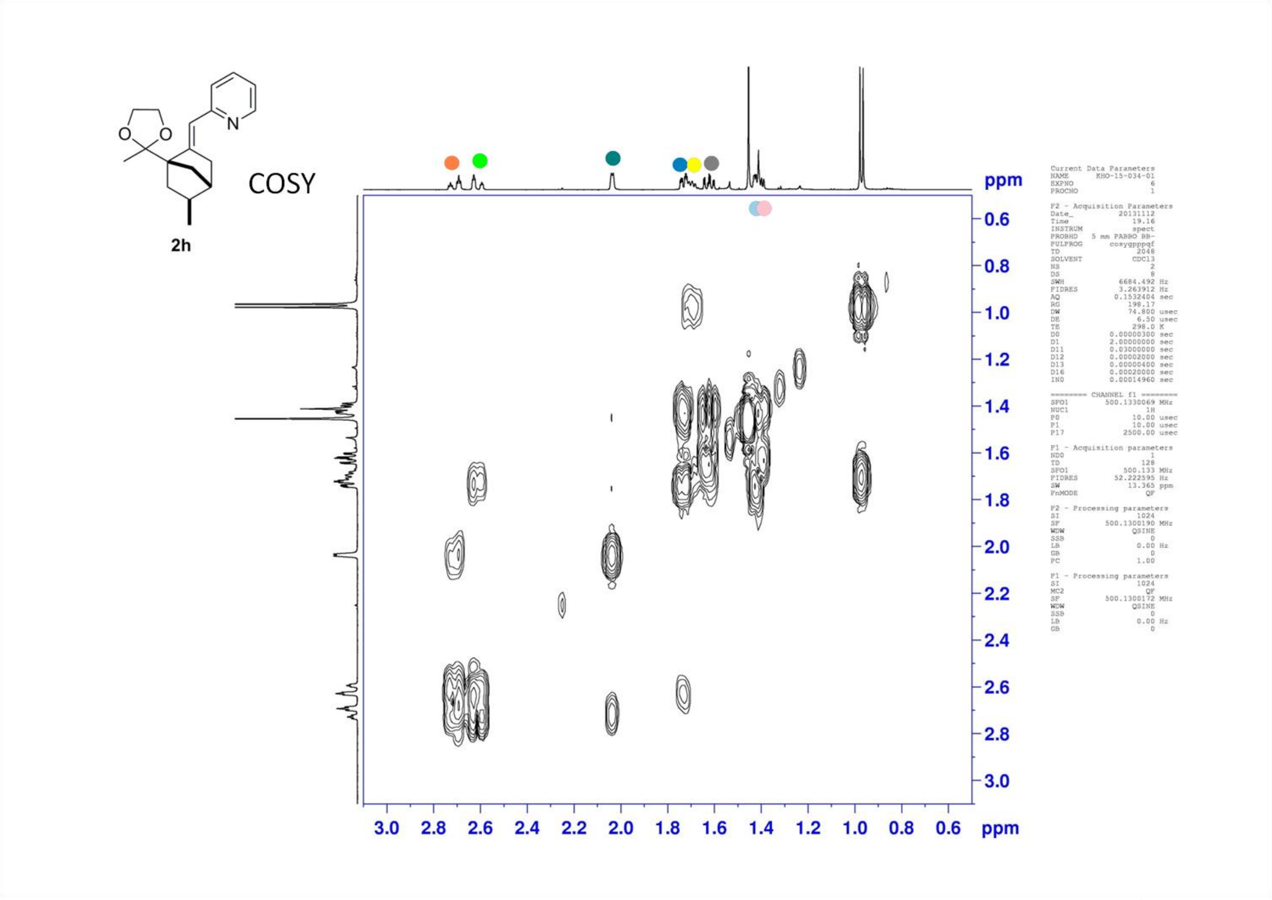
COSY
2h
ppm

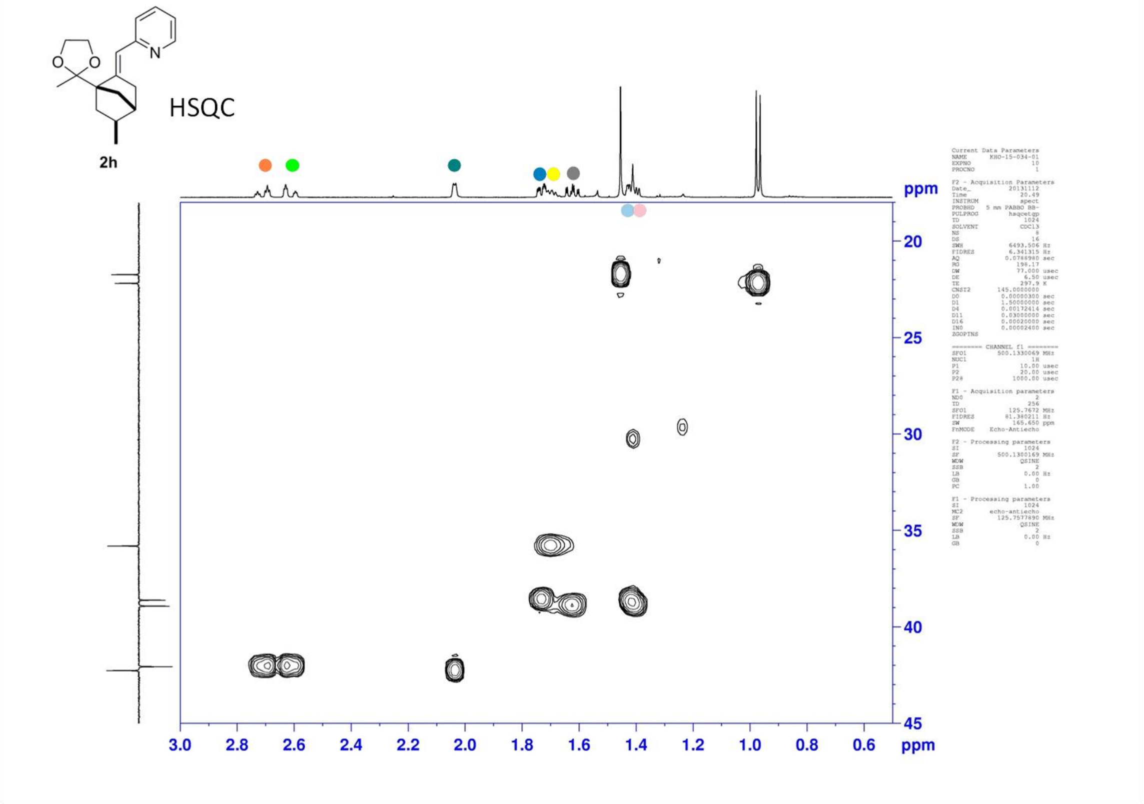
HSQC
2h
ppm

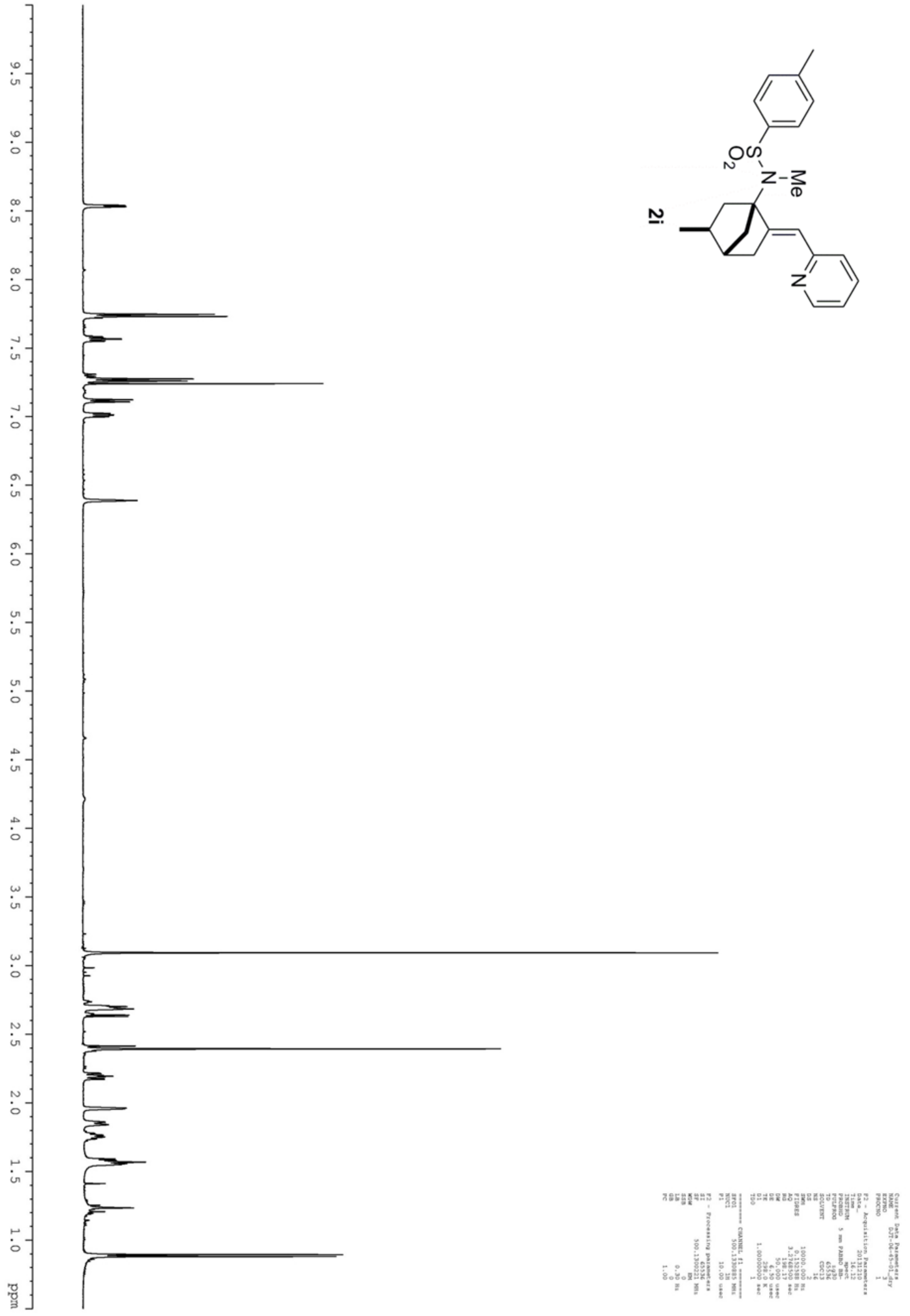

ppm
9.5
9.0
8.5
8.0
7.5
7.0
6.5
6.0
5.5
5.0
4.5
4.0
3.5
3.0
2.5
2.0
1.5
1.0
2i
Me

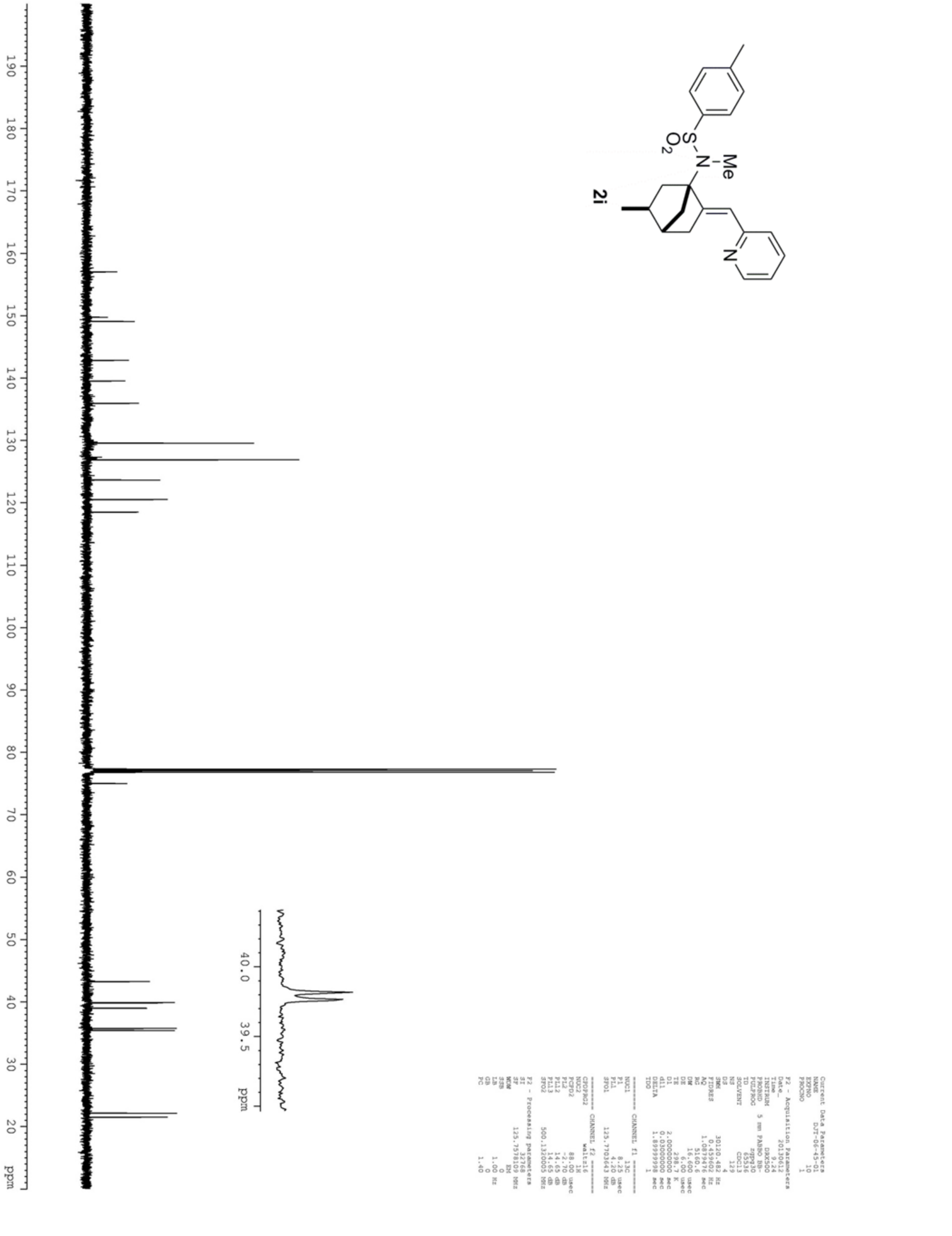
2i
Me
N
ppm

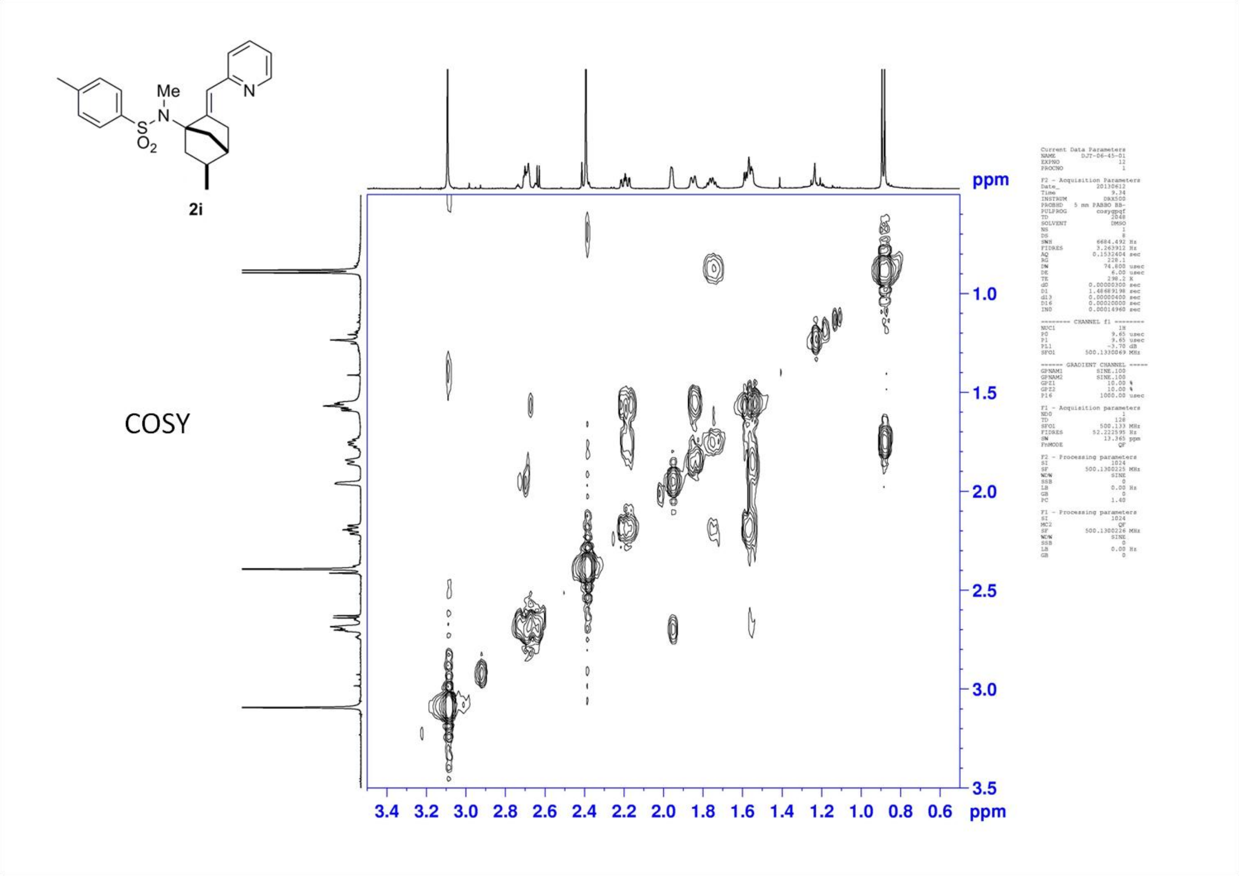
Me
N
N
S
O2
2i
COSY
ppm

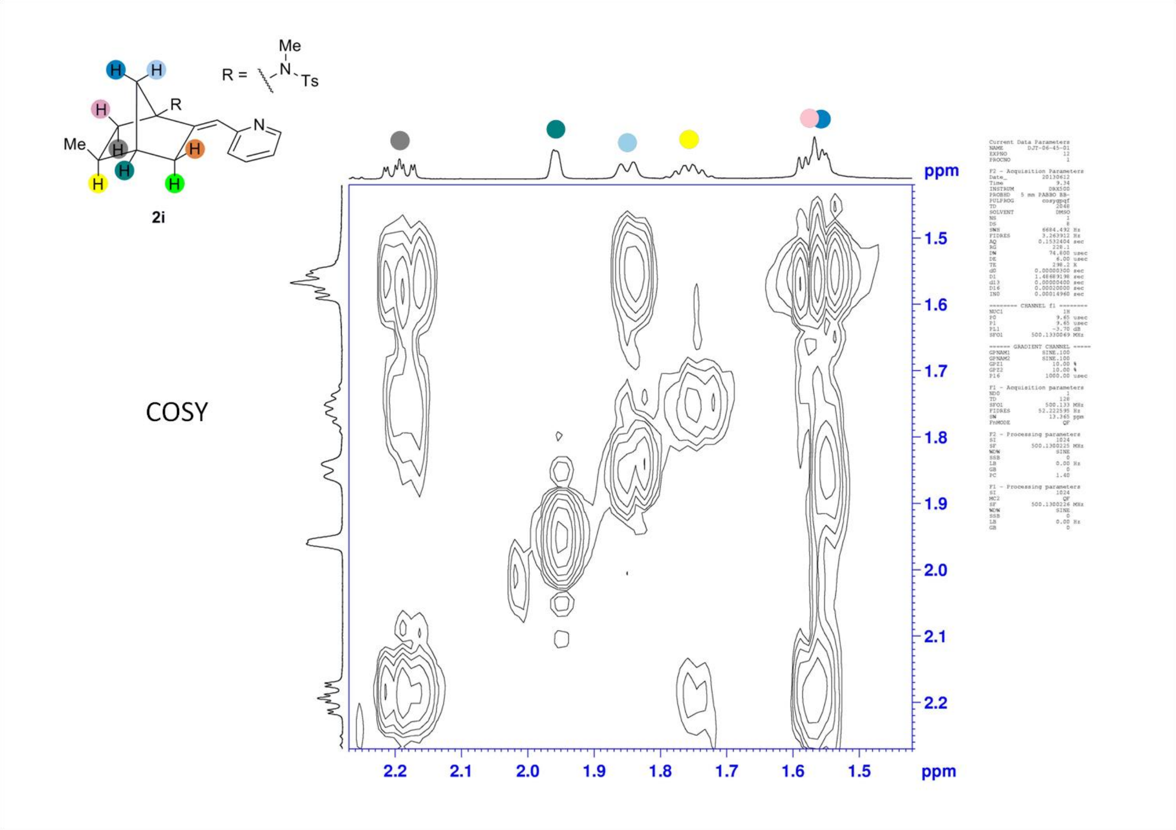
Me
N
Ts
R =
R
H
Me
N
2i
COSY
ppm

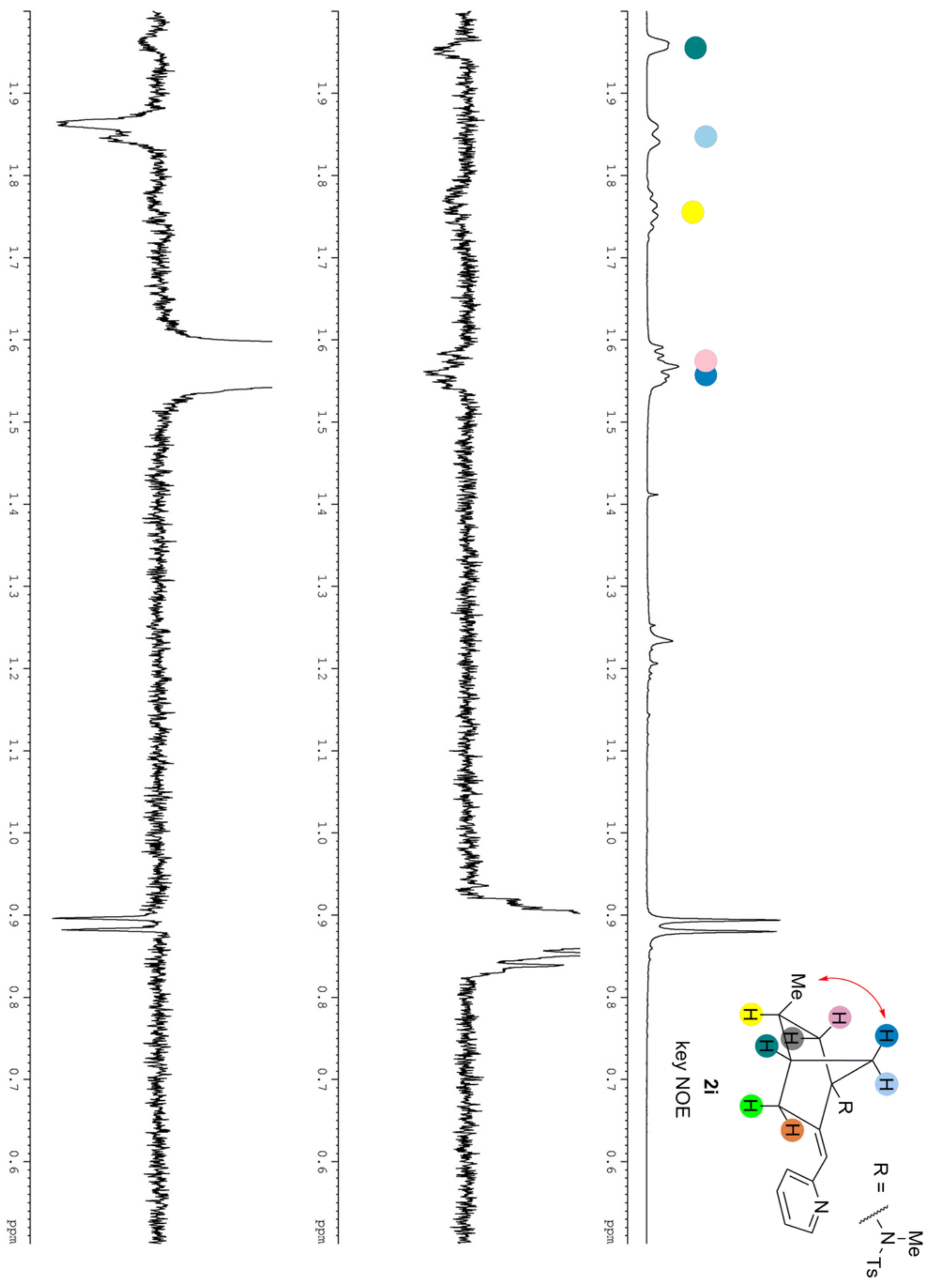
2i
key NOE
R =
Me
N
Ts
ppm

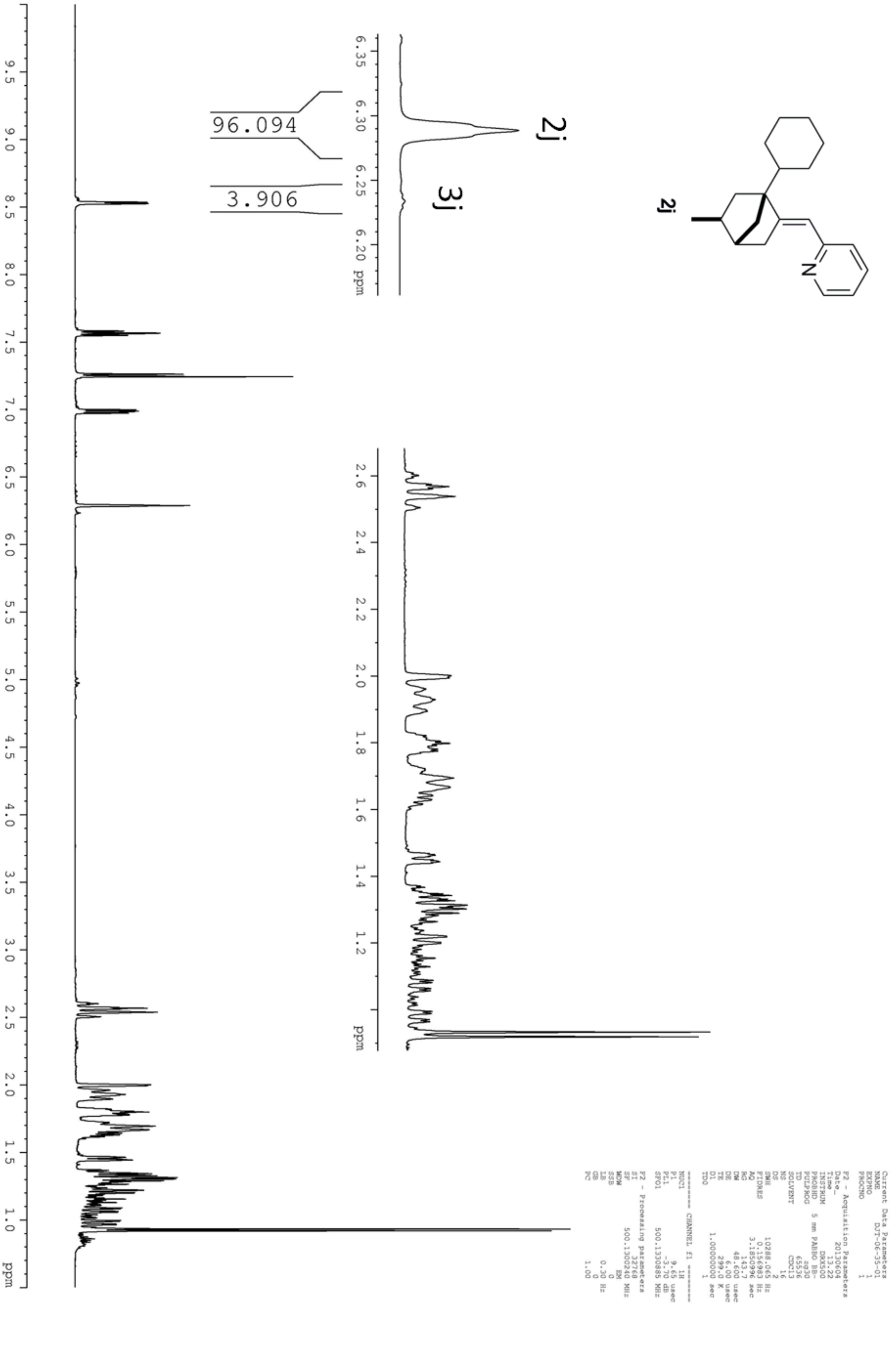
2j
3j
96.094
3.906
2j
ppm

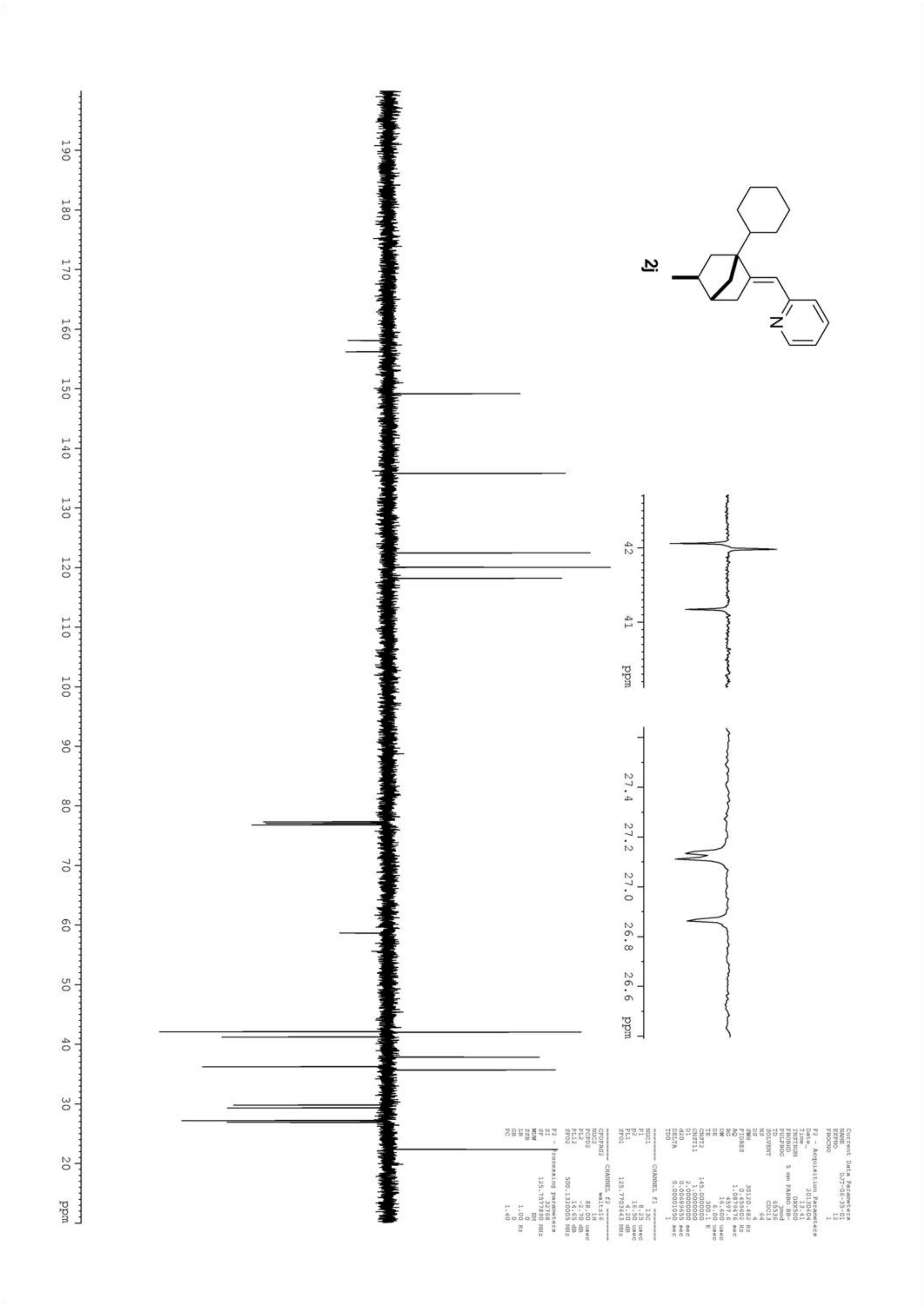
2j
ppm

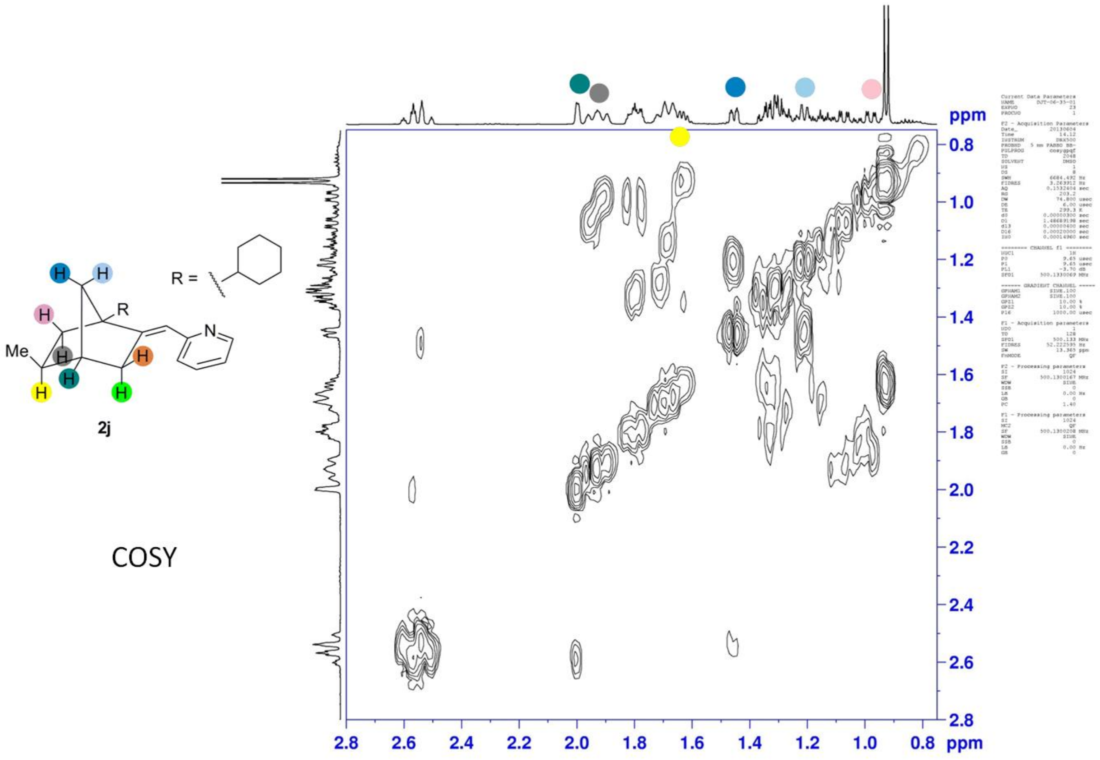
R =
R
N
Me
2j
COSY
ppm

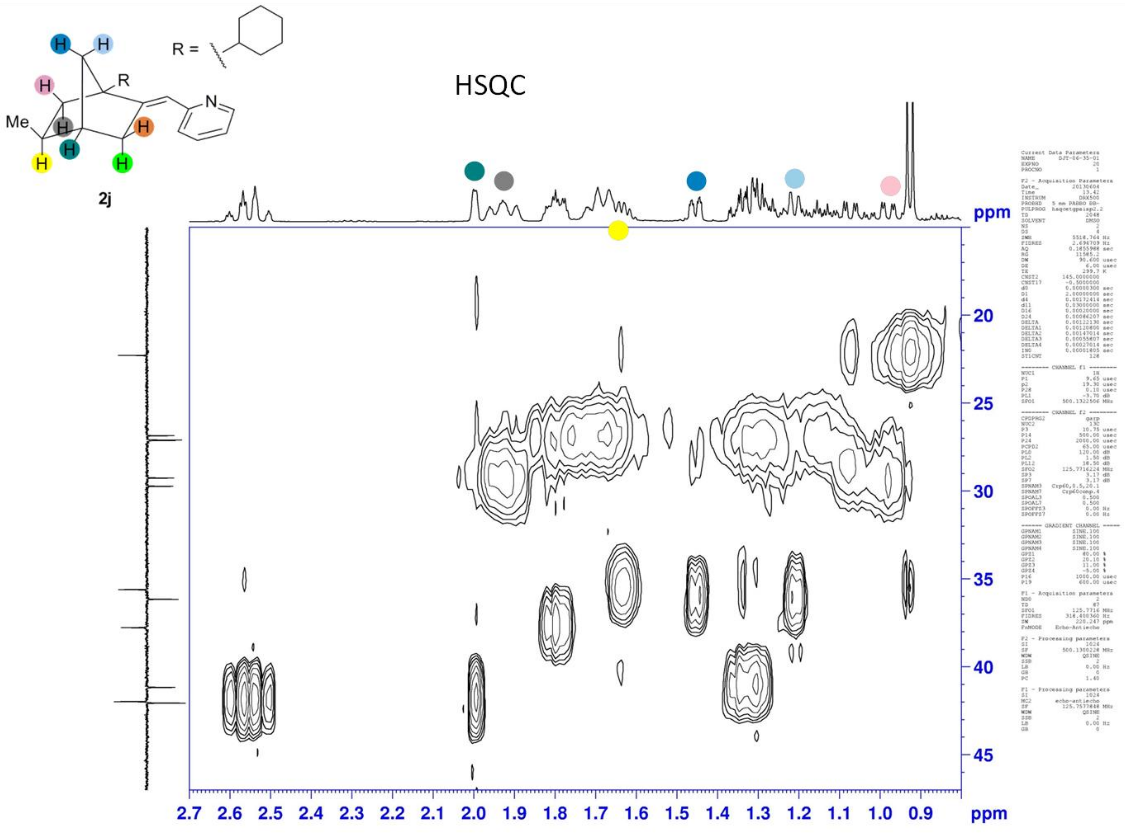
R =
R
N
Me
2j
HSQC
ppm

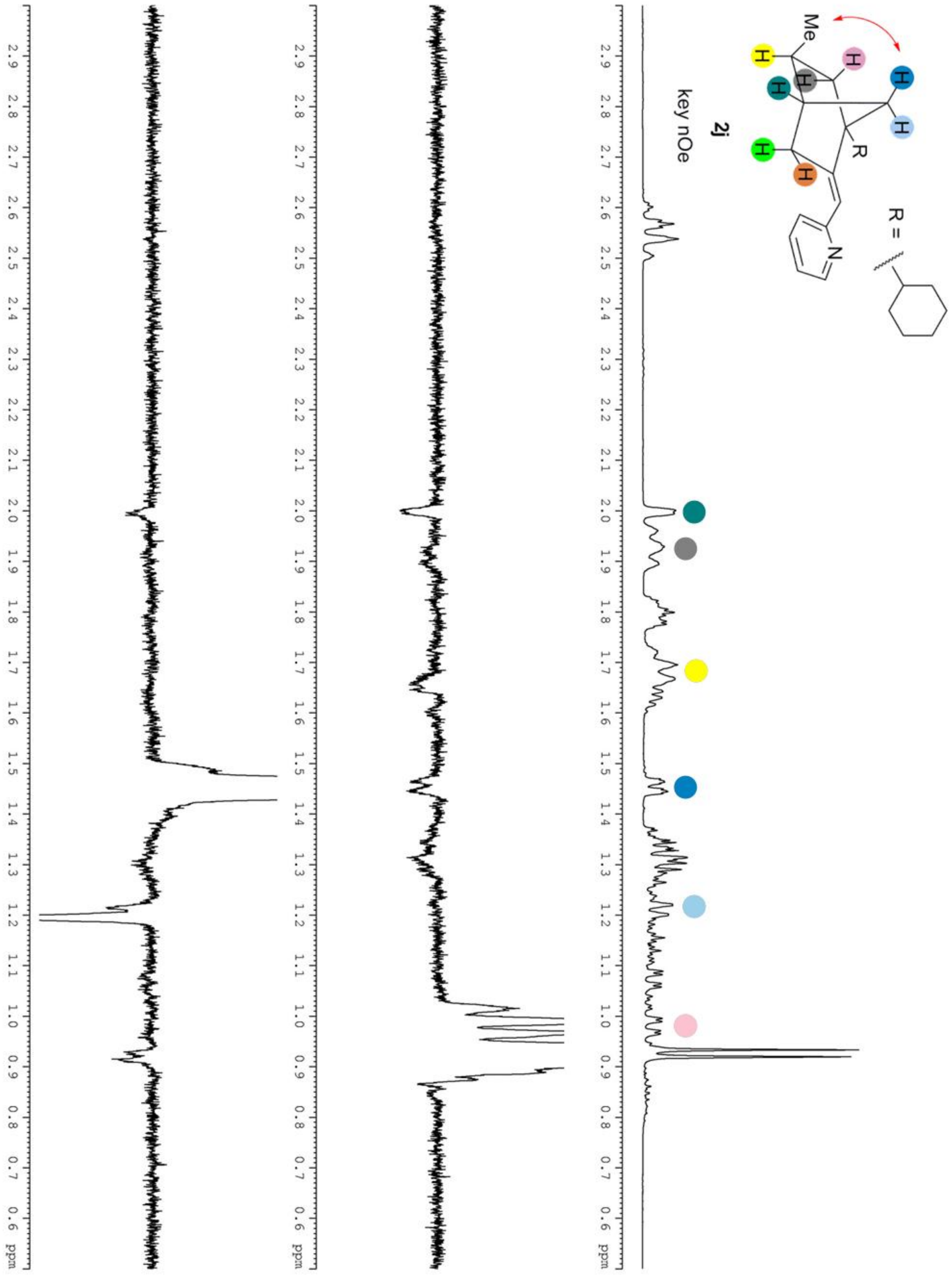
R =
Me
R
N
2j
key nOe
ppm

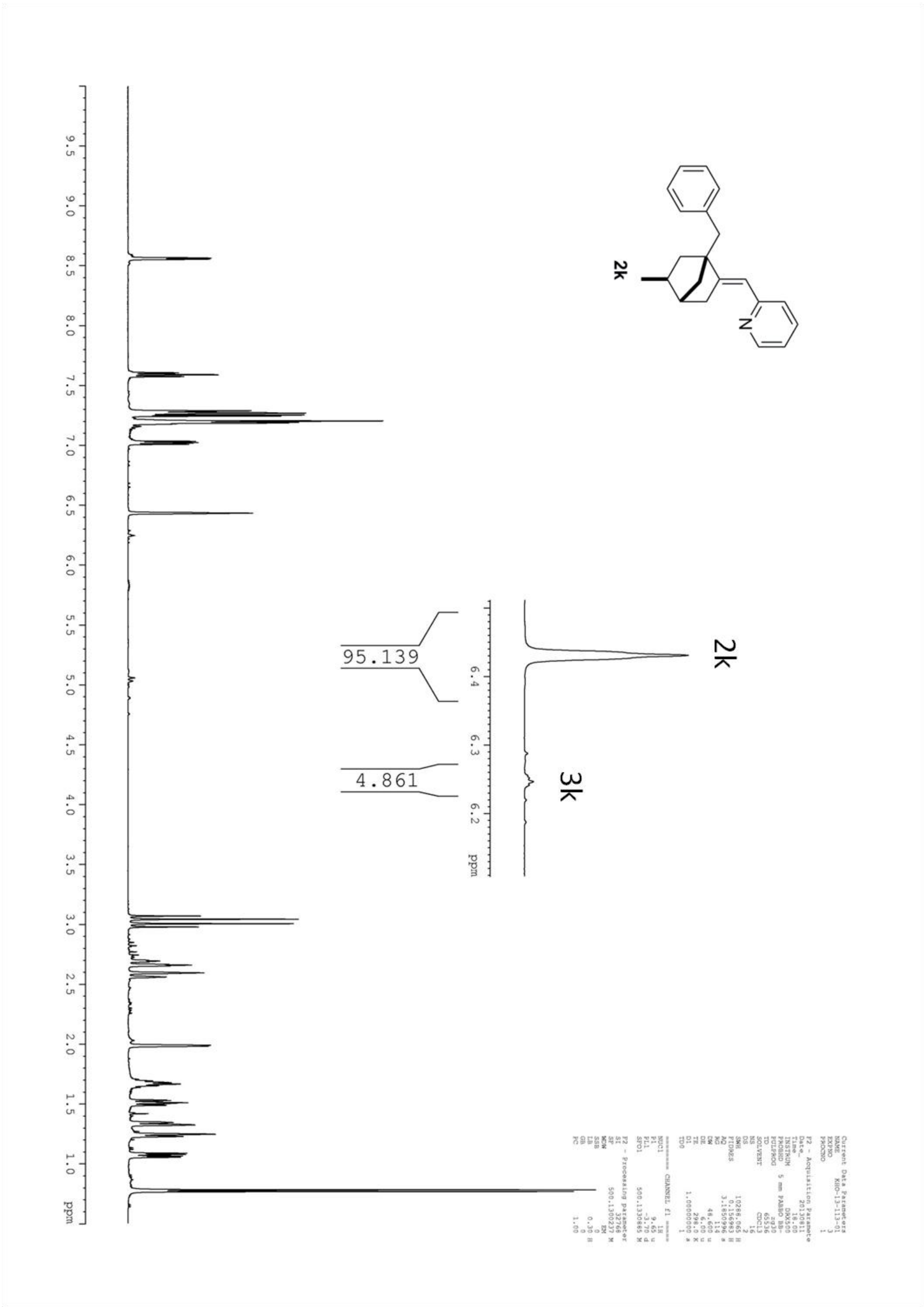
2k
2k
3k
95.139
4.861
ppm
ppm

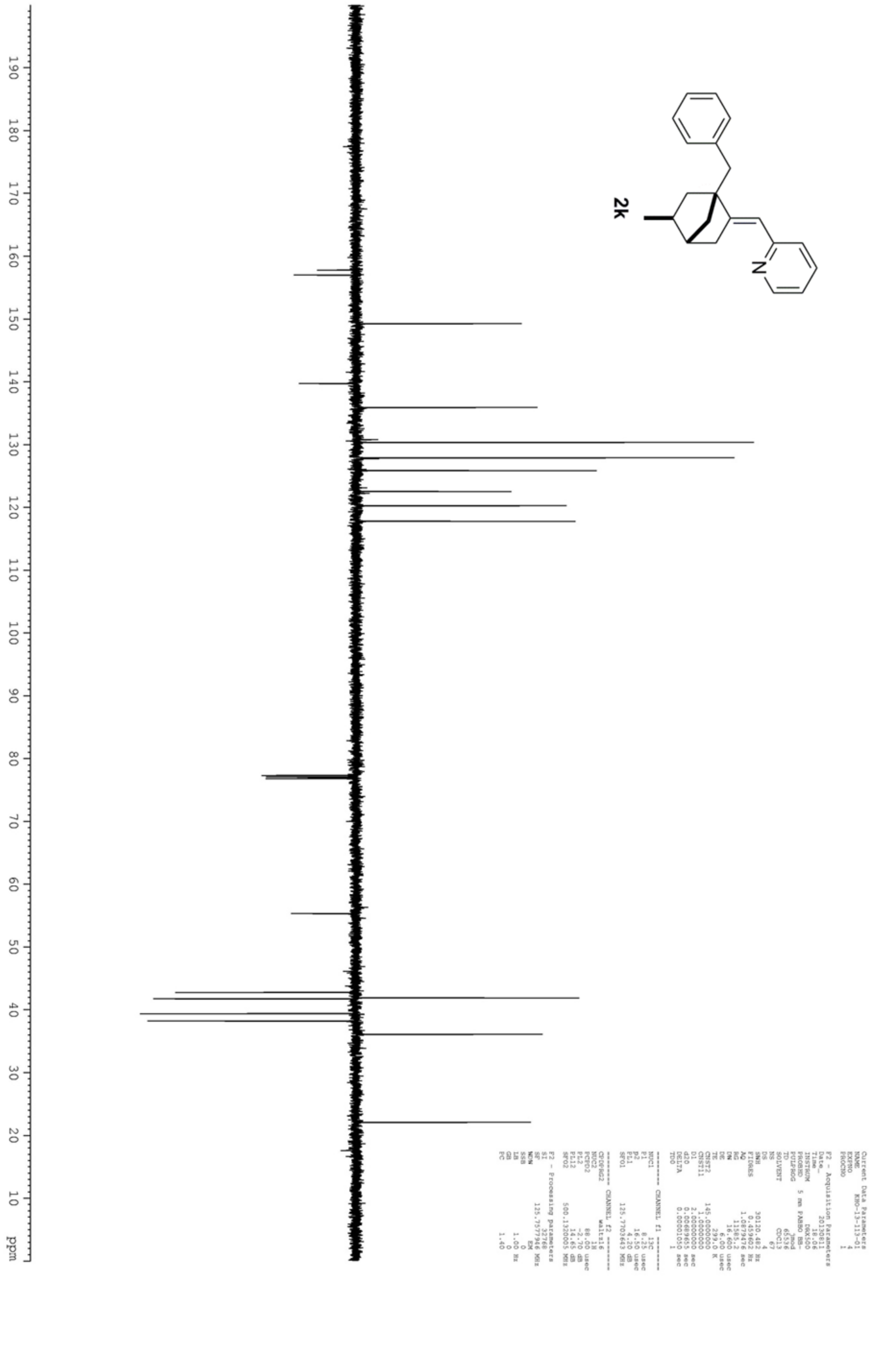

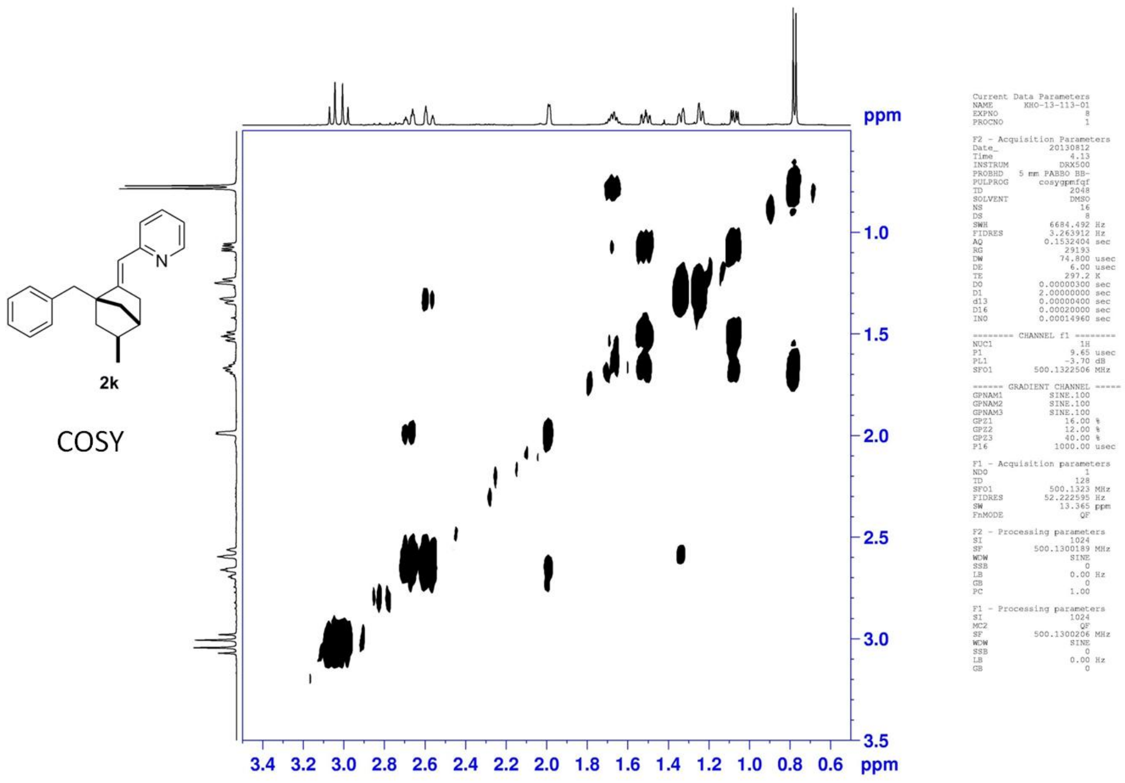
N
2k
COSY
ppm

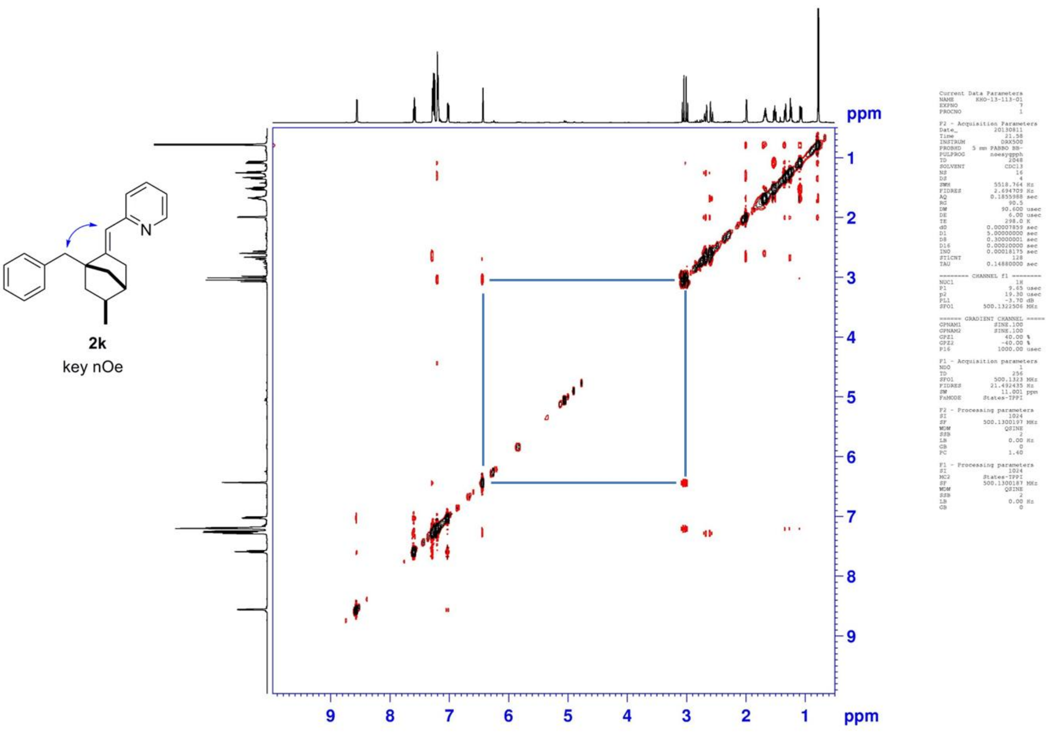
N
2k
key nOe
ppm

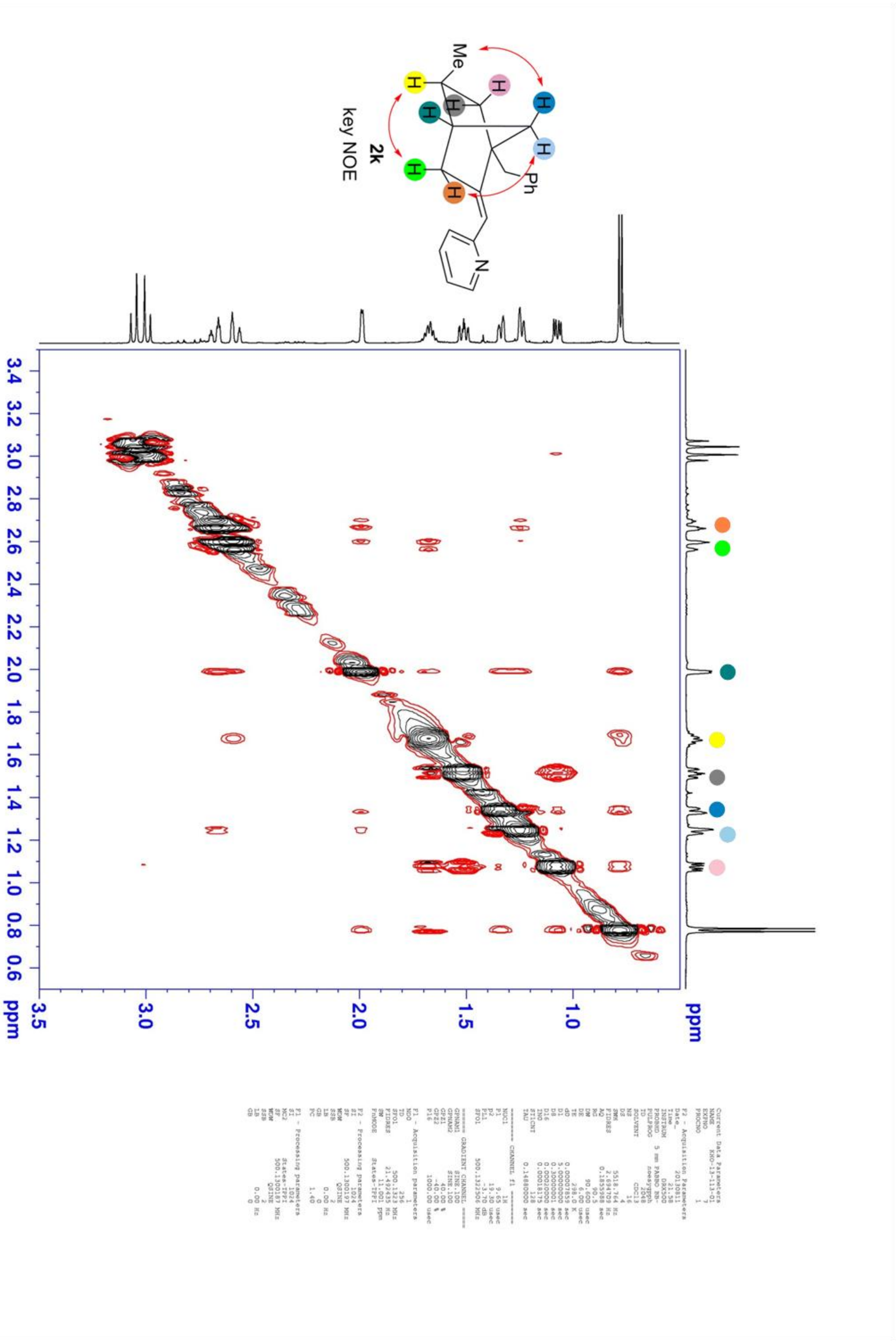
2k
key NOE
Me
Ph
N
ppm
ppm

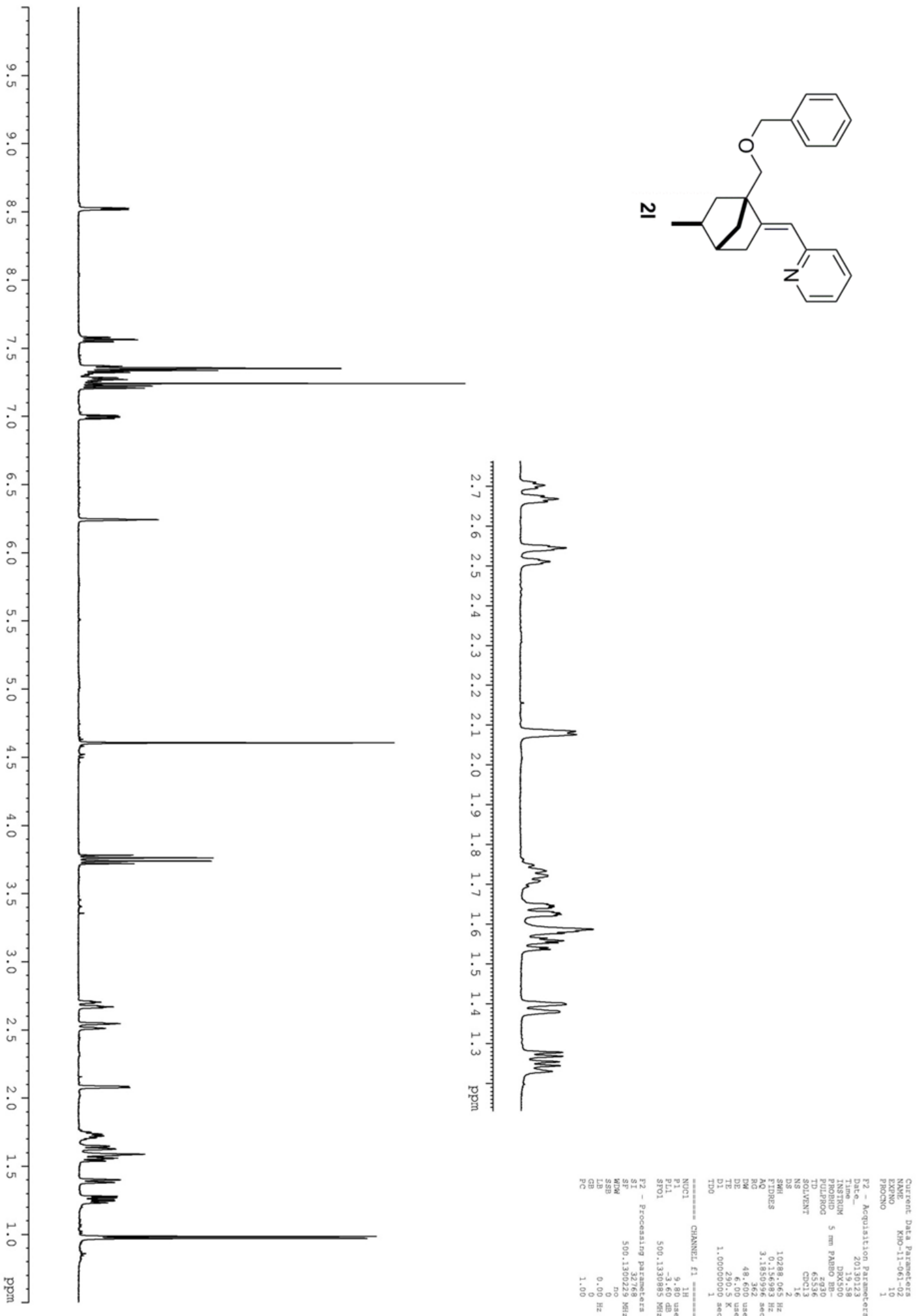
2l
ppm

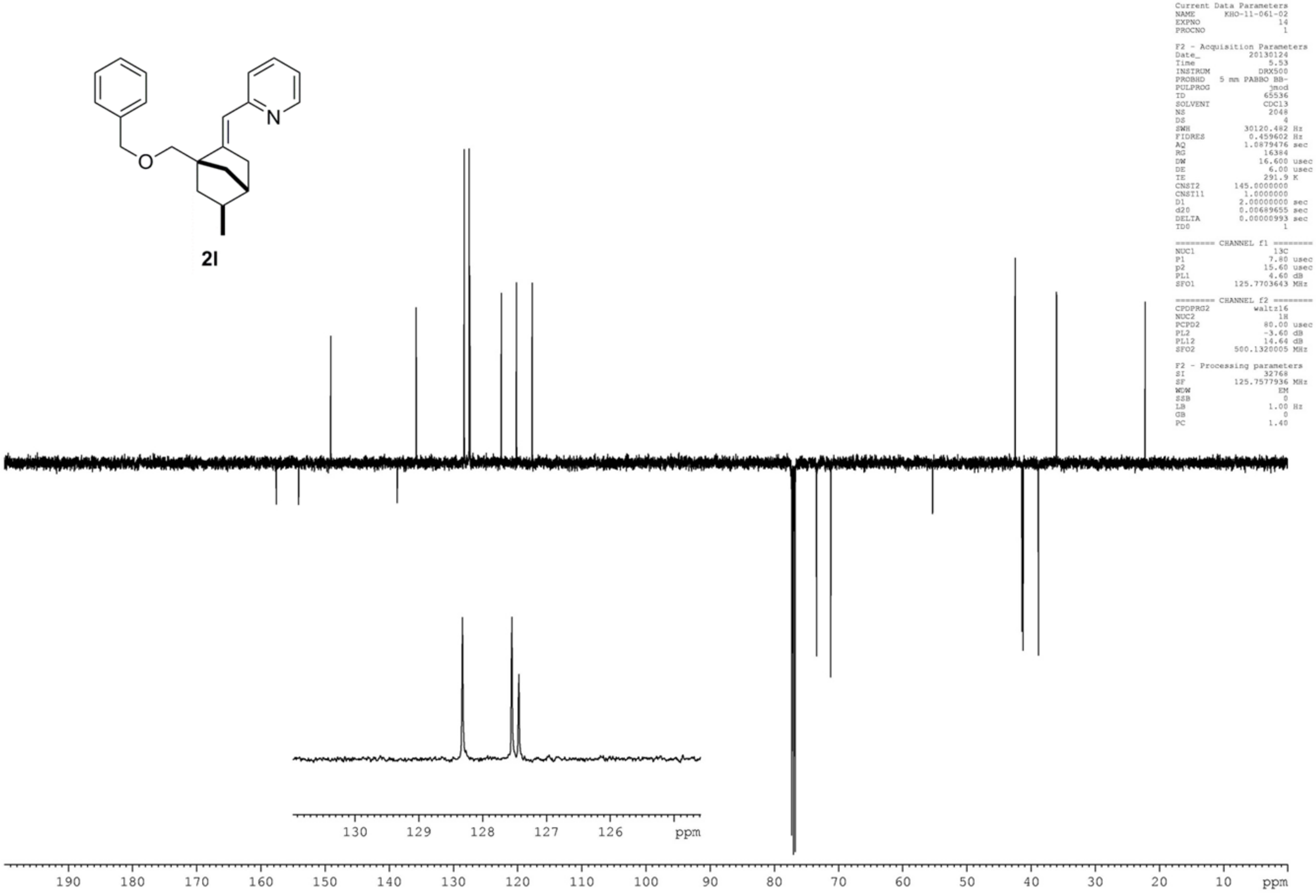
2l
Current Data Parameters
NAME KHO-11-061-02
EXPNO 14
PROCNO 1
F2 - Acquisition Parameters
Date_ 20130124
Time 5.53
INSTRUM DRX500
PROBHD 5 mm PABBO BB-
PULPROG jmod
TD 65536
SOLVENT CDCl3
NS 2048
DS 4
SWH 30120.482 Hz
FIDRES 0.459602 Hz
AQ 1.0879476 sec
RG 16384
DW 16.600 usec
DE 6.00 usec
TE 291.9 K
CNST2 145.0000000
CNST11 1.0000000
D1 2.00000000 sec
d20 0.00689655 sec
DELTA 0.00000993 sec
TD0 1
======== CHANNEL f1 ========
NUC1 13C
P1 7.80 usec
p2 15.60 usec
PL1 4.60 dB
SFO1 125.7703643 MHz
======== CHANNEL f2 ========
CPDPRG2 waltz16
NUC2 1H
PCPD2 80.00 usec
PL2 -3.60 dB
PL12 14.64 dB
SFO2 500.1320005 MHz
F2 - Processing parameters
SI 32768
SF 125.7577936 MHz
WDW EM
SSB 0
LB 1.00 Hz
GB 0
PC 1.40
130 129 128 127 126 ppm
190 180 170 160 150 140 130 120 110 100 90 80 70 60 50 40 30 20 10 ppm

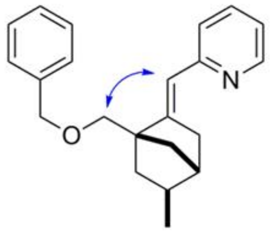
N
O

2l

key nOe

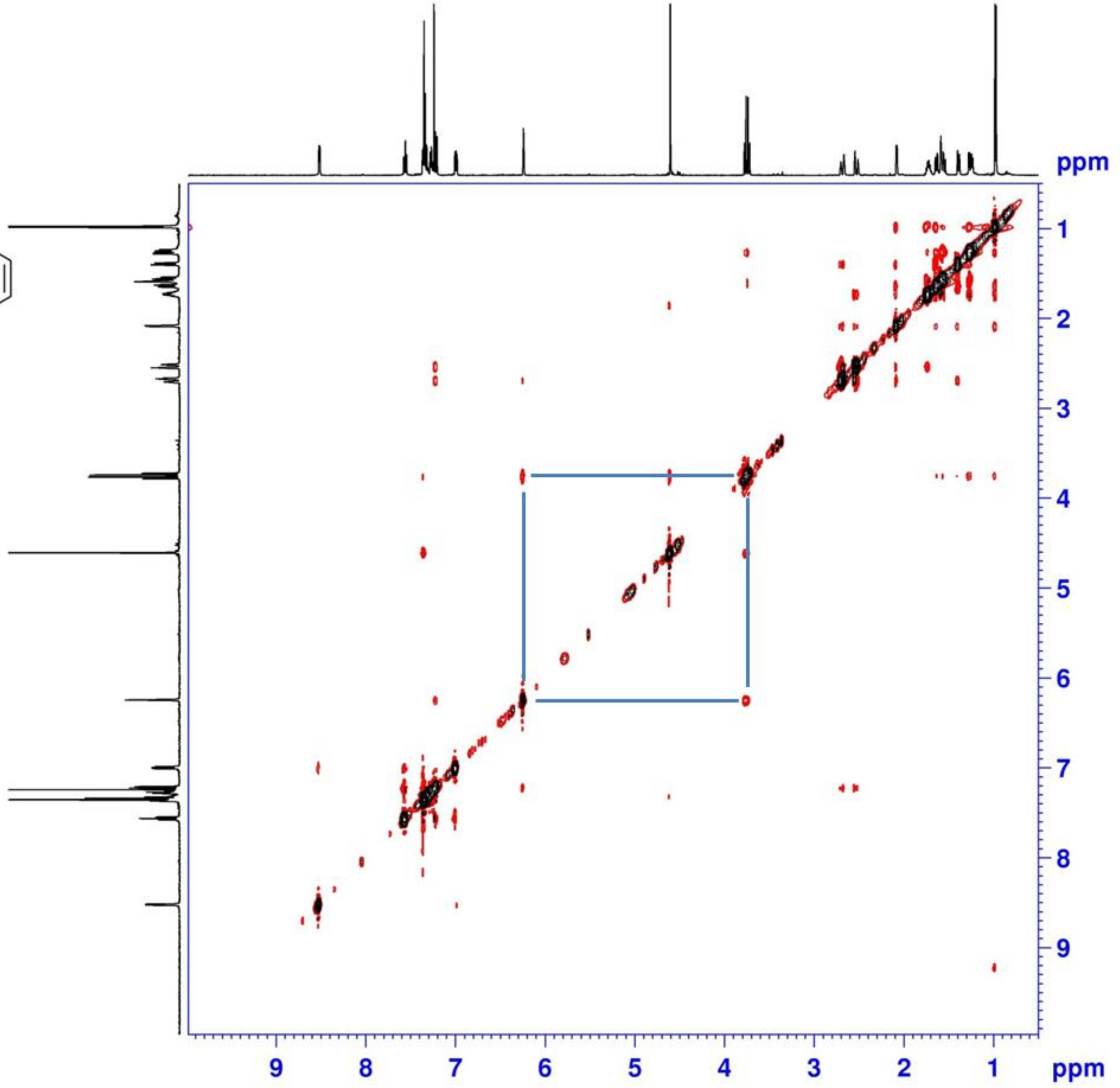
ppm
1
2
3
4
5
6
7
8
9
9
8
7
6
5
4
3
2
1
ppm
Current Data Parameters
NAME KHO-11-061-02
EXPNO 13
PROCNO 1
F2 - Acquisition Parameters
Date_ 20130123
Time 22.26
INSTRUM DRX500
PROBHD 5 mm PABBO BB-
PULPROG noesygpph
TD 2048
SOLVENT CDCl3
NS 32
DS 4
SWH 5518.764 Hz
FIDRES 2.694709 Hz
AQ 0.1855988 sec
RG 322.5
DW 90.600 usec
DE 6.00 usec
TE 290.2 K
d0 0.00007840 sec
D1 2.00000000 sec
D8 0.30000001 sec
D16 0.00020000 sec
IN0 0.00018175 sec
ST1CNT 128
TAU 0.14880000 sec
======== CHANNEL f1 ========
NUC1 1H
P1 9.80 usec
p2 19.60 usec
PL1 -3.60 dB
SFO1 500.1322506 MHz
====== GRADIENT CHANNEL =====
GPNAM1 SINE.100
GPNAM2 SINE.100
GPZ1 40.00 %
GPZ2 -40.00 %
P16 1000.00 usec
F1 - Acquisition parameters
ND0 1
TD 256
SFO1 500.1323 MHz
FIDRES 21.492435 Hz
SW 11.001 ppm
FnMODE States-TPPI
F2 - Processing parameters
SI 1024
SF 500.1300180 MHz
WDW QSINE
SSB 2
LB 0.00 Hz
GB 0
PC 1.40
F1 - Processing parameters
SI 1024
MC2 States-TPPI
SF 500.1300178 MHz
WDW QSINE
SSB 2
LB 0.00 Hz
GB 0

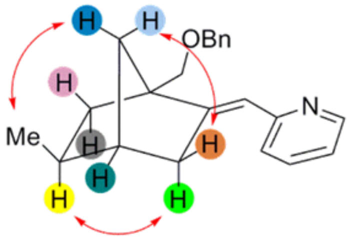

2l
key NOE

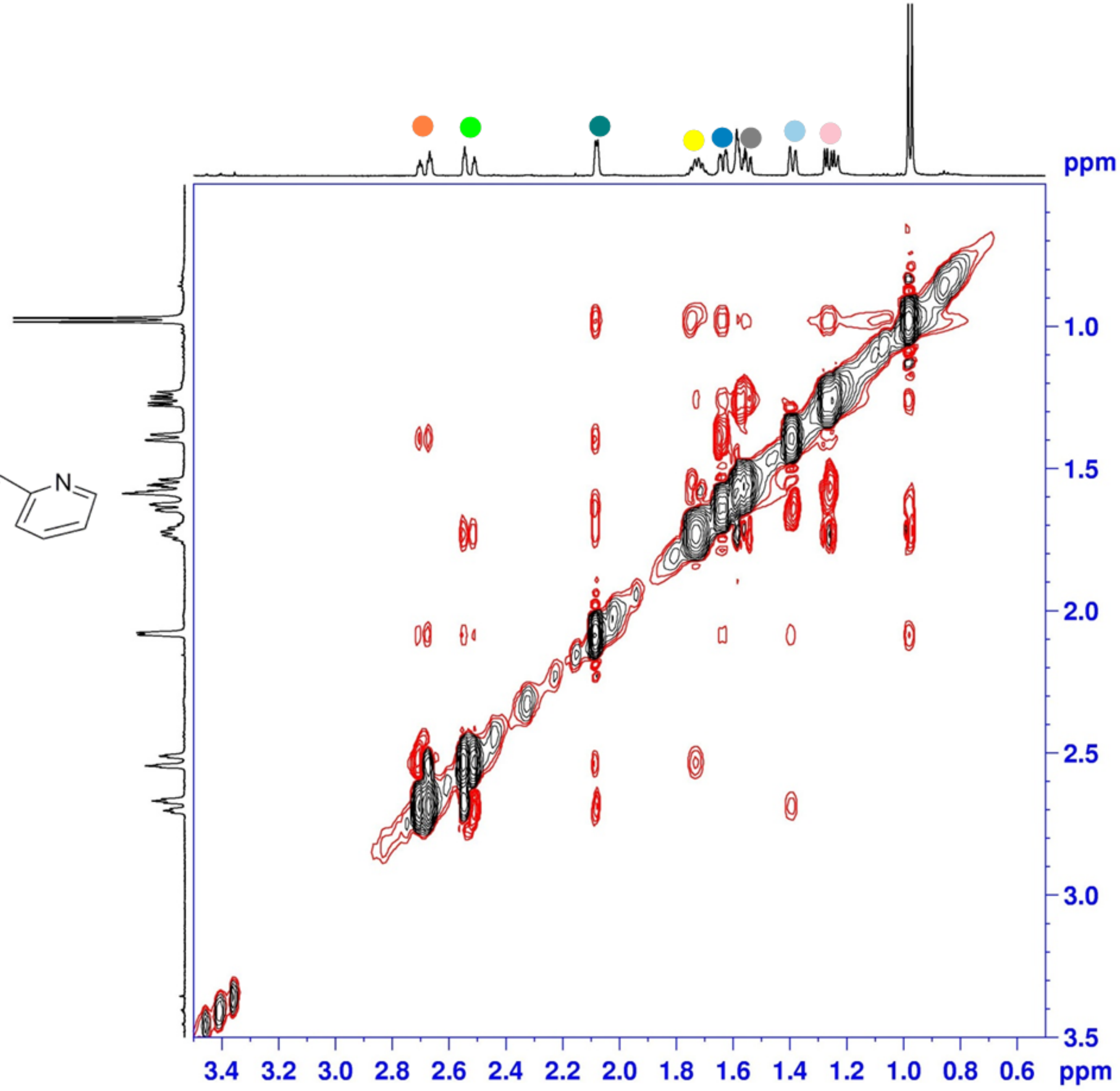


```
Current Data Parameters
NAME          KHO-11-061-02
EXPNO                    13
PROCNO                    1

F2 - Acquisition Parameters
Date_              20130123
Time                  22.26
INSTRUM              DRX500
PROBHD      5 mm PABBO BB-
PULPROG          noesygpph
TD                     2048
SOLVENT               CDCl3
NS                       32
DS                        4
SWH            5518.764 Hz
FIDRES         2.694709 Hz
AQ            0.1855988 sec
RG                    322.5
DW               90.600 usec
DE                 6.00 usec
TE                  290.2 K
d0           0.00007840 sec
D1           2.00000000 sec
D8           0.30000001 sec
D16          0.00020000 sec
IN0          0.00018175 sec
ST1CNT                  128
TAU          0.14880000 sec

======== CHANNEL f1 ========
NUC1                     1H
P1                9.80 usec
p2               19.60 usec
PL1                -3.60 dB
SFO1         500.1322506 MHz

====== GRADIENT CHANNEL =====
GPNAM1            SINE.100
GPNAM2            SINE.100
GPZ1                 40.00 %
GPZ2                -40.00 %
P16             1000.00 usec

F1 - Acquisition parameters
ND0                       1
TD                      256
SFO1           500.1323 MHz
FIDRES        21.492435 Hz
SW               11.001 ppm
FnMODE          States-TPPI

F2 - Processing parameters
SI                     1024
SF          500.1300180 MHz
WDW                   QSINE
SSB                       2
LB                  0.00 Hz
GB                        0
PC                     1.40

F1 - Processing parameters
SI                     1024
MC2             States-TPPI
SF          500.1300178 MHz
WDW                   QSINE
SSB                       2
LB                  0.00 Hz
GB                        0
```

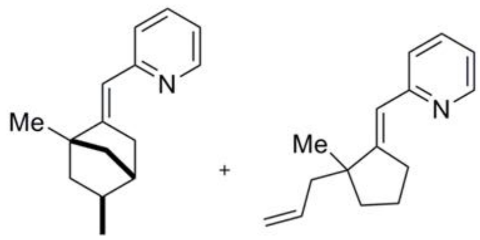
Me
N
Me
N
+

2m **3m**

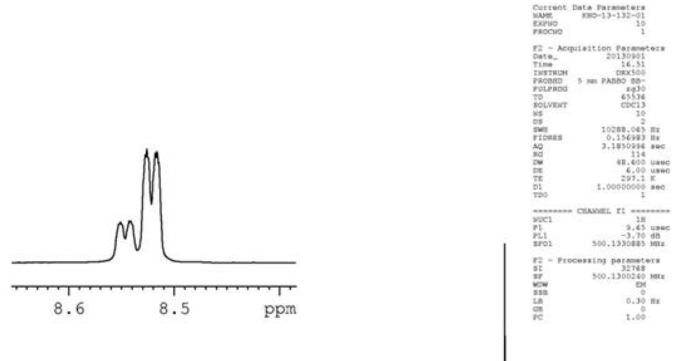
Current Data Parameters
NAME KHO-13-132-01
EXPNO 10
PROCNO 1
F2 - Acquisition Parameters
Date_ 20130901
Time 16.51
INSTRUM DRX500
PROBHD 5 mm PABBO BB-
PULPROG zg30
TD 65536
SOLVENT CDCl3
NS 10
DS 2
SWH 10288.065 Hz
FIDRES 0.156983 Hz
AQ 3.1850996 sec
RG 114
DW 48.600 usec
DE 6.00 usec
TE 297.1 K
D1 1.00000000 sec
TD0 1
======== CHANNEL f1 ========
NUC1 1H
P1 9.65 usec
PL1 -3.70 dB
SFO1 500.1330885 MHz
F2 - Processing parameters
SI 32768
SF 500.1300240 MHz
WDW EM
SSB 0
LB 0.30 Hz
GB 0
PC 1.00
8.6
8.5
ppm

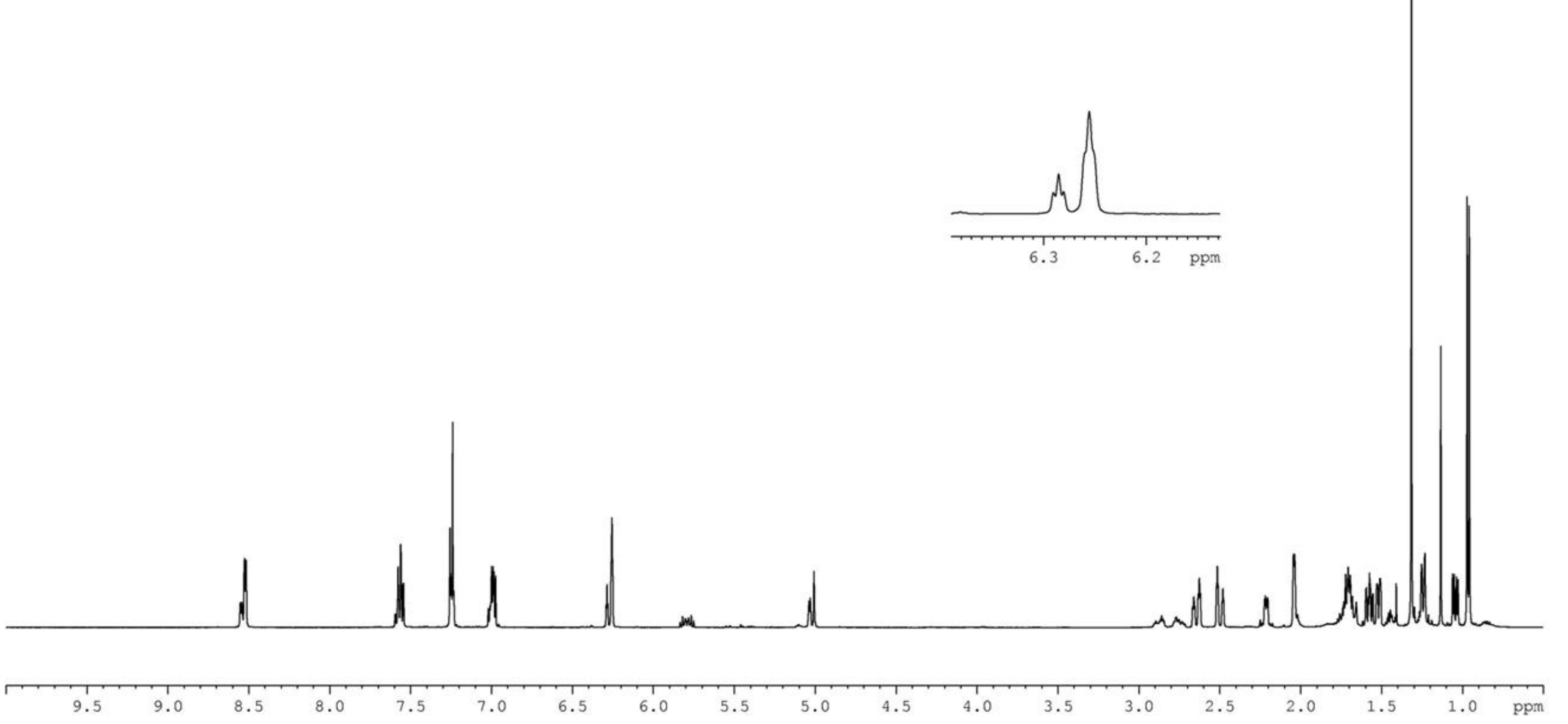
6.3
6.2
ppm
9.5
9.0
8.5
8.0
7.5
7.0
6.5
6.0
5.5
5.0
4.5
4.0
3.5
3.0
2.5
2.0
1.5
1.0
ppm

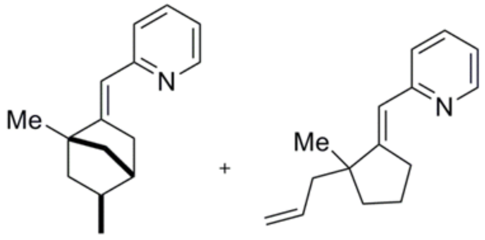

2m + **3m**

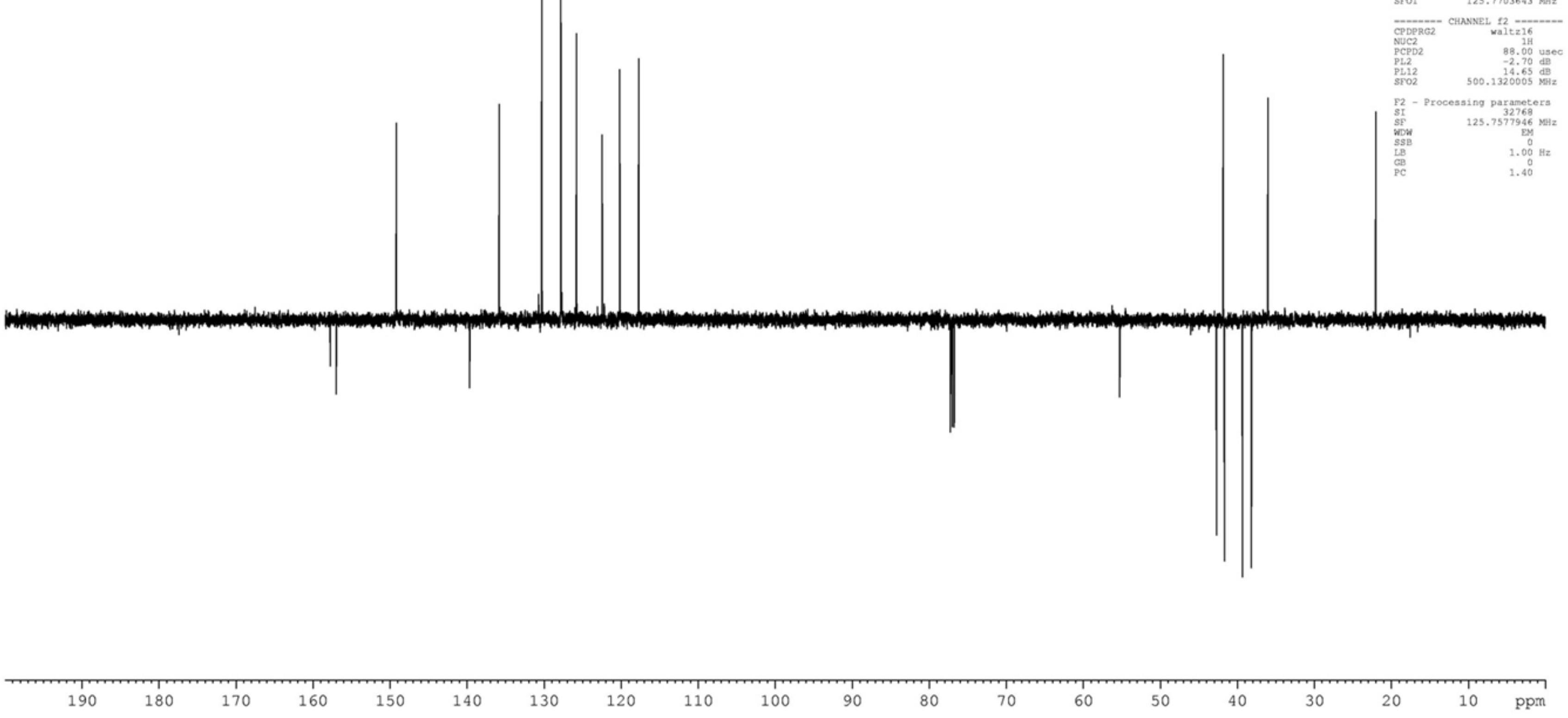

Current Data Parameters
NAME KHO-13-113-01
EXPNO 4
PROCNO 1
F2 - Acquisition Parameters
Date_ 20130811
Time 18.06
INSTRUM DRX500
PROBHD 5 mm PABBO BB-
PULPROG jmod
TD 65536
SOLVENT CDCl3
NS 67
DS 4
SWH 30120.482 Hz
FIDRES 0.459602 Hz
AQ 1.0879476 sec
RG 11585.2
DW 16.600 usec
DE 6.00 usec
TE 299.0 K
CNST2 145.0000000
CNST11 1.0000000
D1 2.00000000 sec
d20 0.00689655 sec
DELTA 0.00001050 sec
TD0 1
======== CHANNEL f1 ========
NUC1 13C
P1 8.25 usec
p2 16.50 usec
PL1 4.20 dB
SFO1 125.7703643 MHz
======== CHANNEL f2 ========
CPDPRG2 waltz16
NUC2 1H
PCPD2 88.00 usec
PL2 -2.70 dB
PL12 14.65 dB
SFO2 500.1320005 MHz
F2 - Processing parameters
SI 32768
SF 125.7577946 MHz
WDW EM
SSB 0
LB 1.00 Hz
GB 0
PC 1.40
190 180 170 160 150 140 130 120 110 100 90 80 70 60 50 40 30 20 10 ppm

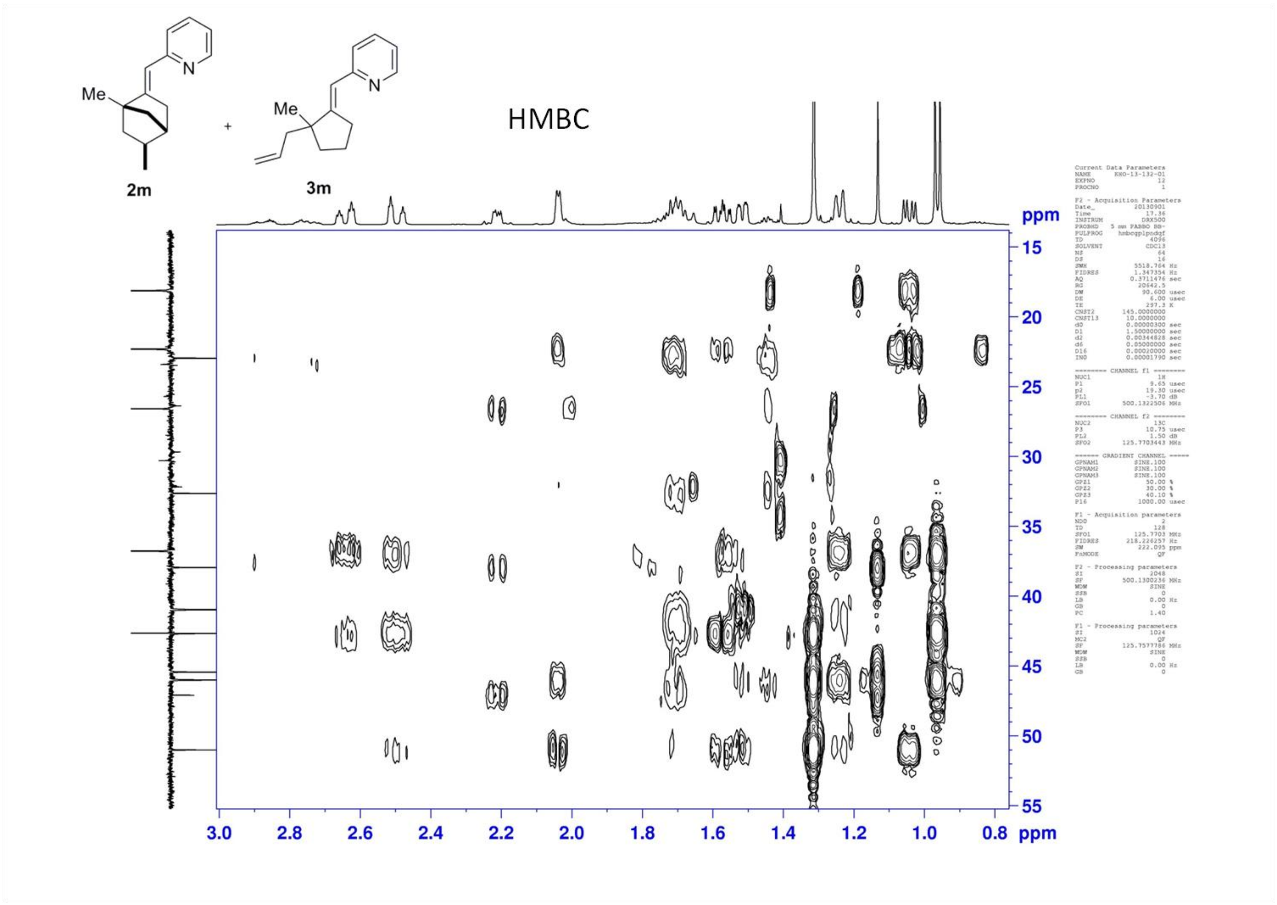
2m
3m
Me
N
HMBC
ppm

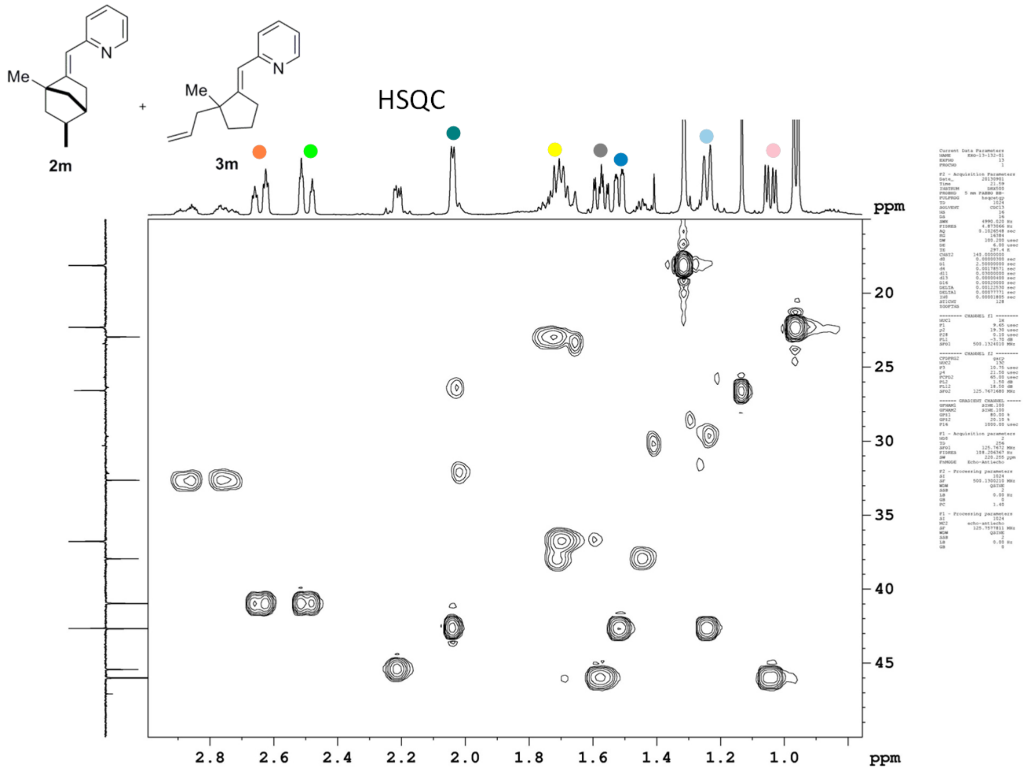
2m
3m
Me
N
HSQC
ppm

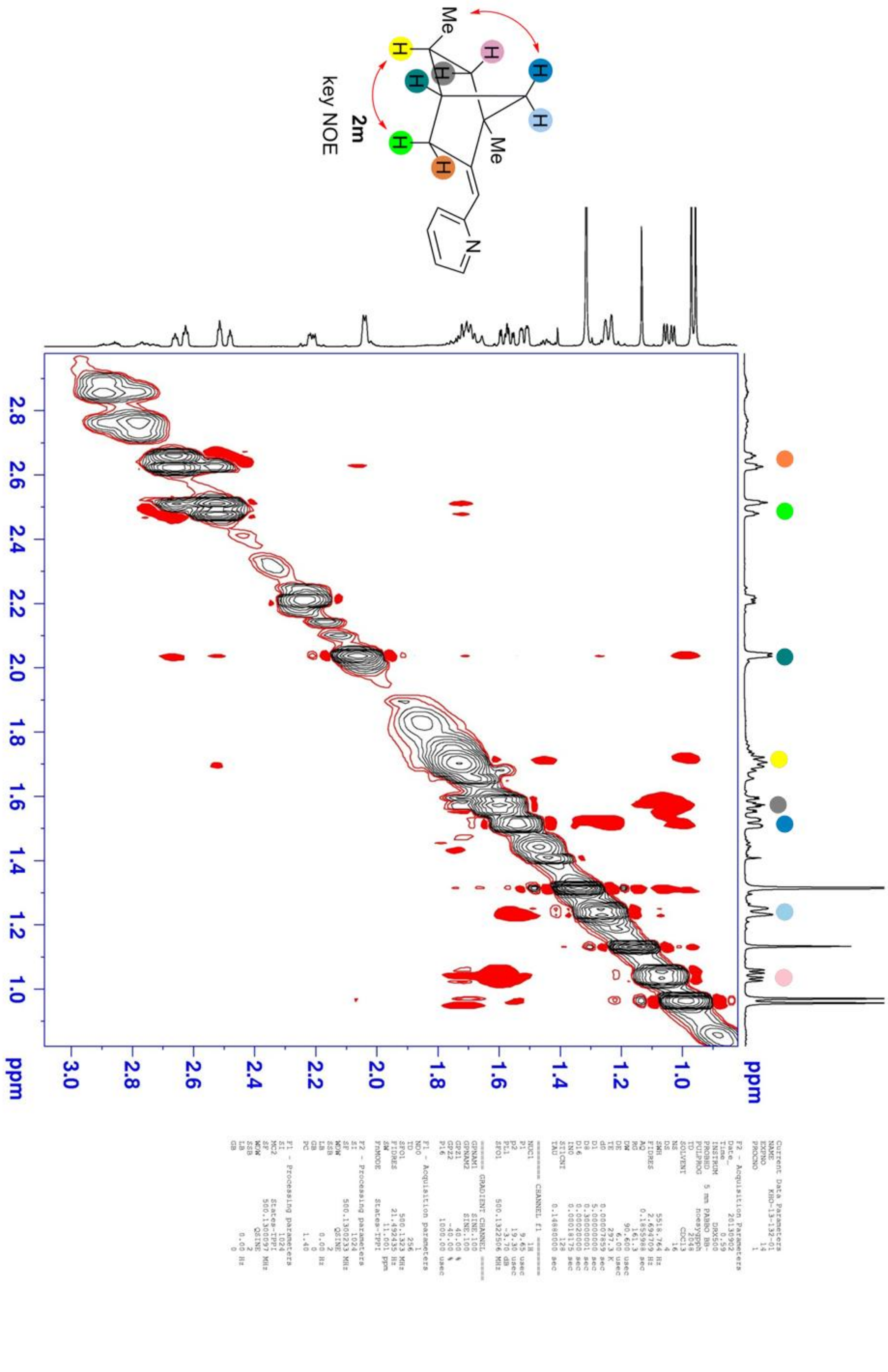
2m
key NOE
ppm
ppm

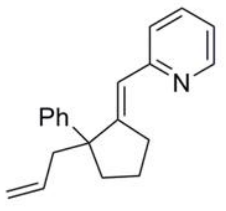

3a

```
Current Data Parameters
NAME      KHO-07-138-01
EXPNO                 1
PROCNO                1

F2 - Acquisition Parameters
Date_          20120430
Time              12.54
INSTRUM          DRX500
PROBHD   5 mm PABBO BB-
PULPROG            zg30
TD                65536
SOLVENT           CDCl3
NS                   16
DS                    2
SWH           10288.065 Hz
FIDRES         0.156983 Hz
AQ            3.1850996 sec
RG                406.4
DW               48.600 usec
DE                 6.00 usec
TE                295.2 K
D1           1.00000000 sec
TD0                   1

======== CHANNEL f1 ========
NUC1                 1H
P1                 9.80 usec
PL1               -3.60 dB
SFO1        500.1330885 MHz

F2 - Processing parameters
SI                32768
SF          500.1300225 MHz
WDW                  EM
SSB                   0
LB                 0.30 Hz
GB                    0
PC                 1.00
```

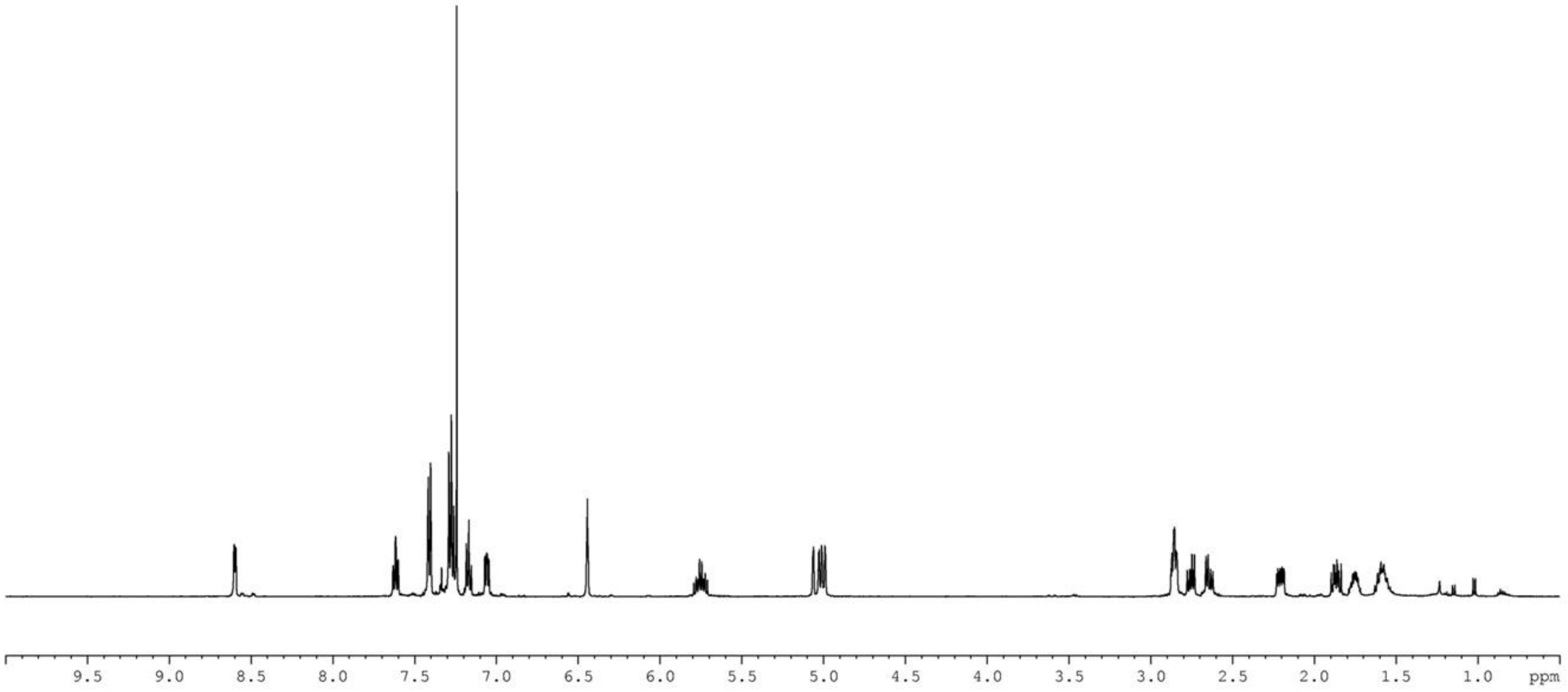

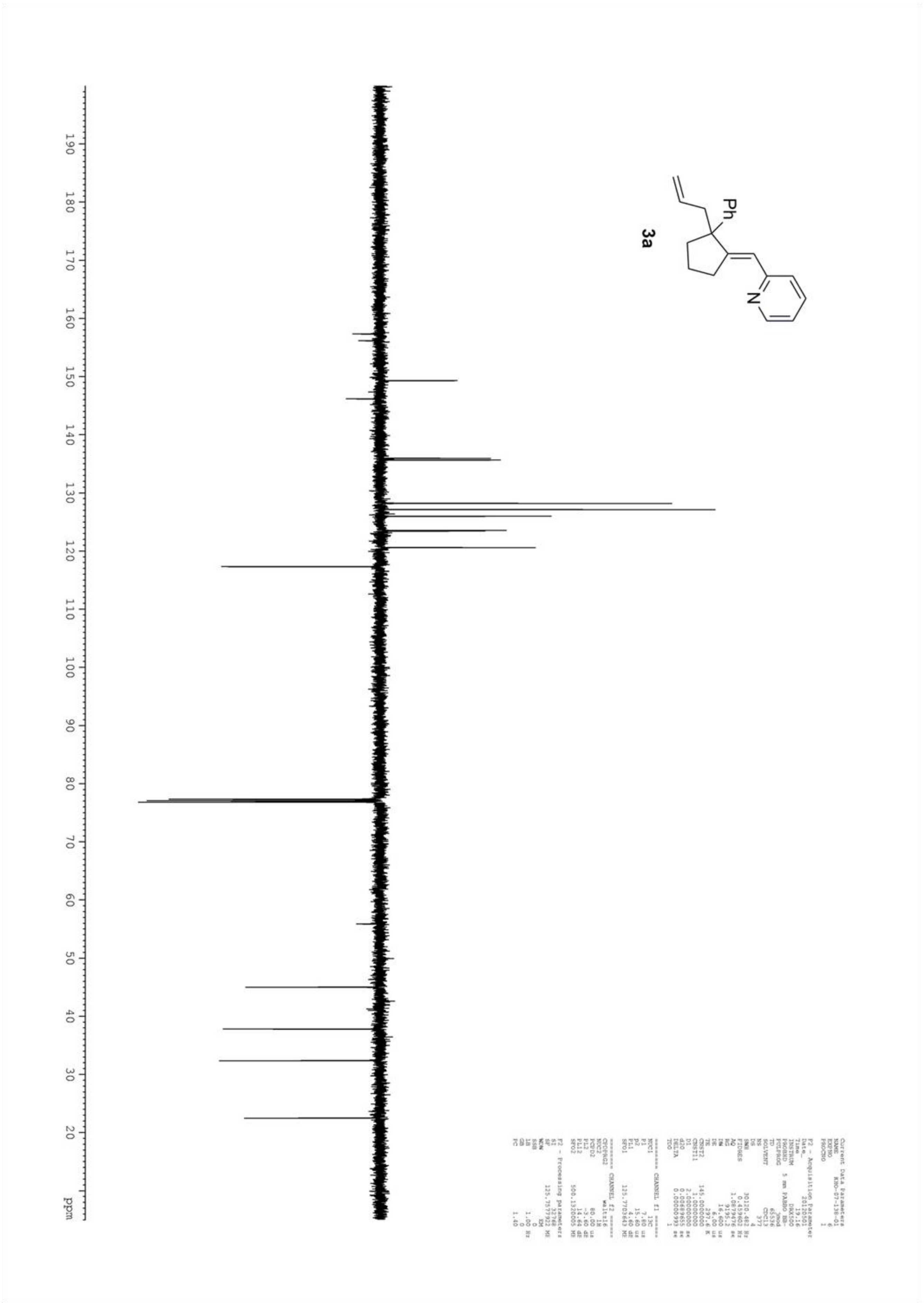
3a
Ph
N
190
180
170
160
150
140
130
120
110
100
90
80
70
60
50
40
30
20
ppm

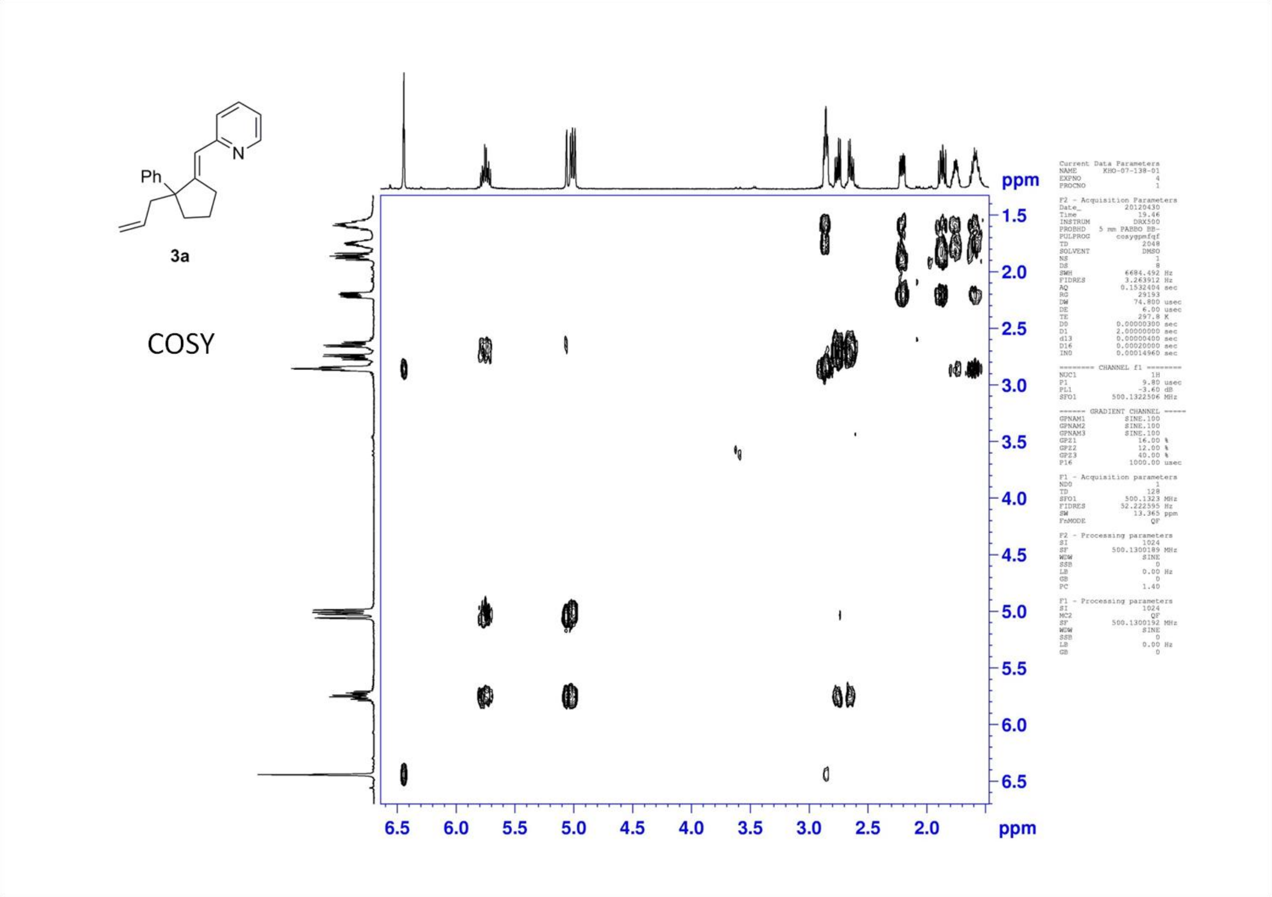
3a
COSY

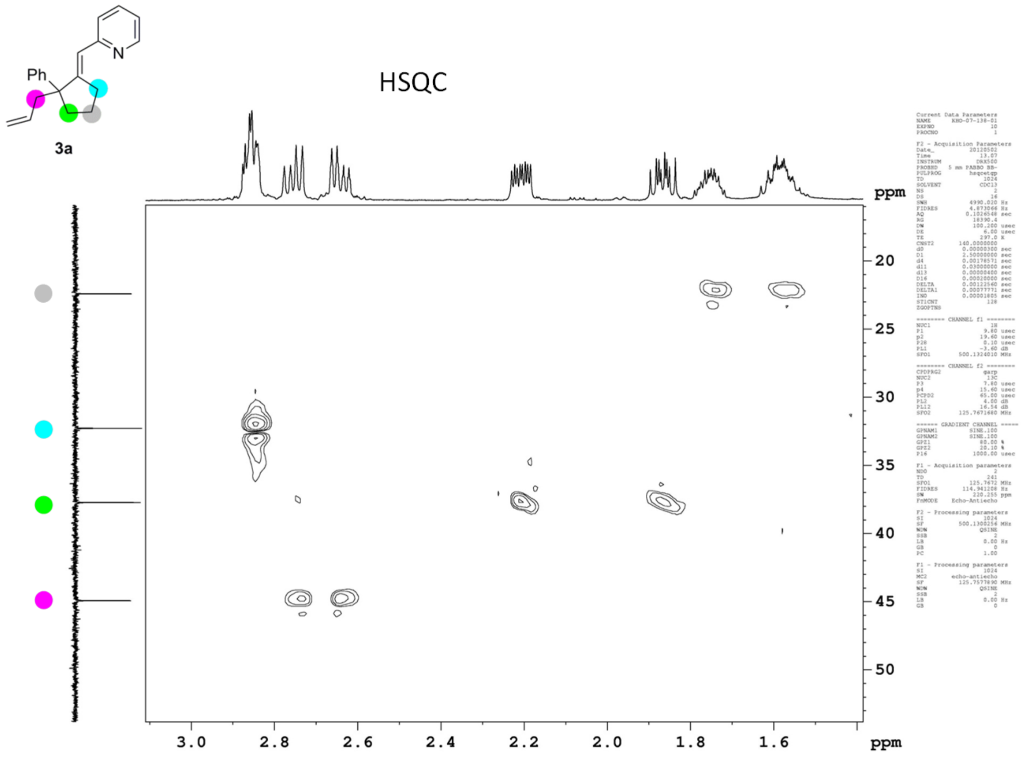
3a
HSQC

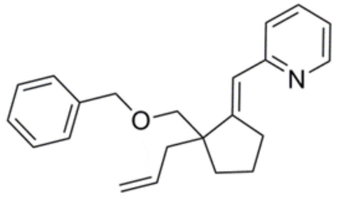

3l

```
Current Data Parameters
NAME        KHO-13-079-01
EXPNO                  10
PROCNO                  1

F2 - Acquisition Parameters
Date_            20130715
Time                20.01
INSTRUM            DRX500
PROBHD   5 mm PABBO BB-
PULPROG              zg30
TD                  65536
SOLVENT             CDCl3
NS                     16
DS                      2
SWH             10288.065 Hz
FIDRES           0.156983 Hz
AQ              3.1850996 sec
RG                    256
DW                 48.600 usec
DE                   6.00 usec
TE                  299.6 K
D1             1.00000000 sec
TD0                     1

======== CHANNEL f1 ========
NUC1                   1H
P1                   9.65 usec
PL1                 -3.70 dB
SFO1          500.1330885 MHz

F2 - Processing parameters
SI                  32768
SF            500.1300238 MHz
WDW                    EM
SSB                     0
LB                   0.30 Hz
GB                      0
PC                   1.00
```

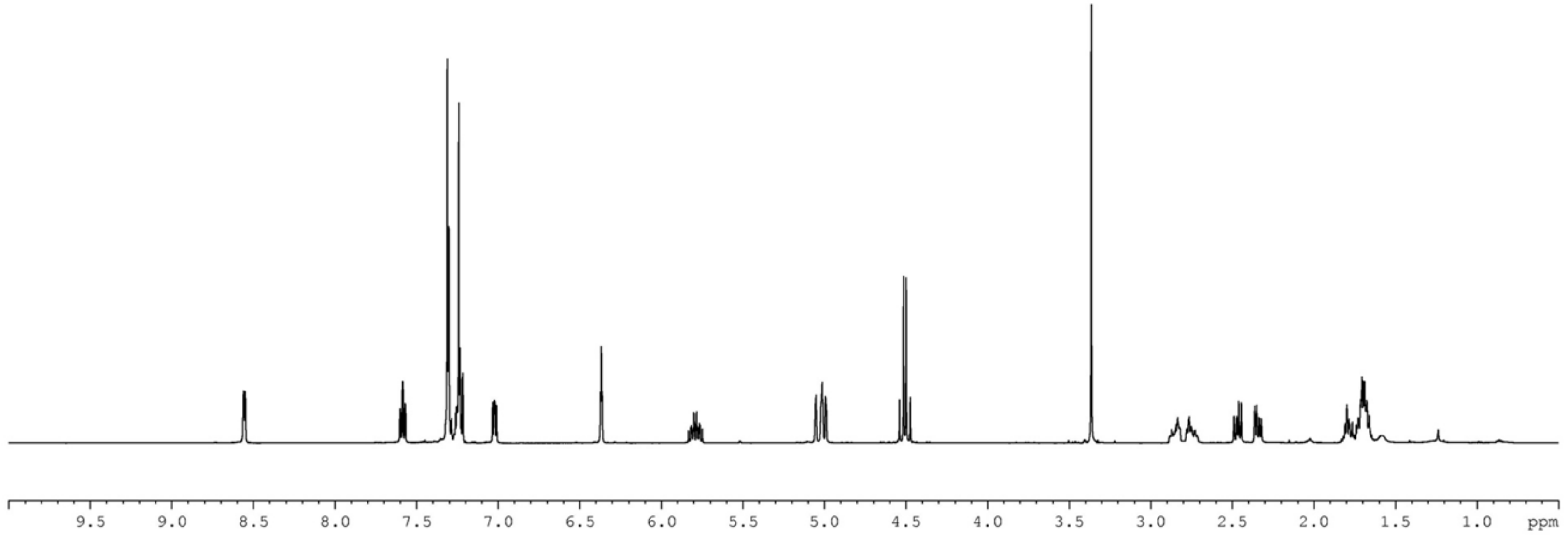

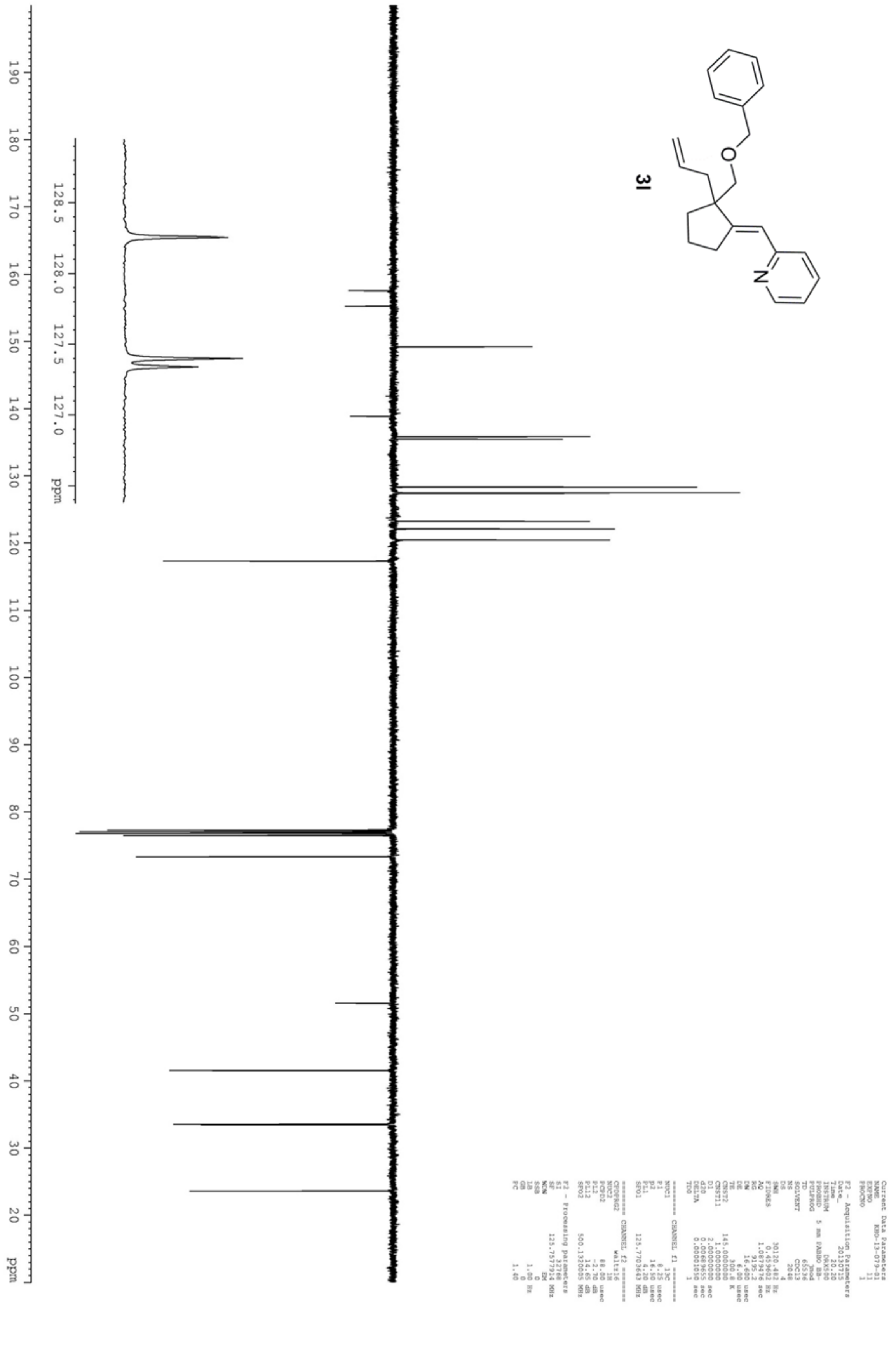
3l
ppm

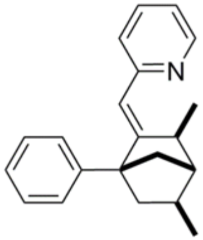

5

Current Data Parameters
NAME MPN-02-037-01-1
EXPNO 18
PROCNO 1

F2 - Acquisition Parameters
Date_ 20130801
Time 10.03
INSTRUM DRX500
PROBHD 5 mm PABBO BB-
PULPROG zg30
TD 65536
SOLVENT CDCl3
NS 16
DS 2
SWH 10288.065 Hz
FIDRES 0.156983 Hz
AQ 3.1850996 sec
RG 161.3
DW 48.600 use
DE 6.00 use
TE 299.4 K
D1 1.00000000 sec
TD0 1

======== CHANNEL f1 ========
NUC1 1H
P1 9.65 use
PL1 -3.70 dB
SFO1 500.1330885 MHz

F2 - Processing parameters
SI 32768
SF 500.1300239 MHz
WDW EM
SSB 0
LB 0.30 Hz
GB 0
PC 1.00

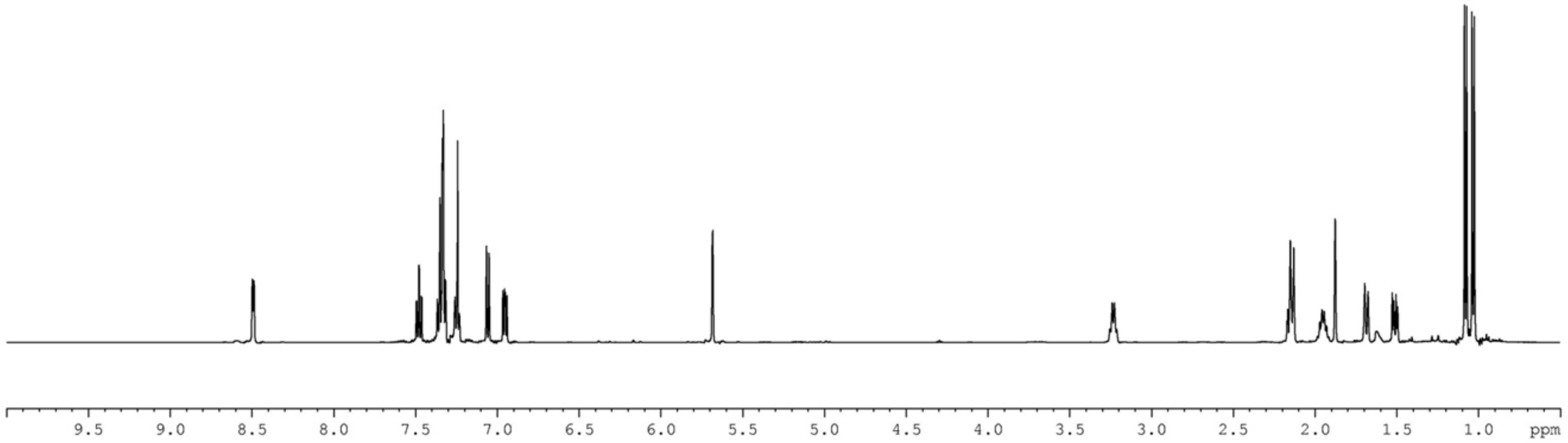

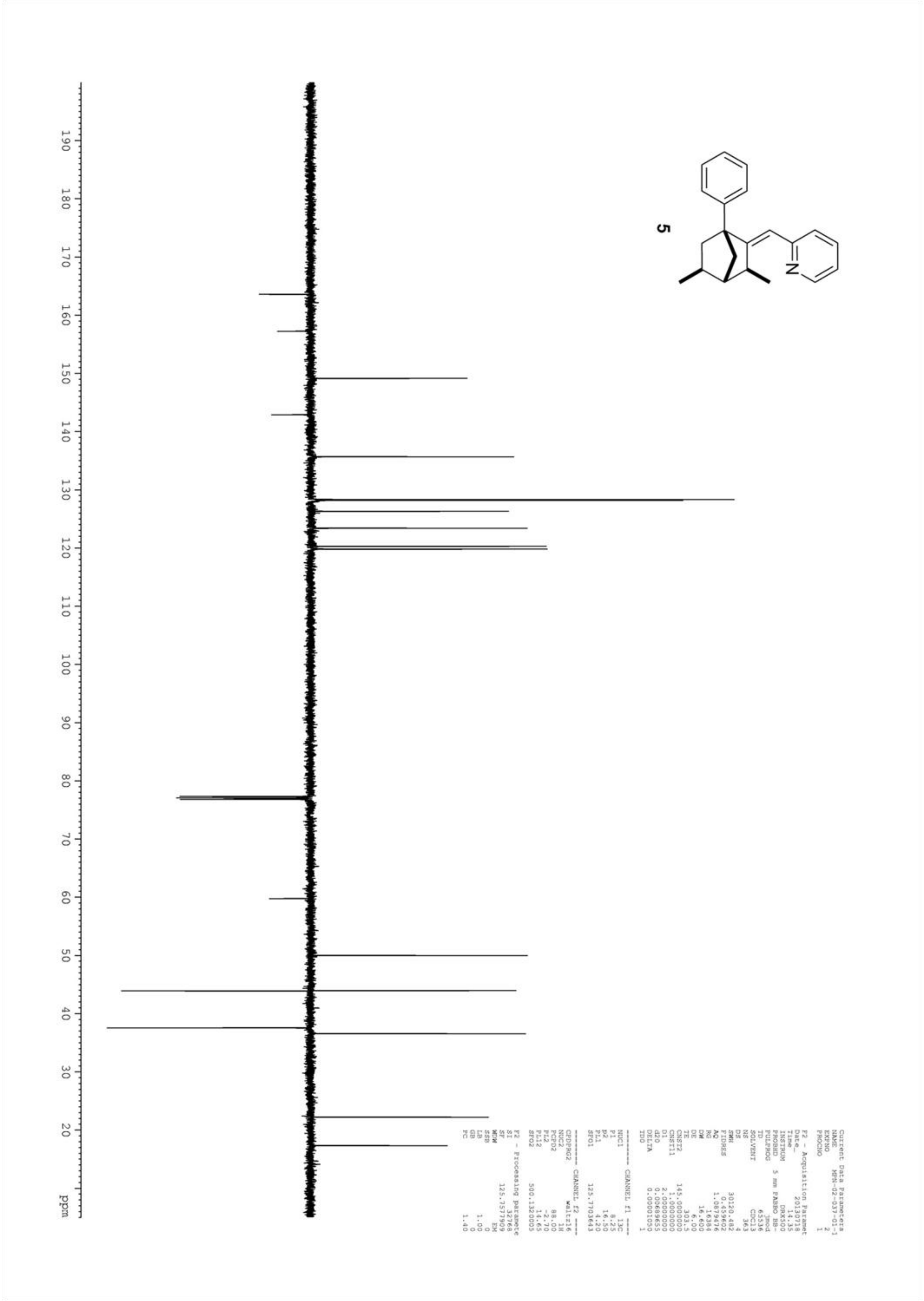

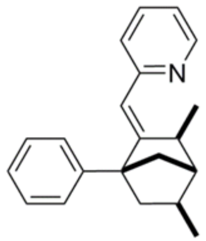

5

COSY

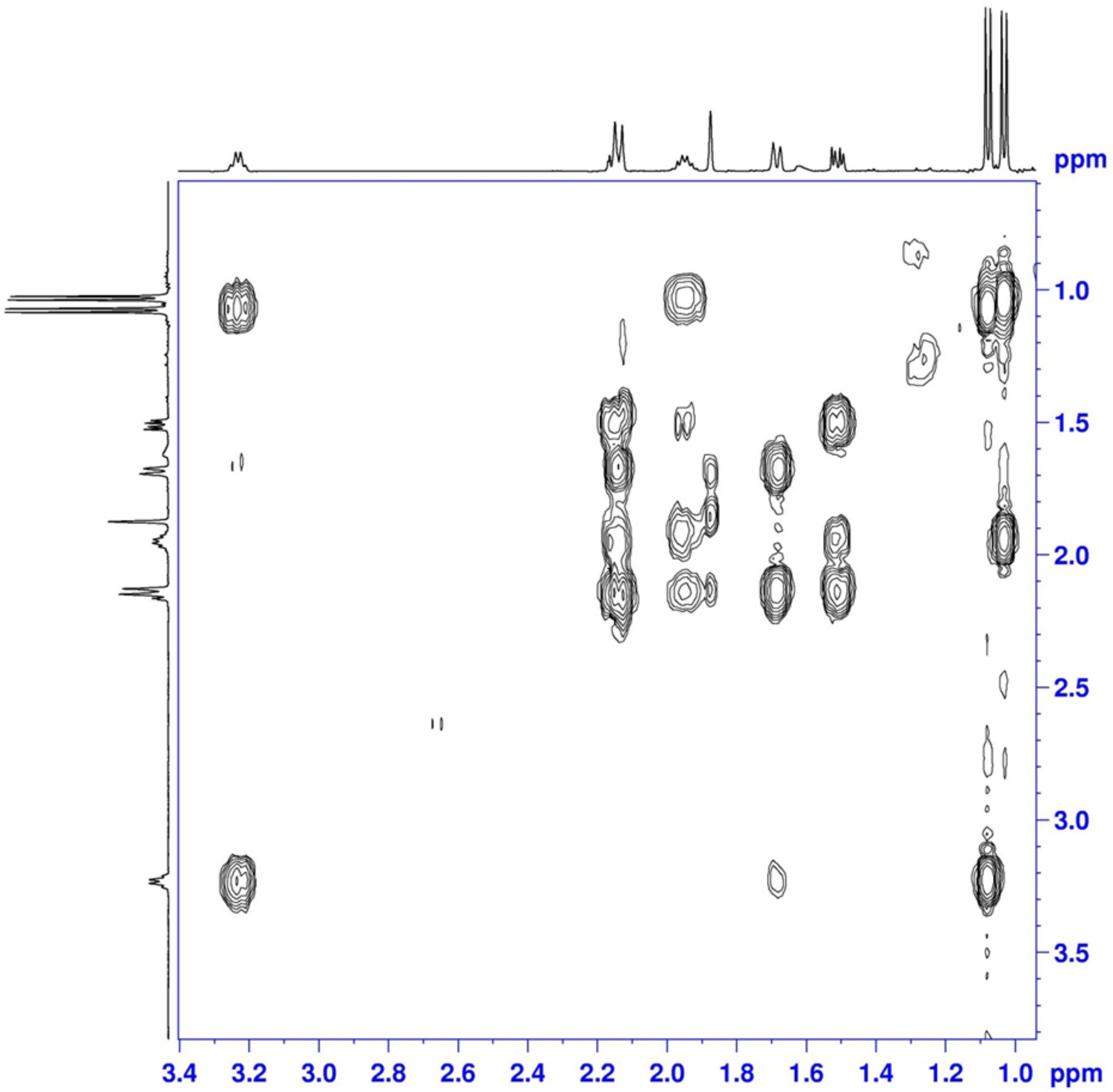


```
Current Data Parameters
NAME      MPN-02-037-01-1
EXPNO                   3
PROCNO                  1

F2 - Acquisition Parameters
Date_            20130718
Time                15.29
INSTRUM            DRX500
PROBHD    5 mm PABBO BB-
PULPROG        cosygpmfqf
TD                   2048
SOLVENT             CDCl3
NS                      4
DS                      8
SWH              6684.492 Hz
FIDRES           3.263912 Hz
AQ              0.1532404 sec
RG                11585.2
DW                 74.800 usec
DE                   6.00 usec
TE                  302.6 K
D0             0.00000300 sec
D1             2.00000000 sec
d13            0.00000400 sec
D16            0.00020000 sec
IN0            0.00014960 sec

======== CHANNEL f1 ========
NUC1                   1H
P1                   9.65 usec
PL1                 -3.70 dB
SFO1          500.1330069 MHz

====== GRADIENT CHANNEL =====
GPNAM1           SINE.100
GPNAM2           SINE.100
GPNAM3           SINE.100
GPZ1                16.00 %
GPZ2                12.00 %
GPZ3                40.00 %
P16               1000.00 usec

F1 - Acquisition parameters
ND0                     1
TD                    128
SFO1              500.133 MHz
FIDRES          52.222595 Hz
SW                 13.365 ppm
FnMODE                 QF

F2 - Processing parameters
SI                   1024
SF            500.1300223 MHz
WDW                  SINE
SSB                     0
LB                   0.00 Hz
GB                      0
PC                   1.00

F1 - Processing parameters
SI                   1024
MC2                    QF
SF            500.1300259 MHz
WDW                  SINE
SSB                     0
LB                   0.00 Hz
GB                      0
```

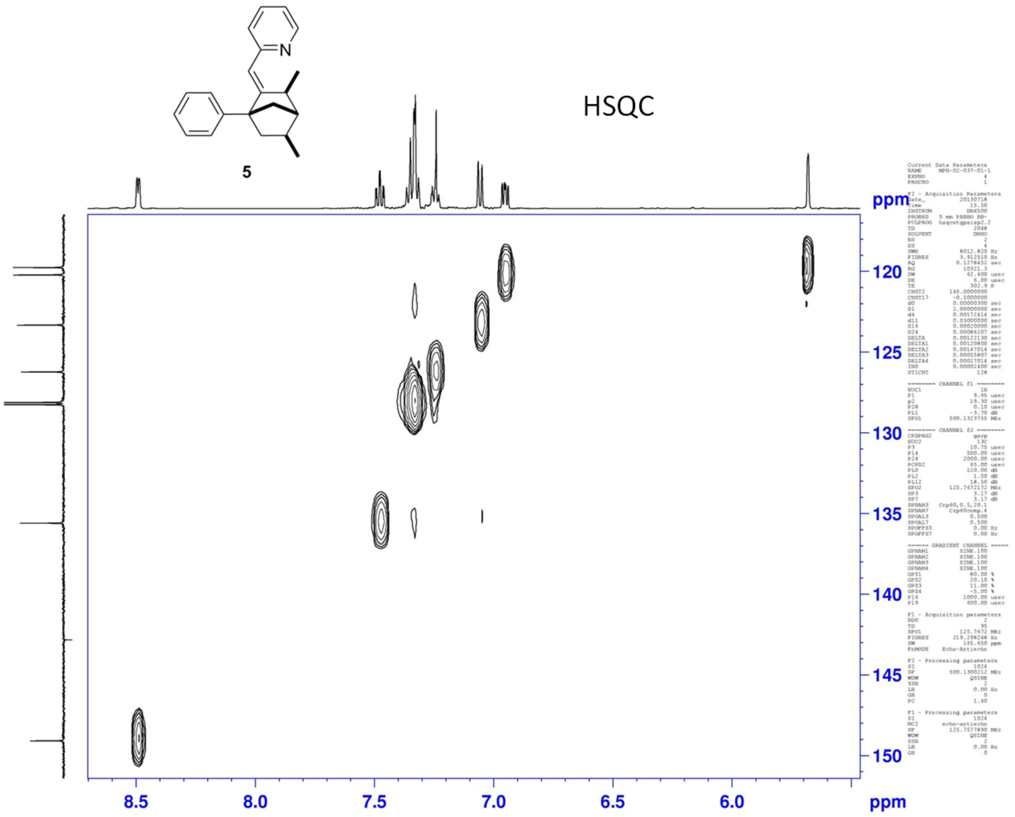
HSQC
5
ppm
120
125
130
135
140
145
150
8.5
8.0
7.5
7.0
6.5
6.0
ppm

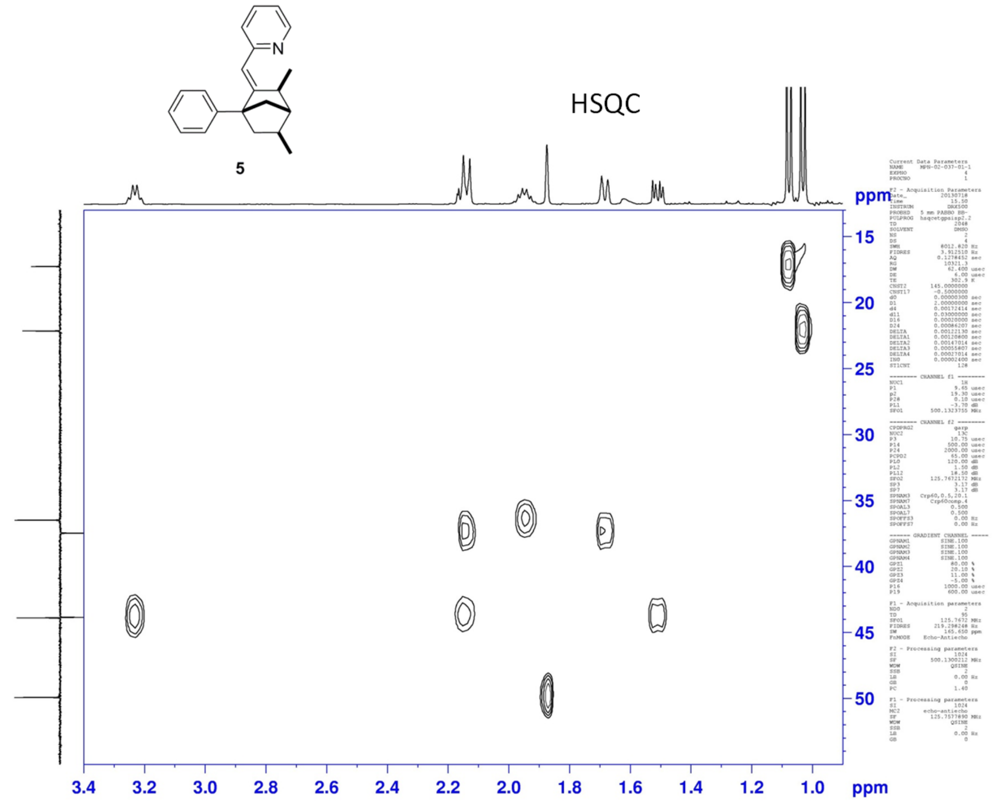
HSQC
5
ppm
15
20
25
30
35
40
45
50
3.4
3.2
3.0
2.8
2.6
2.4
2.2
2.0
1.8
1.6
1.4
1.2
1.0
ppm

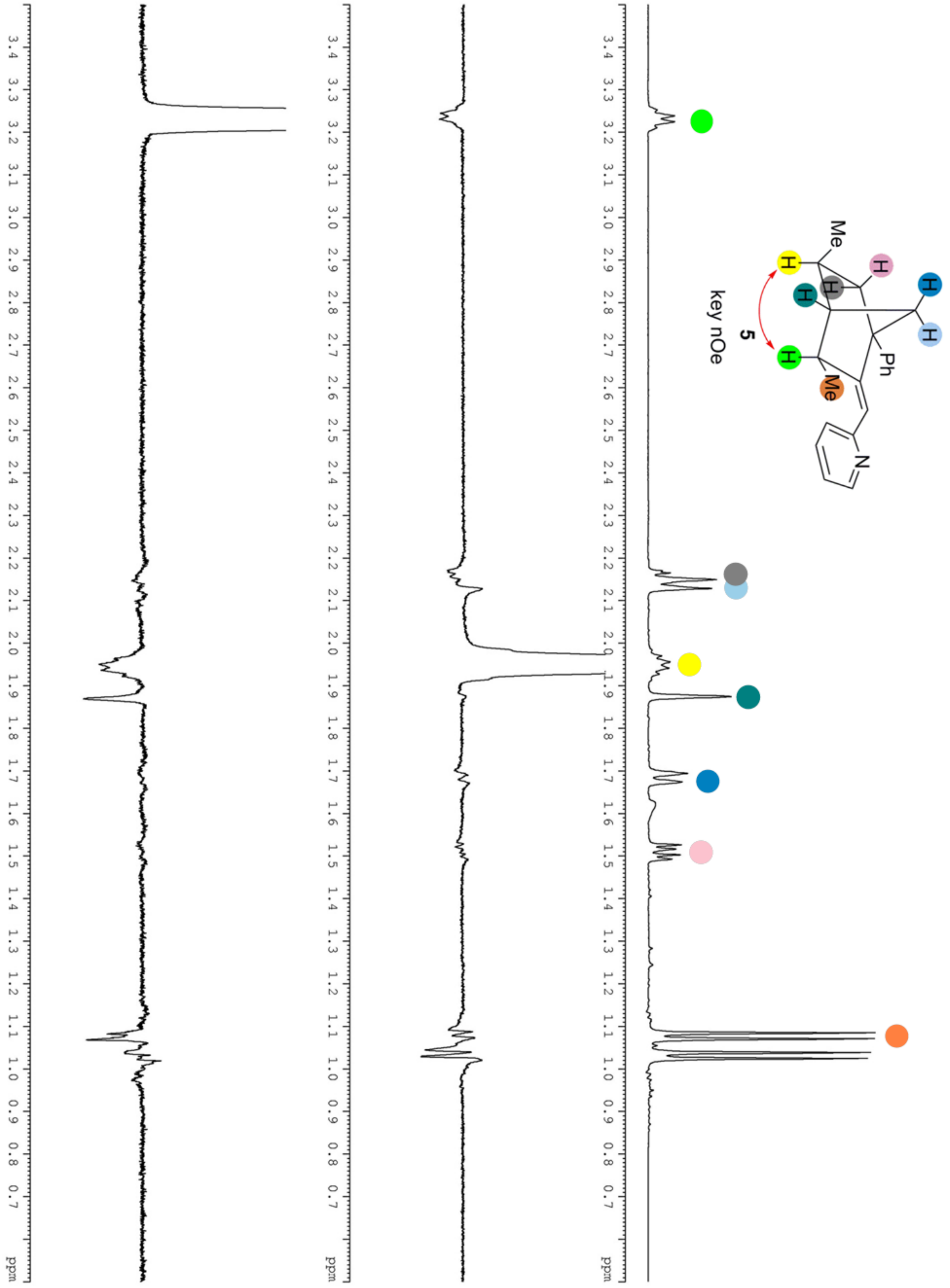
Me
Ph
Me
N
5
key nOe
ppm

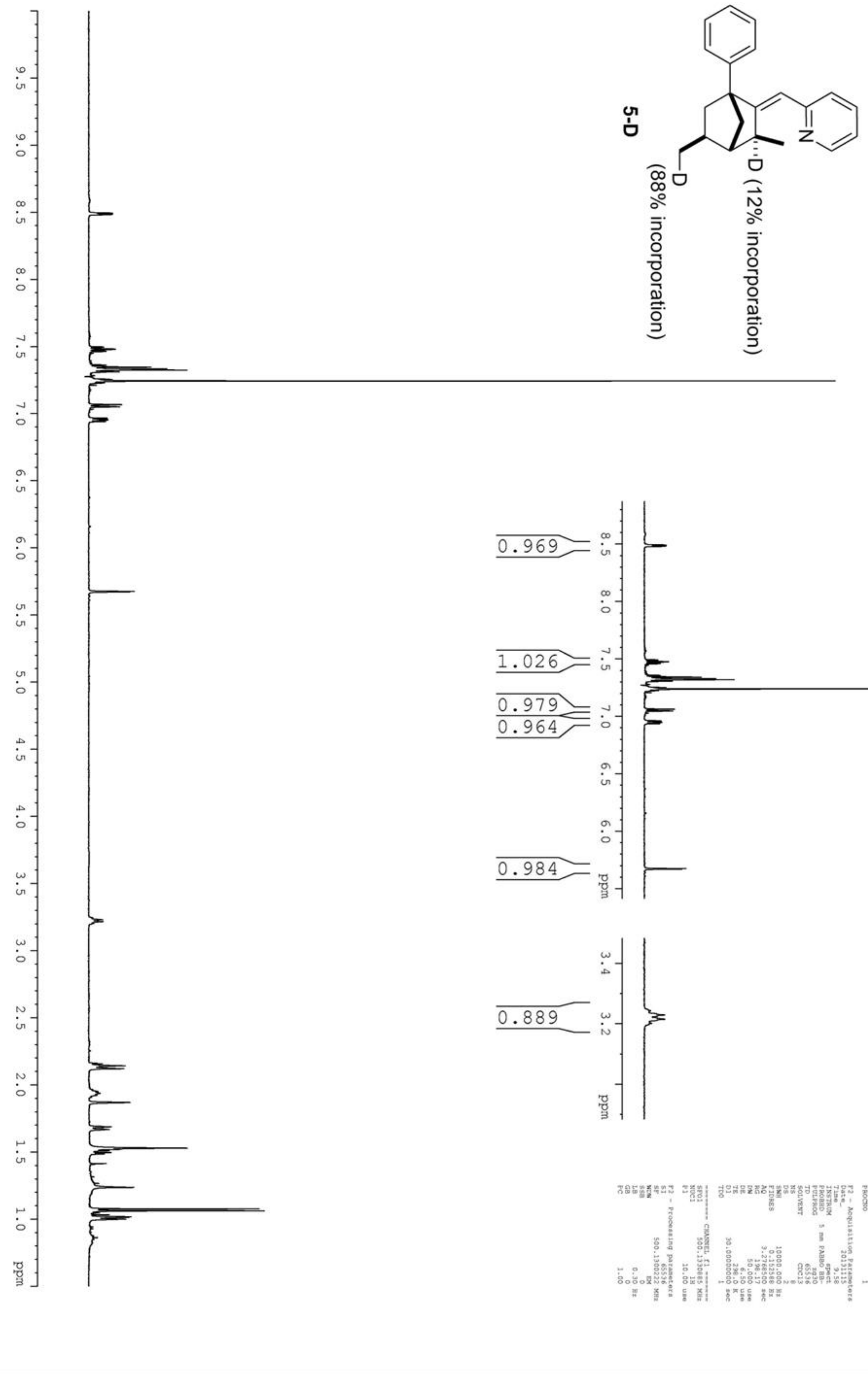

5-D
D (12% incorporation)
D
(88% incorporation)
0.969
1.026
0.979
0.964
0.984
0.889
ppm

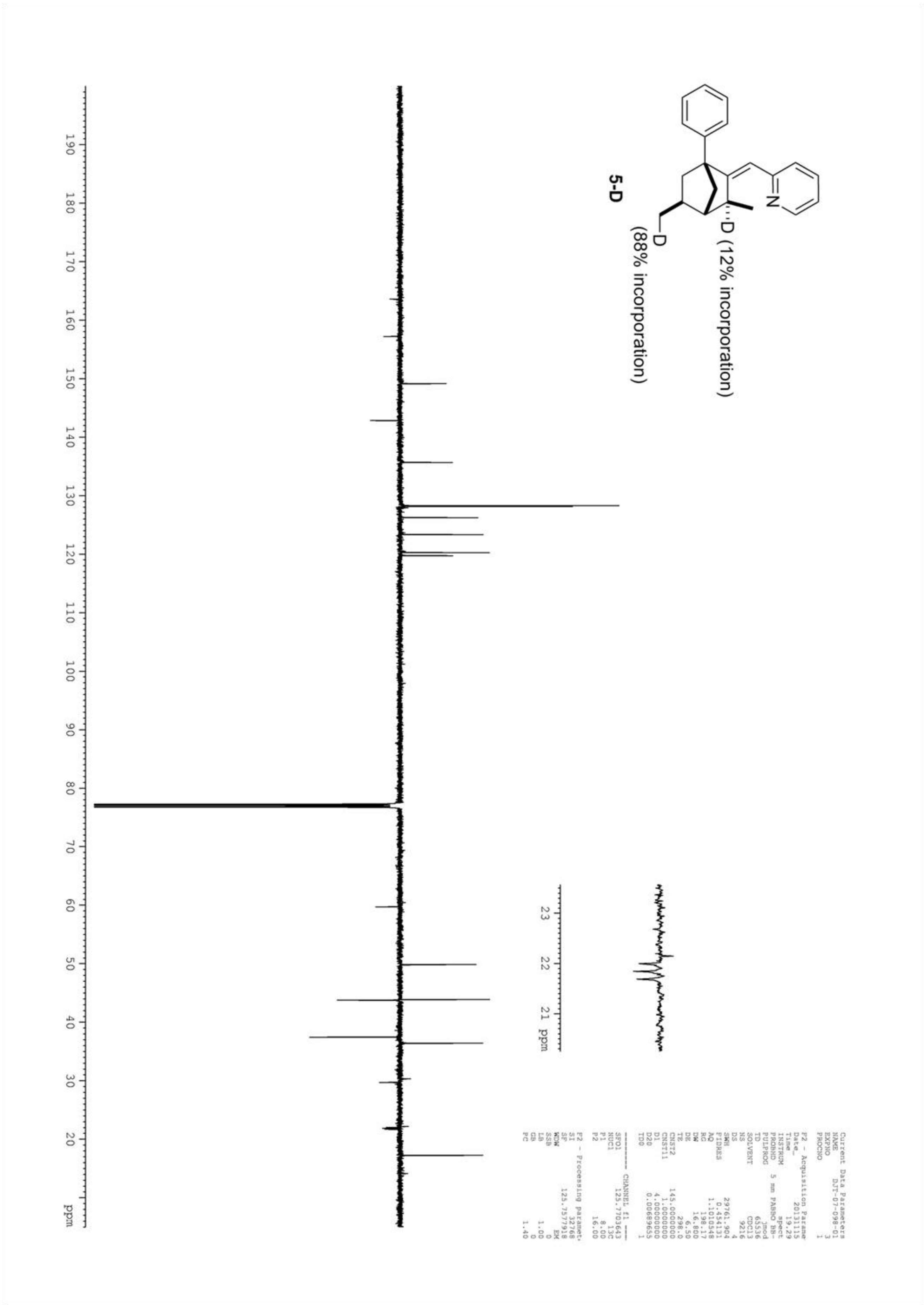
D (12% incorporation)
D
(88% incorporation)
5-D
ppm

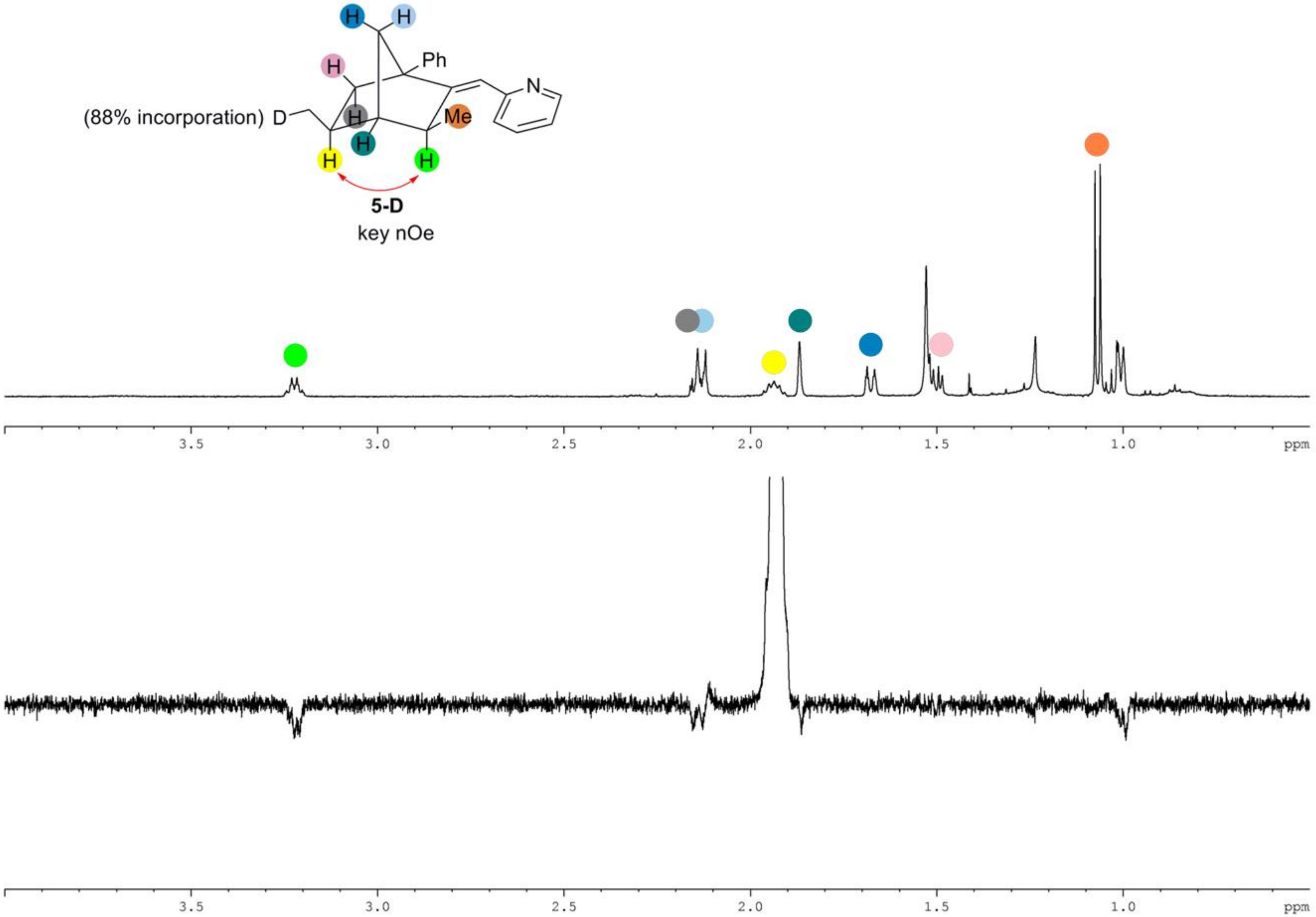
(88% incorporation) D
Ph
Me
N
H
5-D
key nOe
3.5
3.0
2.5
2.0
1.5
1.0
ppm

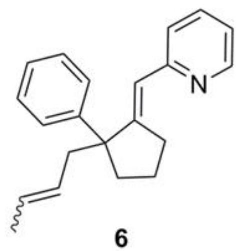

6
(E/Z = 55:45)

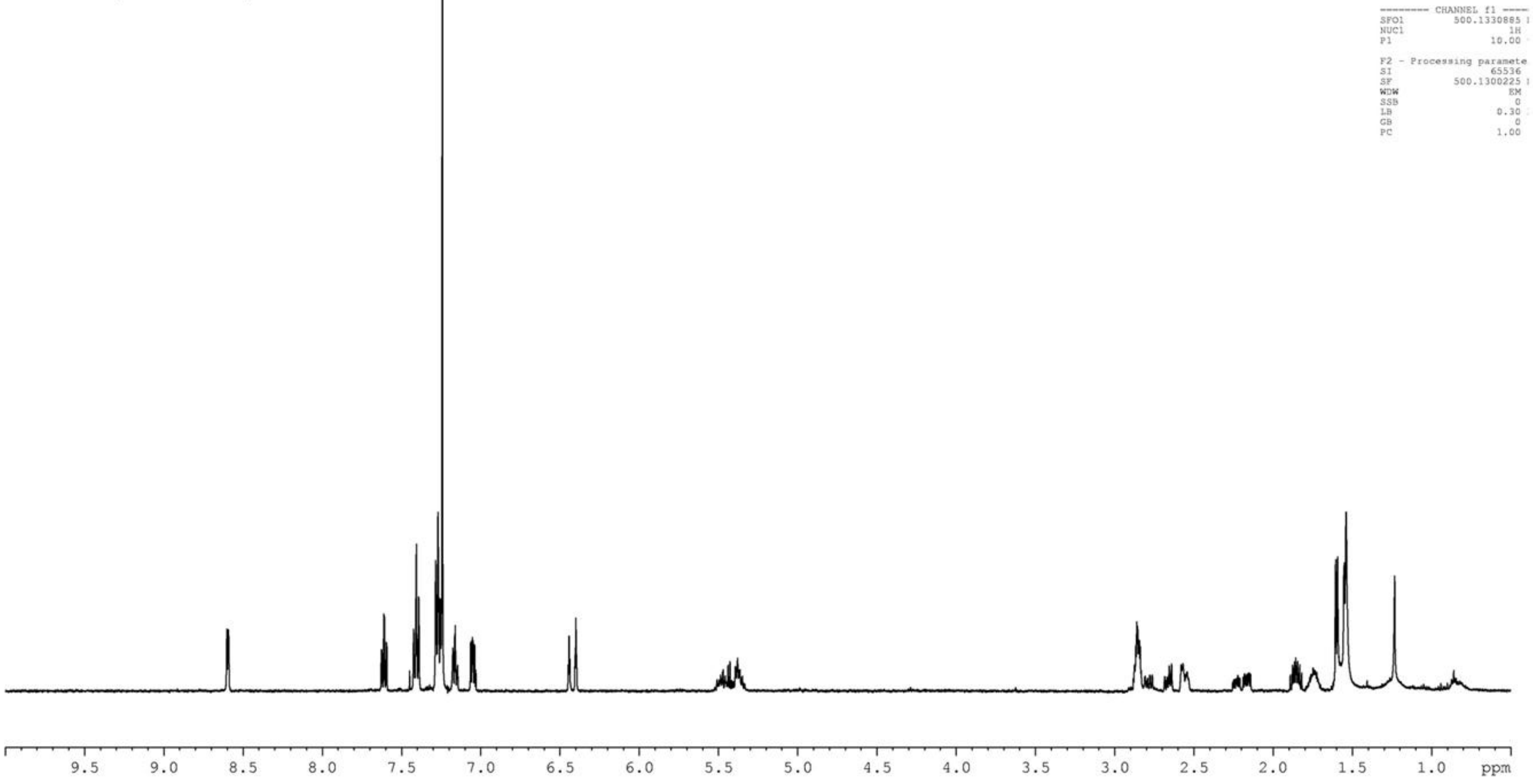
Current Data Parameters
NAME MPN-02-037-3-B-
EXPNO 7
PROCNO 1
F2 - Acquisition Paramet
Date_ 20131016
Time 18.42
INSTRUM spect
PROBHD 5 mm PABBO BB-
PULPROG zg30
TD 65536
SOLVENT CDCl3
NS 16
DS 2
SWH 10000.000
FIDRES 0.152588
AQ 3.2768500
RG 198.17
DW 50.000
DE 6.50
TE 296.0
D1 1.00000000
TD0 1
======== CHANNEL f1 ========
SFO1 500.1330885
NUC1 1H
P1 10.00
F2 - Processing paramete
SI 65536
SF 500.1300225
WDW EM
SSB 0
LB 0.30
GB 0
PC 1.00
9.5 9.0 8.5 8.0 7.5 7.0 6.5 6.0 5.5 5.0 4.5 4.0 3.5 3.0 2.5 2.0 1.5 1.0 ppm

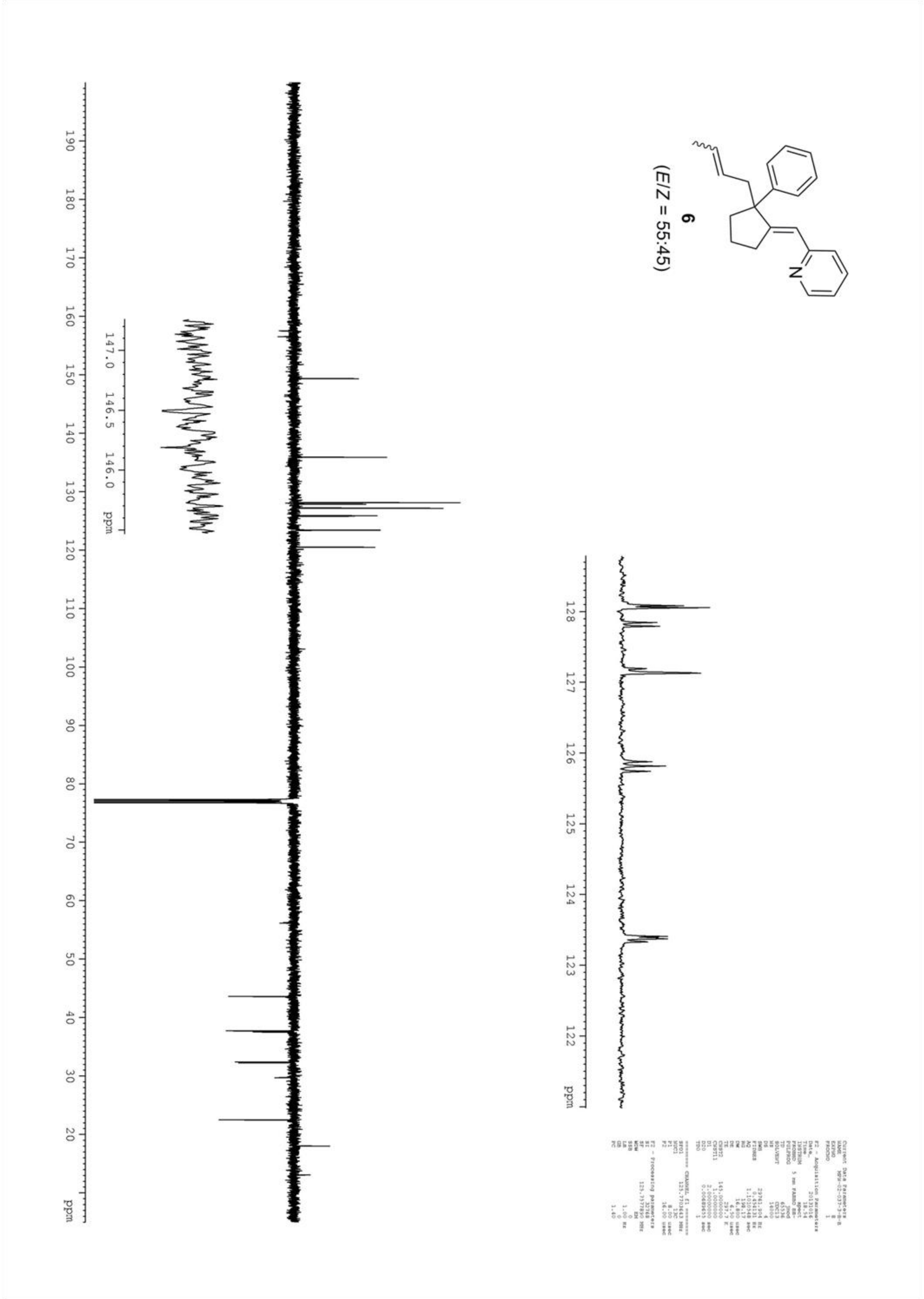
N
6
(E/Z = 55:45)

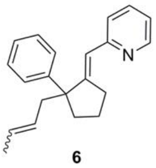

6
(*E*/*Z* = 55:45)

COSY

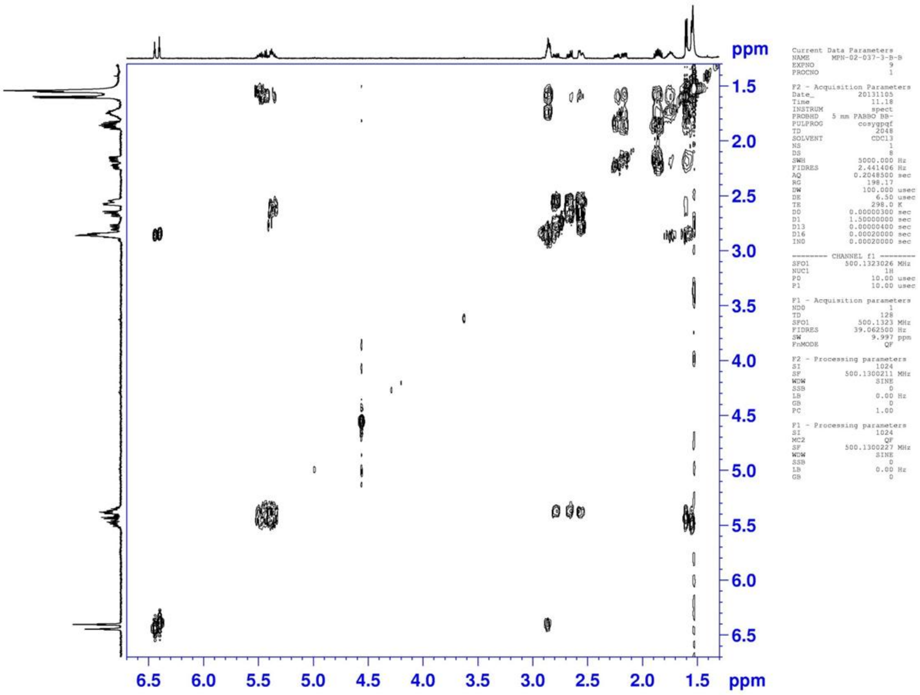

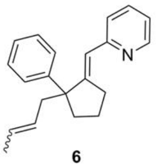

6
(*E*/*Z* = 55:45)

COSY

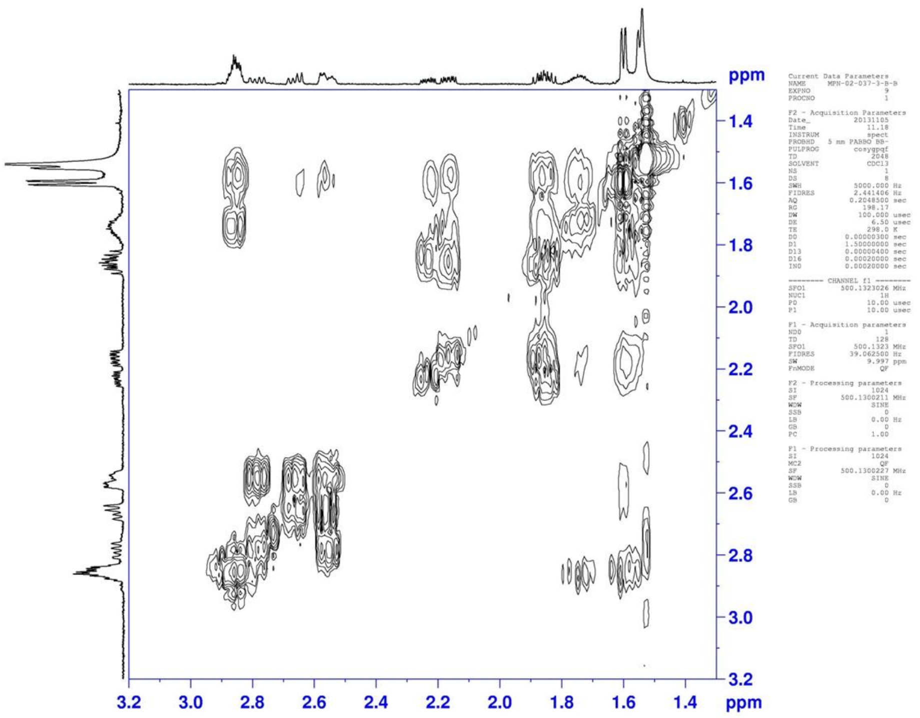

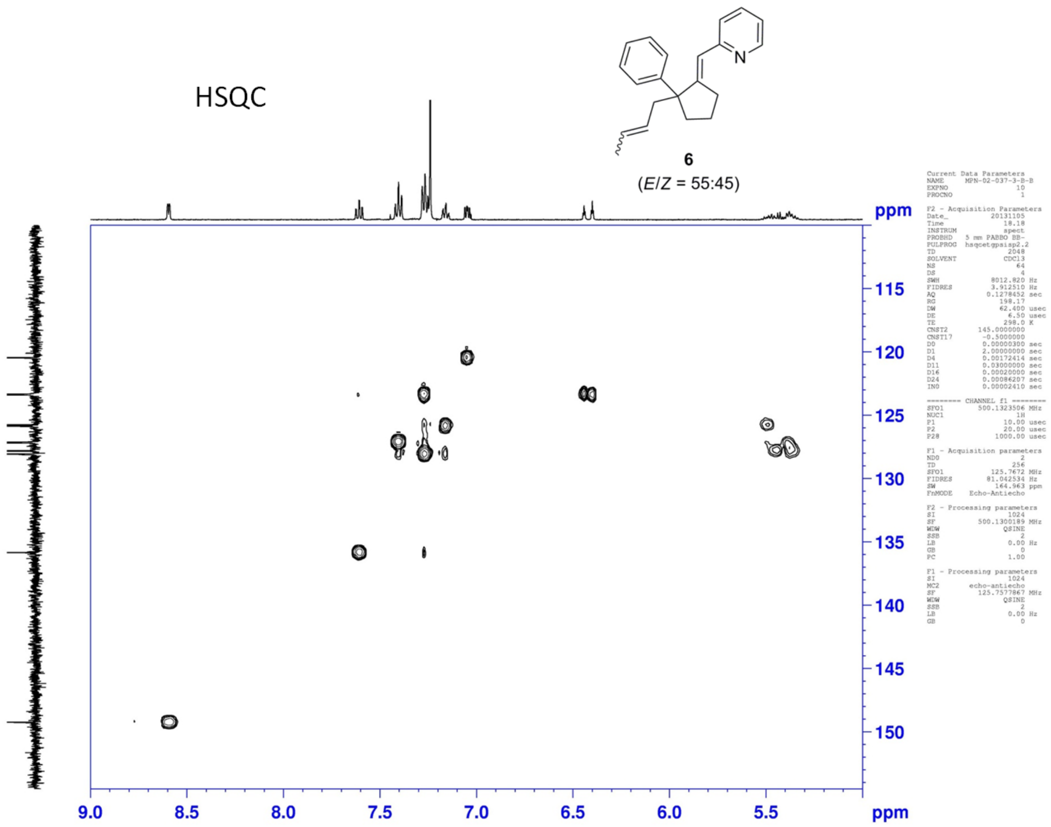
HSQC
N
6
(E/Z = 55:45)
ppm
115
120
125
130
135
140
145
150
9.0
8.5
8.0
7.5
7.0
6.5
6.0
5.5
ppm

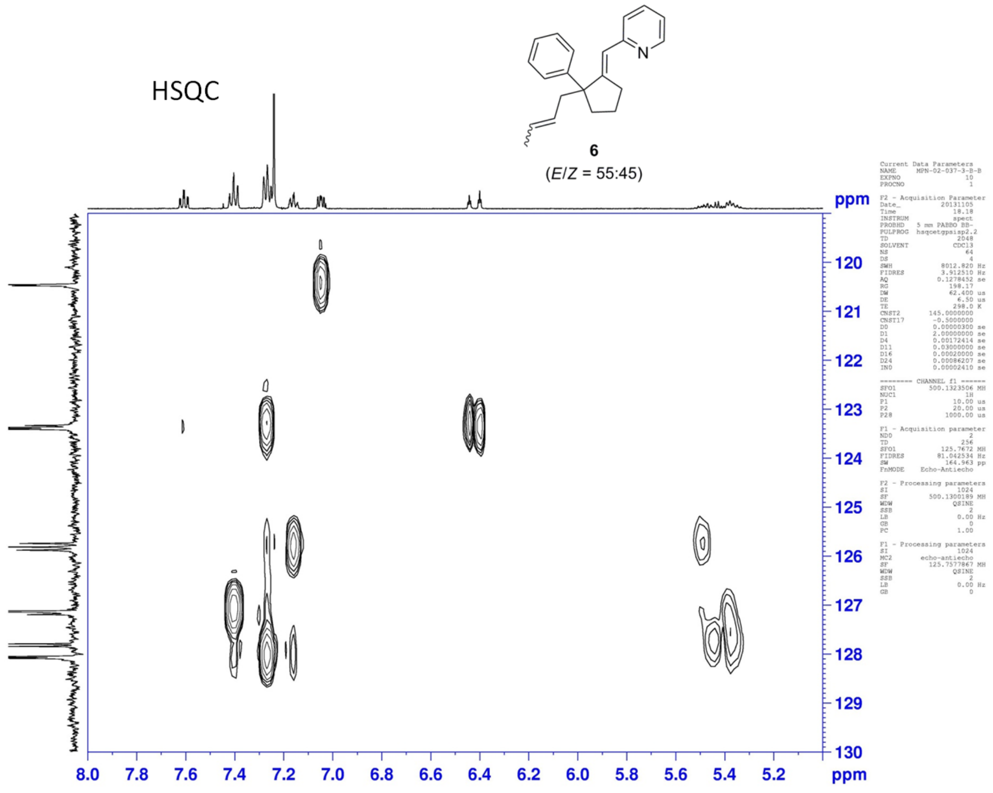
HSQC
N
6
(E/Z = 55:45)
ppm
120
121
122
123
124
125
126
127
128
129
130
8.0
7.8
7.6
7.4
7.2
7.0
6.8
6.6
6.4
6.2
6.0
5.8
5.6
5.4
5.2
ppm

HSQC

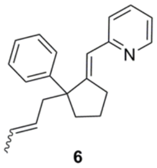

6

(E/Z = 55:45)

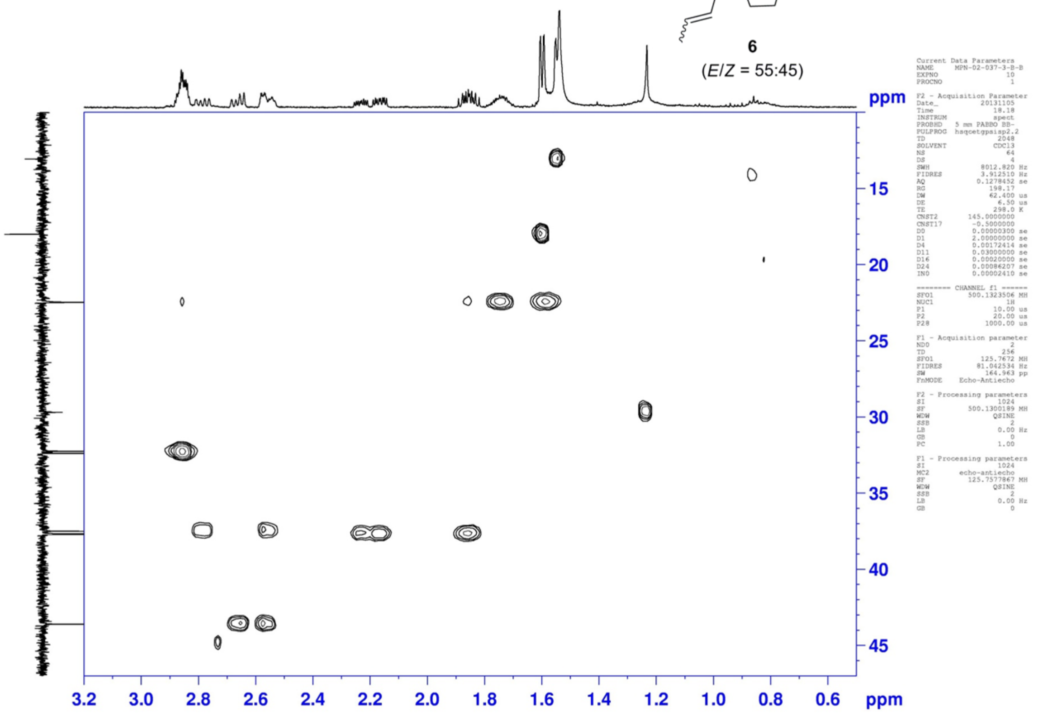

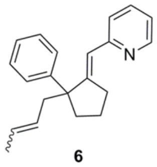

6

(E/Z = 55:45)

HSQC

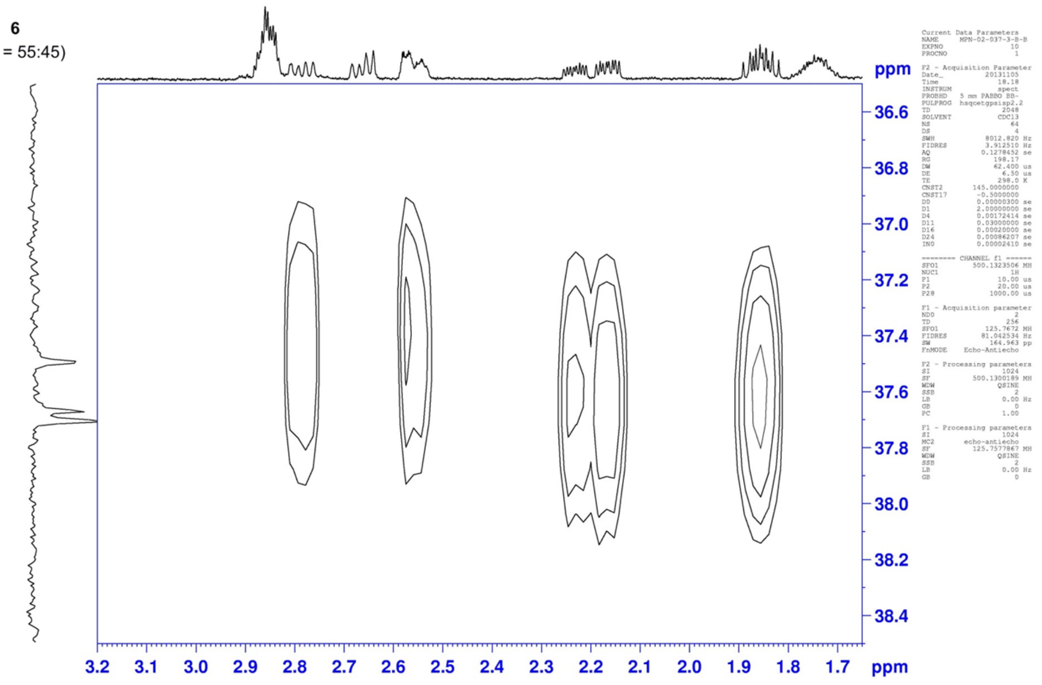

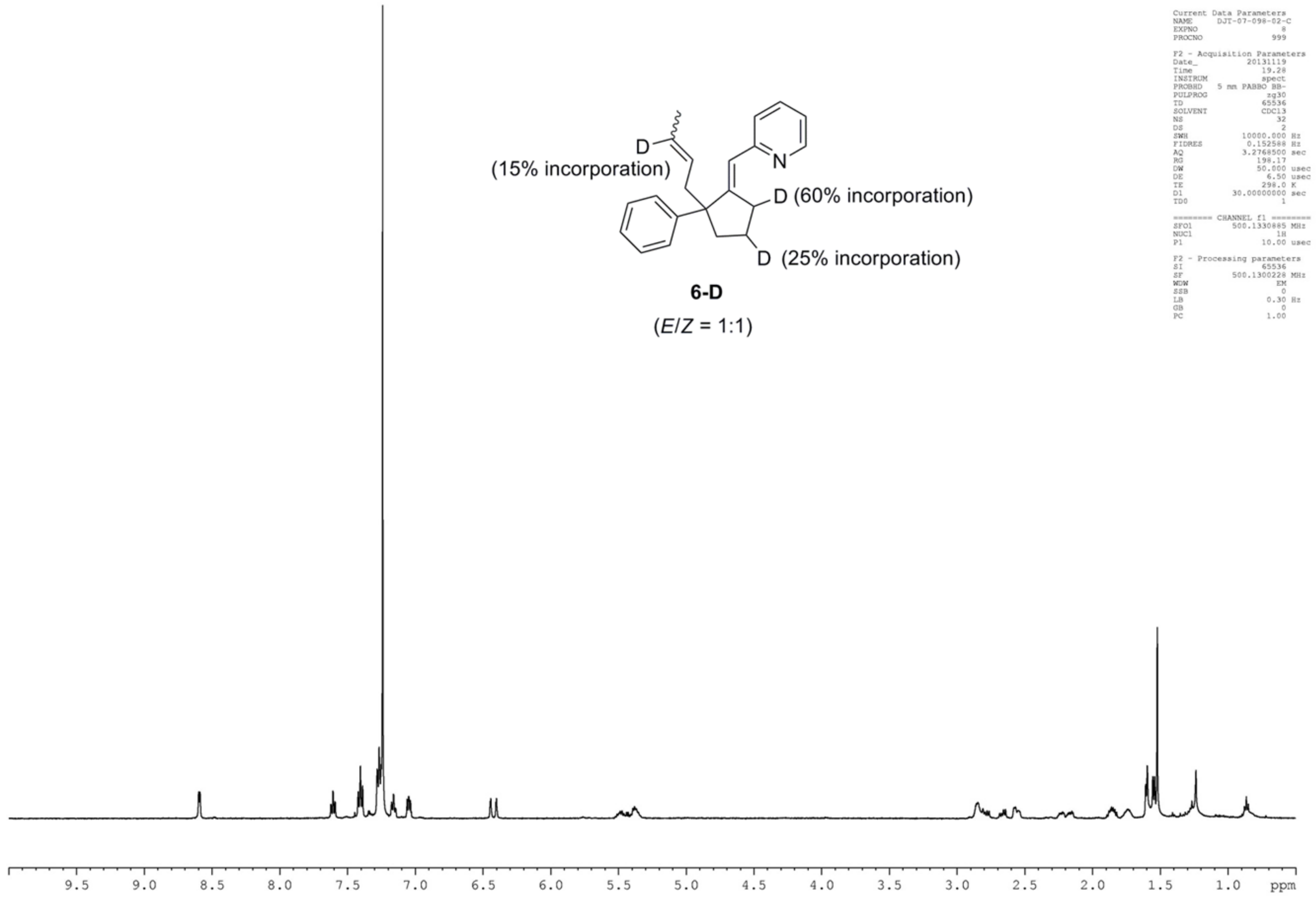

Current Data Parameters
NAME DJT-07-098-02-C
EXPNO 8
PROCNO 999
F2 - Acquisition Parameters
Date_ 20131119
Time 19.28
INSTRUM spect
PROBHD 5 mm PABBO BB-
PULPROG zg30
TD 65536
SOLVENT CDCl3
NS 32
DS 2
SWH 10000.000 Hz
FIDRES 0.152588 Hz
AQ 3.2768500 sec
RG 198.17
DW 50.000 usec
DE 6.50 usec
TE 298.0 K
D1 30.00000000 sec
TD0 1
======== CHANNEL f1 ========
SFO1 500.1330885 MHz
NUC1 1H
P1 10.00 usec
F2 - Processing parameters
SI 65536
SF 500.1300228 MHz
WDW EM
SSB 0
LB 0.30 Hz
GB 0
PC 1.00
D (15% incorporation)
N
D (60% incorporation)
D (25% incorporation)
6-D
(E/Z = 1:1)
9.5
9.0
8.5
8.0
7.5
7.0
6.5
6.0
5.5
5.0
4.5
4.0
3.5
3.0
2.5
2.0
1.5
1.0
ppm

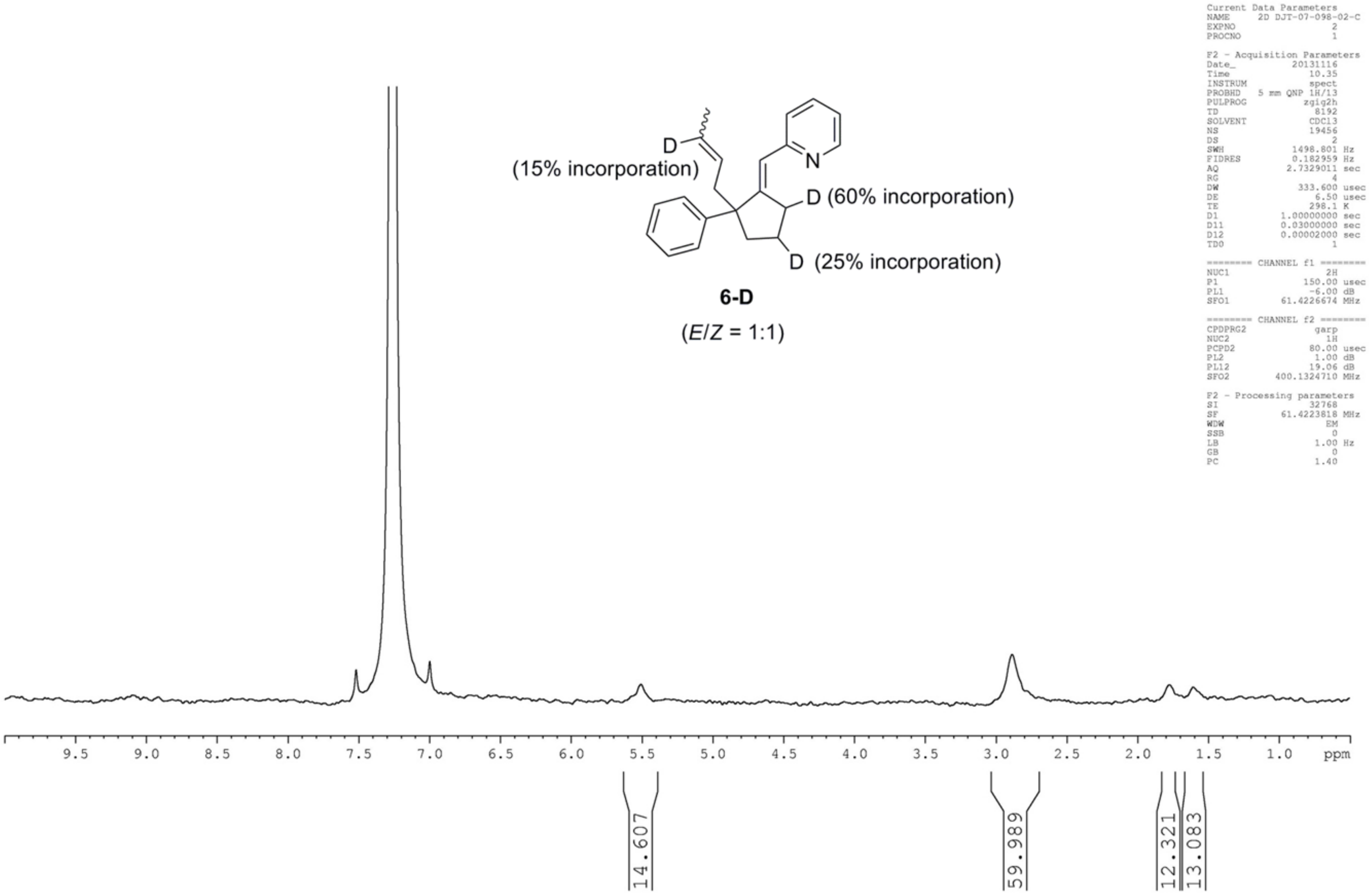

D (15% incorporation)
D (60% incorporation)
D (25% incorporation)
N
6-D
(E/Z = 1:1)
Current Data Parameters
NAME 2D DJT-07-098-02-C
EXPNO 2
PROCNO 1
F2 - Acquisition Parameters
Date_ 20131116
Time 10.35
INSTRUM spect
PROBHD 5 mm QNP 1H/13
PULPROG zgig2h
TD 8192
SOLVENT CDCl3
NS 19456
DS 2
SWH 1498.801 Hz
FIDRES 0.182959 Hz
AQ 2.7329011 sec
RG 4
DW 333.600 usec
DE 6.50 usec
TE 298.1 K
D1 1.00000000 sec
D11 0.03000000 sec
D12 0.00002000 sec
TD0 1
======== CHANNEL f1 ========
NUC1 2H
P1 150.00 usec
PL1 -6.00 dB
SFO1 61.4226674 MHz
======== CHANNEL f2 ========
CPDPRG2 garp
NUC2 1H
PCPD2 80.00 usec
PL2 1.00 dB
PL12 19.06 dB
SFO2 400.1324710 MHz
F2 - Processing parameters
SI 32768
SF 61.4223818 MHz
WDW EM
SSB 0
LB 1.00 Hz
GB 0
PC 1.40
9.5 9.0 8.5 8.0 7.5 7.0 6.5 6.0 5.5 5.0 4.5 4.0 3.5 3.0 2.5 2.0 1.5 1.0 ppm
14.607
59.989
12.321
13.083

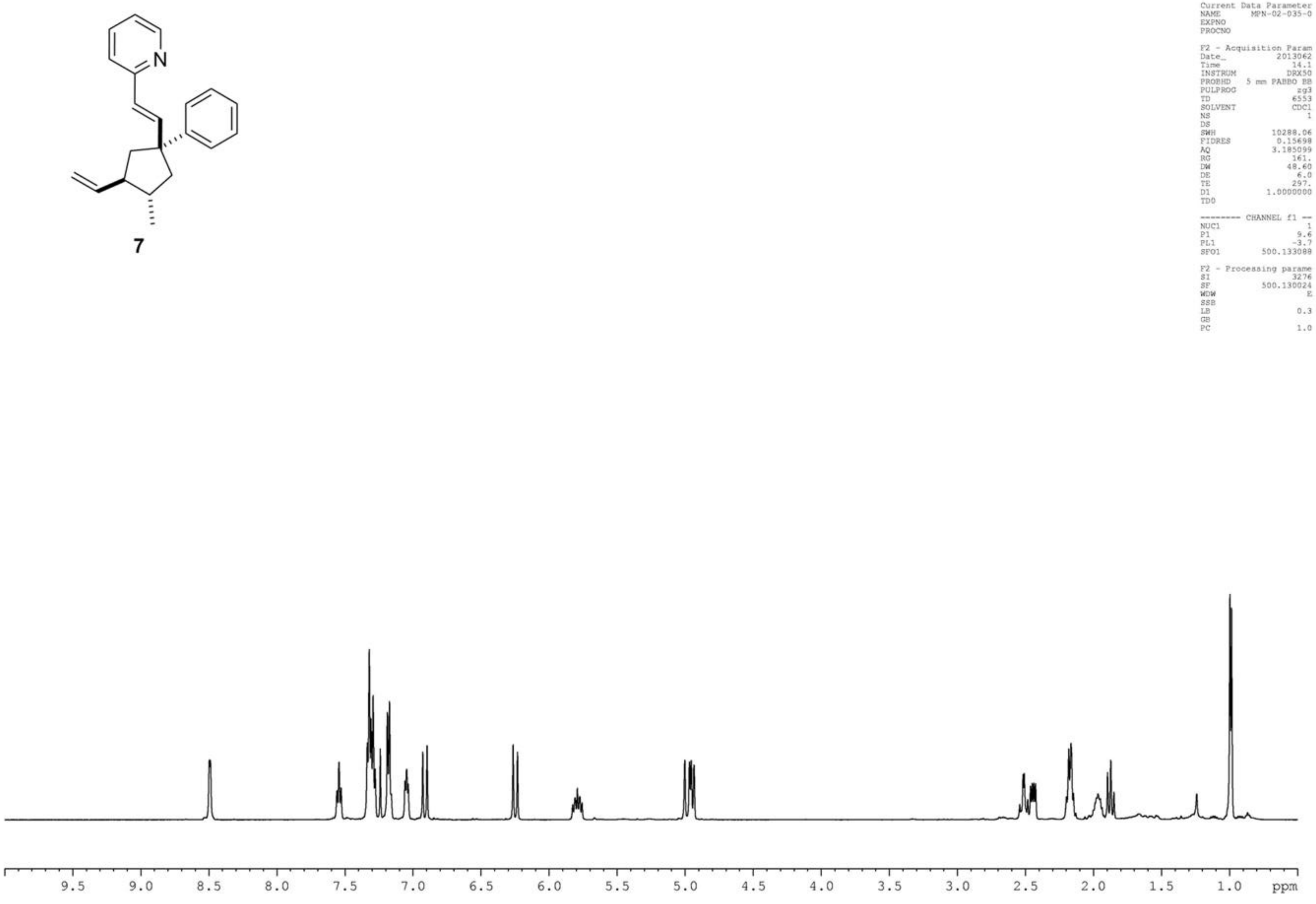
7
Current Data Parameter
NAME MPN-02-035-0
EXPNO
PROCNO
F2 - Acquisition Param
Date_ 2013062
Time 14.1
INSTRUM DRX50
PROBHD 5 mm PABBO BB
PULPROG zg3
TD 6553
SOLVENT CDCl
NS 1
DS
SWH 10288.06
FIDRES 0.15698
AQ 3.185099
RG 161.
DW 48.60
DE 6.0
TE 297.
D1 1.0000000
TD0
======== CHANNEL f1 ==
NUC1 1
P1 9.6
PL1 -3.7
SFO1 500.133088
F2 - Processing parame
SI 3276
SF 500.130024
WDW E
SSB
LB 0.3
GB
PC 1.0
9.5 9.0 8.5 8.0 7.5 7.0 6.5 6.0 5.5 5.0 4.5 4.0 3.5 3.0 2.5 2.0 1.5 1.0 ppm

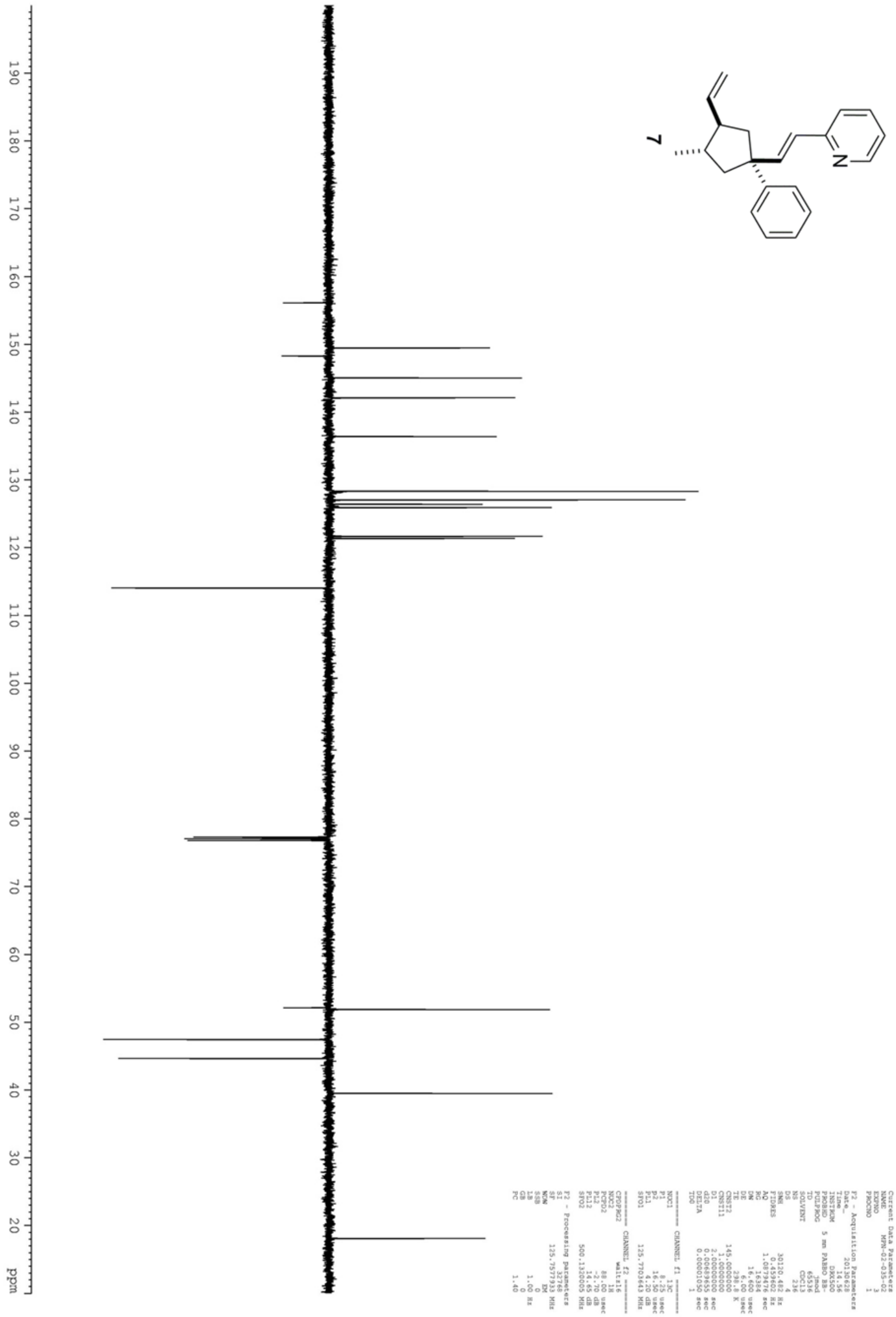

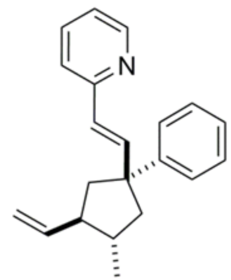

7

COSY

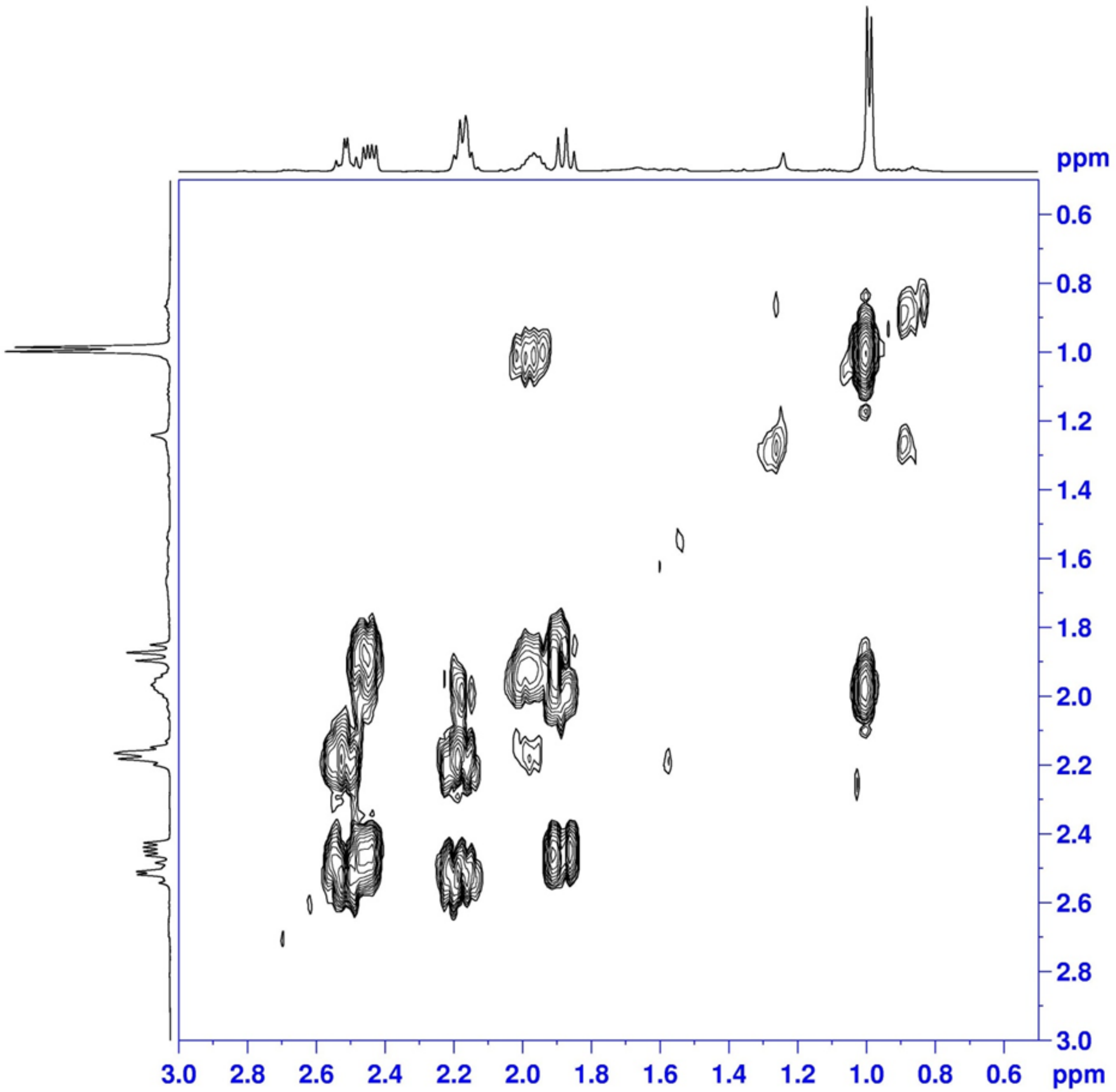


```
Current Data Parameters
NAME        MPN-01-063-05
EXPNO                   4
PROCNO                  1

F2 - Acquisition Parameters
Date_            20130401
Time                10.13
INSTRUM            DRX500
PROBHD   5 mm PABBO BB-
PULPROG        cosygpmfqf
TD                   2048
SOLVENT             CDCl3
NS                      2
DS                      8
SWH              6684.492 Hz
FIDRES           3.263912 Hz
AQ              0.1532404 sec
RG                  26008
DW                 74.800 usec
DE                   6.00 usec
TE                  291.2 K
D0             0.00000300 sec
D1             2.00000000 sec
d13            0.00000400 sec
D16            0.00020000 sec
IN0            0.00014960 sec

======== CHANNEL f1 ========
NUC1                   1H
P1                   9.65 usec
PL1                 -3.70 dB
SFO1          500.1322506 MHz

====== GRADIENT CHANNEL =====
GPNAM1           SINE.100
GPNAM2           SINE.100
GPNAM3           SINE.100
GPZ1                16.00 %
GPZ2                12.00 %
GPZ3                40.00 %
P16               1000.00 usec

F1 - Acquisition parameters
ND0                     1
TD                    128
SFO1             500.1323 MHz
FIDRES          52.222595 Hz
SW                 13.365 ppm
FnMODE                 QF

F2 - Processing parameters
SI                   1024
SF            500.1300116 MHz
WDW                  SINE
SSB                     0
LB                   0.00 Hz
GB                      0
PC                   1.40

F1 - Processing parameters
SI                   1024
MC2                    QF
SF            500.1300109 MHz
WDW                  SINE
SSB                     0
LB                   0.00 Hz
GB                      0
```

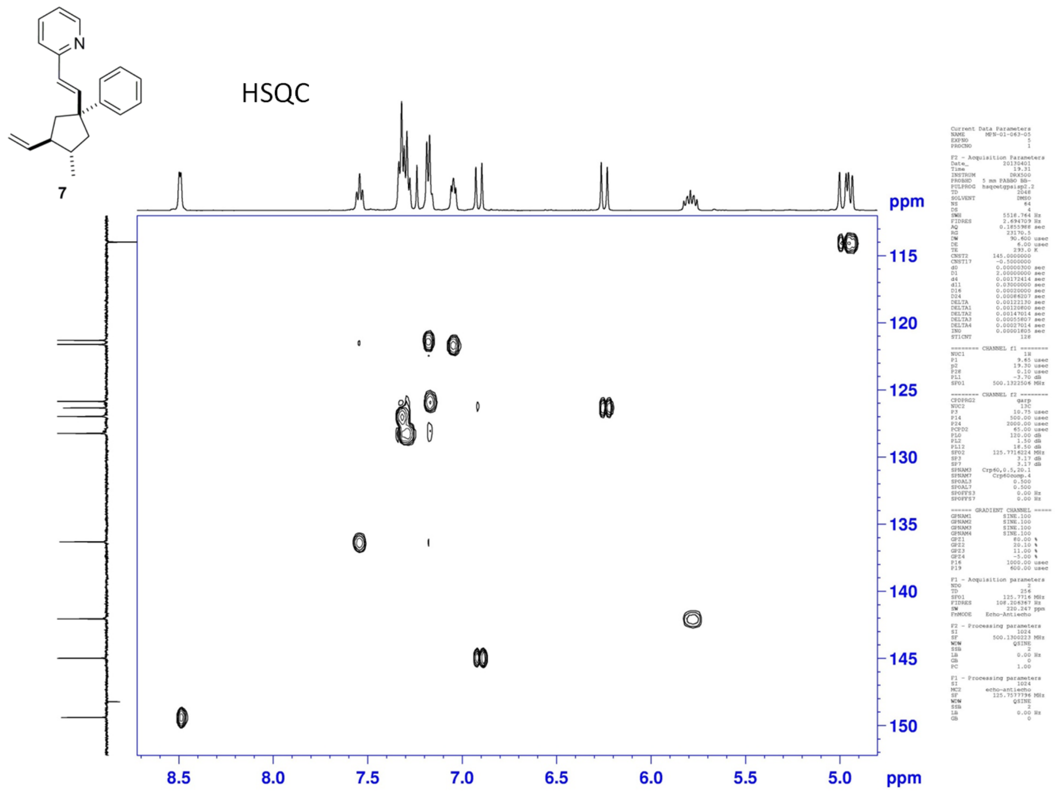
HSQC
7
ppm
115
120
125
130
135
140
145
150
8.5
8.0
7.5
7.0
6.5
6.0
5.5
5.0
ppm

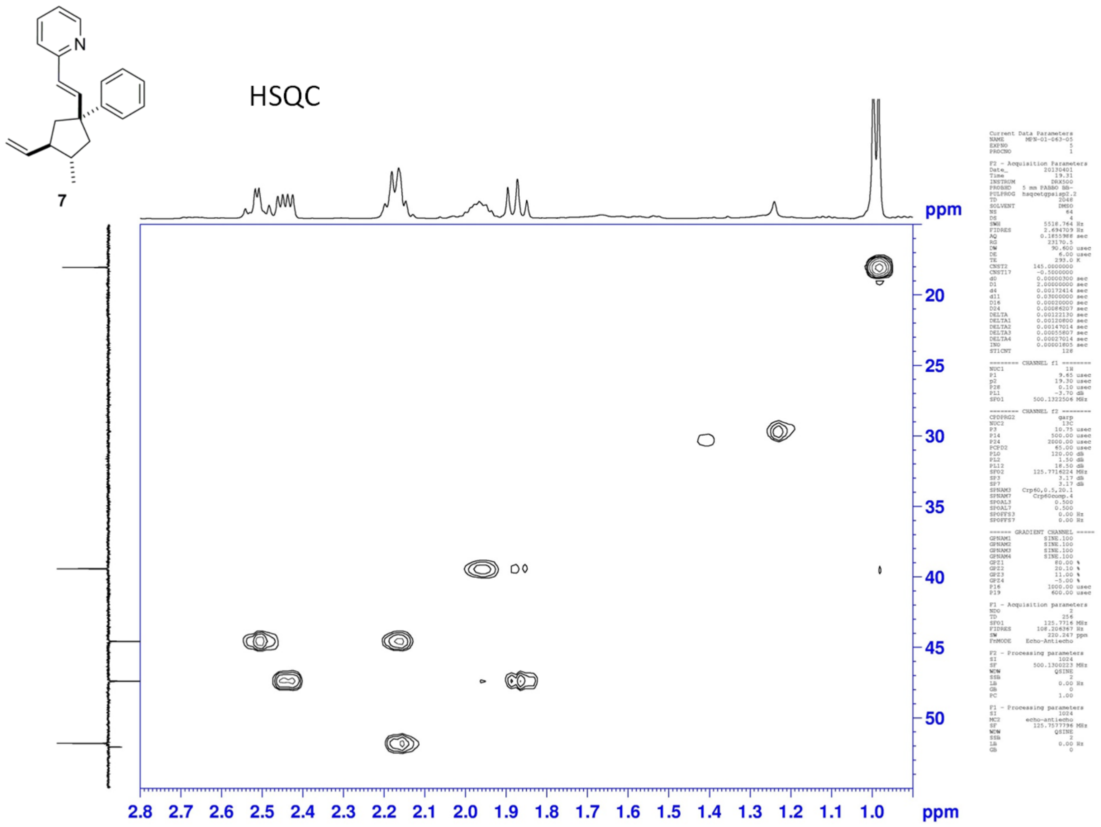
HSQC
7
ppm
20
25
30
35
40
45
50
2.8
2.7
2.6
2.5
2.4
2.3
2.2
2.1
2.0
1.9
1.8
1.7
1.6
1.5
1.4
1.3
1.2
1.1
1.0
ppm

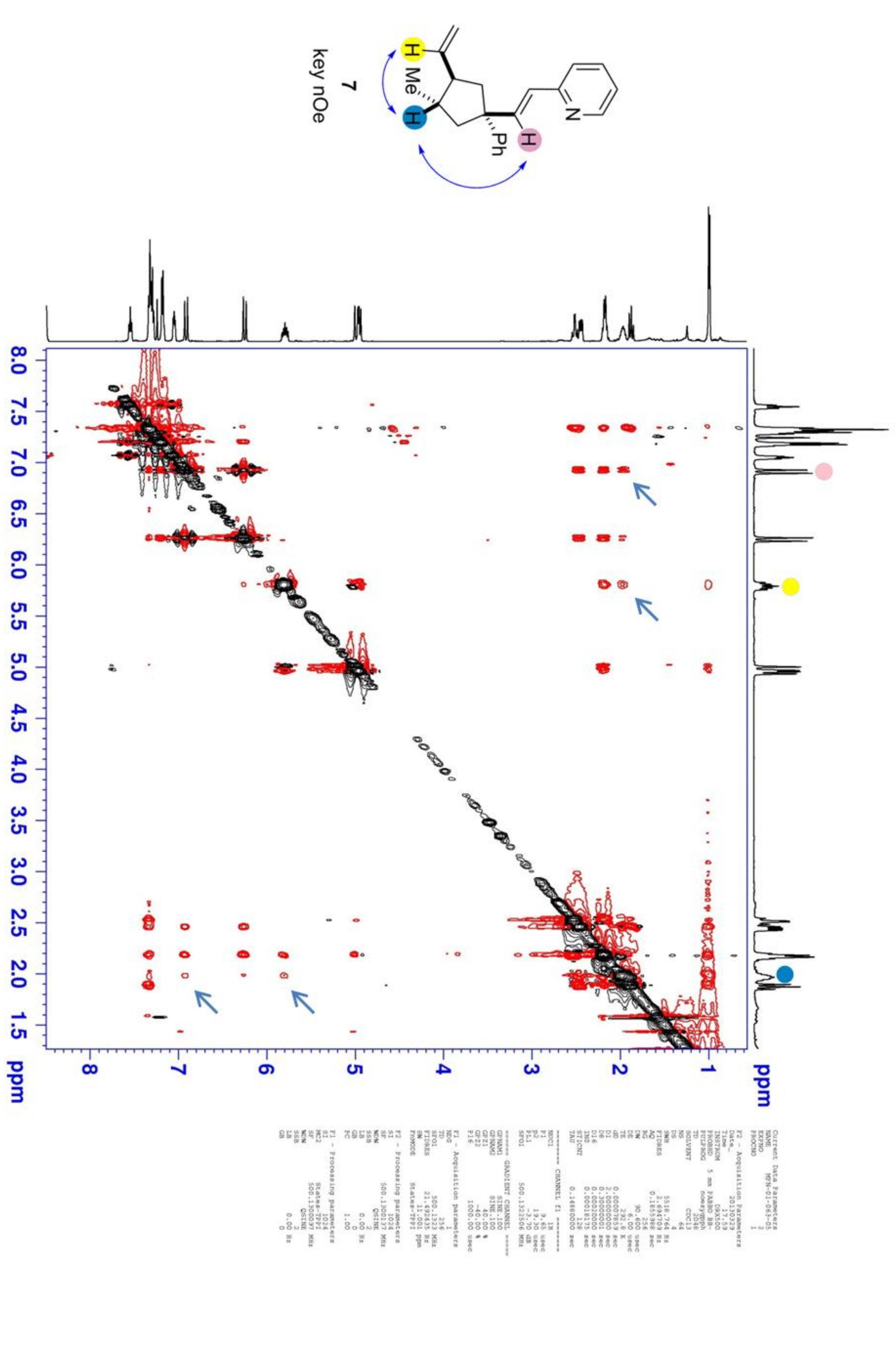

7
key nOe
ppm
ppm

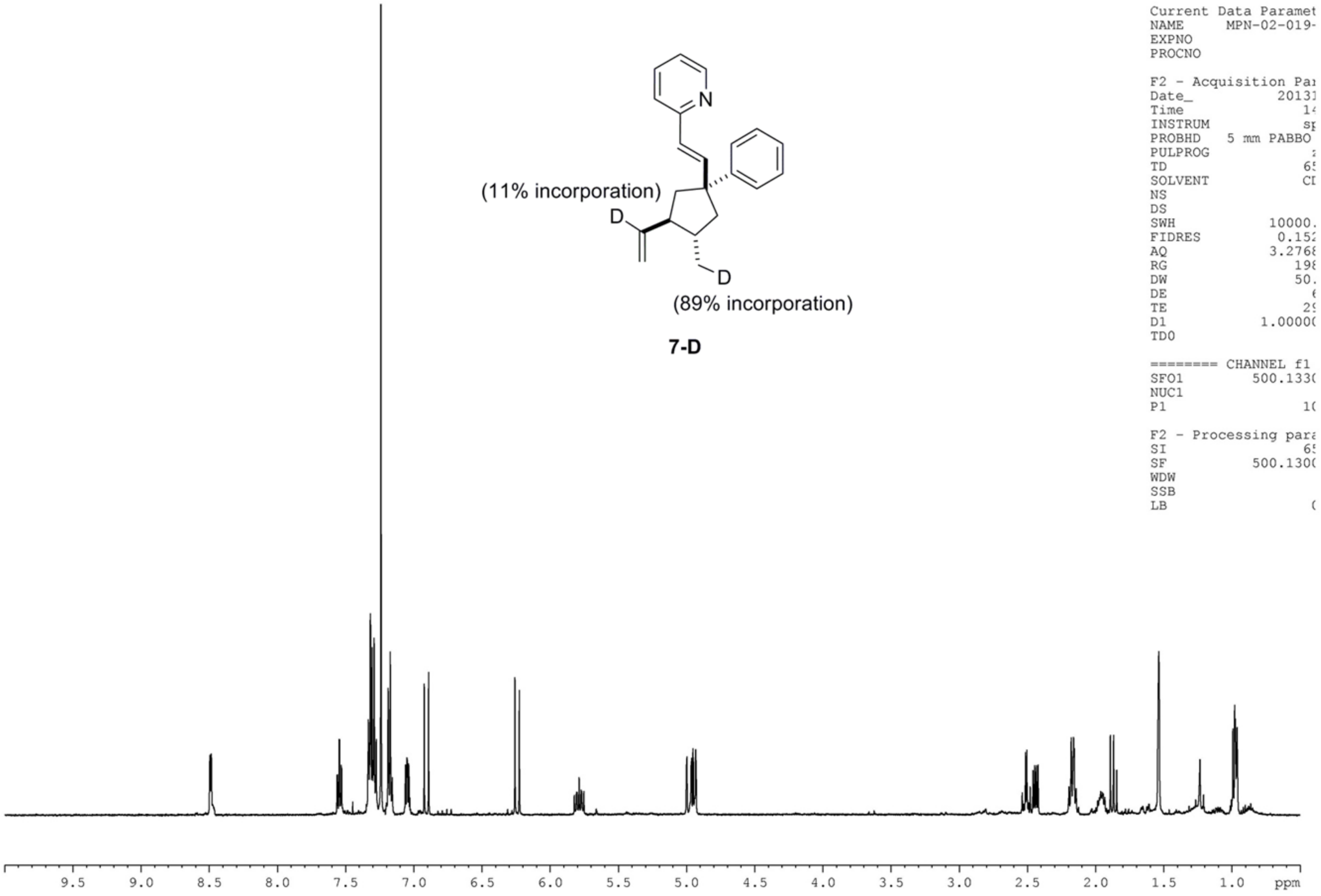
(11% incorporation)
D
N
D
(89% incorporation)
7-D
Current Data Paramet
NAME MPN-02-019-
EXPNO
PROCNO
F2 - Acquisition Pa
Date_ 2013
Time
INSTRUM
PROBHD 5 mm PABBO
PULPROG
TD
SOLVENT
NS
DS
SWH 10000.
FIDRES 0.15
AQ 3.276
RG 19
DW 50.
DE
TE
D1 1.0000
TD0
======== CHANNEL f1
SFO1 500.133
NUC1
P1
F2 - Processing par
SI
SF 500.130
WDW
SSB
LB
9.5 9.0 8.5 8.0 7.5 7.0 6.5 6.0 5.5 5.0 4.5 4.0 3.5 3.0 2.5 2.0 1.5 1.0 ppm

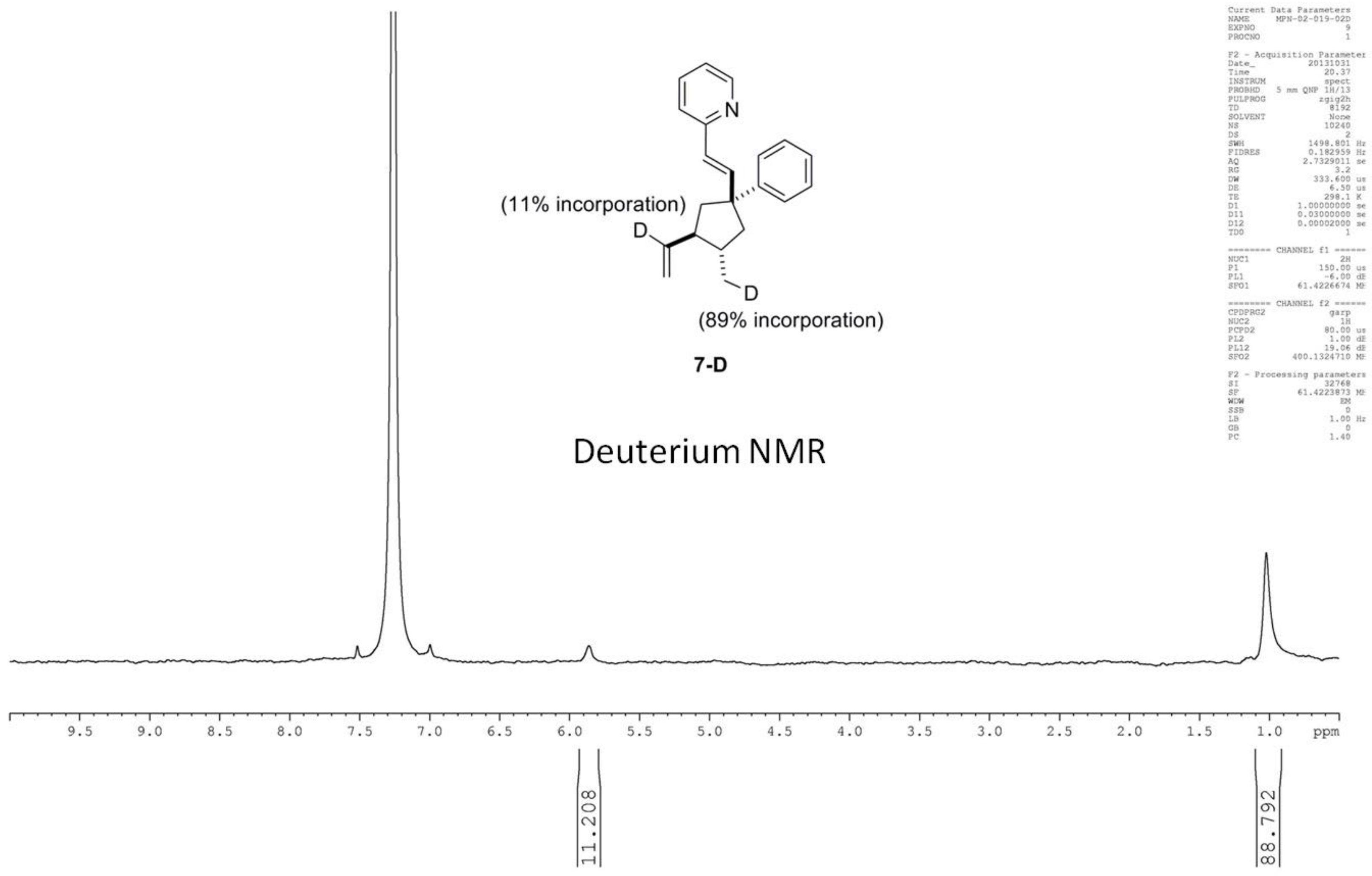
(11% incorporation)
D
N
D
(89% incorporation)
7-D
Deuterium NMR
Current Data Parameters
NAME MPN-02-019-02D
EXPNO 9
PROCNO 1
F2 - Acquisition Parameter
Date_ 20131031
Time 20.37
INSTRUM spect
PROBHD 5 mm QNP 1H/13
PULPROG zgig2h
TD 8192
SOLVENT None
NS 10240
DS 2
SWH 1498.801 Hz
FIDRES 0.182959 Hz
AQ 2.7329011 se
RG 3.2
DW 333.600 us
DE 6.50 us
TE 298.1 K
D1 1.00000000 se
D11 0.03000000 se
D12 0.00002000 se
TD0 1
======== CHANNEL f1 ======
NUC1 2H
P1 150.00 us
PL1 -6.00 dE
SFO1 61.4226674 MH
======== CHANNEL f2 ======
CPDPRG2 garp
NUC2 1H
PCPD2 80.00 us
PL2 1.00 dE
PL12 19.06 dE
SFO2 400.1324710 MH
F2 - Processing parameters
SI 32768
SF 61.4223873 MH
WDW EM
SSB 0
LB 1.00 Hz
GB 0
PC 1.40
9.5 9.0 8.5 8.0 7.5 7.0 6.5 6.0 5.5 5.0 4.5 4.0 3.5 3.0 2.5 2.0 1.5 1.0 ppm
11.208
88.792

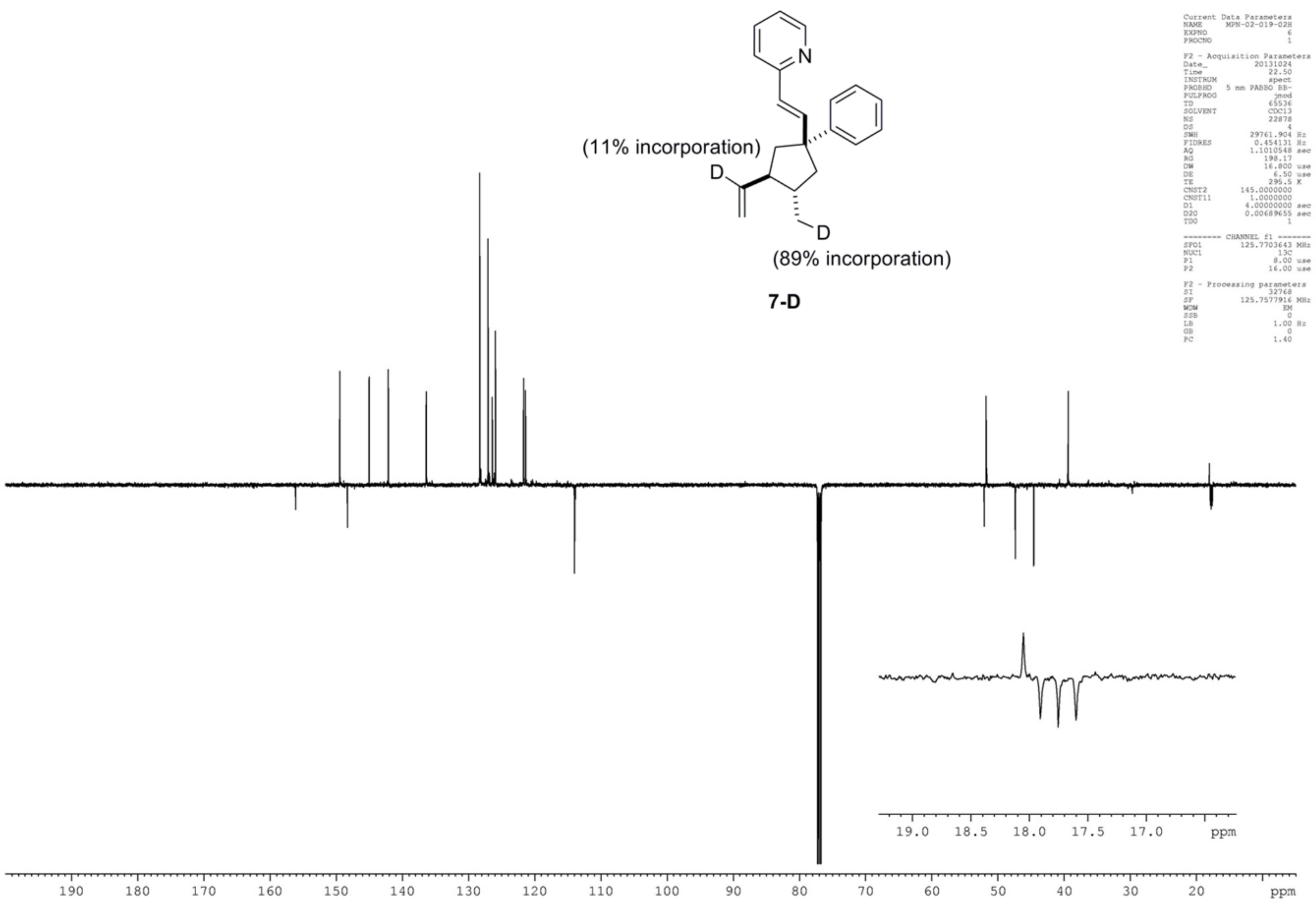
(11% incorporation)
D
N
D
(89% incorporation)
7-D
Current Data Parameters
NAME MPN-02-019-02H
EXPNO 6
PROCNO 1
F2 - Acquisition Parameters
Date_ 20131024
Time 22.50
INSTRUM spect
PROBHD 5 mm PABBO BB-
PULPROG jmod
TD 65536
SOLVENT CDC13
NS 22878
DS 4
SWH 29761.904 Hz
FIDRES 0.454131 Hz
AQ 1.1010548 sec
RG 198.17
DW 16.800 use
DE 6.50 use
TE 295.5 K
CNST2 145.0000000
CNST11 1.0000000
D1 4.00000000 sec
D20 0.00689655 sec
TD0 1
======== CHANNEL f1 ========
SFO1 125.7703643 MHz
NUC1 13C
P1 8.00 use
P2 16.00 use
F2 - Processing parameters
SI 32768
SF 125.7577916 MHz
WDW EM
SSB 0
LB 1.00 Hz
GB 0
PC 1.40
19.0 18.5 18.0 17.5 17.0 ppm
190 180 170 160 150 140 130 120 110 100 90 80 70 60 50 40 30 20 ppm

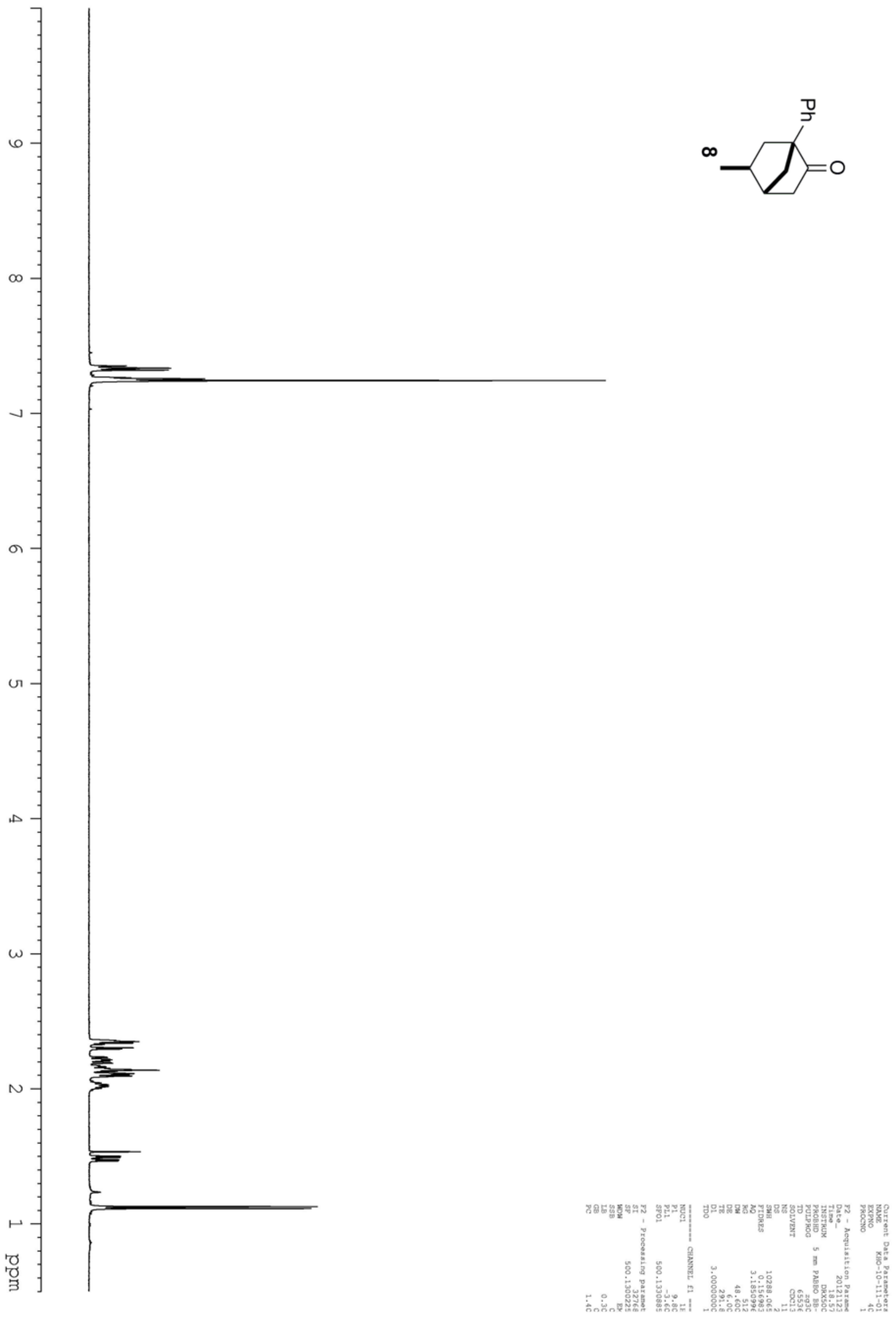

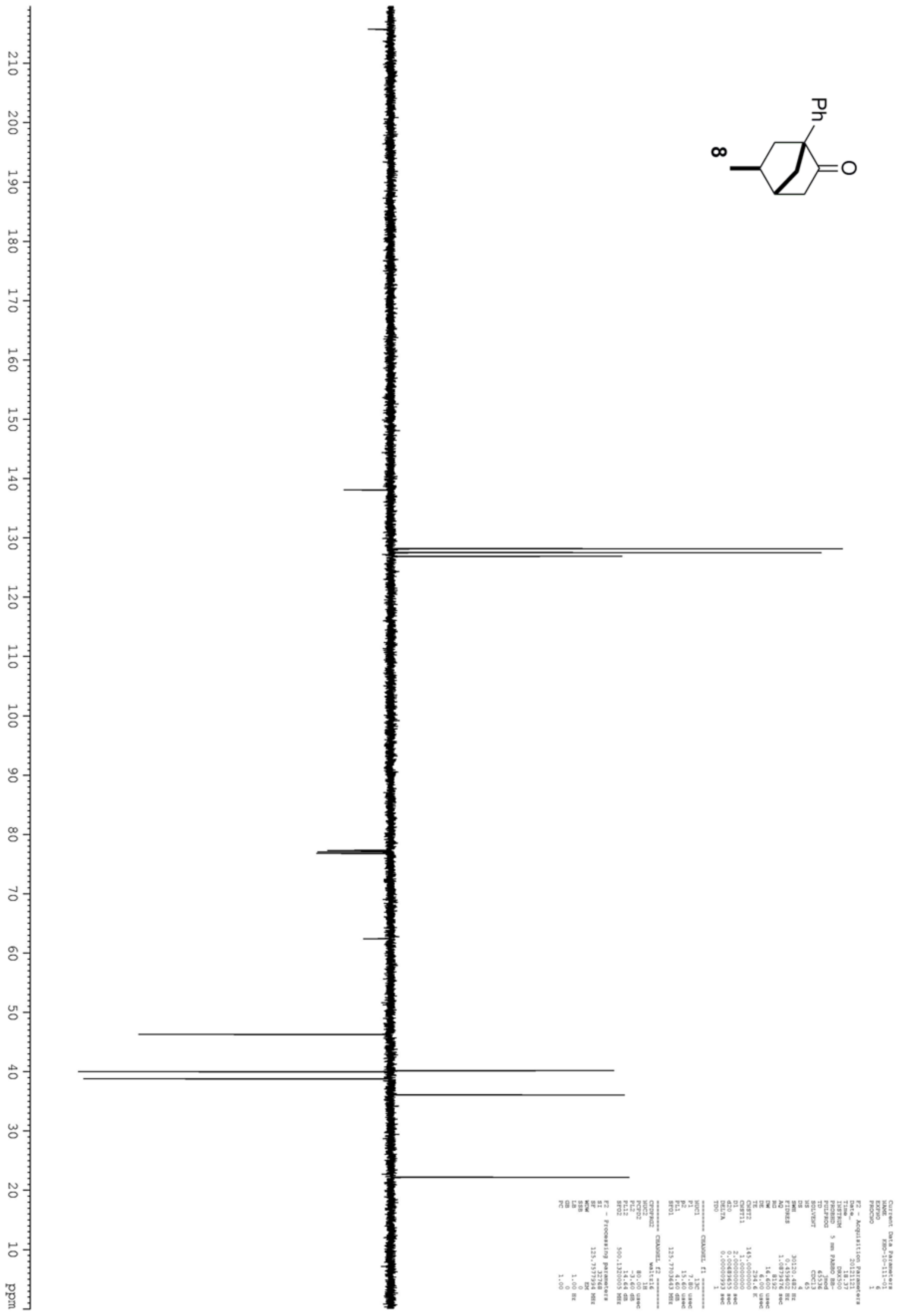

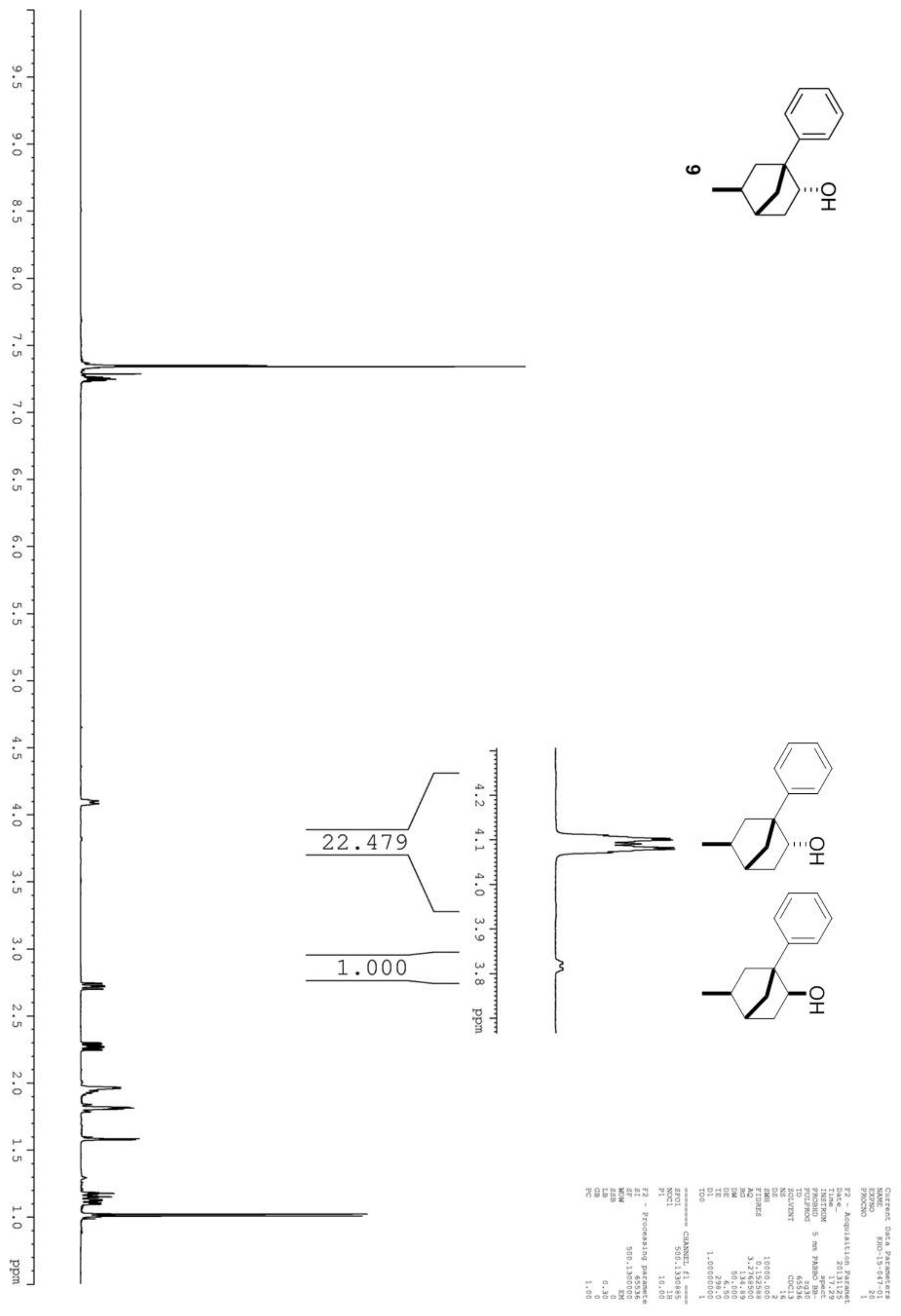
9
OH
22.479
1.000
ppm

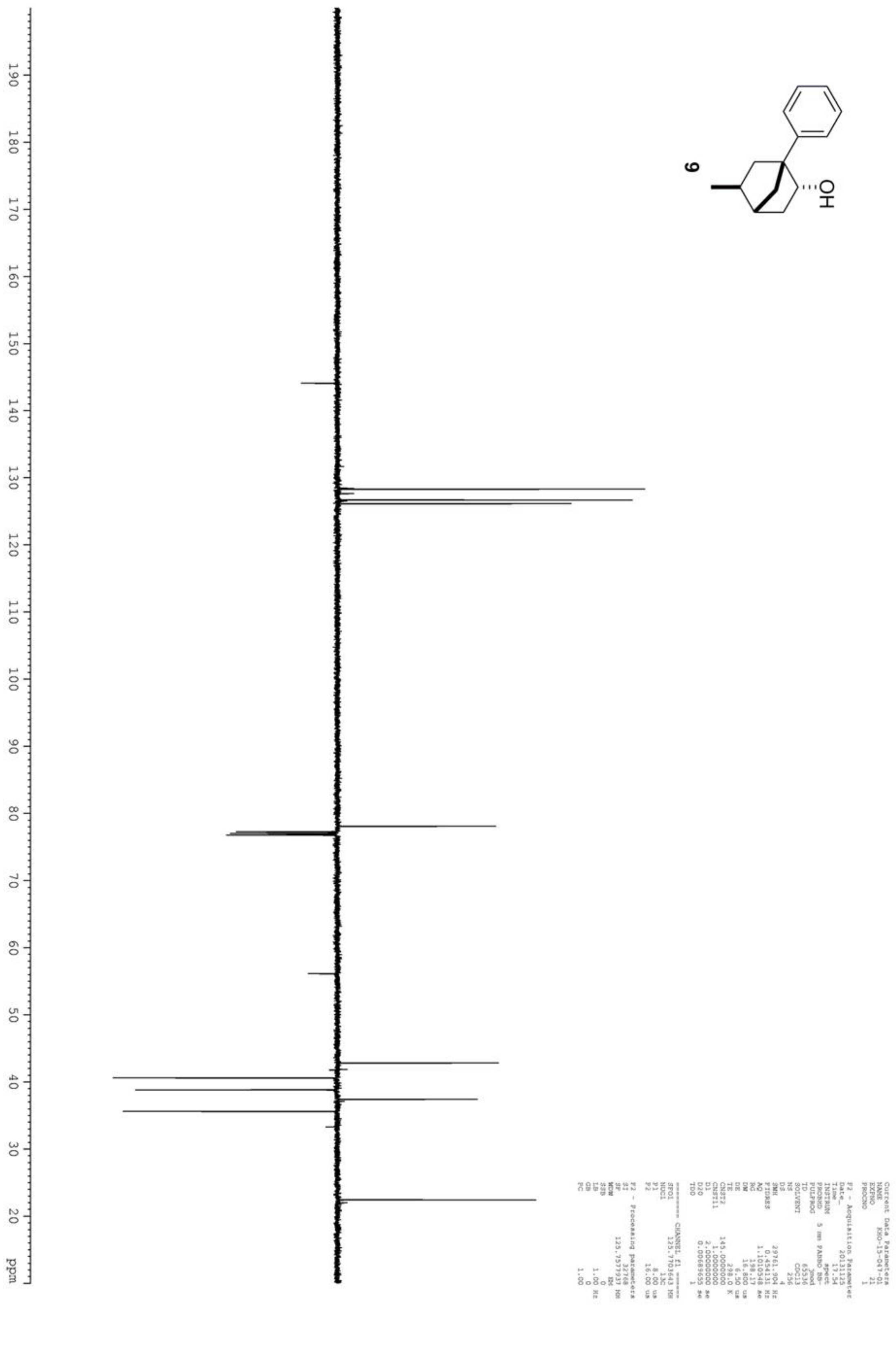

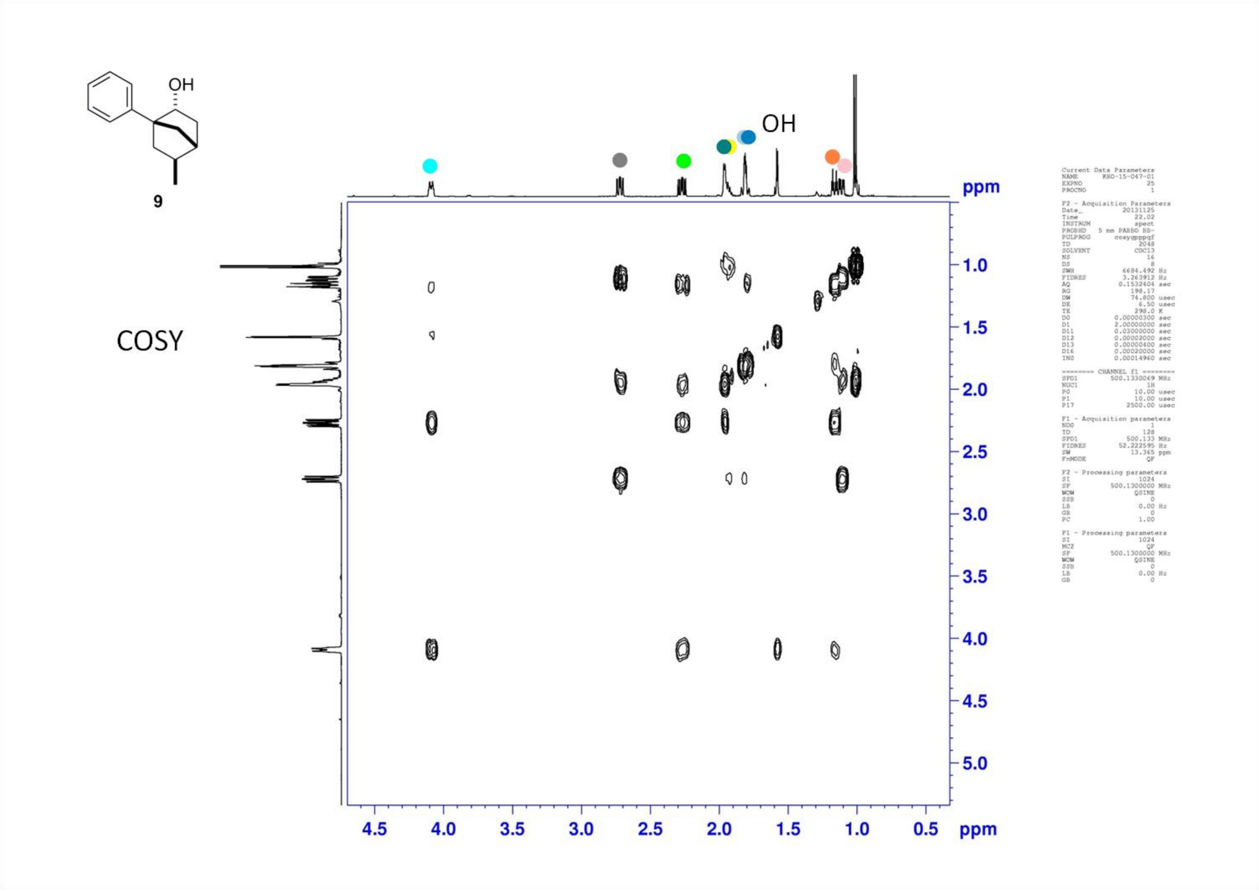
OH
9
OH
COSY
ppm
1.0
1.5
2.0
2.5
3.0
3.5
4.0
4.5
5.0
4.5
4.0
3.5
3.0
2.5
2.0
1.5
1.0
0.5
ppm

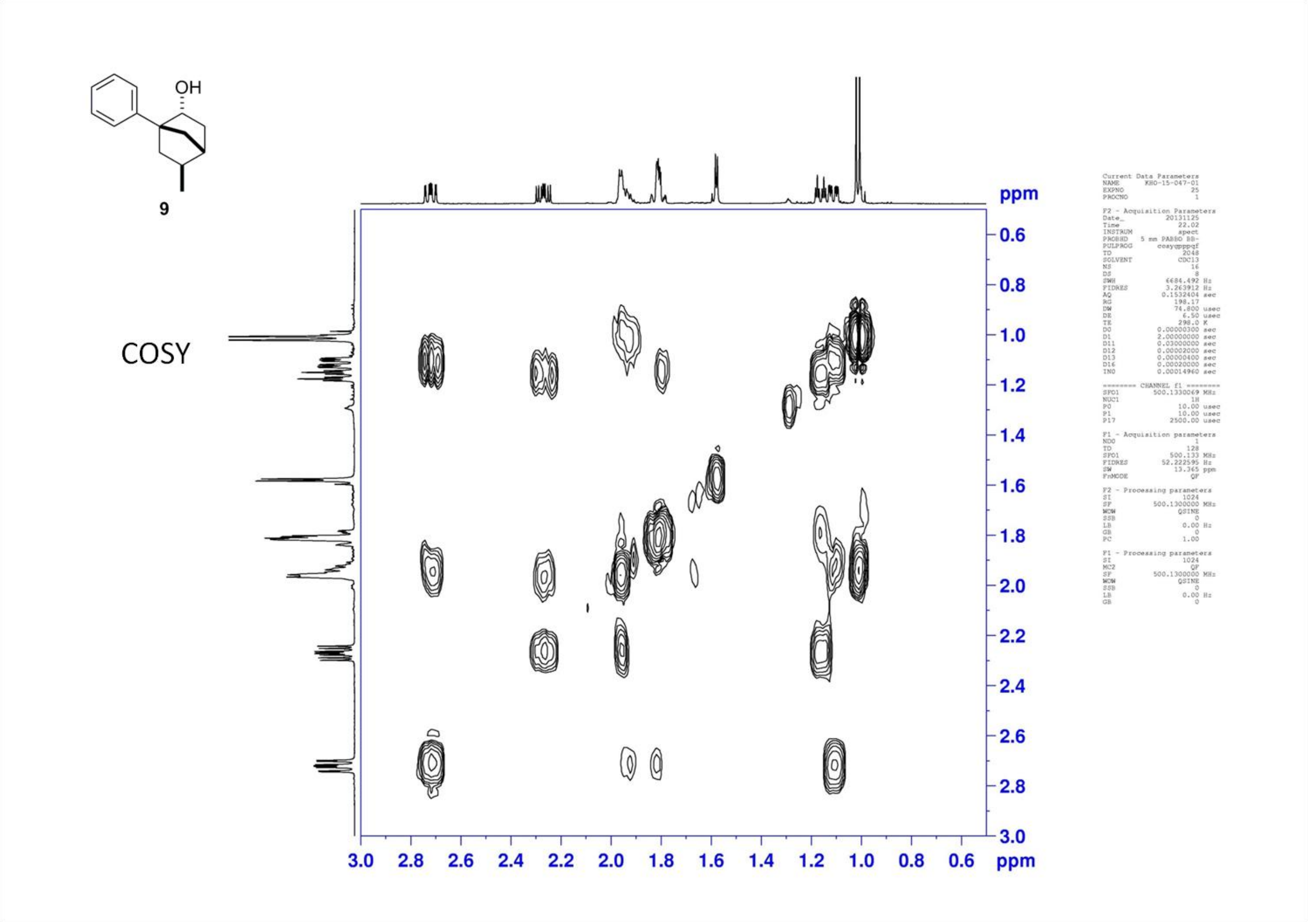
OH
9
COSY
ppm
0.6
0.8
1.0
1.2
1.4
1.6
1.8
2.0
2.2
2.4
2.6
2.8
3.0
3.0
2.8
2.6
2.4
2.2
2.0
1.8
1.6
1.4
1.2
1.0
0.8
0.6
ppm

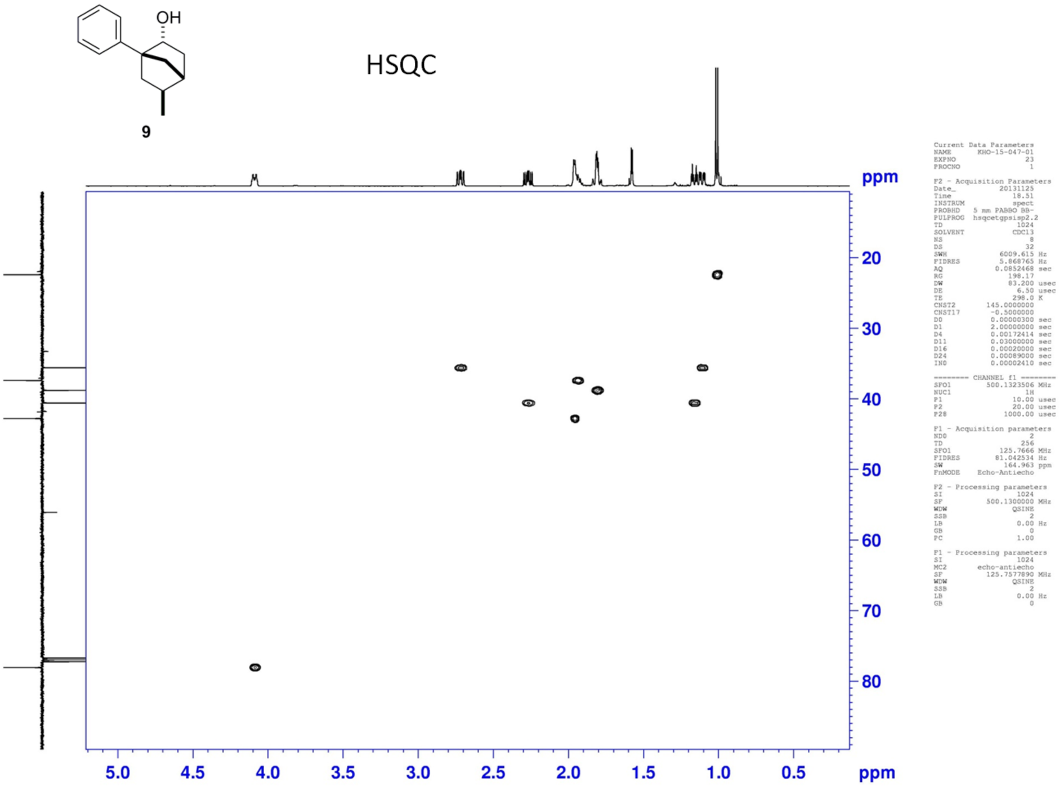
OH
9
HSQC
ppm
20
30
40
50
60
70
80
5.0
4.5
4.0
3.5
3.0
2.5
2.0
1.5
1.0
0.5
ppm

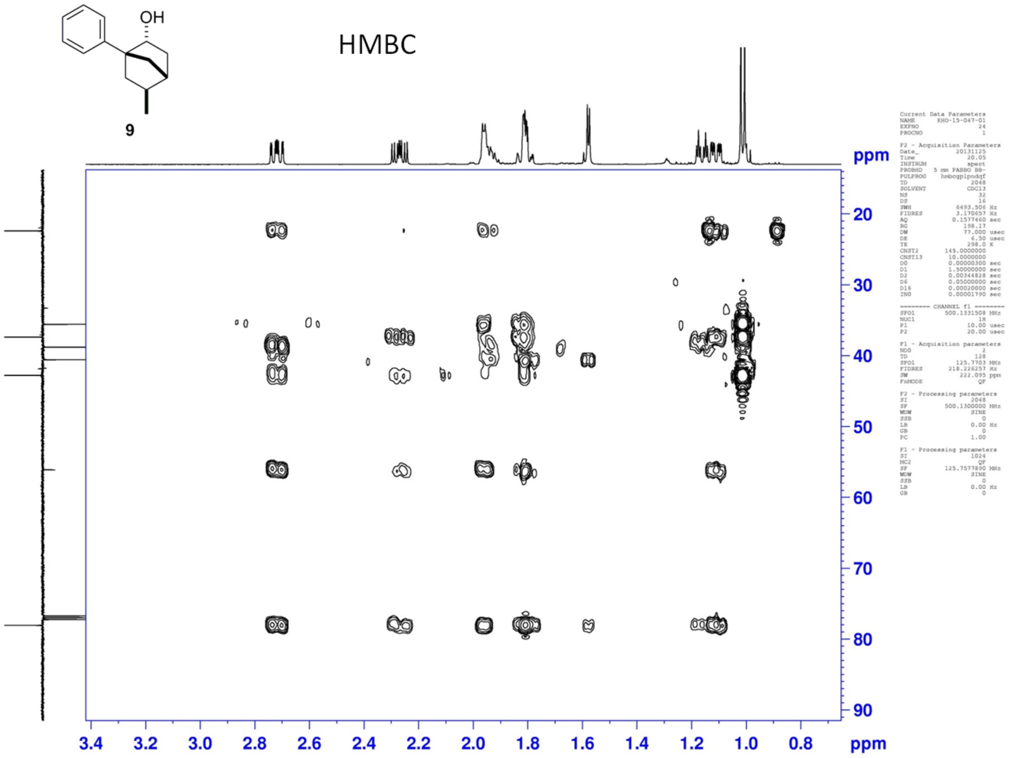
OH
9
HMBC
ppm
20
30
40
50
60
70
80
90
3.4
3.2
3.0
2.8
2.6
2.4
2.2
2.0
1.8
1.6
1.4
1.2
1.0
0.8
ppm

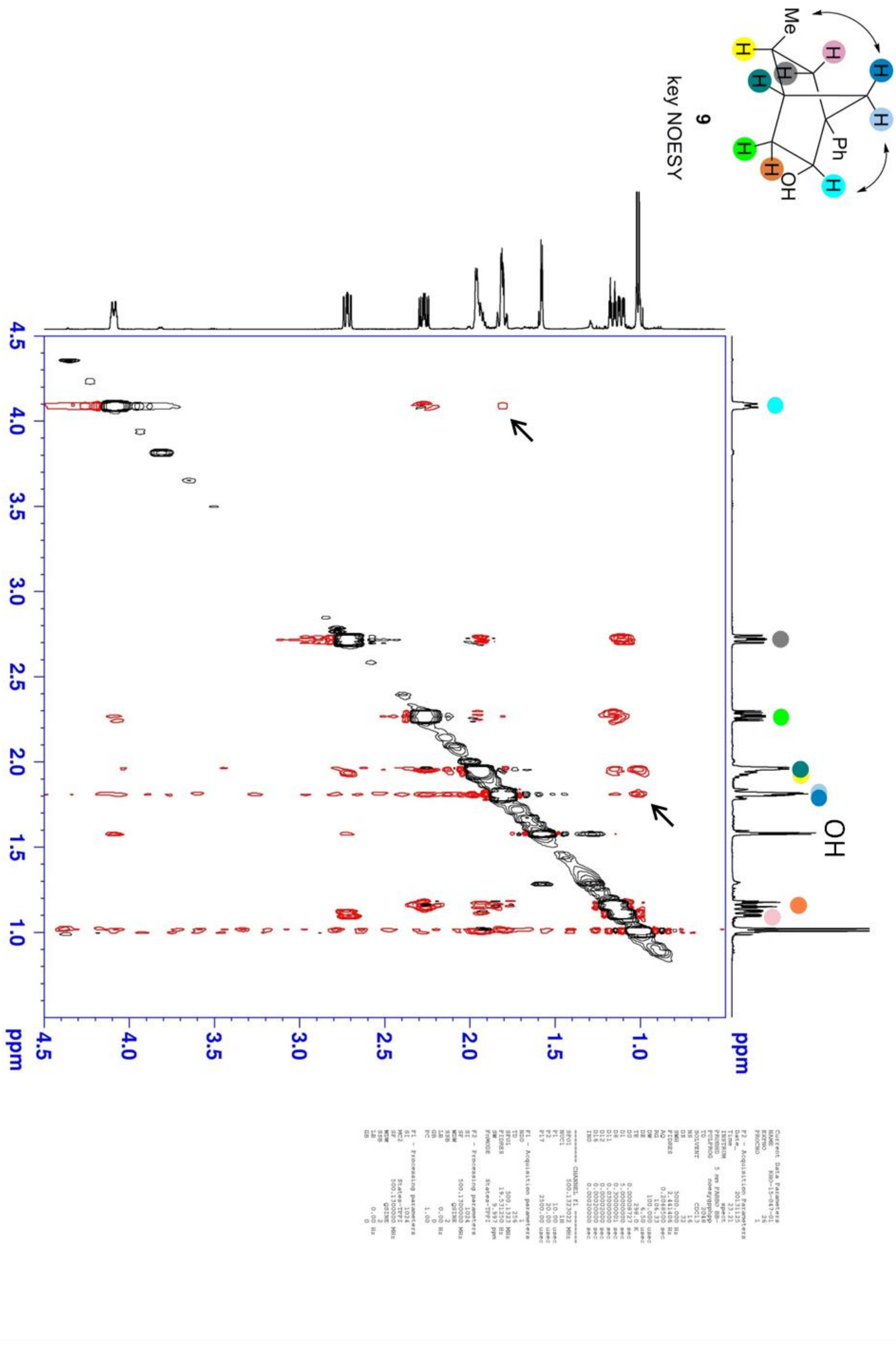
9
key NOESY
Me
Ph
OH
ppm

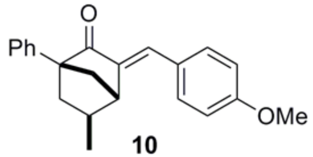

10

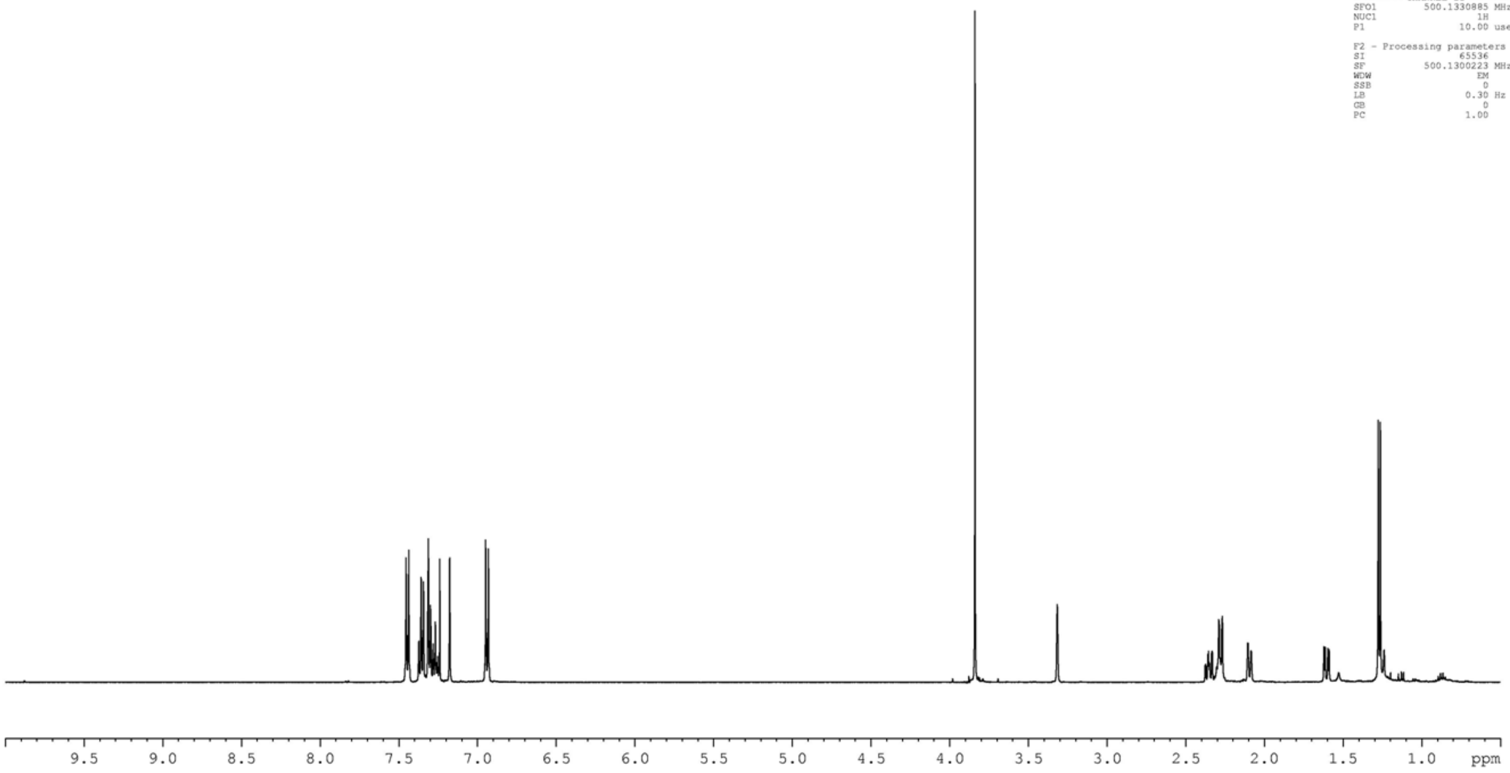


```
Current Data Parameters
NAME       CRA-04-037-01
EXPNO                  4
PROCNO                 1

F2 - Acquisition Parameters
Date_           20140207
Time               18.04
INSTRUM            spect
PROBHD   5 mm PABBO BB-
PULPROG             zg30
TD                 65536
SOLVENT            CDCl3
NS                    16
DS                     2
SWH          10000.000 Hz
FIDRES        0.152588 Hz
AQ           3.2768500 sec
RG              198.17
DW              50.000 usec
DE                6.50 usec
TE               298.0 K
D1          1.00000000 sec
TD0                    1

======== CHANNEL f1 ========
SFO1        500.1330885 MHz
NUC1                 1H
P1                10.00 usec

F2 - Processing parameters
SI                 65536
SF          500.1300223 MHz
WDW                   EM
SSB                    0
LB                  0.30 Hz
GB                     0
PC                  1.00
```

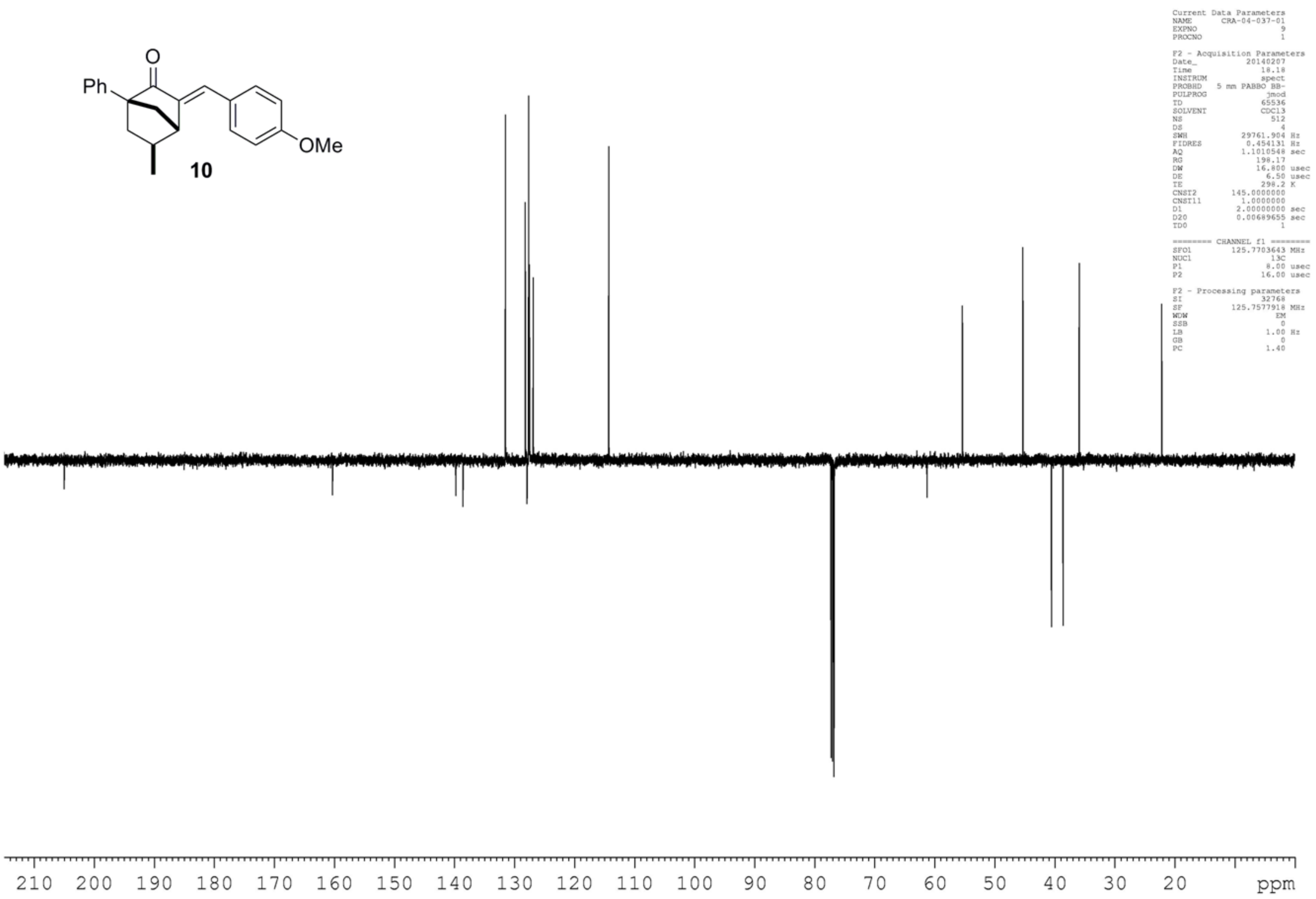
Ph
O
OMe
10
Current Data Parameters
NAME CRA-04-037-01
EXPNO 9
PROCNO 1
F2 - Acquisition Parameters
Date_ 20140207
Time 18.18
INSTRUM spect
PROBHD 5 mm PABBO BB-
PULPROG jmod
TD 65536
SOLVENT CDCl3
NS 512
DS 4
SWH 29761.904 Hz
FIDRES 0.454131 Hz
AQ 1.1010548 sec
RG 198.17
DW 16.800 usec
DE 6.50 usec
TE 298.2 K
CNST2 145.0000000
CNST11 1.0000000
D1 2.00000000 sec
D20 0.00689655 sec
TD0 1
======== CHANNEL f1 ========
SFO1 125.7703643 MHz
NUC1 13C
P1 8.00 usec
P2 16.00 usec
F2 - Processing parameters
SI 32768
SF 125.7577918 MHz
WDW EM
SSB 0
LB 1.00 Hz
GB 0
PC 1.40
210 200 190 180 170 160 150 140 130 120 110 100 90 80 70 60 50 40 30 20 ppm

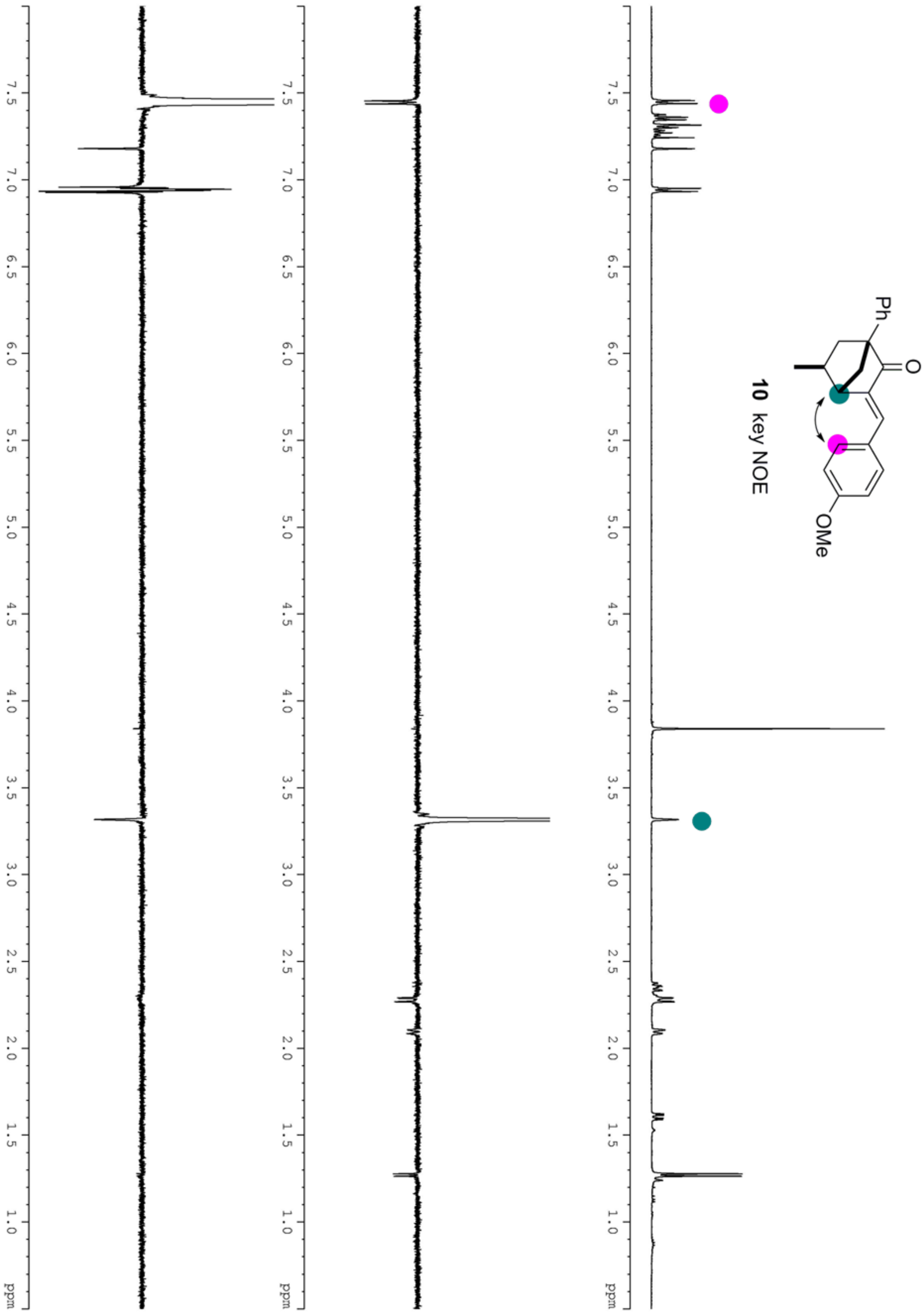
Ph
O
OMe
10 key NOE
7.5
7.0
6.5
6.0
5.5
5.0
4.5
4.0
3.5
3.0
2.5
2.0
1.5
1.0
ppm

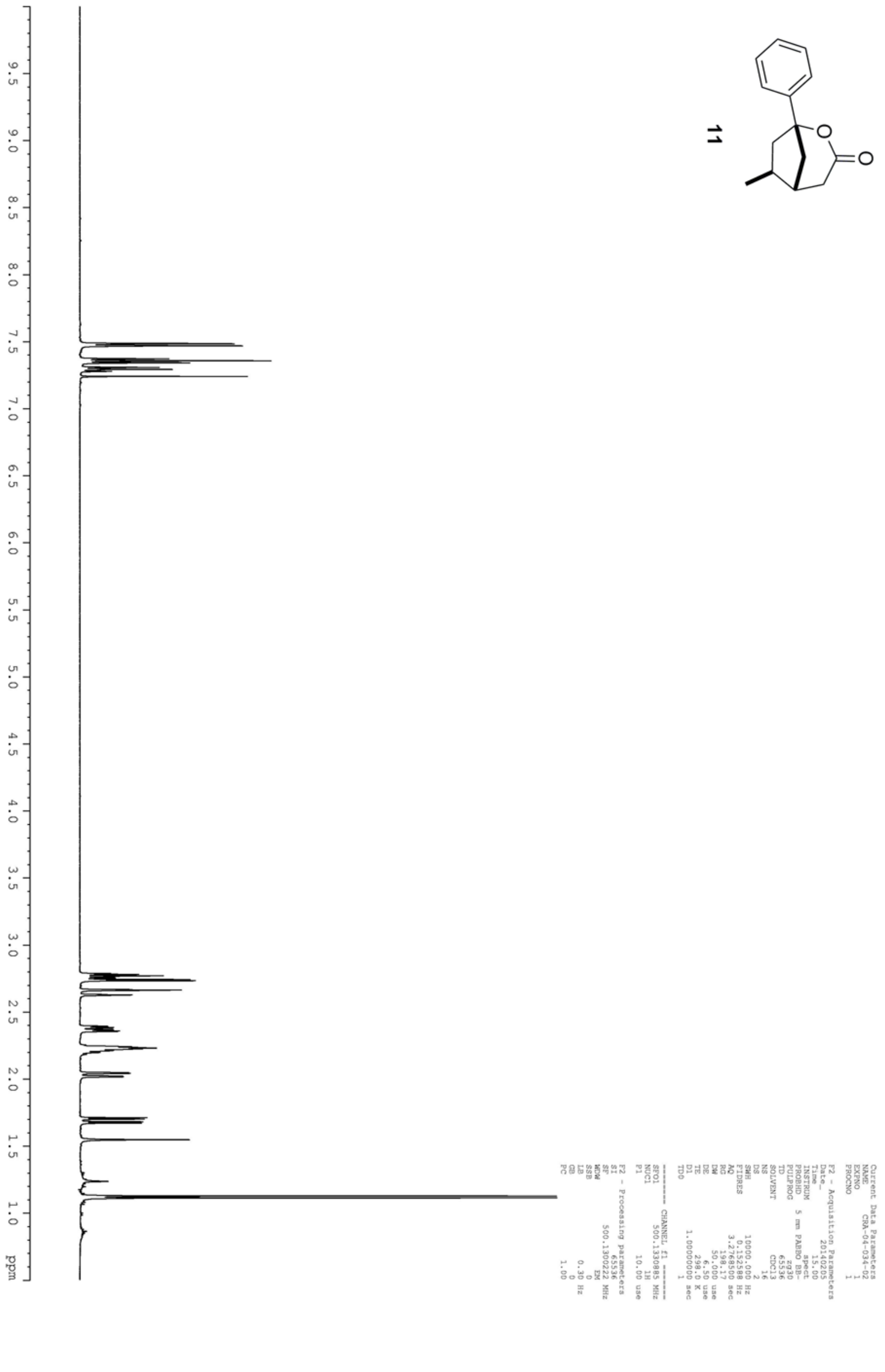

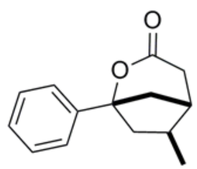

11

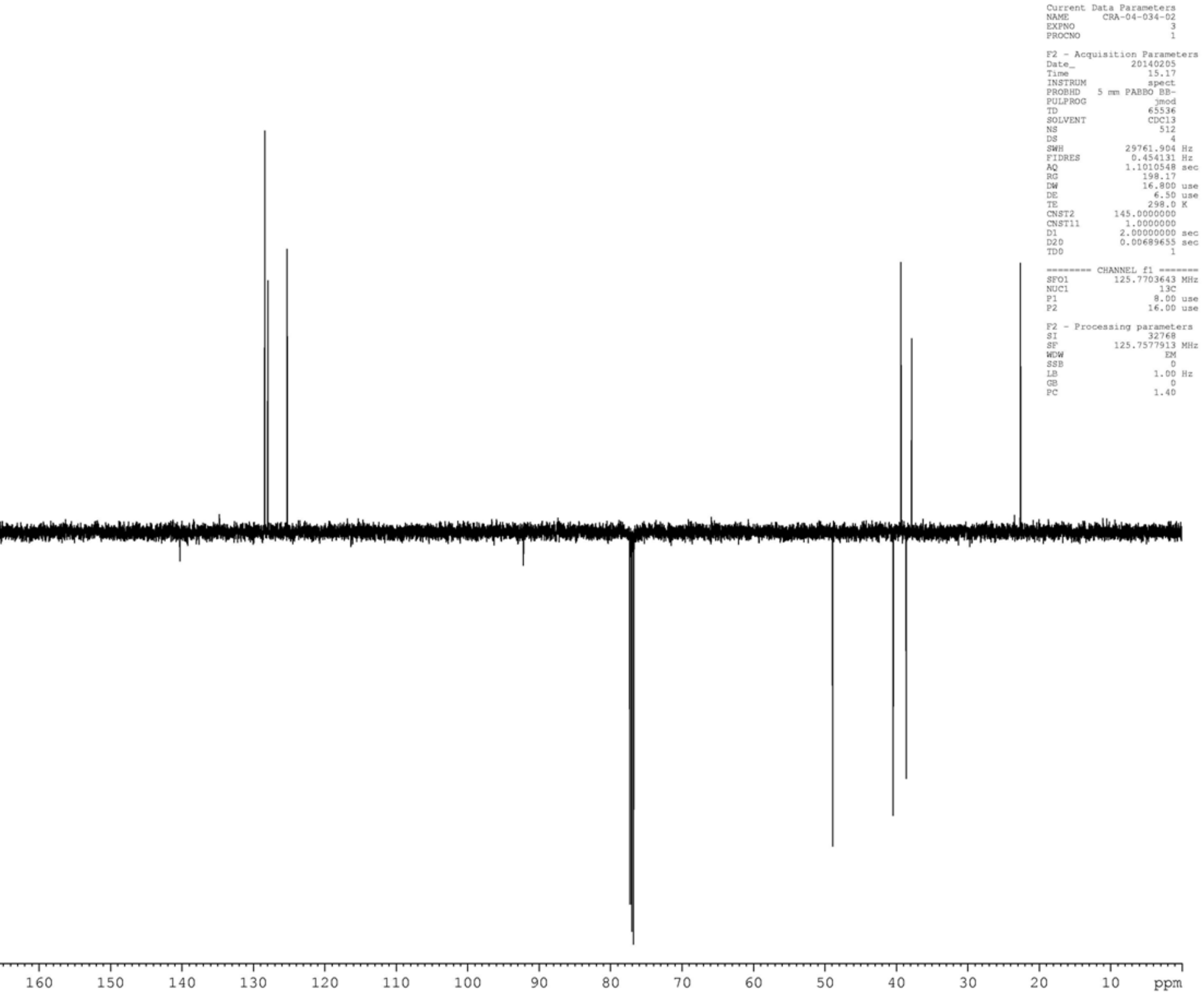
Current Data Parameters
NAME CRA-04-034-02
EXPNO 3
PROCNO 1
F2 - Acquisition Parameters
Date_ 20140205
Time 15.17
INSTRUM spect
PROBHD 5 mm PABBO BB-
PULPROG jmod
TD 65536
SOLVENT CDCl3
NS 512
DS 4
SWH 29761.904 Hz
FIDRES 0.454131 Hz
AQ 1.1010548 sec
RG 198.17
DW 16.800 use
DE 6.50 use
TE 298.0 K
CNST2 145.0000000
CNST11 1.0000000
D1 2.00000000 sec
D20 0.00689655 sec
TD0 1
======== CHANNEL f1 ========
SFO1 125.7703643 MHz
NUC1 13C
P1 8.00 use
P2 16.00 use
F2 - Processing parameters
SI 32768
SF 125.7577913 MHz
WDW EM
SSB 0
LB 1.00 Hz
GB 0
PC 1.40
190
180
170
160
150
140
130
120
110
100
90
80
70
60
50
40
30
20
10
ppm

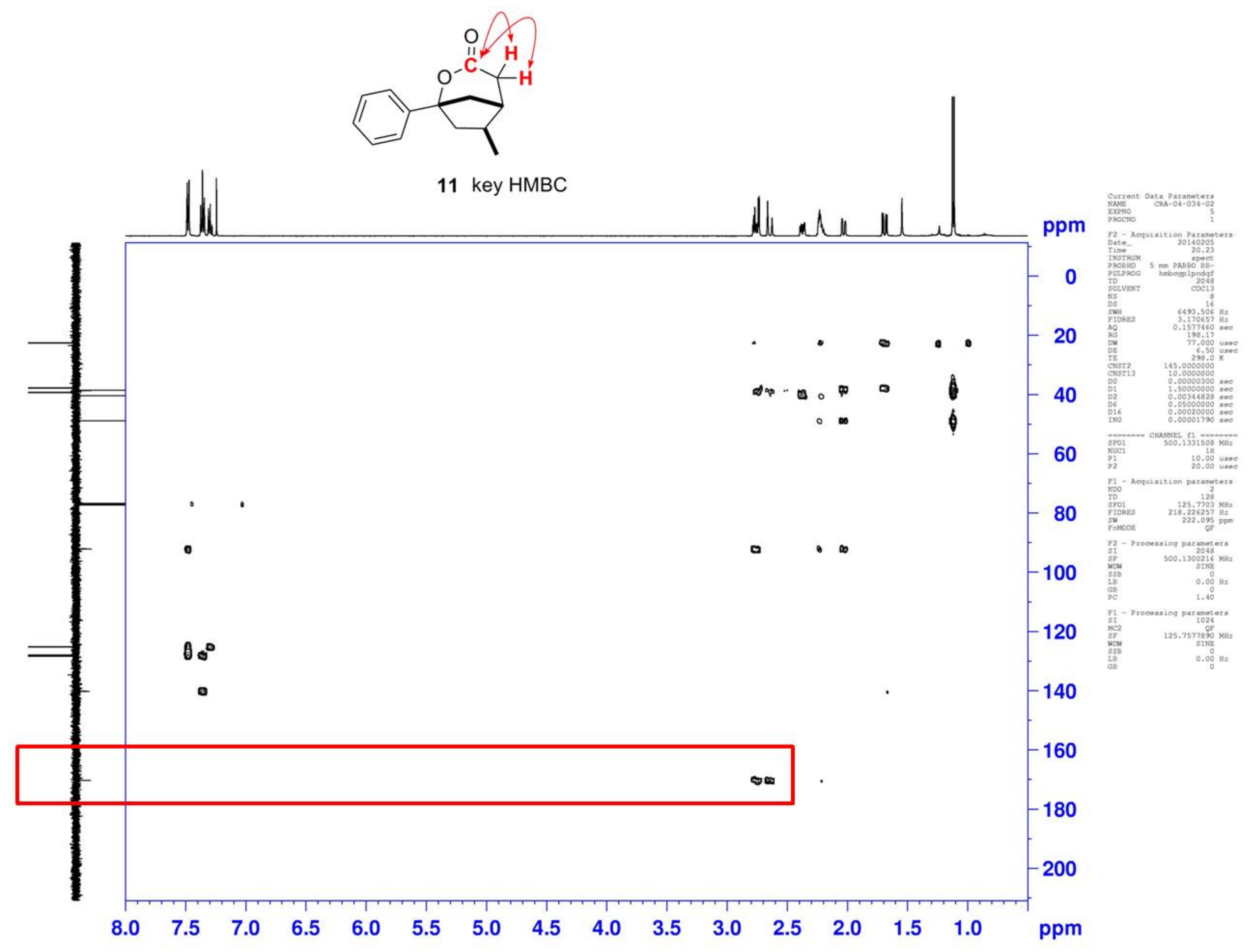
11 key HMBC
ppm
0
20
40
60
80
100
120
140
160
180
200
8.0 7.5 7.0 6.5 6.0 5.5 5.0 4.5 4.0 3.5 3.0 2.5 2.0 1.5 1.0
ppm
Current Data Parameters
NAME CRA-04-034-02
EXPNO 5
PROCNO 1
F2 - Acquisition Parameters
Date_ 20140205
Time 20.23
INSTRUM spect
PROBHD 5 mm PABBO BB-
PULPROG hmbcgplpndqf
TD 2048
SOLVENT CDCl3
NS 8
DS 16
SWH 6493.506 Hz
FIDRES 3.170657 Hz
AQ 0.1577460 sec
RG 198.17
DW 77.000 usec
DE 6.50 usec
TE 298.0 K
CNST2 145.0000000
CNST13 10.0000000
D0 0.00000300 sec
D1 1.50000000 sec
D2 0.00344828 sec
D6 0.05000000 sec
D16 0.00020000 sec
IN0 0.00001790 sec
======== CHANNEL f1 ========
SFO1 500.1331508 MHz
NUC1 1H
P1 10.00 usec
P2 20.00 usec
F1 - Acquisition parameters
ND0 2
TD 128
SFO1 125.7703 MHz
FIDRES 218.226257 Hz
SW 222.095 ppm
FnMODE QF
F2 - Processing parameters
SI 2048
SF 500.1300216 MHz
WDW SINE
SSB 0
LB 0.00 Hz
GB 0
PC 1.40
F1 - Processing parameters
SI 1024
MC2 QF
SF 125.7577890 MHz
WDW SINE
SSB 0
LB 0.00 Hz
GB 0

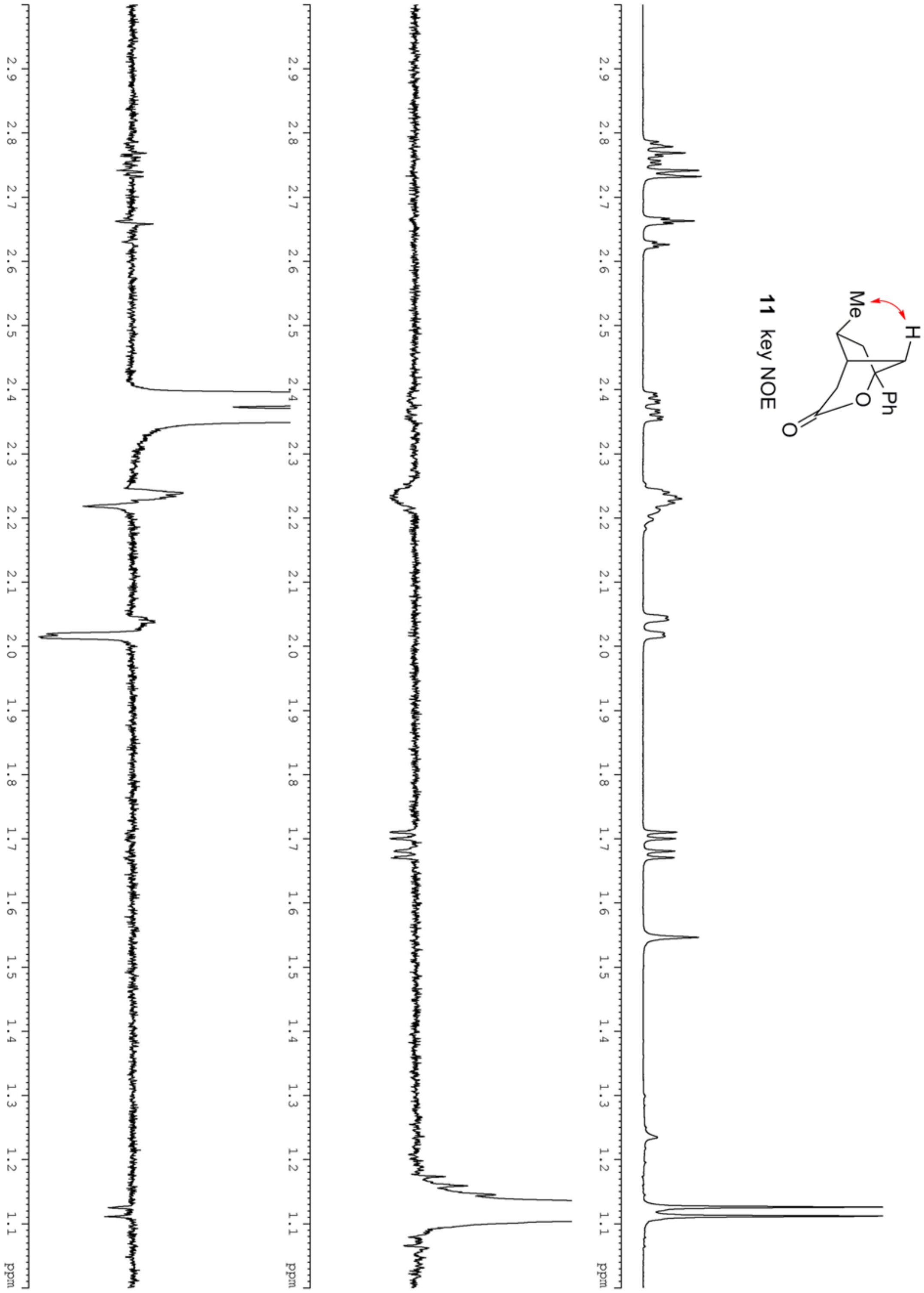
11 key NOE
H
Ph
O
O
Me
ppm

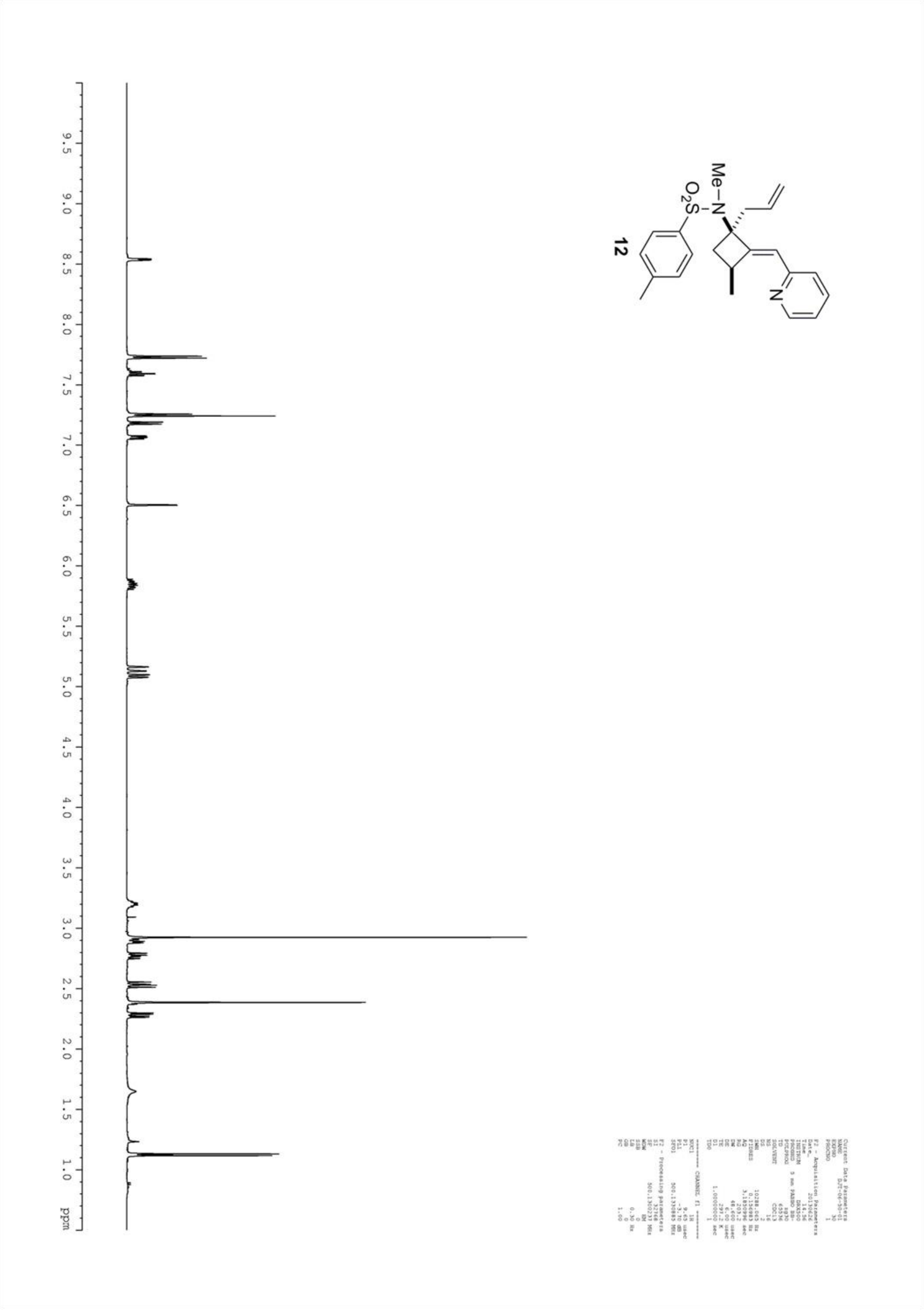
12
ppm

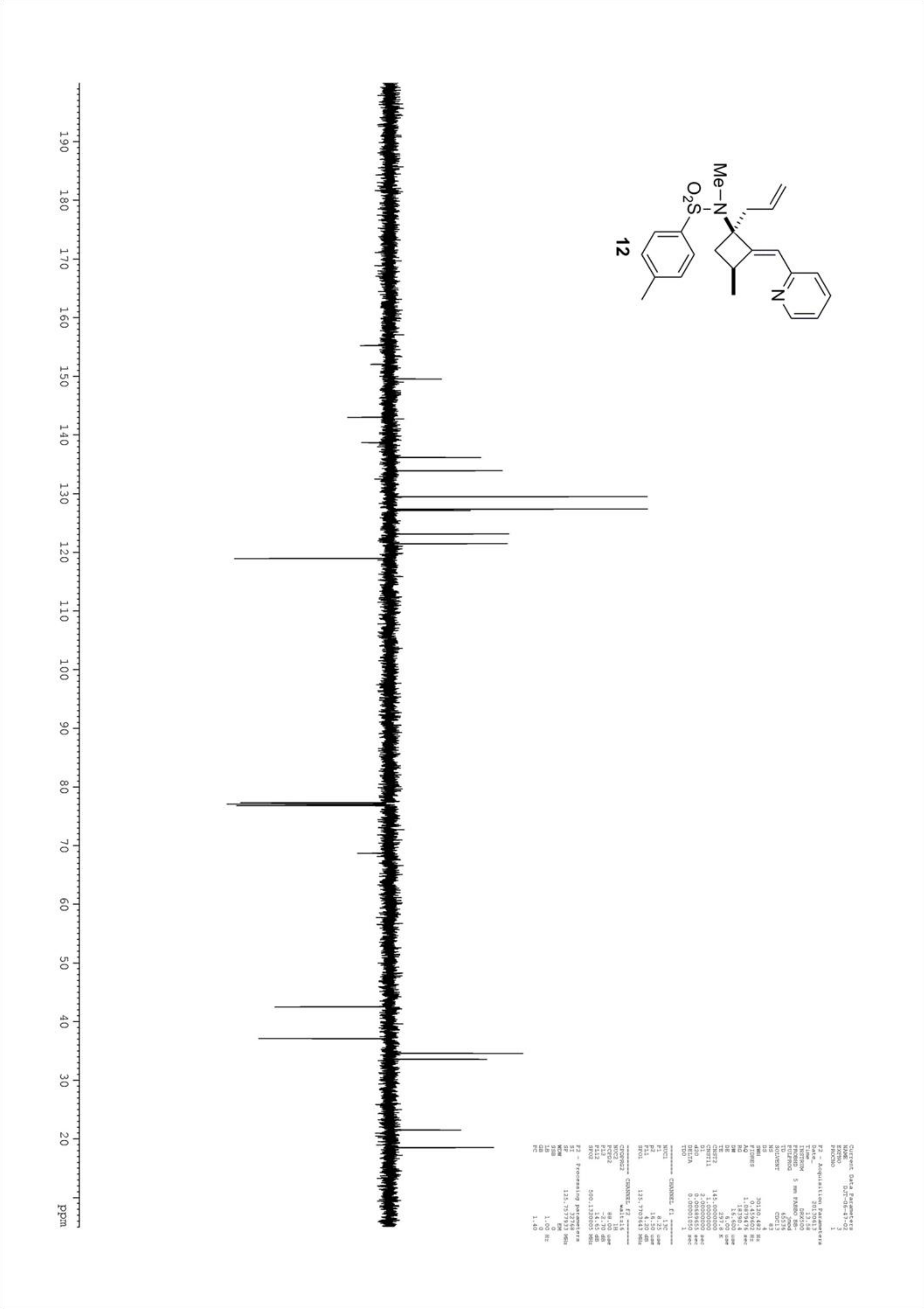
Me–N
O2S
N
12
ppm

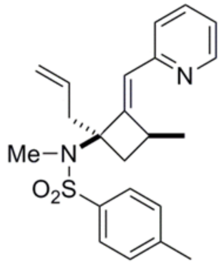
Me−N
O2S
N

12

COSY

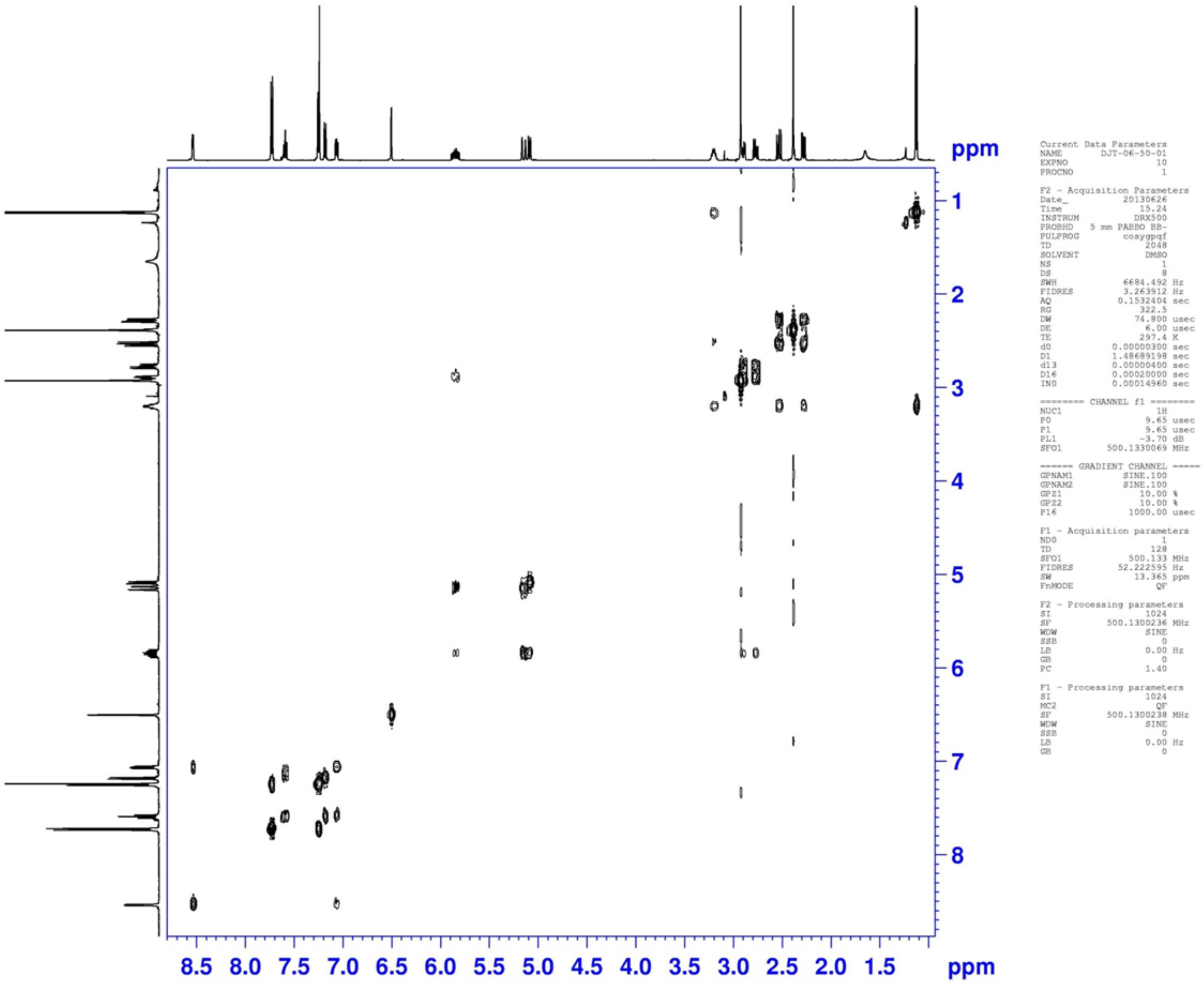
ppm
1
2
3
4
5
6
7
8
8.5
8.0
7.5
7.0
6.5
6.0
5.5
5.0
4.5
4.0
3.5
3.0
2.5
2.0
1.5
ppm
Current Data Parameters
NAME DJT-06-50-01
EXPNO 10
PROCNO 1
F2 - Acquisition Parameters
Date_ 20130626
Time 15.24
INSTRUM DRX500
PROBHD 5 mm PABBO BB-
PULPROG cosygpqf
TD 2048
SOLVENT DMSO
NS 1
DS 8
SWH 6684.492 Hz
FIDRES 3.263912 Hz
AQ 0.1532404 sec
RG 322.5
DW 74.800 usec
DE 6.00 usec
TE 297.4 K
d0 0.00000300 sec
D1 1.48689198 sec
d13 0.00000400 sec
D16 0.00020000 sec
IN0 0.00014960 sec
======== CHANNEL f1 ========
NUC1 1H
P0 9.65 usec
P1 9.65 usec
PL1 -3.70 dB
SFO1 500.1330069 MHz
====== GRADIENT CHANNEL =====
GPNAM1 SINE.100
GPNAM2 SINE.100
GPZ1 10.00 %
GPZ2 10.00 %
P16 1000.00 usec
F1 - Acquisition parameters
ND0 1
TD 128
SFO1 500.133 MHz
FIDRES 52.222595 Hz
SW 13.365 ppm
FnMODE QF
F2 - Processing parameters
SI 1024
SF 500.1300236 MHz
WDW SINE
SSB 0
LB 0.00 Hz
GB 0
PC 1.40
F1 - Processing parameters
SI 1024
MC2 QF
SF 500.1300238 MHz
WDW SINE
SSB 0
LB 0.00 Hz
GB 0

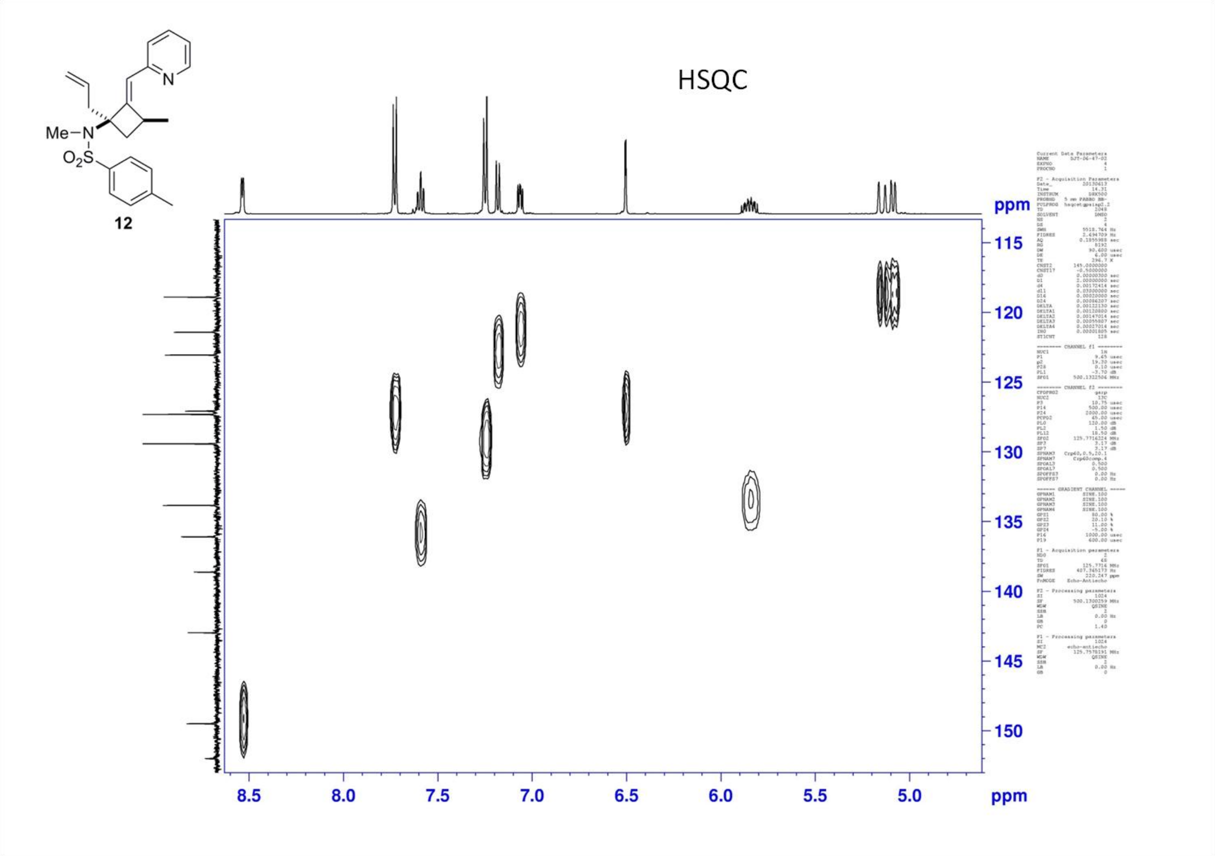
HSQC
Me–N
O_2S
N
12
ppm
115
120
125
130
135
140
145
150
8.5
8.0
7.5
7.0
6.5
6.0
5.5
5.0
ppm

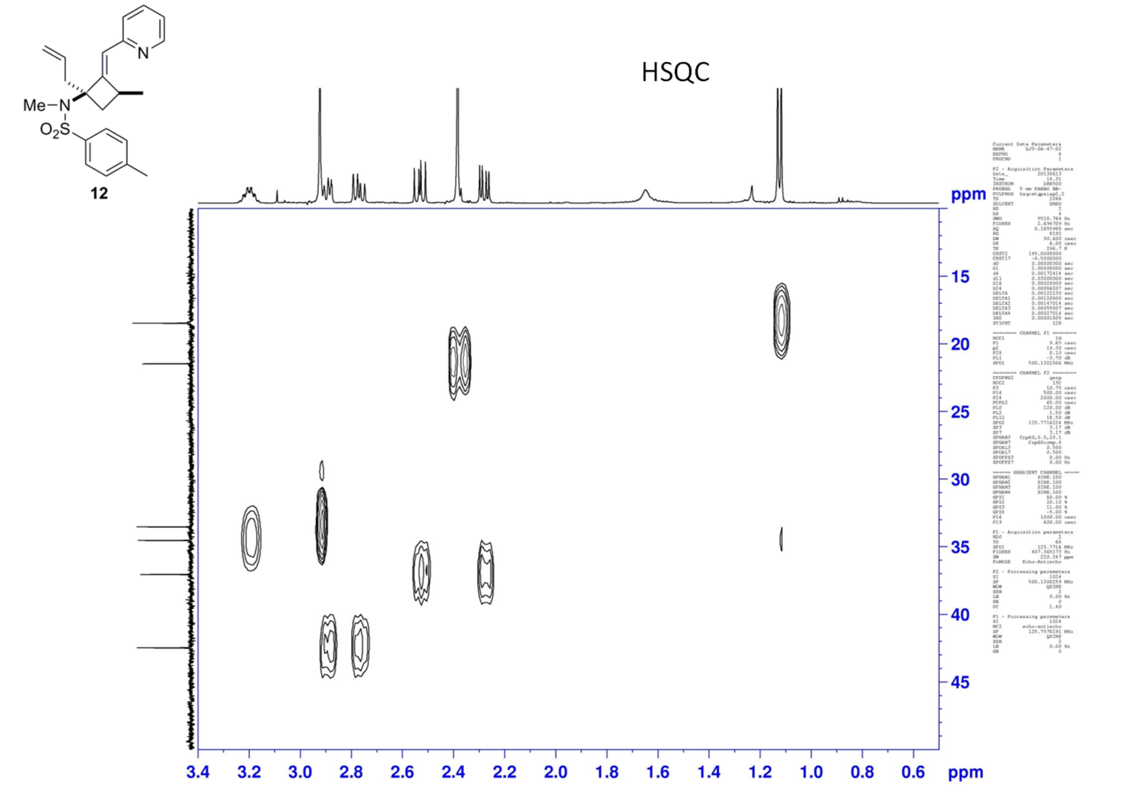
HSQC
Me–N
O_2S
N
12
ppm
15
20
25
30
35
40
45
3.4
3.2
3.0
2.8
2.6
2.4
2.2
2.0
1.8
1.6
1.4
1.2
1.0
0.8
0.6
ppm

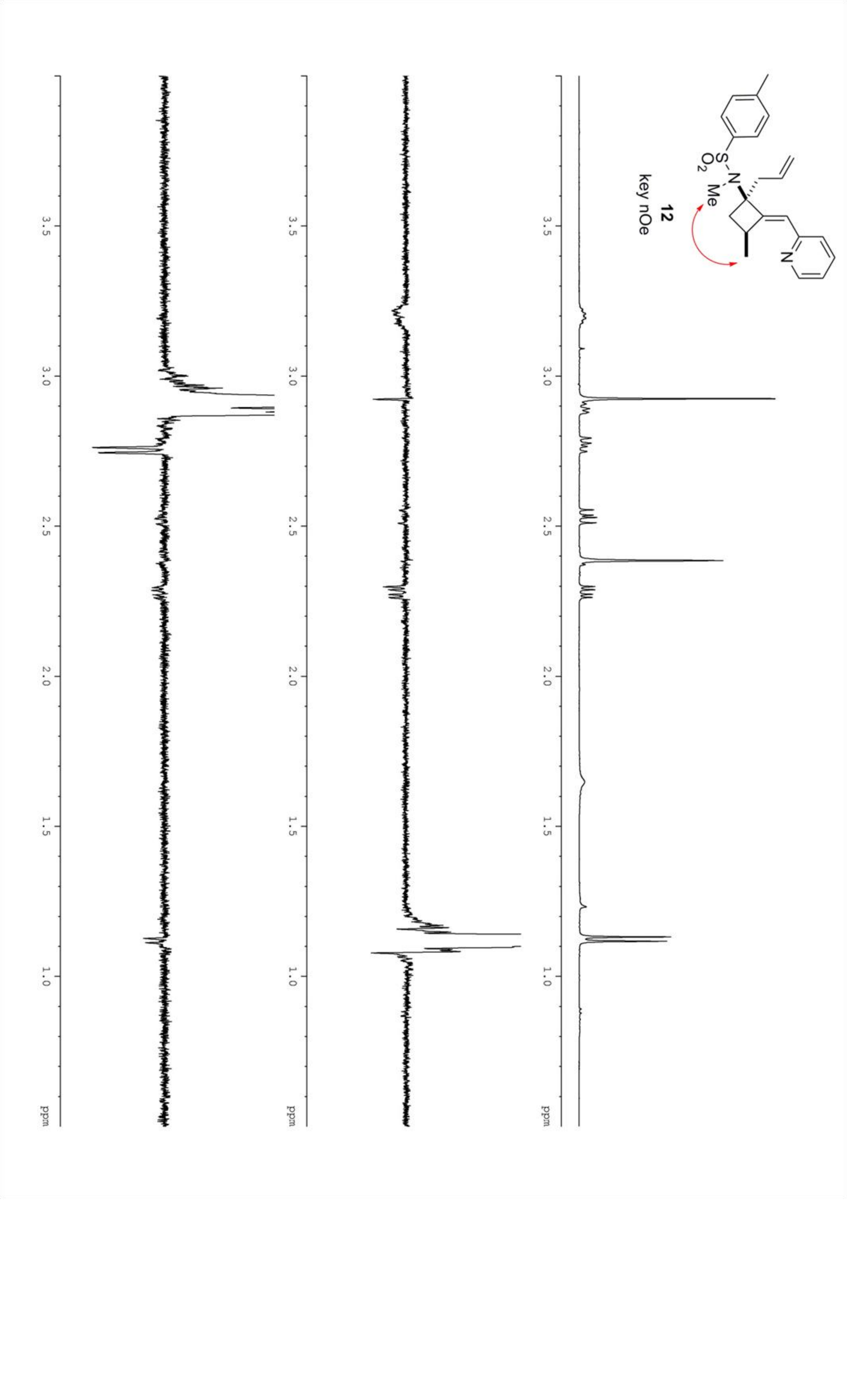
12
key nOe
3.5
3.0
2.5
2.0
1.5
1.0
ppm

Table 1 Crystal data and structure refinement for Hydrochloride salt of 2a

Empirical formula	$C_{20}H_{22}NCl$
Formula weight	311.84
Temperature	100.01°K
Crystal system	orthorhombic
Space group	$Pca2_1$
Unit cell dimensions	a = 7.0429(4) Å α = 90.00°
	b = 14.2625(9) Å β = 90.00°
	c = 16.0706(10) Å γ = 90.00°
Volume	1614.28(17) $Å^3$
Z	4
Density (calculated)	1.283 mg/mm^3
Absorption coefficient	2.038 mm^{-1}
F(000)	664.0
Crystal size	0.3 × 0.145 × 0.045 mm^3
Radiation	Cu Kα (λ = 1.54178)
2Θ range for data collection	6.2 to 144.52°
Index ranges	$-7 \le h \le 8$, $-17 \le k \le 17$, $-19 \le l \le 19$
Reflections collected	17764
Independent reflections	3082[R(int) = 0.0205]
Data/restraints/parameters	3082/1/200
Goodness-of-fit on F^2	1.065
Final R indexes [I>=2σ (I)]	R_1 = 0.0259, wR_2 = 0.0693
Final R indexes [all data]	R_1 = 0.0259, wR_2 = 0.0693
Largest diff. peak/hole	0.24/-0.27 e.$Å^{-3}$
Flack parameter	0.040(10)

Table 2 Crystal data and structure refinement for 2g

Empirical formula	$C_{22}H_{21}NO$
Formula weight	315.40
Temperature	100.01 °K
Crystal system	monoclinic
Space group	$P2_{1/n}$
Unit cell dimensions	a = 7.9736 (14) Å α = 90.00°
	b = 20.850 (4) Å β = 96.38 (2)°
	c = 10.0853(17) Å γ = 90.00°
Volume	1666.3(5) $Å^3$
Z	4
Density (calculated)	1.257 mg/mm^3
Absorption coefficient	0.076 mm^{-1}
F(000)	672.0
Crystal size	0.5 × 0.45 × 0.35 mm^3
Radiation	MoKα (λ = 0.71073)
2Θ range for data collection	3.9 to 52.76°
Index ranges	$-9 \le h \le 9$, $-26 \le k \le 26$, $-12 \le l \le 12$
Reflections collected	17198
Independent reflections	3390 [R_{int} = 0.0379, R_{sigma} = 0.0293]
Data/restraints/parameters	3390/0/301
Goodness-of-fit on F^2	1.084
Final R indexes [I>=2σ (I)]	R_1 = 0.0461, wR_2 = 0.1143
Final R indexes [all data]	R_1 = 0.0510, wR_2 = 0.1185
Largest diff. peak/hole	0.30/-0.41 e.$Å^{-3}$

Crystal structure of **2g**

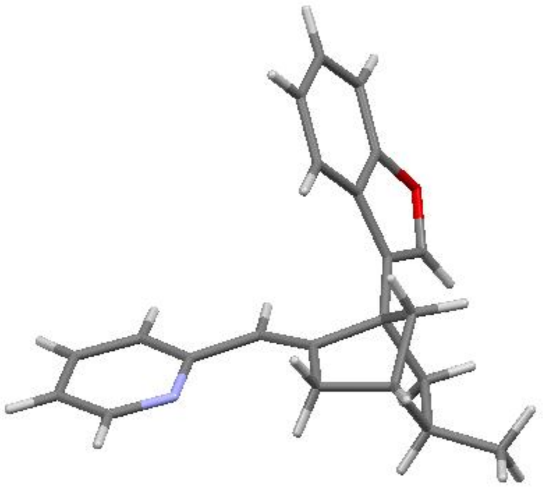

Supplement: Supplementary file 1 [file anie0053-4209-sd1.pdf]
